# The simplest Diels–Alder reactions are not *endo*-selective†

**DOI:** 10.1039/d0sc04553e

**Published:** 2020-10-06

**Authors:** William J. Lording, Thomas Fallon, Michael S. Sherburn, Michael N. Paddon-Row

**Affiliations:** Research School of Chemistry, Australian National University Canberra ACT 2601 Australia michael.sherburn@anu.edu.au; School of Chemistry, University of New South Wales NSW 2052 Australia

## Abstract

There is a widespread perception that the high level of *endo* selectivity witnessed in many Diels–Alder reactions is an intrinsic feature of the transformation. In contrast to expectations based upon this existing belief, the first experimental Diels–Alder reactions of a novel, deuterium-labeled 1,3-butadiene with commonly used mono-substituted alkenic dienophiles (acrolein, methyl vinyl ketone, acrylic acid, methyl acrylate, acrylamide and acrylonitrile) reveal kinetic *endo* : *exo* ratios close to 1 : 1. Maleonitrile, butenolide, α-methylene γ-butyrolactone, and *N*-methylmaleimide behave differently, as does methyl vinyl ketone under Lewis acid catalysis. CBS-QB3 calculations incorporating solvent and temperature parameters give *endo* : *exo* product ratios that are in near quantitative agreement with these and earlier experimental findings. This work challenges the preconception of innate *endo*-selectivity by providing the first experimental evidence that the simplest Diels–Alder reactions are not *endo*-selective. Trends in behaviour are traced to steric and electronic effects in Diels–Alder transition structures, giving new insights into these fundamental processes.

## Abstract 2

Cycloadditions of deuterium-labeled 1,3-butadiene with monosubstituted alkenic dienophiles challenge the widespread assumption of *endo*-selectivity in prototypical Diels–Alder reactions.

## Graphical abstract

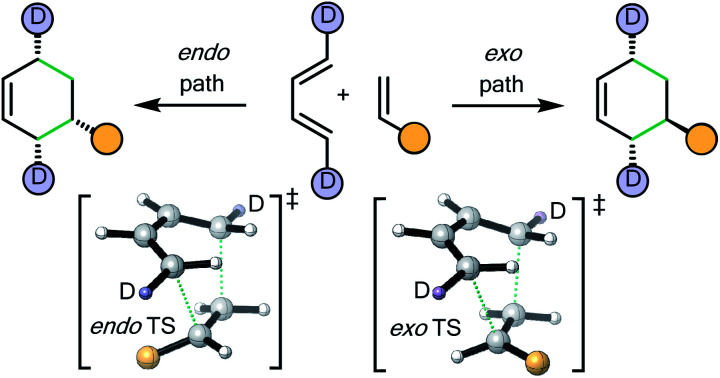

## Introduction

The Diels–Alder (DA) reaction^1^ remains one of the most important reactions in chemical synthesis.^2^ The most well-known pericyclic reaction unites a diene and a dienophile through a concerted, thermally allowed 4 + 2 cycloaddition, which generates a new six membered ring, two new σ-bonds and up to four contiguous stereocenters.^3^ The transformation has found wide application in chemical synthesis by virtue of its tolerance towards substitution and the inclusion of diverse functionality within the diene and dienophile.^4^ The reaction played a central role in the development of theories of organic reactivity, including the conservation of orbital symmetry^5^ and frontier molecular orbital (FMO) theory.^6^ The longevity of the DA reaction is unparalleled: it is as significant today as it was 50 years ago. Indeed, synthetic chemistry would be unrecognizable without it.^7^

**Scheme 1:**
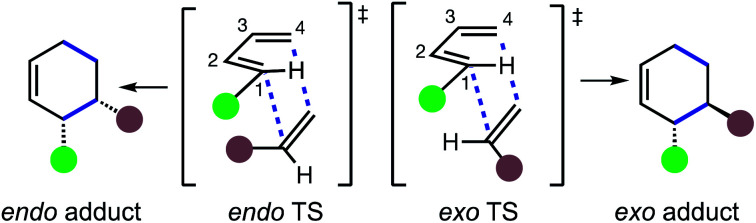
*Endo*/*exo* transition structures (TSs) and products in Diels–Alder (DA) reactions. A requirement for distinct *endo* and *exo*-stereoisomers is two different groups a reacting site in *both* diene and dienophile.

A fundamental attribute of DA reactions between 1,3-butadienes and substituted olefinic dienophiles is the potential for the formation of *endo* and *exo* diastereomeric products. These diastereomeric products result from two distinct transition state structures (TSs) in which a specific dienophile substituent is either closer to (*endo*) or more distant from (*exo*) C2 and C3 of the diene (Scheme 1).

Certain structural requirements must be met in the diene and dienophile for the generation of *endo* and *exo* diastereomers. Specifically, the substituents on at least one of the two ends of the diene (*i.e.* C1 and/or C4) *and* one of the two dienophile carbons must be different. If the dienophile does not fulfil this requirement (*e.g.* ethylene) then *endo*- and *exo*-TSs are not possible. Conversely, if only the diene does not satisfy this condition (as in 1,3-butadiene) then non-equivalent *endo* and *exo*-TSs are generated, but they deliver the same cycloadduct.

Early experimental studies on *endo*/*exo* stereoselectivity in Diels–Alder reactions by Alder and Stein led to the empirical rule of the “maximum accumulation of unsaturation”.^8^ Often cited as the “Alder *endo* rule”, the *endo* mode of addition is favored by dienophiles bearing unsaturated groups in conjugation with the dienophile's reacting double bond (*i.e.*Scheme 1, 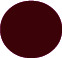 = COR, CN, *etc.*). Various theoretical proposals have been advanced to explain the *endo* selectivity of DA reactions. Secondary orbital interactions (SOIs) are the most widely accepted cause,^9^ and the two most common types are those proposed by Woodward and Hoffmann (WH SOI)^10^ and by Salem and Houk (SH SOI).^11^ The former involves overlap of diene C2 with the carbonyl carbon of the dienophile substituent, and the latter involves overlap of diene C3 with the oxygen of the dienophile carbonyl substituent. Some cycloadditions, for example dimerizations of cyclopentadiene (CPD) and 1,3-butadiene (BD) exhibit bispericyclic TSs, whereupon the SH-type SOI becomes indistinguishable from one of the two σ-bonds being formed.^12^ The origin of *endo*/*exo* selectivity in DA reactions and the existence of SOIs has been debated,^13^ with other types of interactions being invoked to explain *endo*-selective DA reactions, amongst them solvent effects,^14^ electrostatic forces,^15^ and pre-reaction van der Waals forces.^16^

The empirical Alder *endo* rule is successful in predicting the strong *endo*-selectivity of kinetically controlled, thermal DA reactions involving rigid and highly activated cyclic dienophiles such as maleic anhydride and benzoquinone. Its extension to thermal reactions of acyclic dienophiles is less clear cut. Furthermore, there are many counterexamples to the *endo* rule (*i.e.* reactions exhibiting an *exo* preference) and this number continues to grow.^17–20^*Exo*-selectivity is often attributed to diene and dienophile substitution patterns that generate destabilizing steric strain in *endo*-TSs,^21^ although certain catalysts are also effective in promoting *exo*-selective DA reactions.^22^

**Scheme 2:**
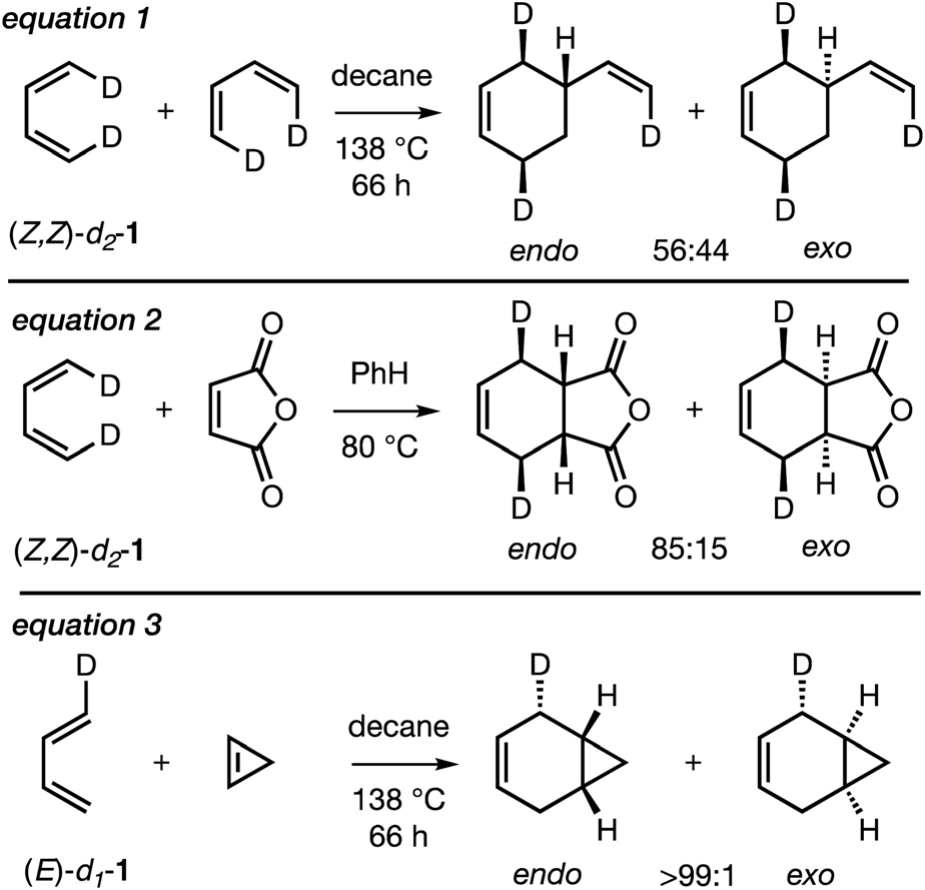
Reported experimental studies on the *endo*/*exo*-selectivity of Diels–Alder reactions involving deuterium-labeled 1,3-butadienes (*Z*,*Z*)-*d*_2_-**1** and (*E*)-*d*_1_-**1**. Both *E*- and *Z*-isomers of 1-deutero-1,3-butadiene (eqn (3)) were used but only one stereoisomer is shown for clarity.

In spite of the large number of mechanistic studies on the Diels–Alder reaction,^3^ only three experimental studies have been carried out on the *endo*/*exo*-selectivity of Diels–Alder reactions involving the parent, archetypal 1,3-butadiene (BD), as summarized in Scheme 2. Thus, the Diels–Alder dimerization of (*Z*,*Z*)-1,4-dideutero-1,3-butadiene (*Z*,*Z*)-*d*_2_-**1** (eqn (1)) was found to be very mildly (*endo* : *exo* = 56 : 44)^23^*endo*-selective, whereas the Diels–Alder reaction of (*Z*,*Z*)-*d*_2_-**1** with maleic anhydride (eqn (2)) was more strongly *endo*-selective (*endo* : *exo* = 85 : 15)^24^ and the DA reaction between *E*/*Z*-deutero-1,3-butadiene (*E*/*Z*)-*d*_1_-**1** and cyclopropene (eqn (3)) was very strongly *endo*-selective (*endo* : *exo* >99 : 1).^25^ These outcomes were attributed to controlling SOIs.^13*b*,26^

The observations of a large difference in the degree of *endo* selectivity between BD, acting as *dienophile* (Scheme 2, eqn (1)) and the more reactive maleic anhydride and cyclopropene dienophiles (Scheme 2, eqn (2) and (3), respectively) have attracted recent computational investigations employing distortion–interaction or activation-strain methods,^27^ as well as energy decomposition analysis techniques.^28^ In the case of the DA reaction between BD and MA, Fernández and Bickelhaupt attributed high *endo* selectivity to unfavourable steric interactions in the *exo*-TS pathway.^29^ In the case of the cyclopropene BD reaction, Houk and co-workers attribute *endo* selectivity to several factors including favorable CH⋯π SOIs in the *endo*-TS.^30^ The important question of whether *endo*-selective DA reactions of BD are more generally preferred remains open, since DA reactions of BD with a range of alkene dienophiles bearing substituents covering a broad spectrum of electron withdrawing properties have not yet been reported.

Several computational studies bearing on this issue have appeared, dealing largely with acrolein and acrylonitrile dienophiles. Density functional theory (DFT) and *ab initio* MO calculations predict moderate to strong *endo* selectivity for the BD + acrolein Diels–Alder reaction, in the gas phase^31^ and in solution,^32^ the degree of *endo* selectivity being predicted to increase markedly in the Lewis acid catalyzed reaction,^33–35^ a finding that is consistent with simple frontier MO arguments^36^ and those relating to diminished Pauli repulsion between the diene and dienophile π-systems.^37^

Computational results for the Diels–Alder reaction between BD and acrylonitriles are not clear cut. Gas phase Hartree–Fock calculations predict modest *exo* selectivity for the reactions of BD and CPD with acrylonitrile and maleonitrile.^38,39^ The predicted *exo* pathway for the CPD reactions is at variance with the experimentally observed *endo* mode for this diene with acrylonitrile and maleonitrile.^40^ However, inclusion of non-specific solvent effects, in the form of self-consistent reaction field theory, reversed the preferred mode to *endo* for the reaction of both BD and CPD with the two acrylonitriles.^39^ It was concluded that solvent polarity, not SOIs, is responsible for the *endo* selectivity in these reactions,^39^ although the *endo* selectivity for the reactions with BD remained an experimentally untested prediction. As part of a DFT (B3LYP) study of intramolecular Diels–Alder reactions, we investigated substituent effects on *endo*/*exo* selectivities of Diels–Alder reactions between BD and monosubstituted ethylenic dienophiles (CH_2_=CH–Z; Z = CN, CO_2_Me, CO_2_H, NO_2_, CHO, COMe).^41^ It was found that *endo* selectivity is predicted for methyl vinyl ketone and acrolein. However, this finding, like those from earlier studies that used the Hartree–Fock procedure,^38,39^ is unreliable because B3LYP seriously underestimates dispersion energies, thereby skewing the selectivity towards the *exo* reaction channel.

**Fig. 1:**
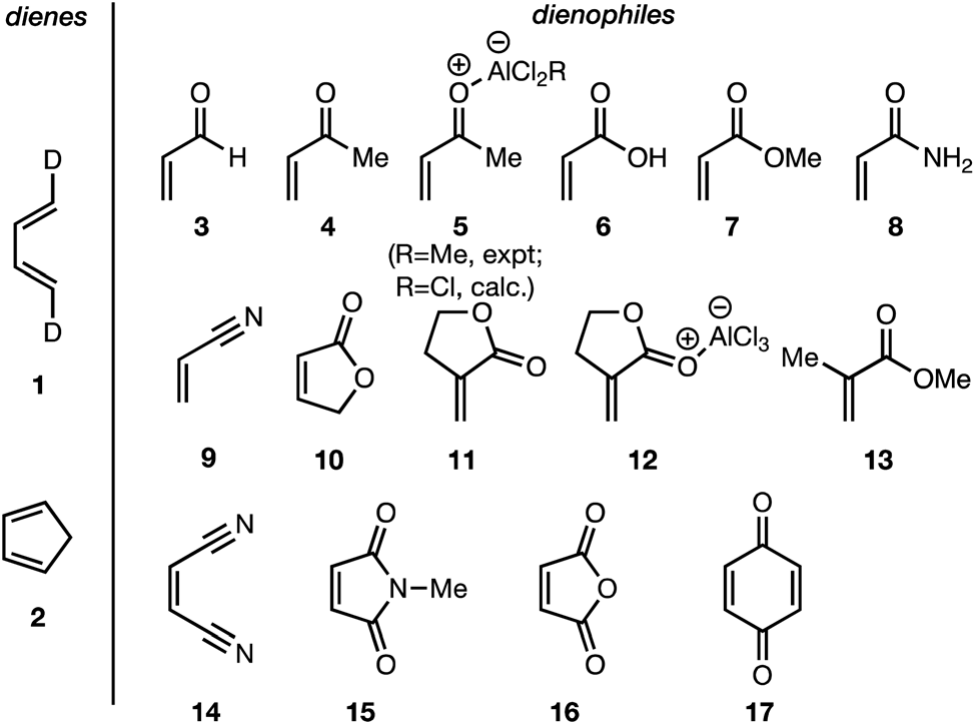
Diels–Alder reactions between the depicted dienes and dienophiles under investigation in this joint experimental–computational study.

In summary, there exists an important gap in our knowledge—both experimental and computational—concerning the *endo*/*exo* selectivity in Diels–Alder reactions involving the most fundamental diene of all, 1,3-butadiene. We have addressed this lacuna and, in this paper, we present the results of our experimental determination of the stereochemical outcomes from the reaction of (1*E*,3*E*)-1,4-dideutero-1,3-butadiene **1** with a wide range of dienophiles (Fig. 1). Also presented are the results of DA reactions between the same dienophiles with CPD,^42^ and a high-level quantum chemical study of these reactions.

## Results and discussion

### Synthesis of (1*E*,3*E*)-1,4-dideutero-1,3-butadiene **1**

**Scheme 3:**
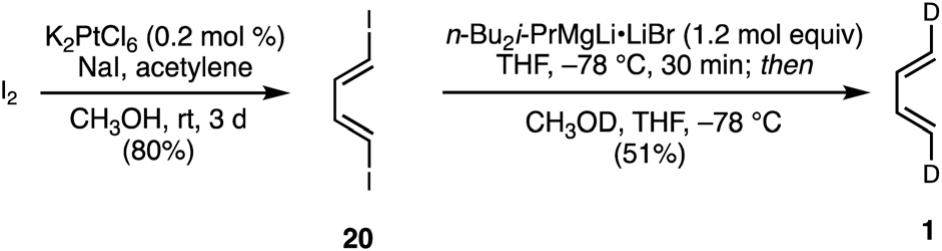
Synthesis of (1*E*,3*E*)-1,4-dideutero-1,3-butadiene.

As discussed above (Scheme 2), previous studies^23–25^ were carried out with (1*E*)- and (1*Z*)-1-deutero-1,3-butadiene, (*E*/*Z*)-*d*_1_-**1**, and (1*Z*,3*Z*)-1,4-dideutero-1,3-butadiene, (*Z*,*Z*)-*d*_2_-**1**. (1*E*)- and (1*Z*)-1-deutero-1,3-butadienes are unsuitable for our purposes, since they would lead to mixtures of regioisomeric products with mono-substituted dienophiles. We elected not to repeat the published synthesis of (1*Z*,3*Z*)-1,4-dideutero-1,3-butadiene^43^ due to the involvement of intricate separations and low yields. Ultimately, we targeted the previously unreported (1*E*,3*E*)-1,4-dideutero-1,3-butadiene, **1**. The requirements for this synthesis would be challenging, since the study mandated access to multigram quantities of this volatile (bp = −4 °C) hydrocarbon in high purity. Our successful two step synthesis of (1*E*,3*E*)-1,4-dideutero-1,3-butadiene **1** is shown in Scheme 3.

Optimization of the reported^44^ Pt(iv)-catalyzed iodinative dimerization of acetylene allowed convenient access to (1*E*,3*E*)-1,4-diiodo-1,3-butadiene **1** in high stereochemical purity on multigram scale. Metal–halogen exchange of di-iodide **20** using Oshima's trialkylmagnesate reagent^45^ followed by deutero-demetalation with MeOD furnished the target (1*E*,3*E*)-1,4-dideutero-1,3-butadiene **1** in a highly stereoretentive manner (>95% 1*E*,3*E*- and >90% *d*_2_). Following purification, this compound was kept as a benzene or CH_2_Cl_2_ solution for ease of storage and handling.

### Diels–Alder reactions

Uncatalyzed cycloaddition reactions between the new, labeled 1,3-butadiene **1** and the dienophiles acrolein **3**, methyl vinyl ketone **4**, acrylic acid **6**, methyl acrylate **7**, acrylamide **8**, acrylonitrile **9**, maleonitrile **14**, butenolide **10**, α-methylene γ-butyrolactone **11** and *N*-methylmaleimide **16** were carried out in benzene solution, and the results are summarized in Table 1. Experimental *endo* : *exo* ratios were determined by quantitative 800 MHz ^1^H NMR spectroscopy.^46,47^ The majority of reactions were carried out with a 1 : 1 molar ratio of starting diene and dienophile at 1 M concentrations in sealed tubes at 145 °C, the exceptions being the more reactive dienophiles maleonitrile **14** (100 °C) and *N*-methylmaleimide **15** (20 °C).

**Table 1:** *Endo*/*exo* selectivities of DA reactions of commonly used dienophiles with (1*E*,3*E*)-1,4-dideutero-1,3-butadiene **1** and cyclopentadiene **2**

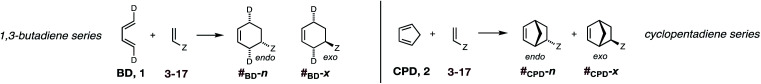									
Dienophile	Diene	Adducts	Temp. (°C)	Time	Isolated yield (%)	*Endo* : *exo* (exp.)	*Endo* : *exo* (calc.)^a^	(s-*cis endo* : s-*trans endo*) : (s-*cis exo* : s-*trans exo*) (calc.)	HOMO_diene_–LUMO_dienophile_ (eV)
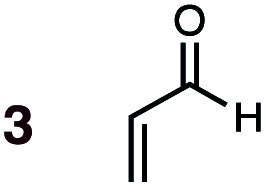	**1**	**3BD-n**, **3BD-x**	145	20 h	37	64 : 36	86 : 14	(0.0 : 5.6) : (4.7 : 9.0)	4.4
	**2**	**3CPD-n**, **3CPD-x**	80	70 min	69	73 : 27	81 : 19	(0.0 : 3.4) : (3.9 : 5.9)	4.0
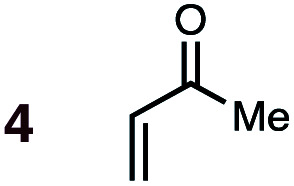	**1**	**4BD-n**, **4BD-x**	145	24 h	64	65 : 35	64 : 36	(0.0 : 7.8) : (1.4 : 11.1)	4.7
	**2**	**4CPD-n**, **4CPD-x**	80	20 min	83	79 : 21	86 : 14	(0.0 : 8.3) : (4.5 : 16.4)	4.3
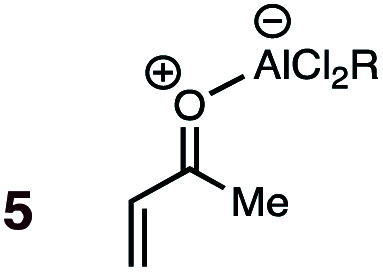	**1**	**5BD-n**, **5BD-x**	−78 to rt	20 h	83	>95 : 5^b^	99 : 1	(0.0 : 17.0) : (13.0 : 27.1)	2.4
	**2**	**5CPD-n**, **5CPD-x**	—	—	—	—	—	—	—
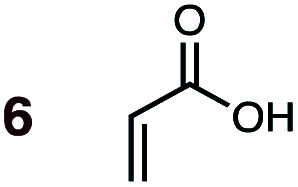	**1**	**6BD-n**, **6BD-x**	145	40 h	90	60 : 40	60 : 40	(0.0 : 4.8) : (1.1 : 5.2)	4.8
	**2**	**6CPD-n**, **6CPD-x**	80	25 min	94	71 : 29	87 : 13	(0.0 : 5.7) : (4.7 : 10.2)	4.4
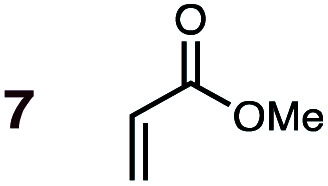	**1**	**7BD-n**, **7BD-x**	145	90 h	83	50 : 50	67 : 33	(0.0 : 4.7) : (1.9 : 5.7)	5.0
	**2**	**7CPD-n**, **7CPD-x**	80	6.5 h	86	77 : 23	76 : 24	(0.0 : 4.2) : (2.6 : 8.9)	4.6
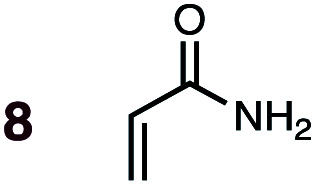	**1**	**8BD-n**, **8BD-x**	145	120 h	9	46 : 54	35 : 65	(1.8 : 7.2) : (0.0 : 11.1)	5.3
	**2**	**8CPD-n**, **8CPD-x**	80	25 min	15	64 : 36	76 : 24	(0.0 : 7.4) : (2.8 : 14.8)	4.9
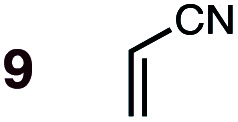	**1**	**9BD-n**, **9BD-x**	145	120 h	76	37 : 63	44 : 56	0.6 : 0.0	4.6
	**2**	**9CPD-n**, **9CPD-x**	80	16 h	87	55 : 45	60 : 40	0.0 : 1.0	4.2
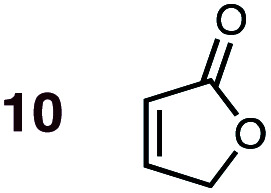	**1**	**10BD-n**, **10BD-x**	145	120 h	27	73 : 27	82 : 18	0.0 : 3.9	4.9
	**2**	**10CPD-n**, **10CPD-x**	80	68 h	40	80 : 20	90 : 10	0.0 : 5.3	4.5
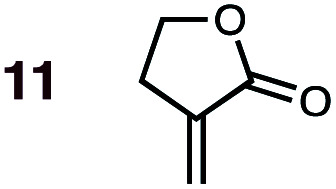	**1**	**11BD-n**, **11BD-x**	145	20 h	87	39 : 61	27 : 73	2.5 : 0.0	4.9
	**2**	**11CPD-n**, **11CPD-x**	80	4 h	72	12 : 88	10 : 90	5.3 : 0.0	4.5
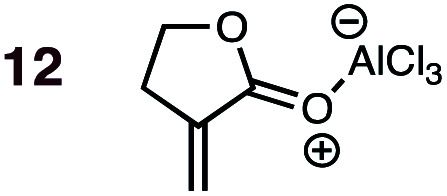	**1**	**12BD-n**, **12BD-x**	—	—	—	—	89 : 11	0.0 : 5.1	3.0
	**2**	**12CPD-n**, **12CPD-x**	−15 °C	—	72	6 : 94^c^	11 : 89	5.2 : 0.0	—
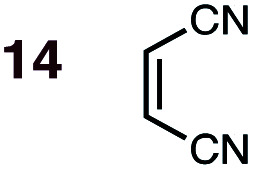	**1**	**14BD-n**, **14BD-x**	100	24 h	75	70 : 30	66 : 43	0.0 : 1.7	3.2
	**2**	**14CPD-n**, **14CPD-x**	20	2 h	81	73 : 27	59 : 41	0.0 : 1.0	2.8
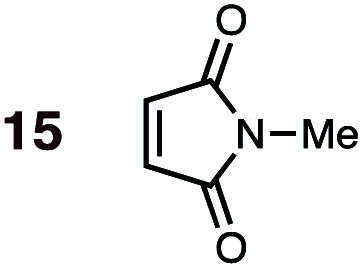	**1**	**15BD-n**, **15BD-x**	20	24 h	92	>99 : 1	99 : 1	0.0 : 10.9	3.5
	**2**	**15CPD-n**, **15CPD-x**	20	15 min	98	>99 : 1	>99 : 1	0.0 : 14.9	3.1
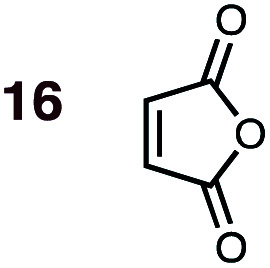	**1**	**16BD-n**, **16BD-x**	80	—	—	85 : 15^d^	98 : 2	0.0 : 9.4	3.0
	**2**	**16CPD-n**, **16CPD-x**	25	—	—	99.5 : 0.5^d^	97 : 3	0.0 : 8.6	2.6
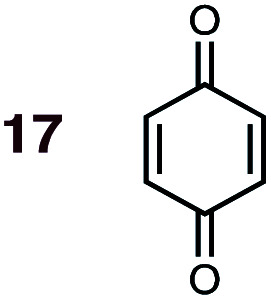	**1**	**17BD-n**, **17BD-x**	—	—	—	—	98 : 2	0.0 : 9.4	2.6
	**2**	**17CPD-n**, **17CPD-x**	20	1 h	80	>99 : 1^e^	>99 : 1	0.0 : 12.7	2.2
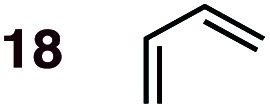	**1**	**18BD-n**, **18BD-x**	138	55 h	66	56 : 44^f^	—	—	—
	**2**	**18CPD-n**, **18CPD-x**	—	—	—	—	—	—	—
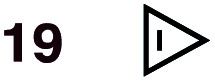	**1**	**19BD-n**, **19BD-x**	0	2 h	—	>99 : 1^g^	—	—	—
	**2**	**19CPD-n**, **19CPD-x**	0	—	97	>99 : 1^h^	—	—	—

a Relative Δ*G*^‡^, CBS-QB3, benzene phase. Calculated at the experimental temperature where available.

b Experiment conducted with MeAlCl_2_.

c Experimental result from Buono and coworkers.^18^

d Experimental results from Stephenson and coworkers.^24^

e Experimental result from Mal and Ray.^57^

f Experimental result from Stephenson *et al.* and Klärner *et al.*^23^

g Experimental result from Baldwin and Reddy.^25^

h Experimental result from Wiberg and Bartley.^58^

The DA reactions of CPD with the same ten dienophiles were carried out in the same manner, except at a lower temperature. Thus, a 1 : 1 molar ratio of CPD and dienophile (1 M concentration solutions of both diene and dienophile in benzene) were heated at 80 °C in the majority of cases, with the DA reactions of the more reactive dienophiles maleonitrile **14** and *N*-methylmaleimide **15** being conducted at 20 °C and the DA reactions of the three least reactive dienophiles employing 3 molar equivalents of CPD.

The majority of DA reactions proceeded cleanly and smoothly, the exceptions being those involving the dienophile acrylamide **8**, which was very low yielding due to its poor DA dienophile reactivity and competing polymerization, presumably through Michael addition pathways. Butenolide was also poorly reactive, giving rise to low yielding reactions with both BD and CPD. At the other end of the reactivity scale, acrolein **3** was the most reactive of the mono-substituted dienophiles.

A significant increase in reactivity is seen with dienophiles carrying two activating groups: compare, for example, the reaction temperatures and times of acrylonitrile **9** and maleonitrile **14** with a specific diene, either BD or CPD. As can be seen from inspection of Table 1, uncatalyzed DA reactions in benzene solution are significantly more facile with CPD than with BD. A MeAlCl_2_-catalyzed (1.1 mol equiv.) reaction between BD and methyl vinyl ketone (*cf.***5**, Fig. 1) was performed at −78 °C with slow warming to ambient temperature over 20 h. This reaction delivered an 83% yield of only the *endo* product, within the limits of detection (>95 : 5).

For representative DA reactions involving BD and CPD, different *endo* : *exo* ratios of selected products were exposed to the reaction conditions under which they were formed. An unchanged ratio was returned in each case, confirming the kinetic control of these reactions.^47^ The reaction between labeled 1,3-butadiene **1** and acrylonitrile **9** was also performed at 350 °C in the gas phase, which led to a 50 : 50 mixture of *endo* : *exo* isomers. This result is consistent with a thermodynamically-controlled process.^47^

A list of additional DA diene–dienophile combinations that were studied computationally are listed in the following Computational methodology section. A small group of previously published experimental and computational results are also included in Table 1.

### Computational methodology

Optimized geometries of reactants and transition structures (TSs) and their energies were calculated using the composite *ab initio* CBS-QB3 method, which is a member of the complete basis set methods developed by Petersson *et al.*^48,49^ The CBS-QB3 method uses a B3LYP/6-31G^†^ optimized geometry and frequencies together with CCSD(T), MP4SDQ, and MP2 single-point calculations and a CBS extrapolation to produce accurate energies. The CBS-QB3 method successfully calculates reliable energetics of pericyclic reactions, including DA reactions.^50–53^ The calculations were carried out for both gas phase reactions and in benzene solvent using the polarizable continuum model (PCM).^54,55^ Standard states used for calculating free energies were 1 atmosphere of pressure and 298.15 K for gas phase reactions and 1 M for solution phase reactions. Gas phase and solution phase reactions were modeled and the latter data are in better agreement with experimental findings. Solution phase reactions were modeled at both 298.15 K and at the experimental reaction temperature: while both were in good agreement with experimental values, the latter were closer.

To check the reliability of the optimized geometries, energies and *endo* : *exo* ratios, geometry optimizations were also performed on representative examples using the B3LYP-D3 method with single-point energy refinements then computed at the CCSD(T) level. The TS geometries are extremely close for the two methods, with root-mean-square deviations of only 0.017–0.038 Å across the 8 TSs located. The calculated single point energies and *endo*/*exo* ratios are also very similar. See the ESI for details.† Calculations were carried out using the Gaussian 03 or Gaussian 16 packages.^56^

DA reactions of both BD **1** and CPD **2** with the following fifteen dienophiles were studied: acrolein **3**, methyl vinyl ketone (MVK) **4**, acrylic acid **6**, methyl acrylate **7**, acrylamide **8**, acrylonitrile **9**, maleonitrile **14**, butenolide **10**, α-methylene γ-butyrolactone **11**, *N*-methylmaleimide (NMM) **15**, methyl vinyl ketone·AlCl_3_**5**, α-methylene γ-butyrolactone·AlCl_3_**12**, maleic anhydride (MA) **16**, benzoquinone (BQ) **17**, methyl methacrylate **13**. The first ten dienophiles on this list were also examined experimentally. The computed DA reaction of methyl vinyl ketone·AlCl_3_ serves as a simulacrum for the experiment performed with MeAlCl_2_ as catalyst. The other DA reactions were calculated to benchmark with literature results, or to provide predictions.

For acyclic dienophiles with conjugating carbonyl groups, specifically **3**, **4**, **6**, **7** and **8**, s-*cis* and s-*trans* conformations of the dienophilic enone group are possible for both *endo*- and *exo*-modes of cycloaddition. In these instances, the calculated *endo* : *exo* ratios factor in the relative Boltzmann contributions of each of the four TSs. As can be seen from the data in Table 1, TSs with s-*cis* conformations are generally preferred.

Additional information is presented in Tables S1 and S2 in the ESI† for DA reactions with BD and CPD, respectively. This includes CBS-QB3 relative *H*^‡^_298 K_ and *G*^‡^_298 K_ energies (kJ mol^−1^) in both the gas phase and benzene phase, along with calculated *endo* : *exo* product distributions for the thirty DA reactions described above (fifteen each for BD and CPD). B3LYP/6-31G* calculated HOMO_diene_–LUMO_dienophile_ energy gaps, as well as atomic polar tensor (APT) charge transfer (CT) from diene to dienophile in TS, and TS dipole moments, in gas phase and benzene phase DA TSs for the thirty DA reactions described above (fifteen each for BD and CPD) are also presented.

**Fig. 2:**
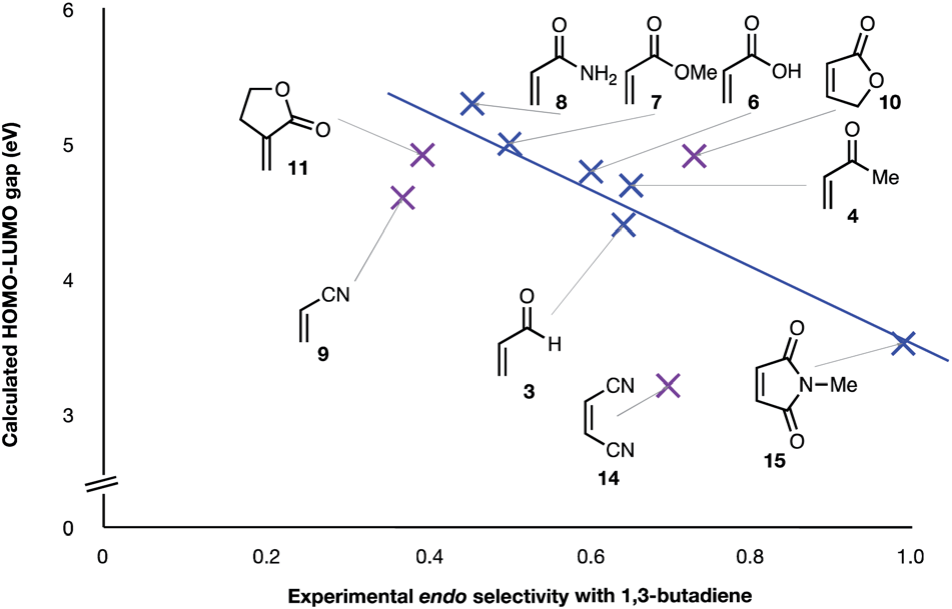
Experimental *endo* selectivities of ten common dienophiles with 1,3-butadiene and cyclopentadiene (*x* axis) *vs.* HOMO_diene_–LUMO_dienophile_ gap. Weaker correlations are colored purple.

**Fig. 3:**
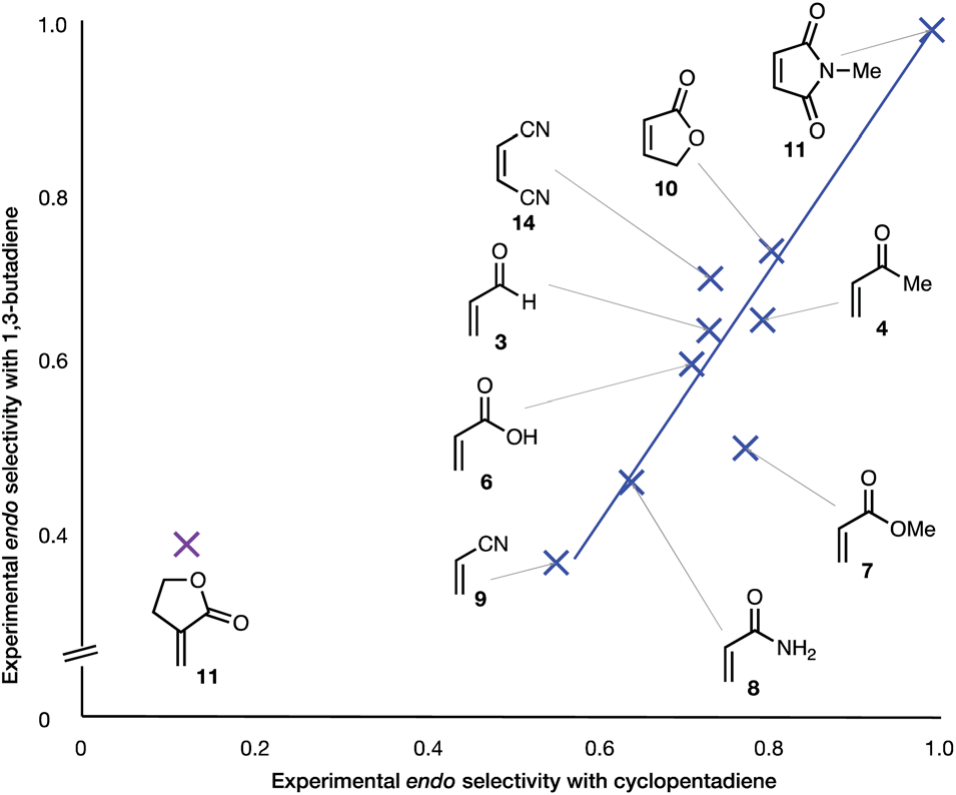
Experimental *endo* selectivities of ten common dienophiles with 1,3-butadiene (*y* axis) and cyclopentadiene (*x* axis), showing an approximate correlation in all but one case.

The following seven observations based upon the data in Table 1 are noteworthy:

(1) The CBS-QB3 method gives calculated *endo* : *exo* product ratios that are generally in very good agreement with experimental data. The agreement between calculated selectivities and these new experimental outcomes validates the theoretical framework of these studies. Benzene phase calculations generally give ratios that are closer to the experimental values than gas phase calculations, although noteworthy (*ca.* 10–15%) differences in *endo* : *exo* ratios between gas and benzene phases are seen only in the cases of acrylamide **8**, acrylonitrile **9** and maleonitrile **14**. A higher *endo*-selectivity is predicted in solution in every case (Tables S1 and S3†). Calculated *endo* : *exo* product ratios from ΔΔ*G*^‡^ values corrected to the experimental temperature are also generally closer than are those calculated at 298 K. The widest disparity between experiment and calculation, in energetic terms, is that involving the previously reported experimental result between MA **16** and BD in PhH at 80 °C (ref. 24) (Scheme 2, eqn (3)), with the experimental *endo* : *exo* value lower (85 : 15) than our calculated value (98 : 2). In light of (a) the closer correlation between calculated and experimental values in other cases, and (b) the much higher *endo*-selectivity observed with the closely-related dienophiles NMM **15** and BQ **17** (see point 2), we suspect that this previously published experimental value is erroneous.^59^

(2) Dienophiles carrying two electron withdrawing groups are more strongly *endo*-selective than are those with one. The three cyclic dienophiles NMM **15**, MA **16** and BQ **17** give very high *endo*-selectivity with both BD and CPD, while the acyclic dienophile maleonitrile **14**, with two *cis*-disposed and powerfully electron-withdrawing cyano-groups, is the least selective of these doubly-activated dienophiles (*endo* : *exo* = 70 : 30 with BD; *endo* : *exo* = 73 : 27 with CPD).

(3) The six mono-substituted dienophiles acrolein **3**, methyl vinyl ketone **4**, acrylic acid **6**, methyl acrylate **7**, acrylamide **8**, acrylonitrile **9** do not display strong selectivity with BD, giving *endo* : *exo* ratios in the range 65 : 35 to 37 : 63. We note, however, that *endo*-selectivity generally diminishes with decreasing electron-withdrawing power, and acrolein **3**, methyl vinyl ketone **4**, acrylic acid **6**, methyl acrylate **7**, and acrylamide **8** show an correlation between higher *endo*-selectivity and smaller HOMO_diene_–LUMO_dienophile_ gap (Fig. 2). Dienophiles with two electron-withdrawing groups (point 2) have the smallest HOMO_diene_–LUMO_dienophile_ gap, and the highest *endo*-selectivity. Acrylonitrile **9** and maleonitrile **14**, with relatively small HOMO_diene_–LUMO_dienophile_ gaps, give anomalously low amounts of *endo*-products.

(4) Both acrylonitrile **9** (*endo* : *exo* = 37 : 63) and α-methylene γ-butyrolactone **11** (*endo* : *exo* = 39 : 61) show an *exo* preference in their DA reactions with BD (Fig. 2). The former is consistent with earlier gas phase calculations on the DA reaction between BD + acrylonitrile,^38,39^ but not with those which included electrostatic solvent effects.^39^ The preferred *exo* selectivity of α-methylene γ-butyrolactone **11** has been noted previously in its reactions with the bis-TMS ether of (2*E*,4*E*)-hexa-2,4-diene-1,6-diol^20^ and with CPD.^18^ Parenthetically, the *exo*-selectivity of the **11** + CPD DA reaction remains high under catalysis with AlCl_3_ (*i.e.***12** + CPD), both in calculation and experiment, whereas the **11** + BD reaction is calculated to undergo a switch to strong *endo*-selectivity under AlCl_3_ catalysis (*i.e.***12** + BD).

(5) Whereas the Diels–Alder reaction of methyl acrylate **7** with BD is non-stereoselective (*endo* : *exo* = 50 : 50), the corresponding reaction of butenolide **10** is *endo* favored (*endo* : *exo* = 73 : 27) (Table 1). This stronger *endo* preference of butenolide **10** over methyl acrylate **7** is evident in the case of CPD as diene, although the latter dienophile also now displays some degree of *endo* selectivity (butenolide **10**: *endo* : *exo* = 80 : 20; methyl acrylate **7**: *endo* : *exo* = 77 : 23).

(6) With the exception of α-methylene γ-butyrolactone **11**, the *endo* : *exo* ratio increases upon change of diene from BD to CPD. In the case of the α-methylene γ-butyrolactone **11**, the percentage *endo* product falls dramatically upon this change, from 39% to 12% (Fig. 3).

(7) A significant enhancement in *endo* preference upon Lewis acid activation is seen in the MeAlCl_2_-promoted DA reaction between labeled BD and methyl vinyl ketone (*cf.***5** + BD). When performed uncatalyzed at 145 °C, this reaction (**4** + BD) gives a 65 : 35 *endo* : *exo* ratio, whereas the *endo* isomer is essentially the sole product detected in the Lewis acid catalyzed reaction at 20 °C (Table 1).

At the start of our discussion of these observations, we note that, in the reactions of monosubstituted dienophiles with BD and CPD, the ΔΔ*G*^‡^ values between *endo* and *exo* pathways are less than 3.5 kJ mol^−1^ (Table 1, entries 1–6). This small energy difference makes the deconvolution of the various contributions to stereocontrol impossible. Our calculations of diene → dienophile charge transfer and dipole moments of TS (see Tables S5 and S6†) neither provided insights into the origins of the selectivity trends described above, nor explanatory information pertaining to the outlying results.

The two most general correlations found in the experimental and computational data are highlighted in Fig. 2 and 3. Fig. 3 shows that, with one exception, the *endo*/*exo*-selectivity of DA reactions between dienophiles and both BD and CPD follow similar trends, albeit with slightly higher *endo*-preferences for CPD. A second general correlation (albeit a rough one) is between the magnitude of the *endo*-stereoselectivity and the size of the HOMO_diene_–LUMO_dienophile_ energy gap (Fig. 2). We previously noted this trend in a broad scope DFT (B3LYP) study (see Introduction).^41^ This observation of enhanced *endo*-stereoselectivity with a smaller HOMO_diene_–LUMO_dienophile_ energy gap is suggestive of SOIs, although this correlation does not constitute evidence of causation.

The results most worthy of brief discussion are exceptions to these general trends, specifically: (a) the anomalously high proportion of *exo*-adducts from BD and CPD DA reactions involving maleonitrile **9** and acrylonitrile **14**; (b) the enhanced *endo*-selectivity of butenolide **10** over methyl acrylate **7**; and (c) the *exo*-selectivity of uncatalyzed DA reactions of α-methylene γ-butyrolactone **11**, and the divergent stereoselectivities of this dienophile in catalyzed DA reactions (*i.e.***12**) with BD and CPD.

### On the anomalous behavior of maleonitrile **9** and acrylonitrile **14**

**Fig. 4:**
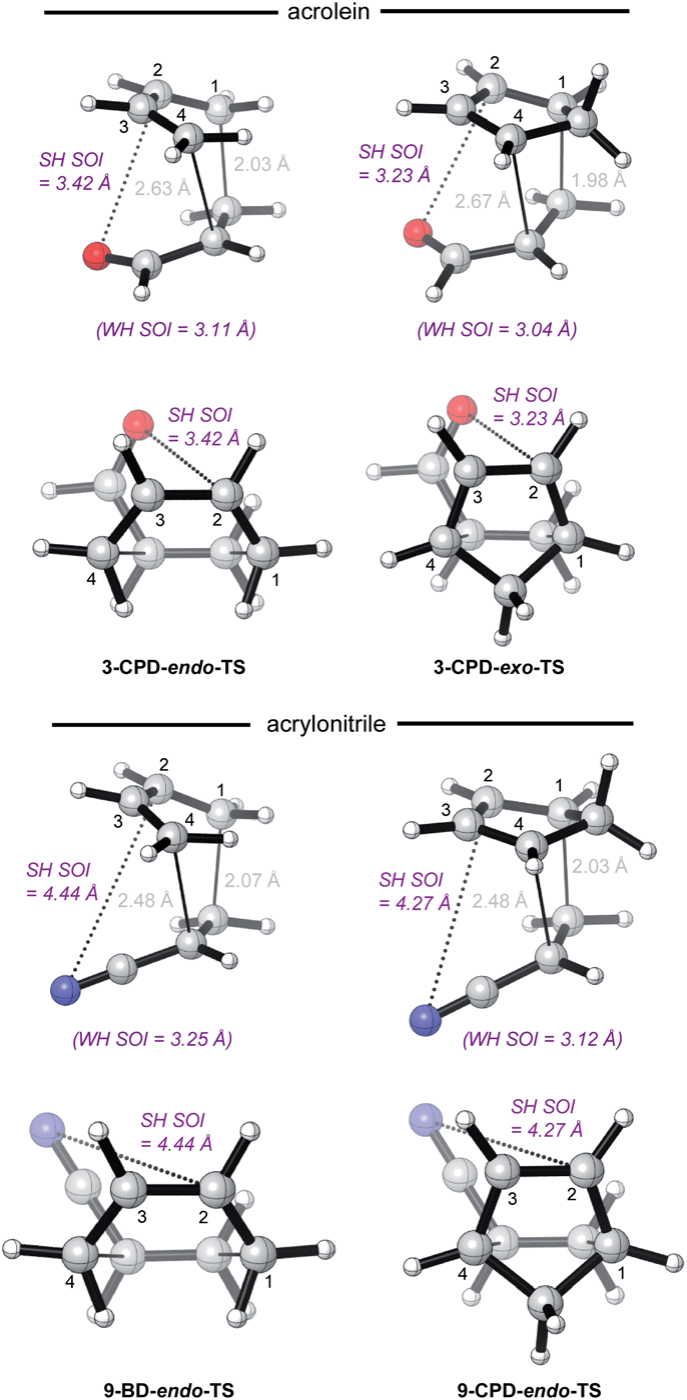
CBS-QB3 *endo*-TSs of uncatalyzed DA reaction of acrolein **3** and acrylonitrile **9** with BD and CPD, highlighting distances between nuclei that may participate in SH-type SOIs. Top row: perspective view; bottom row: view down the forming C–C bonds, from the diene side of the TS.

The anomalously low *endo*-selectivities of DA reactions of acrylonitrile **9** and maleonitrile **14** with BD and CPD can be accounted for by a lack of Salem–Houk (SH) SOIs in DA *endo*-TSs involving nitriles. The *endo*-TSs of the reactions of acrylonitrile **9** with BD and CPD, along with those of the dominant s-*cis* conformation of acrolein **3**, are depicted in Fig. 4.

A comparison of these TSs shows that, whereas the internuclear distances between the acrolein O and diene C2 in DA TSs involving both BD and CPD are 3.42 and 3.23 Å, respectively, in the corresponding acrylonitrile **9** TSs, the N to diene C2 distances are significantly longer, at 4.44 and 4.27 Å, hence unlikely to benefit from stabilizing SH SOIs. A similar situation occurs in the *endo*-TSs for the DA reactions of BD and CPD with maleonitrile **14**. As an aside, the slightly shorter distances seen in CPD DA reactions are due to the shorter C1⋯C4 distance in the 1,3-butadiene moiety of CPD (2.33 Å) relative to BD (2.90 Å): the greater splaying in BD is clearly visible from the lower set of structures depicted in Fig. 4. We also note that internuclear distances (*i.e.* carbonyl C/nitrile C to diene C3) for WH SOIs are similar for both systems (3.04–3.25 Å), which is again most apparent from inspection of the lower set of structures depicted in Fig. 4.

### On the enhanced *endo*-selectivity of butenolide 10 over methyl acrylate **7**

**Fig. 5:**
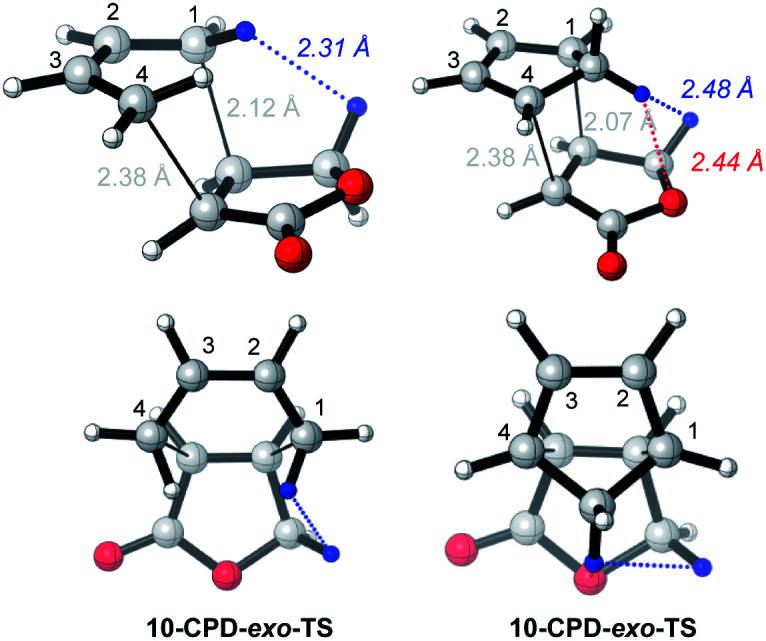
CBS-QB3 *exo*-TSs of uncatalyzed DA reactions of butanolide **10** with BD and CPD, highlighting close contacts. Top row: perspective view; bottom row: view down the forming C–C bonds, from the diene side of the TS.

The enhanced *endo* selectivity of DA reactions between BD/CPD + butenolide **10***vs.* BD/CPD + methyl acrylate **7** is probably the result of destabilizing steric interactions in *exo*-TSs involving butenolide **10** (Fig. 5).

Thus, a close contact is identifiable between a proton on the diene and a butenolide dienophile methylene proton which points toward the diene. In the case of BD, an *inside* methylene proton is close to a butenolide methylene proton (2.31 Å), whereas in the case of CPD, it is the CPD methylene proton directed toward the dienophile that clashes with the same butenolide methylene proton (2.48 Å), in addition to the butenolide ring oxygen (2.44 Å). No such destabilizing steric interaction operates in the *exo*-TSs involving methyl acrylate for two reasons: (a) methyl acrylate lacks an allylic methylene group; and (b) the preferred TSs involving methyl acrylate have s-*cis* C=C–C=O conformations, hence the methoxy group cannot clash with the diene. Parenthetically, a *Z*-crotonate ester is a cognate of butenolide, since it carries an allylic methyl group and prefers an s-*trans* C=C–C=O TS conformation. It is noteworthy that *sec*-butyl *Z*-crotonate was more *endo* selective (by 0.5 kJ mol^−1^) than methyl acrylate in its reaction with CPD.^19^ We propose that similar destabilizing steric interactions are operating in this related system.

### On the anomalous behavior of α-methylene γ-butyrolactone

The exception to the trend of enhanced *endo*-selectivity with CPD *vs.* BD is with the dienophile α-methylene γ-butyrolactone **11**, which instead exhibits enhanced *exo*-selectivity with CPD. Both experimental (published work^18^) and calculated (this work) *Lewis acid catalyzed* versions of these reactions provide additional, intriguing results (Table 1). Whereas AlCl_3_-catalyzed DA reactions of CPD + **12** give the same *exo*-stereoselectivity as the thermal reaction CPD + **11** (*endo* : *exo* = *ca.* 10 : 90), our calculations predict a complete reversal in stereoselectivity for the BD + **11** and BD + **12** reactions (thermal: *endo* : *exo* = 10 : 90; catalyzed: *endo* : *exo* = 88 : 12).

**Fig. 6:**
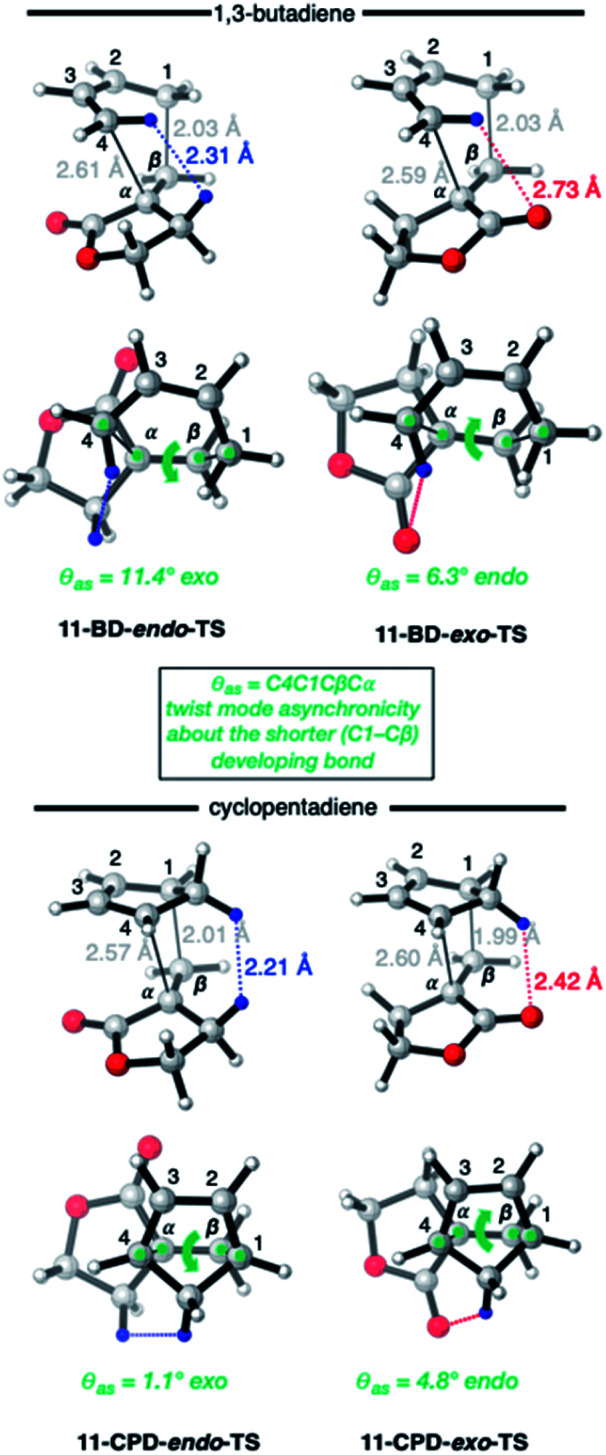
CBS-QB3 *endo*- and *exo*-TSs of the uncatalyzed DA reaction of α-methylene γ-butyrolactone **11** with BD and CPD, highlighting key destabilizing close contacts, and the direction and magnitude of twist-mode asynchronicity. Top row: perspective view; bottom row: view down the forming C–C bonds, from the diene side of the TS.

The origin of these interesting results with α-methylene γ-butyrolactone **11** (and its AlCl_3_ complex **12**) may be traced to geometrical factors in the TSs of the thermal and Lewis acid-catalyzed DA reactions of this dienophile with BD and CPD. Focusing firstly at TSs of the uncatalyzed reactions (Fig. 6), we can see that all TSs have similar length shorter (1.99–2.03 Å) and longer (2.51–2.61 Å) developing bonds, and similar bond forming asynchronicities, Δ*r*_as_ (range = 0.56–0.61 Å).

As expected, the shorter developing bond is to the β-position of the dienophile enone group. In each TS, a close contact between (a) a dienophile methylene proton pointing toward the diene, and (b) a proton on the diene, is identifiable. In the case of BD, the most significant steric clash (2.31 Å) is between an *inside* BD methylene proton and a dienophile **11** allylic methylene proton in the *endo*-TS. An even closer contact (2.21 Å) occurs in the *endo*-TS of the CPD + **11** TS, which involves the CPD methylene proton directed toward the dienophile. Hence, we can explain the *exo*-selectivity of uncatalyzed DA reactions of α-methylene γ-butyrolactone **11** with both BD and CPD by identifying that the *endo*-TSs are disfavored on steric grounds, and that the higher *exo*-selectivity in the CPD case is due to the steric clash being more severe. The ESI† contains further calculations and additional discussion on the influence of methylene lactone ring size on DA selectivity.

**Fig. 7:**
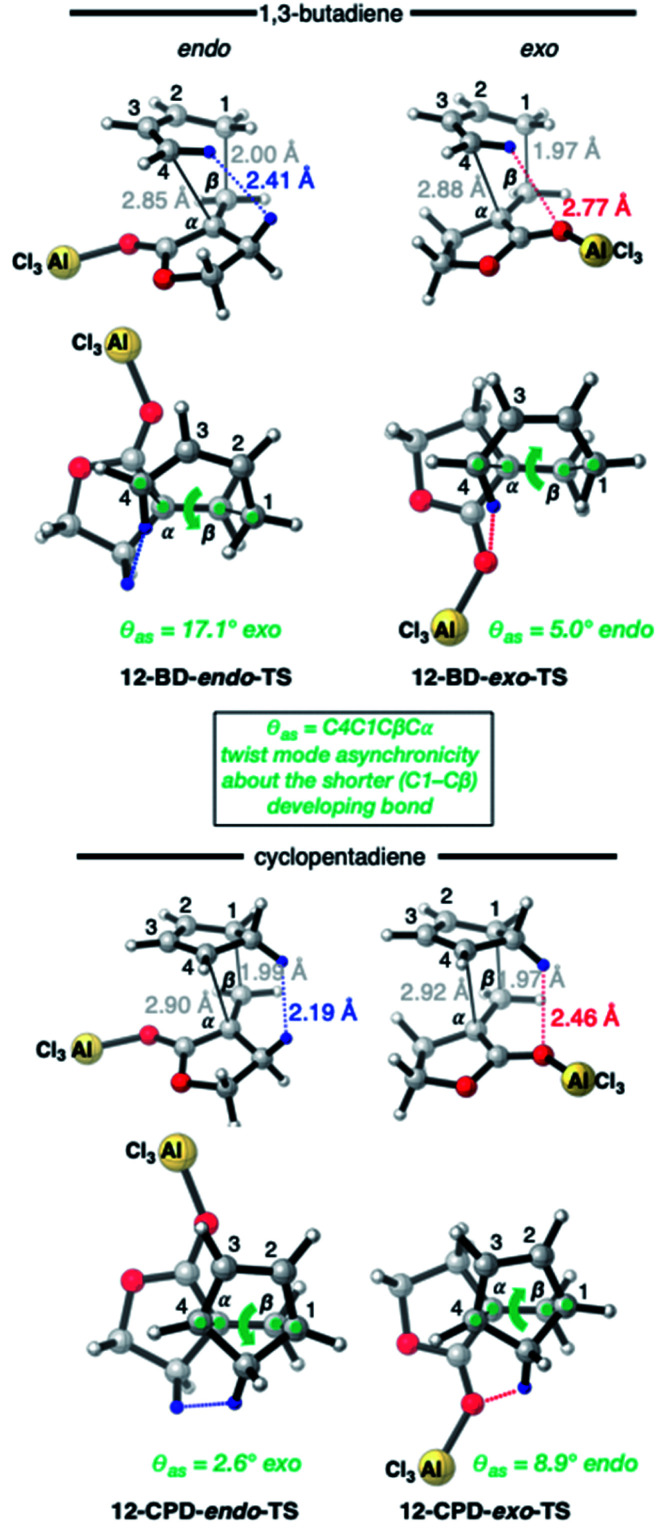
CBS-QB3 *endo*- and *exo*-TSs of the AlCl_3_-catalyzed DA reaction of α-methylene γ-butyrolactone (*i.e.***12**) with BD and CPD, highlighting key destabilizing close contacts, and the direction and magnitude of twist-mode asynchronicity. Top row: perspective view; bottom row: view down the forming C–C bonds, from the diene side of the TS.

The four TSs of the AlCl_3_-catalyzed DA reactions of α-methylene γ-butyrolactone **11** (*i.e.***12**) with BD and CPD are depicted in Fig. 7. Again, similar length shorter (1.97–2.00 Å) and longer (2.85–2.92 Å) developing bonds are seen throughout the four TSs, along with similar bond forming asynchronicities, Δ*r*_as_ (range = 0.85–0.95 Å). A comparison of the catalyzed with the uncatalyzed DA TSs shows that the longer developing bond is significantly extended in the catalyzed reaction. The same close contacts are present in the four catalyzed reaction TSs as in the uncatalyzed ones. In the *endo*-TS of the catalyzed CPD + **12** reaction, the distance (2.19 Å) remains similarly close to that seen in the uncatalyzed CPD + **11** reaction (2.21 Å). Furthermore, in the corresponding *exo*-TS of the same catalyzed and uncatalyzed reactions, the distances are also similar (2.42 and 2.46 Å). Therefore, the similar *exo*-selectivity for both catalyzed and uncatalyzed reactions involving CPD are understandable on the basis of similar steric effects operating in each pair of TSs. In contrast, in the case of catalyzed BD + **12***endo*-TS, the distance between the *inside* BD methylene proton and the dienophile allylic methylene proton is extended (2.41 Å) relative to the uncatalyzed BD + **11***endo*-TS (2.31 Å). We propose that this extension in the BD *endo*-TS alleviates destabilizing steric strain and, perhaps with the assistance of SOIs, is the cause of the switch to *endo*-selectivity under Lewis acid catalysis. The ESI† contains further discussion on the implications of the twist mode asynchronicity differences between the DA TS shown in Fig. 6 and 7.

## Conclusions

In summary, we have conducted the first experimental–computational investigation into the *endo* : *exo* selectivity of Diels–Alder reactions between the simplest diene and ten commonly-used dienophiles. The reactions of cyclopentadiene, one of the most commonly-used dienes, with the same ten dienophiles were also performed. This work was facilitated by the first preparative synthesis of (1*E*,3*E*)-1,4-dideutero-1,3-butadiene in high stereochemical purity, a compound and synthesis that will find application in other investigations.

The most surprising finding from this study is that the most commonly used mono-substituted alkenic dienophiles (acrolein, methyl vinyl ketone, acrylic acid, methyl acrylate, acrylamide and acrylonitrile) are not *endo*-selective in thermal Diels–Alder reactions with 1,3-butadiene. Generally, for a given dienophile, *endo* : *exo* selectivities for cyclopentadiene are *ca.* 5–20% higher than with 1,3-butadiene.

The CBS-QB3 method gives calculated *endo* : *exo* product ratios that are in very good agreement with experimental findings, hence validating the theoretical framework of this study. These models have broader value to those interested in a deeper understanding of the most important synthetic reaction, and its application in synthesis.

## Conflicts of interest

There are no conflicts to declare.

## Supplementary Material

SC-011-D0SC04553E-s001

SC-011-D0SC04553E-s002
