# Supplementary material for: The simplest Diels–Alder reactions are not endo-selective
Source: Chem Sci. 2020 Oct 6;11(43):11915–26. doi: 10.1039/d0sc04553e (PMC8162770; doi:10.1039/d0sc04553e)
Supplement: SC-011-D0SC04553E-s001 [file SC-011-D0SC04553E-s001.pdf]

Electronic Supplementary Material (ESI) for Chemical Science.
This journal is © The Royal Society of Chemistry 2020

Electronic Supplementary Information for:

The Simplest Diels–Alder Reactions are not Endo-Selective

William J. Lording, Thomas Fallon, Michael S. Sherburn* and Michael N. Paddon-Row*

CONTENTS

1 ADDITIONAL DISCUSSION **2**
1.1 ADDITIONAL DISCUSSION ON THE INFLUENCE OF METHYLENE LACTONE RING SIZE ON DIELS-ALDER SELECTIVITY 2
1.2 FURTHER DISCUSSION ON THE IMPLICATIONS OF THE TWIST MODE ASYNCHRONICITY DIFFERENCES BETWEEN THE DIELS-ALDER TSS SHOWN IN FIGURES 6 AND 7 2

2 EXPERIMENTAL SECTION **4**
2.1 GENERAL METHODS 4
2.2 DIELS-ALDER REACTIONS BETWEEN CYCLOPENTADIENE AND COMMON DIENOPHILES. 20

3 ANISOTROPIC DISPLACEMENT ELLIPSOID PLOTS FOR 14_{CPD}-N, AND 14_{CPD}-X **29**

4 REFERENCES **29**

5 COSY AND NOE SPECTRA FOR COMPOUNDS 4_{CPD}-N AND 4_{CPD}-X **30**

6 ^{1}H AND ^{13}C NMR SPECTRA OF COMPOUNDS 1-20 .... **34**

7 COMPUTATIONAL ANALYSIS **94**
7.1 TABLE S1. HOMO-LUMO GAP, RELATIVE $H^{‡}_{298K}$ AND $G^{‡}_{298K}$ (KJ/MOL) AND PRODUCT DISTRIBUTION (PD), AND CHARGE TRANSFER PROPERTIES ARISING FROM VARIOUS STEREOCHEMICAL MODES FOR BUTADIENE WITH DIENOPHILES. 94
7.2 TABLE S2. HOM O-LUMO GAP, RELATIVE $H^{‡}_{298K}$ AND $G^{‡}_{298K}$ (KJ/MOL) AND PRODUCT DISTRIBUTION (PD), AND CHARGE TRANSFER PROPERTIES ARISING FROM VARIOUS STEREOCHEMICAL MODES FOR CYCLOPENTADIENE WITH DIENOPHILES. 95
7.3 GEOMETRY OPTIMISATION AND ENERGY BENCHMARK 96
7.4 EXPLANATION OF HOW A LOWER HOMO-LUMO GAP LEADS TO INCREASED ENDO-SELECTIVITY 98
7.5 CARTESIAN COORDINATES OF OPTIMISED STRUCTURES AND CBS-QB3 ENERGIES 98

1 Additional Discussion

1.1 Additional discussion on the influence of methylene lactone ring size on Diels-Alder selectivity

The calculated TSs for the uncatalyzed (benzene, 25 °C) DA reactions between BD and the four and six-membered analogs of α-methylene γ-butyrolactone **11,** namely α-methylene β-propiolactone **20** and α-methylene δ-valerolactone **21**, predict mild *endo*-selectivity (*endo*:*exo* = 59:41) and moderate *exo*-selectivity (*endo*:*exo* = 28:72), respectively. These TSs (see below) exhibit the same twist asynchronicities and close contacts as those between BD and α-methylene γ-butyrolactone **11**, but with progressively longer developing bonds (ca. 2.4 Å → 2.6 Å → 2.7 Å) as the dienophile ring size is increased. The distance between the *inside* BD methylene proton and the dienophile allylic methylene proton in the *endo*-TS is longest with α-methylene β-propiolactone **20**, which is consistent with its greater *endo*-selectivity, which is associated with the widened angle between the exocyclic C=C bond and adjacent allylic methylene group in **20** relative to **11** and **21**. A stabilizing interaction between an *inside* BD methylene proton and the carbonyl oxygen in the *exo*-TSs may also contribute to the *exo*-preference of the DA reactions between BD and **11** or **21**.

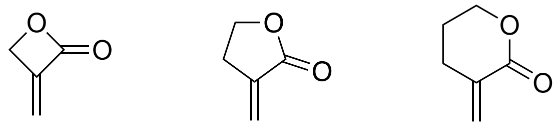

20 **11** **21**

1.2 Further discussion on the implications of the twist mode asynchronicity differences between the Diels-Alder TSs shown in Figures 6 and 7

A final interesting question with this series of DA reactions pertains to why, for the catalyzed reactions, the BD *endo*-TS alleviates steric strain, whereas the CPD *endo*-TS does not, given that both TS have very similar developing bond lengths. The answer to this question lies in the way in which the bond length asynchronicity manifests in the TS geometries. Identifiable within all eight TS of **Figures 6** and **7** (manuscript, for both catalyzed and uncatalyzed reactions) is twist-mode asynchronicity.[1] Specifically, a twist in the *exo*-direction with respect to the shortest developing bond is seen in the four *endo*-TSs, and conversely a twist in the *endo*-direction with respect to the shortest developing bond is seen in the four *exo*-TSs. Twist asynchronicity is most pronounced in the uncatalyzed and catalyzed BD *endo*-TS (twist-mode asynchronicity dihedral angle, θ_{as} = 11.4° and 17.1°, respectively). The large *exo*-twist in the catalyzed BD *endo*-TS functions to alleviate the

[1] (a) Brown, F. K.; Singh, U. C.; Kollman, P. A.; Raimondi, L.; Houk, K. N.; Bock, C. W. *J. Org. Chem.* **1992**, *57*, 4862-4869. (b) Raimondi, L.; Brown, F. K.; Gonzalez, J.; Houk, K. N. *J. Am. Chem. Soc.* **1992**, *114*, 4796-4804. (c) Brown, F. K.; Houk, K. N. *Tetrahedron Lett.* **1985**, *26*, 2297-2300.

aforementioned destabilizing steric strain, by moving the two protons involved in the close contact away from each other. This *exo*-twist also brings the dienophile carbonyl group closer to the diene, thereby permitting stabilizing SOIs. These two factors combine to favor the *endo*-pathway for the catalyzed reaction. In contrast, the uncatalyzed and catalyzed CPD *endo*-TS exhibit only a very small *exo*-twist (θ_{as} = 1.1° and 2.6°, respectively), since in this case, the pivoting of the dienophile in an *exo*-direction about the short developing bond would cause the dienophile to move closer to the CPD methylene group, hence resulting in greater steric strain. Perhaps this inhibition in *exo*-twist asynchronicity of the CPD *endo*-TS also prevents the adoption of a geometry that would enhance SOIs. Irrespective of SOIs, the *exo*-TS remains favored for the catalyzed reaction.

2 Experimental Section

2.1 General Methods

^{1}H NMR spectra were recorded at 800 MHz and 400 MHz using a Bruker AVANCE 800 or Varian 400 spectrometer. Residual protium signals in acetone-d_6 (δ 2.05 ppm), toluene-d_8 (δ 2.08 ppm), DMSO-d_6 (δ 2.50 ppm), C_6D_6 (δ 7.16 ppm) and $CDCl_3$ (δ 7.26 ppm) were used as internal references for ^{1}H NMR spectra recorded in these solvents. Coupling constants (J) are quoted to the nearest 0.1 Hz. ^{13}C NMR spectra were recorded at 200 MHz, 100 MHz using a Bruker AVANCE 800 or Varian 400 instrument. The central line of the solvent triplet acetone-d_6 (δ 2.05 ppm), toluene-d_8 (δ 2.08 ppm), DMSO-d_6 (δ 2.50 ppm), C_6D_6 (δ 7.16 ppm) and $CDCl_3$ (δ 7.26 ppm) was used as internal reference for ^{13}C NMR spectra recorded in this solvent. Assignment of ^{13}C NMR signals was assisted by DEPT or HSQC experiments. IR spectra were recorded on a Perkin–Elmer Spectrum One spectrometer as thin films between sodium chloride plates for oils or as potassium bromide discs for solid products. Low resolution mass spectra were recorded on a Finnigan PolarisQ mass spectrometer using electron impact (EI^+) ionisation mode at 70 eV. High-resolution mass spectra were recorded using VG Autospec operating at 70 eV. Melting points were measured on a Reichert melting point stage and are uncorrected. Analytical TLC was performed using Merck silica gel plates, pre-coated with silica gel 60 F_{254} (0.2 mm). Flash chromatography was conducted using Merck Kiesegel 60 (230 – 400 mesh) silica gel. Commercially available chemicals were purified by standard procedures or used as purchased.

Cyclopentadiene was prepared by cracking dicyclopentadiene. Freshly prepared samples were either used directly or made up to 1.0 M solutions in benzene, which were stored in a –15 °C freezer prior to use. Quoted *endo:exo* ratios refer to values obtained from 800 MHz ^{1}H NMR spectroscopic analysis of crude reaction mixtures and were recorded in duplicate (maximum difference between runs = +/- 0.5%).

Reactions were conducted under a positive pressure of dry argon or nitrogen in flame-dried glassware. Benzene, diethyl ether, tetrahydrofuran and toluene were dried over sodium and distilled from benzophenone ketyl. Dichloromethane was distilled from calcium hydride. Methanol, ethanol, dimethylformamide, dimethyl sulfoxide, chlorobenzene and 1,2-dichlorobenzene were purified by the methods of Armarego and Chai.[1] Commercially available chemicals were purified by standard procedures or used as purchased. Sodium sulfate and magnesium sulphate were dried at 160 °C for 24 h prior to use.

(1*E*,3*E*)-1,4-Diiodo-1,3-butadiene (20)

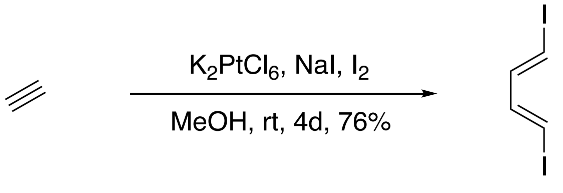

(**20**)

This compound was prepared according to the modified procedure of the Beletskaya group.[2] A stream of acetylene was bubbled through a solution of sodium iodide (3.0 g, 20.0 mmol) in methanol (25 mL) for 10 mins. K_2PtCl_6 (25 mg, 0.051 mmol) and iodine (6.0 g, 23.2 mmol) were added and the mixture was stirred at room temperature under an atmosphere of acetylene (2.5 L, 102 mmol) in darkness for 4 days. The progress of the reaction was judged by the consumption of acetylene. The reaction mixture was diluted with water (50 mL), treated with excess saturated aqueous $Na_2S_2O_5$, and the resulting suspension was stirred vigorously for 5 mins. It was then partitioned between water (700 mL) and diethyl ether (200 mL) and BHT (2 mg) was added. The aqueous layer was discarded, and the organic layer was washed three times with water (100 mL) and once with brine (100 mL), dried over $MgSO_4$ and concentrated under vacuum, giving the title compound (**20**) as a pale yellow solid (5.66 g, 18.5 mmol, 76% based on I_2), contaminated with approximately 2% (103 mg, 0.37 mmol) (*E*)-1,2-diiodoethylene. NMR, MS and IR spectral data all matched the literature values. A single crystal X-ray analysis of the product was also obtained in order to confirm the structure.

(1E,3E)-1,4-dideutero-1,3-butadiene (1)

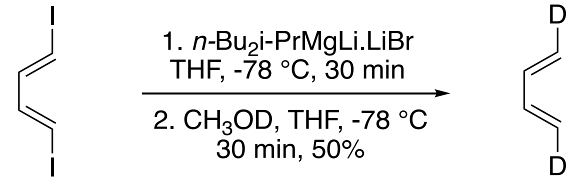

(**20**) (**1**)

This compound was prepared by applying the procedure of Kitigawa and coworkers.[3] A two neck round bottom flask was attached to a nitrogen line fitted with a needle valve and charged with a solution of isopropylmagnesium bromide in THF (19.7 mL, 1.14 M, 22.5 mmol) and an additional 25 mL THF. The contents of the flask were cooled to 0 °C and treated with a solution of *n*-BuLi in hexanes (28.1 mL, 1.6 M, 45.0 mmol). After stirring at 0 °C for 30 mins, the resulting solution was cooled to –78 °C and a solution of (1*E*,3*E*)-1,4-diiodo-1,3-butadiene (**20**) (5.66 g, 18.5 mmol) in THF (5 mL) was added rapidly, and in a single portion. After stirring at –78 °C for 30 mins, CH_3OD (2.64 g, 3.26 mL, 80 mmol) was added, the needle valve was closed and the reaction mixture was stirred at –78 °C for a further 30 mins. One of the necks of the flask was then fitted

with an air condenser connected in sequence to a column packed with cooled, oven dried silica, a liquid nitrogen trap containing 2 mL benzene or CH_2Cl_2, and a bubbler, and the flask was removed from the cooling bath and allowed to warm to room temperature. The contents of the flask were then gently heated to reflux, and once the reaction mixture was at reflux, the needle valve was opened, allowing a slow stream of nitrogen to pass through the headspace of the flask and vent at the bubbler. (1*E*,3*E*)-1,4-dideutero-1,3-butadiene (**1**) (530 mg, 9.5 mmol, 50%) accumulated in the trap and was handled in benzene or CH_2Cl_2 solution (Figure S1). ^{1}H NMR (800 MHz, $CDCl_3$) δ/ppm 6.58-6.52 (m, 2H), 5.44-5.38 (m, 1.94 H) and 5.33-5.29 (m, 0.104 H); ^{13}C NMR (200 MHz, $CDCl_3$) δ/ppm 137.8 (1:1:1 triplet, J = 2.0 Hz) and 117.2 (1:1:1 triplet, J = 24.5 Hz); MS (70 eV, EI): *m/z* (%): 56 (100) $[M]^+$, 40 (70).

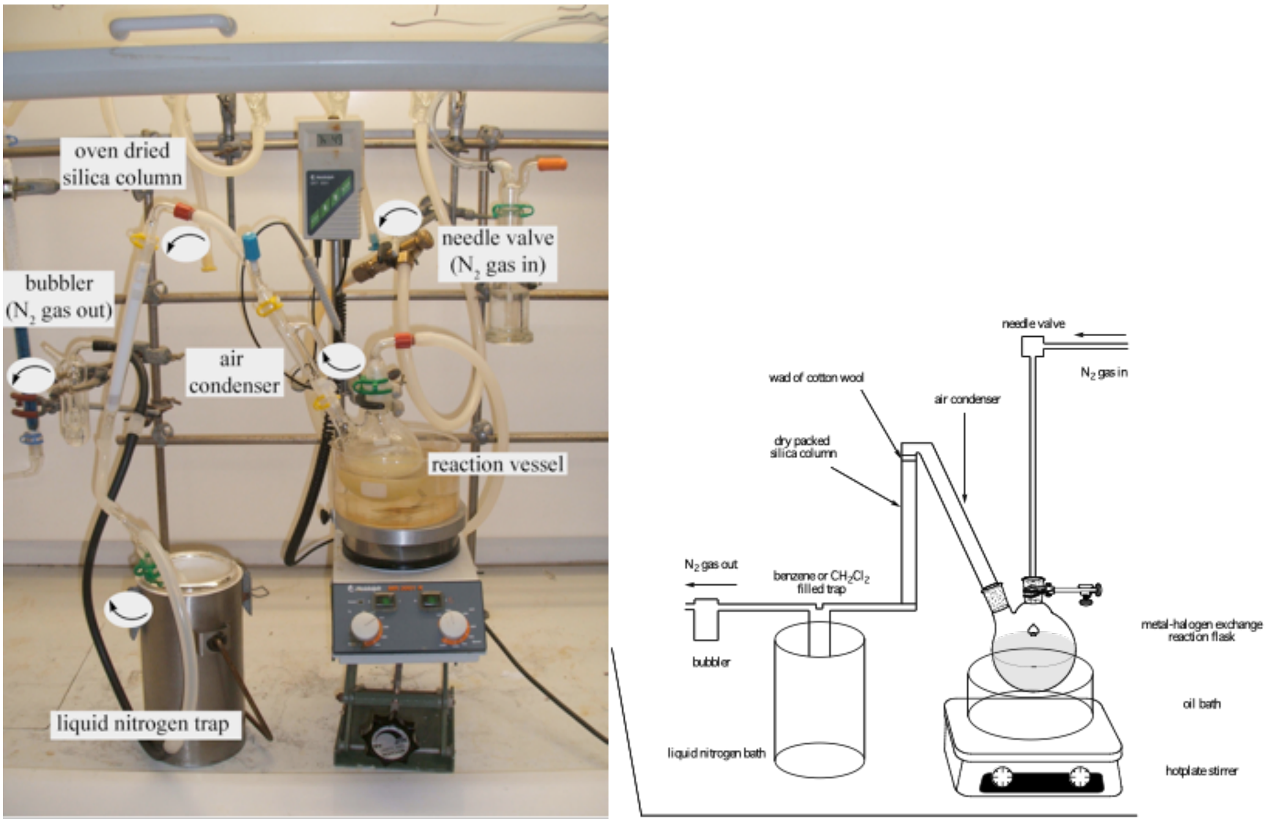

Figure S1. Apparatus for production of solutions of (1*E*,3*E*)-1,4-dideutero-1,3-butadiene (**1**) in benzene or CH_2Cl_2. All joints were wrapped in parafilm and the reaction flask was protected from light.

DA reactions of (1E,3E)-1,4-dideutero-1,3-butadiene

General procedure for Diels-Alder reactions of (1E,3E)-1,4-dideutero-1,3-butadiene (1)

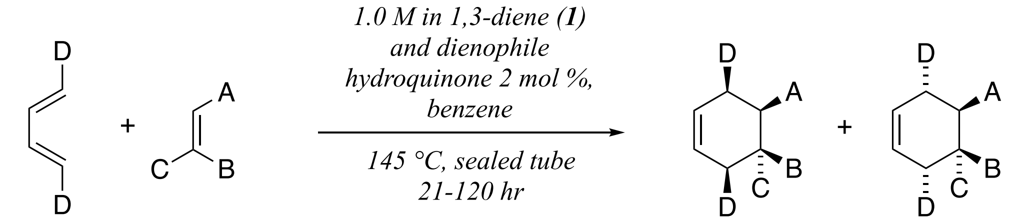

1,3-diene **1** | dienophile **(3-15)** | *Typical conditions* | *endo* adduct **($3\text{-}15_{BD}$-n)** | *exo* adduct **($3\text{-}15_{BD}$-x)**

Hydroquinone (2 mg, 0.02 mmol), the dienophile (1.0 mmol) and a solution of (1*E*,3*E*)-1,4-dideutero-1,3-butadiene (**1**) in benzene (1.0 mL, 1.0 M, 1.0 mmol) were sealed into a glass ampoule and heated to 145 °C for the stated time. The ampoule was cooled and opened and the reaction mixture was concentrated under vacuum. The residue was purified by flash column chromatography to give a mixture of DA adducts **$3\text{-}15_{BD}$-n** and **$3\text{-}15_{BD}$-x**. The *endo:exo* ratio was obtained from integration values from the quantitative ^{1}H NMR spectrum of the adduct mixture and the sample of (1*E*,3*E*)-1,4-dideutero-1,3-butadiene (**1**) used in the DA reaction, as described in the following section. The DA reaction of (1*E*,3*E*)-1,4-dideutero-1,3-butadiene (**1**) and methyl vinyl ketone (**4**) is used as an example.

Calculation of *endo:exo* selectivity for the DA reactions of (1E,3E)-1,4-dideutero-1,3-butadiene (1)

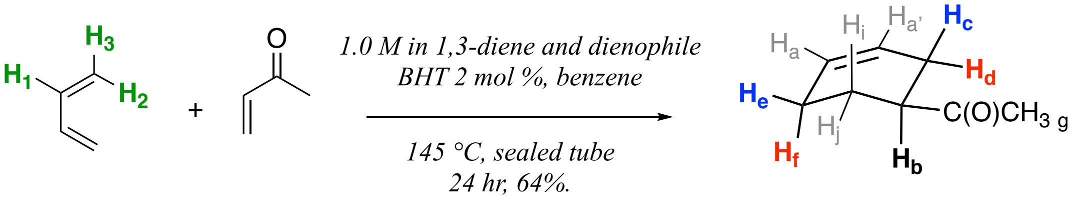

(1) **(4)** **(4_{BD}-n/x)**

Resonance	Integral	Resonance	Integral
H_1	1.00	H_b	1.00
H_2	0.96	H_c	0.41
H_3	0.12	H_d	0.67
		H_e	0.41
		H_f	0.66

In calculating the *endo:exo* ratio for the DA reaction of (1*E*,3*E*)-1,4-dideutero-1,3-butadiene (**1**) with methyl vinyl ketone (**4**), it is convenient to normalise the ^{1}H NMR integrals of the ^{2}H enriched sites in the adduct mixture by comparison with the ^{1}H NMR integrals of sites containing ^{2}H at natural abundance; approximately 0.015%. This gives the ^{1}H content of ^{2}H enriched sites as a

simple fraction of the natural content. It is also convenient to express the *endo*:*exo* ratio in terms which sum to unity:

$$(n : x) = (endo : exo)$$
$$n + x = 1$$

Equations expressing the observed values of the integrals at the deuterium enriched sites of the DA adducts **4$_{BD}$-n** and **4$_{BD}$-x** in terms of the *endo:exo* ratio and the integrals of the deuterium enriched sites of the (1*E*,3*E*)-1,4-dideutero-1,3-butadiene (**1**) were constructed:

$$H_{c,e} = xH_2 + nH_3$$
and
$$H_{d,f} = xH_3 + nH_2$$

Solving the first of these for *n* gives:

$$n = (H_{d,f} - H_3)/(H_2 - H_3)$$

Substituting integral data gives the calculated *endo:exo* selectivity of the reaction:

$$n = (H_{d,f} - H_3)/(H_2 - H_3)$$
$$n = (0.67 - 0.12) /(0.96 - 0.12)$$
$$n = 0.65$$
$$x = (1 - n) = 0.35$$

The figures obtained from the two calculations correspond to within 1%. The calculated *endo:exo* ratio of the DA reaction of (1*E*,3*E*)-1,4-dideutero-1,3-butadiene (**1**) with methyl vinyl ketone (**4**) is 65:35. The *endo:exo* ratios of the DA reactions of (1*E*,3*E*)-1,4-dideutero-1,3-butadiene with other dienophiles, (**3**), (**5**), (**6**), (**7**), (**8**), (**9**), (**10**), (**11**), (**14**) and (**15**), were obtained in a similar manner.

Cyclohex-3-enecarbaldehyde (3$_{BD}$)

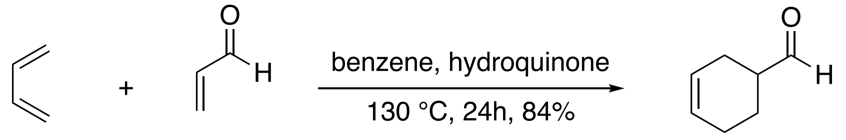

(**1**) (**3**) (**3$_{BD}$**)

This compound was prepared according to the modified procedure of Diels and Alder.[4] A solution of acrolein (**3**) (280 mg, 5.0 mmol) and hydroquinone (3 mg, 0.027 mmol) in benzene (10 mL) was stirred under an atmosphere of 1,3-butadiene (**1**) in a screw cap tube fitted with a rubber septum for 10 mins at rt. Excess 1,3-butadiene (**1**) dissolved into the solution. The tube was then sealed and placed in a preheated oil bath with stirring at 130 °C for 24 h. The tube was then removed from the

oil bath, cooled and opened, and its contents were concentrated under vacuum. The crude material was purified by flash column chromatography eluting with 40-60 petrol:ethyl acetate (40:1) to give the title compound (**3BD**) as a colourless oil (462 mg, 4.20 mmol, 84%): R_f 0.2 40-60 petrol:ethyl acetate (40:1); ^{1}H NMR (800 MHz, C_6D_6/DMSO-d_6 49:1) δ/ppm 9.29 (s, 1H), 5.51-5.46 (m, 2H), 1.99-1.95 (m, 1H), 1.95-1.91 (m, 1H), 1.87-1.82 (m, 1H), 1.82-1.76 (m, 1H), 1.72-1.66 (m, 1H), 1.56-1.51 (m, 1H) and 1.34-1.28 (m, 1H); ^{13}C NMR (200 MHz, C_6D_6/DMSO-d_6 49:1) δ/ppm 202.7, 127.1, 125.1, 45.9, 24.4, 23.8 and 22.1; IR (thin film) ν = 3026, 2921, 2840, 2710, 1726, 1652, 1438 cm^{-1}; MS (70 eV, EI): *m/z* (%): 110 (70) [M]$^+$, 92 (26), 81 (79), 79 (100), 57 (36); HRMS calc for $C_7H_{10}O$ [M]$^+$: 110.0732; found: 110.0732.

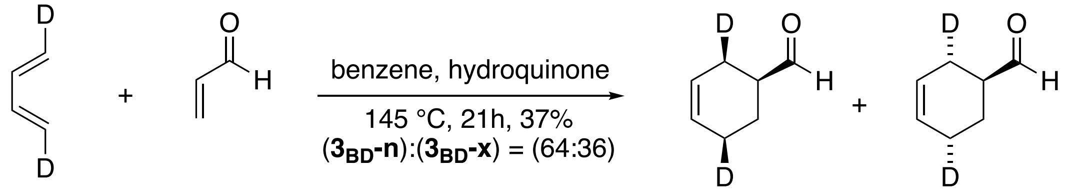

(**1**) (**3**) (**3BD-n**) (**3BD-x**)

Hydroquinone (2 mg, 0.02 mmol), acrolein (**3**) (56 mg, 1.0 mmol) and a solution of (1*E*,3*E*)-1,4-dideutero-1,3-butadiene (**1**) in benzene (1.0 mL, 1.0 M, 1.0 mmol) were sealed into a glass ampoule and heated to 145 °C for 21 h. The ampoule was cooled and opened and the reaction mixture was concentrated under vacuum. The residue was purified by flash column chromatography eluting with 40-60 petrol:ethyl acetate (40:1) to give a mixture of the Diels-Alder adducts (**3BD-n**) and (**3BD-x**) (*endo:exo* 64:36) as a colourless oil (50 mg, 0.37 mmol, 37%): R_f 0.2 40-60 petrol:ethyl acetate (40:1); IR (thin film) ν = 3026, 2924, 2867, 2161 (C-D), 1726, 1647, 1452 cm^{-1}; MS (70 eV, EI): *m/z* (%): 112 (71) [M]$^+$, 83 (100), 80 (84), 56 (56); HRMS calc for $C_7H_8D_2O$ [M]$^+$: 112.0857; found: 112.0854.

1-(Cyclohex-3-enyl)ethanone (4BD)

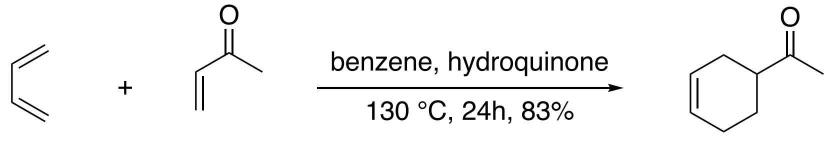

(**1**) (**4**) (**4BD**)

This compound was prepared according to the modified procedure of Petrov.[5] A solution of methyl vinyl ketone (**4**) (350 mg, 5.0 mmol) and hydroquinone (3 mg, 0.027 mmol) in benzene (10 mL) was stirred under an atmosphere of 1,3-butadiene (**1**) in a screw cap tube fitted with a rubber septum for 10 mins at rt. Excess 1,3-butadiene (**1**) dissolved into the solution. The tube was then

sealed and placed in a preheated oil bath with stirring at 130 °C for 24 h. The tube was then removed from the oil bath, cooled and opened, and its contents were concentrated under vacuum. The crude material was purified by flash column chromatography eluting with 40-60 petrol:ethyl acetate (40:1) to give the title compound (**4$_{BD}$**) as a colourless oil (513 mg, 4.13 mmol, 83%): R_f 0.2 40-60 petrol:ethyl acetate (40:1); ^{1}H NMR (800 MHz, C_6D_6/DMSO-d_6 24:1) δ/ppm 5.57-5.52 (m, 2H), 2.18-2.11 (m, 1H), 2.10-2.03 (m, 1H), 1.92-1.87 (m, 1H), 1.87-1.82 (m, 1H), 1.82-1.75 (m, 1H), 1.72 (3H, s), 1.69-1.64 (m, 1H) and 1.42-1.35 (m, 1H); ^{13}C NMR (200 MHz, C_6D_6/DMSO-d_6 24:1) δ/ppm 209.0, 126.7, 125.7, 47.0, 27.4, 27.0, 24.9 and 24.7; IR (thin film) ν = 3026, 2921, 2840, 1710, 1653, 1436 cm^{-1}; MS (70 eV, EI): *m/z* (%): 124 (76) [M]$^+$, 109 (22), 81 (98), 79 (43), 43 (100); HRMS calc for $C_8H_{12}O$ [M]$^+$: 124.0888; found: 124.0888.

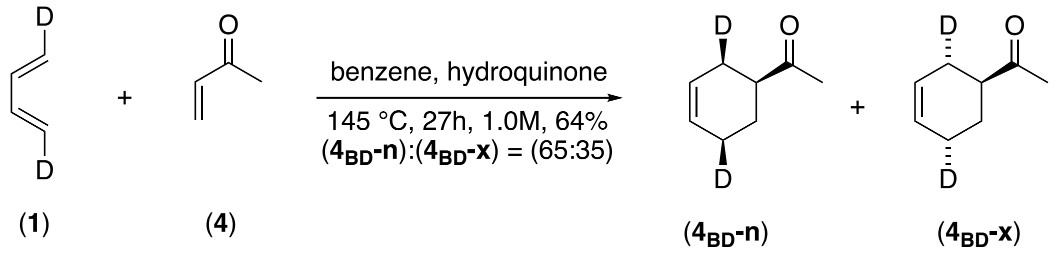

Hydroquinone (2 mg, 0.02 mmol), methyl vinyl ketone (**4**) (70 mg, 1.0 mmol) and a solution of (1*E*,3*E*)-1,4-dideutero-1,3-butadiene (**1**) in benzene (1.0 mL, 1.0 M, 1.0 mmol) were sealed into a glass ampoule and heated to 145 °C for 27 h. The ampoule was cooled and opened, and the reaction mixture was concentrated under vacuum. The residue was purified by flash column chromatography eluting with 40-60 petrol:ethyl acetate (40:1) to give a mixture of the Diels-Alder adducts (**4$_{BD}$-n**) and (**4$_{BD}$-x**) (*endo:exo* 65:35) as a colourless oil (81 mg, 0.64 mmol, 64%): R_f 0.2 40-60 petrol:ethyl acetate (40:1); IR (thin film) ν = 3025, 2924, 2866, 2161 (C-D), 2134 (C-D), 1709, 1647, 1425 cm^{-1}; MS (70 eV, EI): *m/z* (%): 126 (76) [M]$^+$, 111 (16), 81 (84), 82 (40), 43 (100); HRMS calc for $C_8H_{10}D_2O$ [M]$^+$: 126.1014; found: 126.1012.

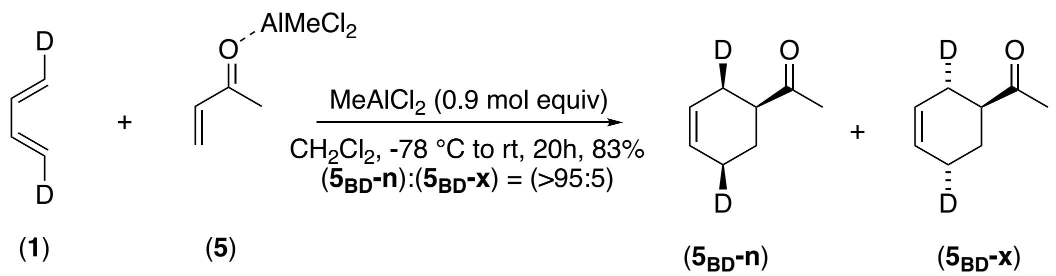

Methyl vinyl ketone (**4**) was treated with 0.9 molar equivalents of the $MeAlCl_2$ in hexanes at –78 °C, and a solution of (1*E*,3*E*)-1,4-dideutero-1,3-butadiene (**1**) in CH_2Cl_2 was added to the dienophile-Lewis acid complex at this temperature. This gave a 0.25 M solution of the 1,3-diene (**1**), with 1.0 molar equivalent of methyl vinyl ketone (**4**), and 0.9 molar equiv. $MeAlCl_2$ in 3:1

CH_2Cl_2:hexanes. The reaction mixture was then allowed to warm to room temperature and was stirred at this temperature for 20 hours. After an aqueous workup and purification by column chromatography, the DA adducts (**5$_{BD}$-n**) and (**5$_{BD}$-x**) were obtained in 83% isolated yield, with an *endo:exo* ratio > 95:5 (**Figure S2**).

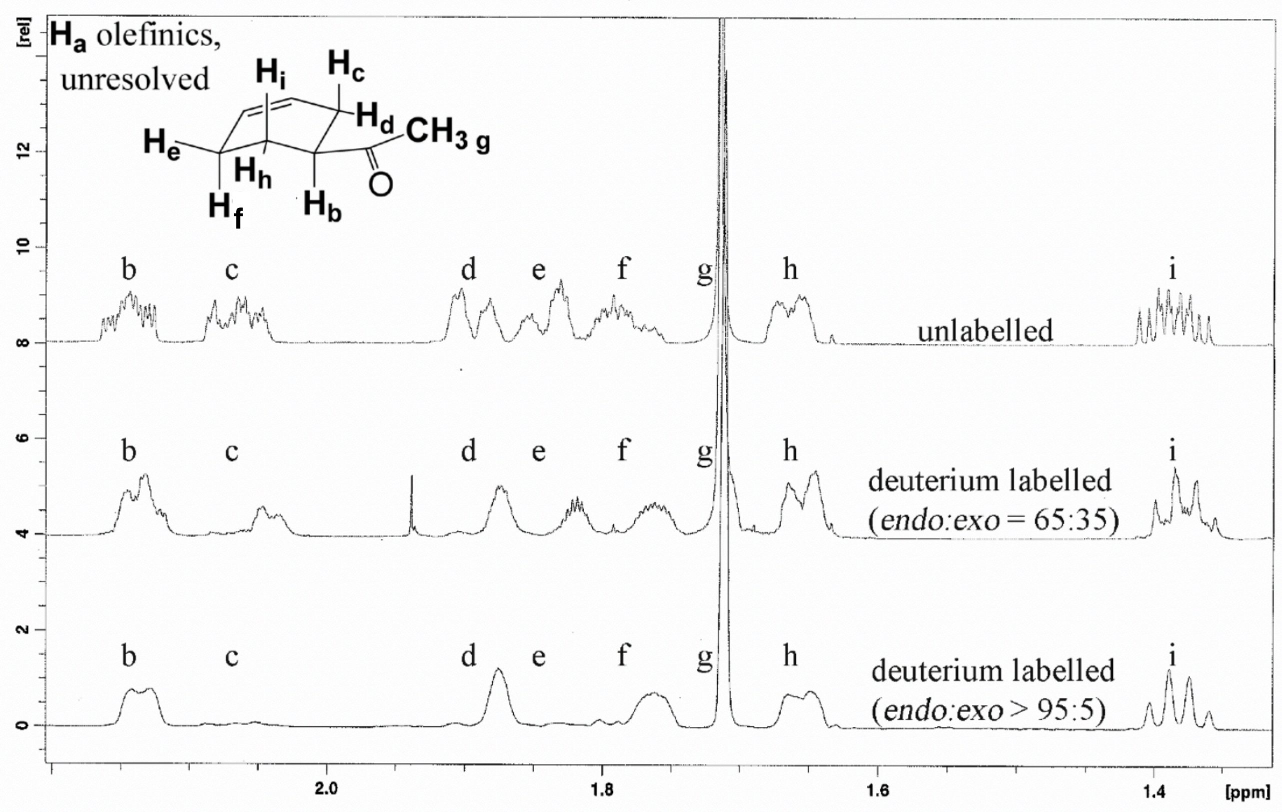

Figure S2. *Comparison of aliphatic regions of the ^1H NMR spectra of unlabelled and deuterium-labelled samples of (4$_{BD}$/5$_{BD}$). Top: Unlabelled sample. Middle: Deuterium labelled sample; DA reaction between methyl vinyl ketone (4) and (E,E)-1,4-dideutero-1,3-butadiene (1) carried out in benzene at 145 °C, (endo:exo = 65:35). Bottom: Deuterium labelled sample; $MeAlCl_2$ promoted DA reaction between methyl vinyl ketone (4) and (E,E)-1,4-dideutero-1,3-butadiene (1) carried out in CH_2Cl_2 and hexanes (3:1), from –78 °C to rt, endo:exo > 95:5.*

Cyclohex-3-enecarboxylic acid (6$_{BD}$)

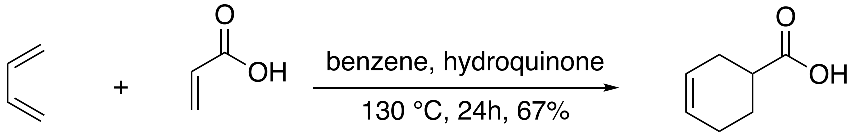

(1) (6) (6$_{BD}$)

This compound was prepared according to the modified procedure of Petrov and Sopov.[6] A solution of freshly distilled acrylic acid (**6**) (1.48 g, 20.5 mmol) and hydroquinone (4 mg, 0.036 mmol) in benzene (10 mL) was stirred under an atmosphere of 1,3-butadiene (**1**) in a screw cap tube fitted with a rubber septum for 10 mins at rt. Excess 1,3-butadiene (**1**) dissolved into the solution. The tube was then sealed and placed in a preheated oil bath with stirring at 130 °C for 24 hr. The tube was then removed from the oil bath, cooled and opened, and its contents were concentrated

under vacuum. The crude material was purified by flash column chromatography eluting with 40-60 petrol:ethyl acetate (4:1) to give the title compound (**6_{BD}**) as a colourless oil (1.75 g, 13.9 mmol, 67%): R_f 0.2 40-60 petrol:ethyl acetate (4:1); ^{1}H NMR (800 MHz, C_6D_6/DMSO-d_6 49:1) δ/ppm 5.61-5.53 (m, 2H), 2.59-2.54 (m, 1H), 2.48-2.41 (m, 1H), 2.31-2.26 (m, 1H), 2.07-2.02 (m, 1H), 1.97-1.90 (m, 1H), and 1.89-1.75 (m, 2H); ^{13}C NMR (200 MHz, C_6D_6/DMSO-d_6 49:1) δ/ppm 177.7, 126.8, 125.9, 35.6, 28.0, 25.6, and 24.8; IR (thin film) ν = 3027, 2922, 2660, 1706, 1638, 1420 cm^{-1}; MS (70 eV, EI): *m/z* (%): 126 (4) $[M]^+$, 108 (44), 81 (79), 80 (100), 55 (87); HRMS calc for $C_7H_{10}O_2$ $[M]^+$: 126.0681; found: 126.0681.

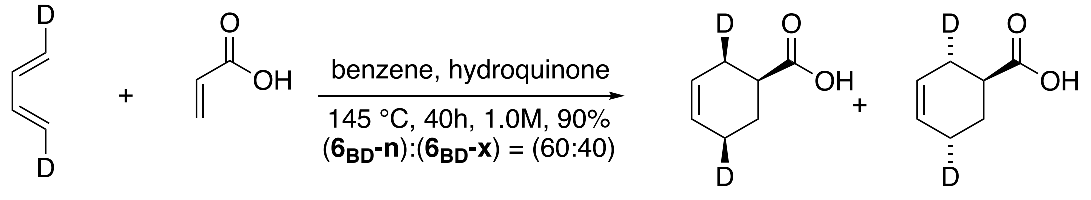

(**1**) (**6**) (**6_{BD}-n**) (**6_{BD}-x**)

Hydroquinone (2 mg, 0.02 mmol), freshly distilled acrylic acid (**6**) (65 mg, 0.9 mmol) and a solution of (1*E*,3*E*)-1,4-dideutero-1,3-butadiene in benzene (1.0 mL, 1.0 M, 1.0 mmol) were sealed into a glass ampoule and heated to 145 °C for 40 h. The ampoule was cooled and opened and the reaction mixture was concentrated under vacuum. The residue was purified by flash column chromatography eluting with 40-60 petrol:ethyl acetate (4:1) to give a mixture of the Diels-Alder adducts (**6_{BD}-n**) and (**6_{BD}-x**) (*endo:exo* 60:40) as a colourless oil (104 mg, 0.81 mmol, 90%): R_f 0.2 40-60 petrol:ethyl acetate (4:1); IR (thin film) ν = 3027, 2927, 2628, 2165 (C-D), 2139 (C-D), 1705, 1651, 1421 cm^{-1}; MS (70 eV, EI): *m/z* (%): 128 (48) $[M]^+$, 110 (32), 83 (97), 82 (100), 55 (47); HRMS calc for $C_7H_{10}D_2O_2$ $[M]^+$: 128.0806; found: 128.0807.

Methyl cyclohex-3-enecarboxylate (7_{BD})

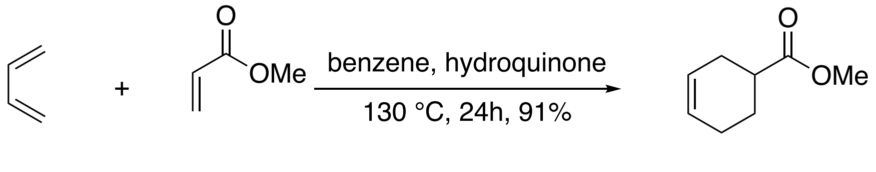

(**1**) (**7**) (**7_{BD}**)

This compound was prepared according to the modified procedure of Doucet and Rumpf.[7] A solution of methyl acrylate (**7**) (430 mg, 5.0 mmol) and hydroquinone (3 mg, 0.027 mmol) in benzene (10 mL) was stirred under an atmosphere of 1,3-butadiene (**1**) in a screw cap tube fitted with a rubber septum for 10 mins at rt. Excess 1,3-butadiene (**1**) dissolved into the solution. The tube was then sealed and placed in a preheated oil bath with stirring at 130 °C for 24 h. The tube

was then removed from the oil bath, cooled and opened, and its contents were concentrated under vacuum. The crude material was purified by flash column chromatography eluting with 40-60 petrol:ethyl acetate (40:1) to give the title compound (**7BD**) as a colourless oil (638 mg, 4.55 mmol, 91%): R_f 0.2 40-60 petrol:ethyl acetate (40:1); ^1H NMR (800 MHz, DMSO-d_6) δ/ppm 5.68-5.63 (m, 2H), 3.61 (s, 3H), 2.54 (1H, dddd, J = 10.1, 9.6, 3.1, 2.4 Hz), 2.22-2.17 (m, 1H), 2.15-2.10 (m, 1H), 2.06-2.00 (m, 2H), 1.92-1.88 (m, 1H) and 1.58-1.52 (m, 1H); ^{13}C NMR (200 MHz, DMSO-d_6) δ/ppm 175.3, 126.6, 125.1, 51.5, 38.3, 27.0, 24.7 and 23.8; IR (thin film) ν = 3027, 2951, 2930, 2843, 1737, 1653, 1436 cm^{-1}; MS (70 eV, EI): *m/z* (%): 140 (24) $[M]^+$, 108 (36), 81 (100), 80 (99), 53 (29); HRMS calc for $C_8H_{12}O_2$ $[M]^+$: 140.0835; found: 140.0837.

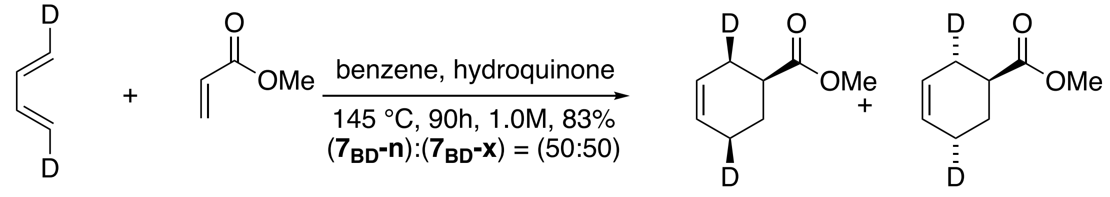

(**1**) (**7**) (**7BD-n**) (**7BD-x**)

Hydroquinone (2 mg, 0.02 mmol), methyl acrylate (**7**) (86.1 mg, 1.0 mmol) and a solution of (1*E*,3*E*)-1,4-dideutero-1,3-butadiene (**1**) in benzene (1.0 mL, 1.0 M, 1.0 mmol) were sealed into a glass ampoule and heated to 145 °C for 90 h. The ampoule was cooled and opened and the reaction mixture was concentrated under vacuum. The residue was purified by flash column chromatography eluting with 40-60 petrol:ethyl acetate (40:1) to give a mixture of the Diels-Alder adducts (**7BD-n**) and (**7BD-x**) (*endo:exo* 50:50) as a colourless oil (118 mg, 0.83 mmol, 83%): R_f 0.2 40-60 petrol:ethyl acetate (40:1); IR (thin film) ν = 3027, 2951, 2930, 2869, 2165 (C-D), 2137 (C-D), 1737, 1649, 1435 cm^{-1}; MS (70 eV, EI): *m/z* (%): 142 (24) $[M]^+$, 110 (16), 83 (100), 82 (61), 55 (51); HRMS calc for $C_8H_{10}D_2O_2$ $[M]^+$: 142.0963; found: 142.0964.

Cyclohex-3-ene-carboxamide (8BD)

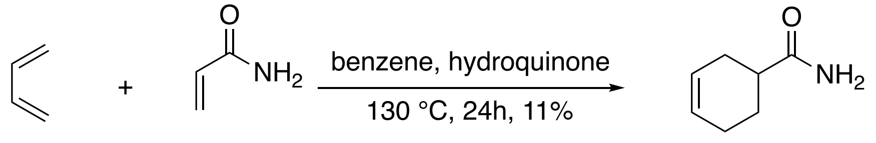

(**1**) (**8**) (**8BD**)

This compound was prepared according to the modified procedure of Hall.[8] Acrylamide (**8**) (125 mg, 1.00 mmol) and hydroquinone (2 mg, 0.018 mmol) were placed in a 2 mL glass ampoule and the neck of the ampoule was fitted with a rubber septum. A solution of 1,3-butadiene (**1**) (60 mg, 1.10 mmol) in benzene (1 mL) was added by syringe and the ampoule was sealed and placed in a

preheated oil bath at 130 °C for 24 h. The ampoule was then removed from the oil bath, cooled and opened, and its contents were concentrated under vacuum. The residue was taken up in CH_2Cl_2 (20 mL) and washed three times with water (20 mL), washed once with brine (20 mL), dried over $MgSO_4$, and concentrated under vacuum. The crude material was purified by flash column chromatography eluting with diethyl ether:ethyl acetate (20:1) to give the title compound (**8$_{BD}$**) as a pale yellow solid (14 mg, 0.11 mmol, 11%): R_f 0.2 diethyl ether:ethyl acetate (20:1); mp 148-149 °C; ^{1}H NMR (800 MHz, acetone-*d$_6$*) δ/ppm 6.75 (s br, 1H), 6.13 (s br, 1H), 5.68-5.61 (m, 2H), 2.44-2.38 (m, 1H), 2.23-2.17 (m, 1H), 2.14-2.00 (m, 3H) 1.96-1.91 (m, 1H) and 1.63-1.56 (m, 1H); ^{13}C NMR (200 MHz, 800 MHz, acetone-*d$_6$*) δ/ppm 178.1, 127.2, 126.6, 41.1, 28.9, 26.7 and 25.5; IR (KBr disk) ν = 3352, 3177, 3029, 2923, 1661, 1625, 1423 cm^{-1}; MS (70 eV, EI): *m/z* (%): 125 (100) $[M]^+$, 105 (100), 108 (20), 96 (28), 81 (99) 67 (60); HRMS calc for $C_7H_{11}NO$ $[M]^+$: 125.0841; found: 125.0841.

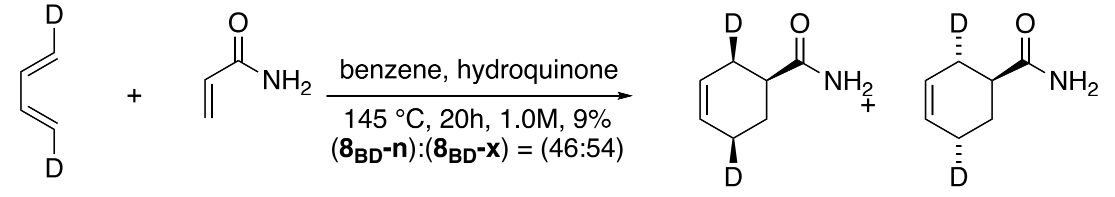

(**1**) (**8**) (**8$_{BD}$-n**) (**8$_{BD}$-x**)

Hydroquinone (2 mg, 0.02 mmol), acrylamide (**8**) (64 mg, 0.9 mmol) and a solution of (1*E*,3*E*)-1,4-dideutero-1,3-butadiene (**1**) in benzene (1.0 mL, 1.0 M, 1.0 mmol) were sealed into a glass ampoule and heated to 145 °C for 20 h. The ampoule was cooled and opened and the reaction mixture was concentrated under vacuum. The residue was purified by flash column chromatography eluting with diethyl ether:ethyl acetate (20:1) to give a mixture of the Diels-Alder adducts (**8$_{BD}$-n**) and (**8$_{BD}$-x**) (*endo:exo* 46:54) as a pale yellow solid (10 mg, 0.081 mmol, 9%): R_f 0.2 diethyl ether:ethyl acetate (20:1) IR (KBr disk) ν = 3352, 3179, 3031, 2923, 2162 (C-D), 2137 (C-D), 1658, 1633, 147 cm^{-1}; MS (70 eV, EI): *m/z* (%): 127 (80) $[M]^+$, 111 (16), 97 (25), 83 (100) 69 (92); HRMS calc for $C_7H_9D_2NO$ $[M]^+$: 127.0966; found: 127.0965.

Cyclohex-3-enecarbonitrile (9$_{BD}$)

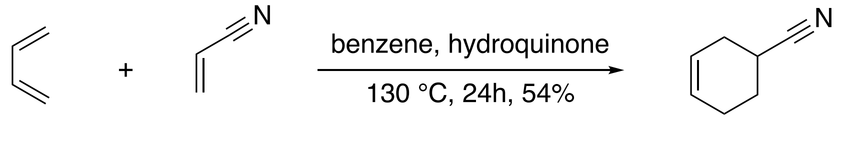

(**1**) (**9**) (**9$_{BD}$**)

This compound was prepared according to the modified procedure of Petrov and Sopov.[6] A solution of acrylonitrile (**9**) (265 mg, 5.0 mmol) and hydroquinone (3 mg, 0.027 mmol) in benzene

(10 mL) was stirred under an atmosphere of 1,3-butadiene (**1**) in a screw cap tube fitted with a rubber septum for 10 mins at room temperature. Excess 1,3-butadiene (**1**) dissolved into the solution. The tube was then sealed and placed in a preheated oil bath with stirring at 130 °C for 24 h. The tube was then removed from the oil bath, cooled and opened, and its contents were concentrated under vacuum. The crude material was purified by flash column chromatography eluting with 40-60 petrol:ethyl acetate (40:1) to give the title compound (**9$_{BD}$**) as a colourless oil (290 mg, 2.71 mmol, 54%): R_f 0.2 40-60 petrol:ethyl acetate (40:1); ^{1}H NMR (800 MHz, $CDCl_3$) *δ*/ppm 5.75 (1H, dddd, *J* = 10.2, 5.7, 2.1, 2.1 Hz), 5.65-5.62 (m, 1H), 2.83-2.80 (m, 1H), 2.41-2.37 (m, 1H), 2.34-2.29 (m, 1H), 2.27-2.21 (m, 1H), 2.13-2.07 (m, 1H), 1.95 (1H, ddddd, *J* = 13.3, 5.8, 5.8, 3.1, 0.8 Hz) and 1.91-1.86 (m, 1H); ^{13}C NMR (200 MHz, $CDCl_3$) *δ*/ppm 127.2, 123.4, 122.6, 28.3, 25.5, 24.4, and 23.1; IR (thin film) ν = 3032, 2931, 2845, 2239, 1653, 1438 cm^{-1}; MS (70 eV, EI): *m/z* (%): 107 (43) $[M]^+$, 92 (20), 80 (46), 67 (25), 54 (100); HRMS calc for C_7H_9N $[M]^+$: 107.0735; found: 107.0738.

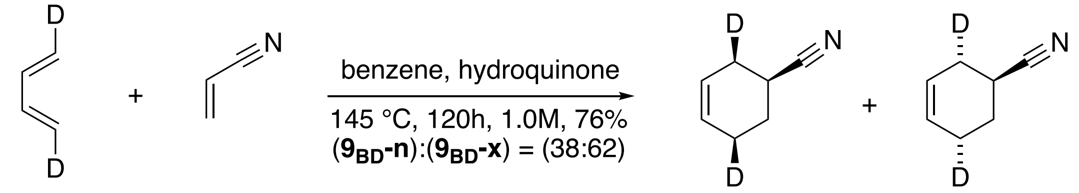

(**1**) (**9**) (**9$_{BD}$-n**) (**9$_{BD}$-x**)

Hydroquinone (2 mg, 0.02 mmol), acrylonitrile (**9**) (53 mg, 1.0 mmol) and a solution of (1*E*,3*E*)-1,4-dideutero-1,3-butadiene (**1**) in benzene (1.0 mL, 1.0 M, 1.0 mmol) were sealed into a glass ampoule and heated to 145 °C for 120 h. The ampoule was cooled and opened and the reaction mixture was concentrated under vacuum. The residue was purified by flash column chromatography eluting with 40-60 petrol:ethyl acetate (40:1) to give a mixture of the Diels-Alder adducts (**9$_{BD}$-n**) and (**9$_{BD}$-x**) (*endo:exo* 38:62) as a colourless oil (83 mg, 0.76 mmol, 76%): R_f 0.2 40-60 petrol:ethyl acetate (40:1); IR (thin film) ν = 3032, 2931, 2872, 2238, 1653, 2164 (C-D), 2133(C-D), 1650, 1452 cm^{-1}; MS (70 eV, EI): *m/z* (%): 109 (100) $[M]^+$, 93 (26), 82 (68), 68 (32), 56 (78); HRMS calc for $C_7H_7D_2N$ $[M]^+$: 109.0861; found: 109.0864.

3a,4,7,7a-Tetrahydroisobenzofuran-1(3H)-one (10$_{BD}$)

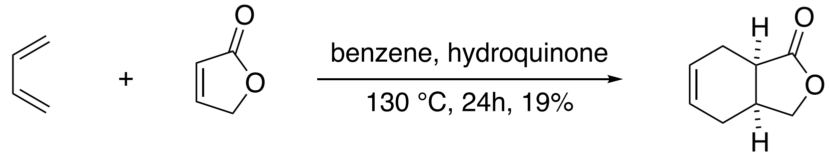

(**1**) (**10**) (**10$_{BD}$**)

This compound was prepared according to the modified procedure of Ortuno and Corbera.[9] A solution of furan-2(5*H*)-one (**10**) (474 mg, 5.64 mmol) and hydroquinone (3 mg, 0.027 mmol) in benzene (10 mL) was stirred under an atmosphere of 1,3-butadiene (**1**) in a screw cap tube fitted with a rubber septum for 10 mins at rt. Excess 1,3-butadiene (**1**) dissolved into the solution. The tube was then sealed and placed in a preheated oil bath with stirring at 130 °C for 24 h. The tube was then removed from the oil bath, cooled and opened, and its contents were concentrated under vacuum. The crude material was purified by flash column chromatography eluting with 40-60 petrol:ethyl acetate (20:1) to give the title compound (**10_{BD}**) as a colourless oil (150 mg, 1.09 mmol, 19%): R_f 0.2 40-60 petrol:ethyl acetate (20:1); ^{1}H NMR (800 MHz, $CDCl_3$) δ/ppm 5.77-5.71 (m, 2H), 4.31 (1H, dd, *J* = 8.5, 5.0 Hz), 4.02 (1H, dd, *J* = 9.2, 2.3 Hz), 2.80-2.75 (m, 1H), 2.65-2.60 (m, 1H), 2.53-2.48 (m, 1H), 2.42-2.36 (m, 1H), 2.30-2.24 (m, 1H) and 1.94-1.88 (m, 1H); ^{13}C NMR (200 MHz, $CDCl_3$) δ/ppm 179.2, 125.3, 125.0, 72.9, 37.4, 32.2, 24.9 and 22.2; IR (thin film) ν = 3031, 2971, 2905, 2843, 1773, 1659, 1480, 1436, 1372 cm^{-1}; MS (70 eV, EI): *m/z* (%): 138 (88) $[M]^+$, 97 (69), 93 (100), 79 (89), 77 (73); HRMS calc for $C_8H_{10}O_2$ $[M]^+$: 138.0681; found: 138.0681.

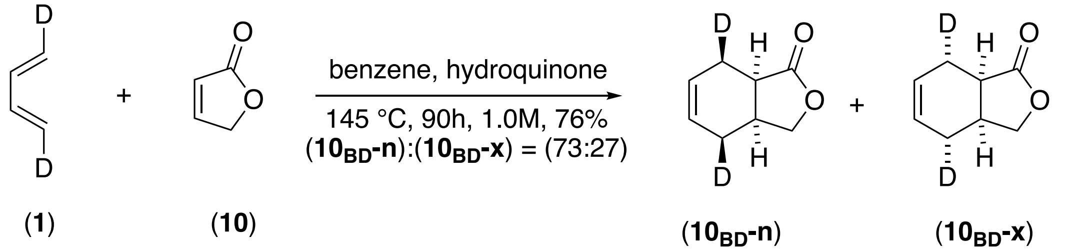

Hydroquinone (2 mg, 0.02 mmol), furan-2(5*H*)-one (**10**) (84 mg, 1.0 mmol) and a solution of (1*E*,3*E*)-1,4-dideutero-1,3-butadiene (**1**) in benzene (1.0 mL, 1.0 M, 1.0 mmol) were sealed into a glass ampoule and heated to 145 °C for 90 h. The ampoule was cooled and opened and the reaction mixture was concentrated under vacuum. The residue was purified by flash column chromatography eluting with 40-60 petrol:ethyl acetate (20:1) to give a mixture of the Diels-Alder adducts (**10_{BD}-n**) and (**10_{BD}-x**) (*endo:exo* 73:27) as a colourless oil (38 mg, 0.27 mmol, 27%): R_f 0.2 40-60 petrol:ethyl acetate (20:1); IR (thin film) ν = 3031, 2972, 2904, 2132, 2115, 1771, 1479, 1434, 1373 cm^{-1}; MS (70 eV, EI): *m/z* (%): 140 (78) $[M]^+$, 98 (63), 94 (85), 81 (100), 78 (82); HRMS calc for $C_8H_8D_2O_2$ $[M]^+$: 140.0807; found: 140.0806.

2-Oxa-spiro[4.5]dec-6-en-1-one (11_{BD})

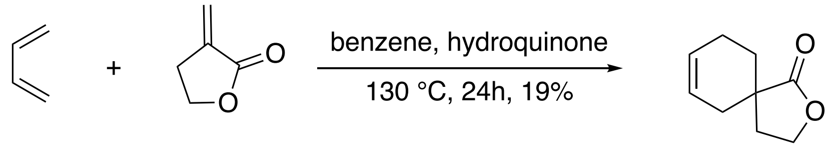

(1) (11) (11_{BD})

This compound was prepared according to the modified procedure of Kwan and coworkers.[10] Hydroquinone (2 mg, 0.018 mmol) was placed in a 2 mL glass ampoule and the neck of the ampoule was fitted with a rubber septum. Dihydro-3-methylenefuran-2(3*H*)-one (**11**) (90 mg, 1.06 mmol) and a solution of 1,3-butadiene (**1**) (55 mg, 1.02 mmol) in benzene (1 mL) were added by syringe and the ampoule was sealed and placed in a preheated oil bath at 130 °C for 24 h. The ampoule was then removed from the oil bath, cooled and opened, and its contents were concentrated under vacuum. The crude material was purified by flash column chromatography eluting with 40-60 petrol:ethyl acetate (20:1) to give the title compound (**11_{BD}**) as a white solid (123 mg, 0.81 mmol, 81%): R_f 0.2 40-60 petrol:ethyl acetate (20:1); mp 60-62 °C; ^{1}H NMR (800 MHz, toluene-*d_8*) *δ*/ppm 5.59-5.45 (m, 1H), 5.41-5.37 (m, 1H), 3.59-3.51 (m, 2H), 2.41 (1H, d, *J* = 17.8 Hz), 1.81 (1H, d, *J* = 17.9 Hz), 1.70 (1H, ddd, *J* = 11.7, 11.7, 4.6 Hz), 1.64-1.58 (m, 1H), 1.50 (1H, d, *J* = 17.4 Hz), 1.37-1.32 (m, 2H) and 1.19 (1H, dd, *J* = 12.9, 4.9 Hz); ^{13}C NMR (200 MHz, toluene-*d_8*) *δ*/ppm 180.2, 126.3, 124.2, 64.2, 40.8, 32.8, 32.2, 28.3 and 22.0; IR (thin film) ν = 2986, 2936, 2842, 1772, 1439 cm^{-1}; MS (70 eV, EI): *m/z* (%): 152 (42) [M]$^+$, 123 (23), 93 (35), 79 (100), 54 (34); HRMS calc for $C_9H_{12}O_2$ [M]$^+$: 152.0837; found: 152.0837.

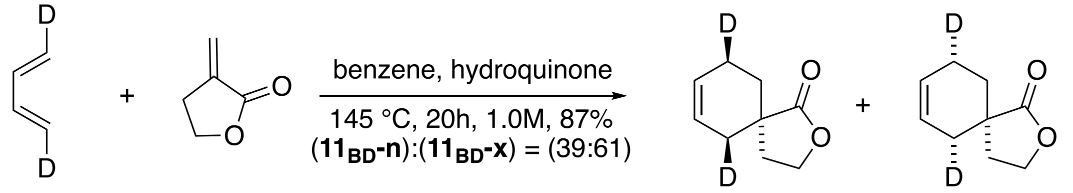

(1) (11) (11_{BD}-n) (11_{BD}-x)

Hydroquinone (2 mg, 0.02 mmol), dihydro-3-methylenefuran-2(3*H*)-one (**11**) (98 mg, 1.0 mmol) and a solution of (1*E*,3*E*)-1,4-dideutero-1,3-butadiene (**1**) in benzene (1.0 mL, 1.0 M, 1.0 mmol) were sealed into a glass ampoule and heated to 145 °C for 20 h. The ampoule was cooled and opened and the reaction mixture was concentrated under vacuum. The residue was purified by flash column chromatography eluting with 40-60 petrol:ethyl acetate (20:1) to give a mixture of the Diels-Alder adducts (**11_{BD}-n**) and (**11_{BD}-x**) (*endo:exo* 39:61) as a colourless oil (134 mg, 0.87 mmol, 87%): R_f 0.2 40-60 petrol:ethyl acetate (20:1); IR (thin film) ν = 3028, 2917, 2895, 2152 (C-

D), 2137 (C-D), 1770, 1645, 1486, 1445, 1372 cm^{-1}; MS (70 eV, EI): *m/z* (%): 154 (56) $[M]^+$, 126 (31) 95 (34), 81 (100), 56 (30); HRMS calc for $C_9H_{10}D_2O_2$ $[M]^+$: 154.0965; found: 154.0963.

Cyclohex-4-ene-1,2-dicarbonitrile (14$_{BD}$)

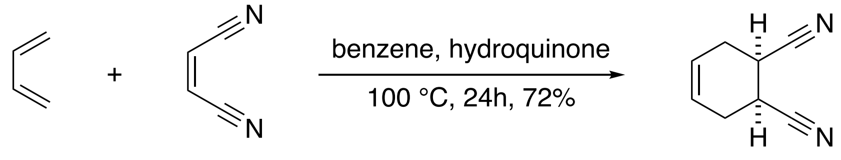

(**1**) (**14**) (**14$_{BD}$**)

This compound was prepared according to the modified procedure of Asastaseva and Vereshchagin.[11] Maleonitrile (**14**) (80.0 mg, 1.02 mmol) and hydroquinone (2 mg, 0.018 mmol) were placed in a 2 mL glass ampoule and the neck of the ampoule was fitted with a rubber septum. A solution of 1,3-butadiene (**1**) (60 mg, 1.10 mmol) in benzene (1 mL) was added by syringe and the ampoule was sealed and placed in a preheated oil bath at 100 °C for 24 h. The ampoule was then removed from the oil bath, cooled and opened, and its contents were concentrated under vacuum. The crude material was purified by flash column chromatography eluting with 40-60 petrol:ethyl acetate (7:1) to give the title compound (**14$_{BD}$**) as a colourless oil (97 mg, 0.72 mmol, 72%): R_f 0.1 40-60 petrol:ethyl acetate (7:1); ^1H NMR (800 MHz, C_6D_6/DMSO-d_6 49:1) δ/ppm 5.10-5.08 (m, 2H), 2.32-2.29 (m, 2H), 1.98-1.93 (m, 2H) and 1.65-1.60 (m, 2H); ^{13}C NMR (200 MHz, C_6D_6/DMSO-d_6 49:1) δ/ppm 123.5, 119.1, 27.3 and 26.3; IR (thin film) ν = 3068, 2932, 2853, 1657, 1438 cm^{-1}; MS (70 eV, EI): *m/z* (%): 132 (34) $[M]^+$, 105 (100), 79 (72), 66 (19), 54 (49); HRMS calc for $C_9H_{12}O_2$ $[M]^+$: 132.0687; found: 132.0690.

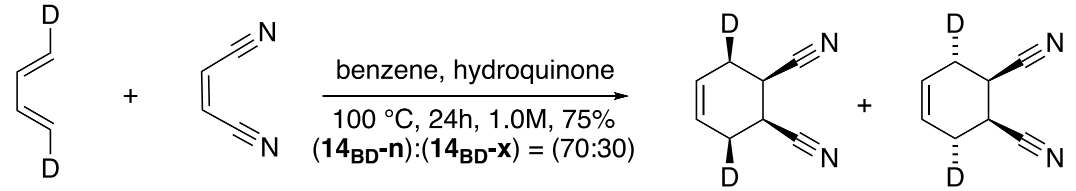

(**1**) (**14**) (**14$_{BD}$-n**) (**14$_{BD}$-x**)

Hydroquinone (2 mg, 0.02 mmol), maleonitrile (**14**) (78 mg, 1.0 mmol) and a solution of *(1E,3E)*-1,4-dideutero-1,3-butadiene (**1**) in benzene (1.0 mL, 1.0 M, 1.0 mmol) were sealed into a glass ampoule and heated to 100 °C for 24 h. The ampoule was cooled and opened and the reaction mixture was concentrated under vacuum. The residue was purified by flash column chromatography eluting with 40-60 petrol:ethyl acetate (7:1) to give a mixture of the Diels-Alder adducts (**14$_{BD}$-n**) and (**14$_{BD}$-x**) (*endo:exo* 70:30) as a colourless oil (101 mg, 0.75 mmol, 75%): R_f 0.1 40-60 petrol:ethyl acetate (7:1); IR (thin film) ν = 3067, 2941, 2245, 2183 (C-D), 2150 (C-D),

1650, 1512 cm^{-1}; MS (70 eV, EI): *m/z* (%): 134 (60) [M]$^+$, 107 (88), 80 (100), 67 (35), 56 (85); HRMS calc for $C_9H_{10}D_2O_2$ [M]$^+$: 134.0813; found: 134.0812.

(3aR,7aS)-3a,4,7,7a-tetrahydro-2-methyl-2H-isoindole-1,3-dione (15$_{BD}$)

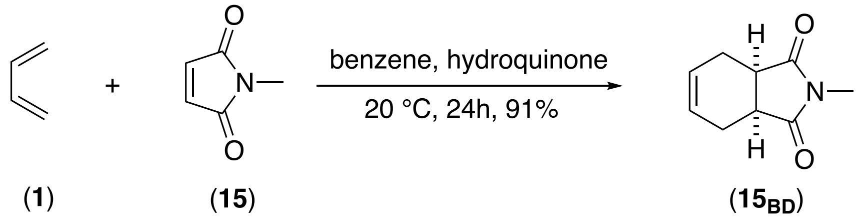

This compound was prepared according to the modified procedure of Rice.[12] *N*-methyl maleimide (**15**) (110.0 mg, 1.02 mmol) and hydroquinone (2 mg, 0.018 mmol) were placed in a 2 mL glass ampoule and the neck of the ampoule was fitted with a rubber septum. A solution of 1,3-butadiene (**1**) (60 mg, 1.10 mmol) in benzene (1 mL) was added by syringe and the ampoule was sealed and placed in a preheated oil bath at 20 °C for 24 h. The ampoule was then removed from the oil bath, cooled and opened, and its contents were concentrated under vacuum. The crude material was purified by flash column chromatography eluting with 40-60 petrol:ethyl acetate (4:1) to give the title compound (**15$_{BD}$**) as a white solid (152 mg, 0.92 mmol, 92%): R_f 0.3 40-60 petrol:ethyl acetate (4:1); mp 74-75 °C; ^{1}H NMR (800 MHz, $CDCl_3$) δ/ppm 5.82-5.80 (m, 2H), 3.04-3.02 (m, 2H), 2.87 (s, 3H), 2.54-2.50 (m, 2H) and 2.18-2.14 (m, 2H); ^{13}C NMR (200 MHz, $CDCl_3$) δ/ppm 177.5, 130.7, 42.3, 33.3 and 24.3; IR (thin film) ν = 3442, 3041, 2952, 1764, 1693, 1442 cm^{-1}; MS (70 eV, EI): *m/z* (%): 165 (100) [M]$^+$, 136 (30), 110 (23), 80 (96), 51 (36); HRMS calc for $C_9H_{11}NO_2$ [M]$^+$: 165.0790; found: 165.0788.

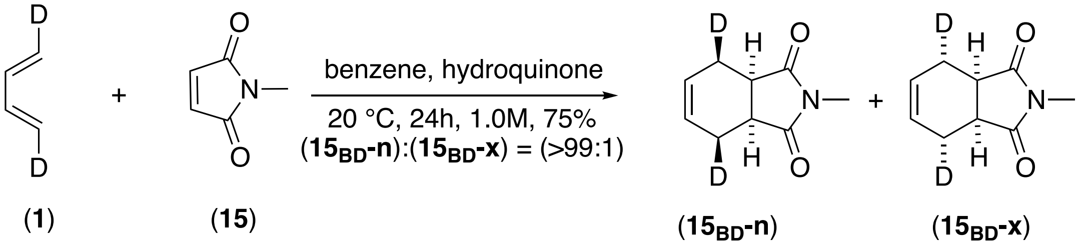

Hydroquinone (2 mg, 0.02 mmol), *N*-methyl maleimide (**15**) (110 mg, 1.0 mmol) and a solution of (1*E*,3*E*)-1,4-dideutero-1,3-butadiene (**1**) in benzene (1.0 mL, 1.0 M, 1.0 mmol) were sealed into a glass ampoule and held at 20 °C for 24 h. The ampoule was cooled and opened and the reaction mixture was concentrated under vacuum. The residue was purified by flash column chromatography eluting with 40-60 petrol:ethyl acetate (4:1) to give a mixture of the Diels-Alder adducts (**15$_{BD}$-n**) and (**15$_{BD}$-x**) (*endo:exo* >99:1) as a white solid (152 mg, 0.95 mmol, 95%): R_f 0.3 40-60 petrol:ethyl acetate (4:1); IR (thin film) ν = 3441, 3045, 2966, 2163 (C-D), 1750, 1699, 1442

cm^{-1}; MS (70 eV, EI): *m/z* (%): 167 (93) $[M]^+$, 138 (40), 111 (33), 82 (100), 56 (49); HRMS calc for $C_9H_9D_2NO_2$ $[M]^+$: 167.0915; found: 167.0914.

Gas Phase Reaction of (1E,3E)-1,4-dideutero-1,3-butadiene (1) with acrylonitrile (9)

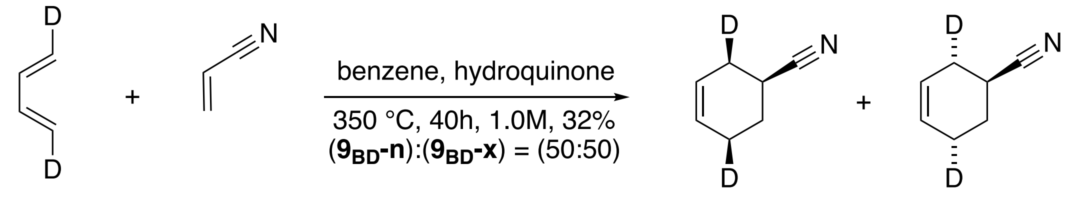

(1) (9) (9BD-n) (9BD-x)

Acrylonitrile (**9**) (53 mg, 1.0 mmol) and a solution of (1*E*,3*E*)-1,4-dideutero-1,3-butadiene (**1**) in benzene (1.0 mL, 1.0 M, 1.0 mmol) were sealed into a 1 L glass vessel under vacuum, and heated to 350 °C for 40 h. The vessel was cooled and opened, and the reaction mixture was concentrated under vacuum. The residue was purified by flash column chromatography eluting with 40-60 petrol:ethyl acetate (40:1) to give a mixture of the Diels-Alder adducts (**9$_{BD}$-n**) and (**9$_{BD}$-x**) (*endo:exo* 50:50) as a pale yellow oil (35 mg, 0.32 mmol, 32%): R_f 0.2 40-60 petrol:ethyl acetate (40:1); IR (thin film) ν = 3032, 2931, 2872, 2238, 1653, 2164 (C-D), 2133(C-D), 1650, 1452 cm^{-1}; MS (70 eV, EI): *m/z* (%): 109 (100) $[M]^+$, 93 (26), 82 (68), 68 (32), 56 (78); HRMS calc for $C_7H_7D_2N$ $[M]^+$: 109.0861; found: 109.0864.

2.2 Diels-Alder reactions between cyclopentadiene and common dienophiles.

Bicyclo[2.2.1]hept-5-ene-2-carbaldehyde (3$_{CPD}$-n) and (3$_{CPD}$-x)

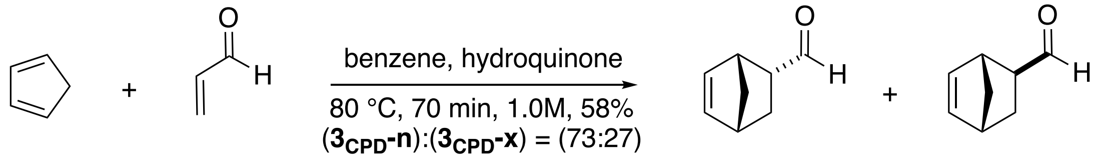

(2) (3) (3CPD-n) (3CPD-x)

Acrolein (**3**) (0.067 mL, 1.0 mmol) was added to an ampule followed by a solution of cyclopentadiene (**2**) in benzene (1.0 mL of a 1.0M solution in PhH, 1.0 mmol). The ampule was sealed and placed in a heating bath at 80 °C for 70 min. The reaction mixture was allowed to cool to ambient temperature then the ampule was cracked. At this stage the *endo:exo* ratio was recorded from equivalent runs conducted in benzene-d_6 as solvent and observed using quantitative ^{1}H NMR spectroscopy at 800 MHz. The reaction mixture was then directly subjected to flash chromatography on silica gel eluting with a 10:90 mixture of Et_2O and 30 – 40 °C petroleum spirits. The separated *endo*- and *exo*-products were obtained as colourless, volatile liquids (total yield 71 mg, 58%).

Endo adduct **3_{CPD}-n:** (51 mg, 42%): R_f = 0.26 (Et_2O : 30 – 40 °C petrol (10:90)); ν_{max}(film)/cm^{-1}; 3061, 2974, 2870, 2813, 2718, 1718 and 1569; ^{1}H NMR (800MHz; C_6D_6) δ/ppm 9.19 (1H, d, J = 2.3 Hz), 5.89 (1H, dd, J = 5.6 and 3.0 Hz), 5.72 (1H, dd, J = 5.7 and 2.8 Hz), 2.80 (1H, s), 2.55 (1H, s), 2.43 - 2.41 (1H, m), 1.46 (1H, ddd, J = 12.1, 9.1, 3.8 Hz), 1.25 (1H, ddd, J = 11.8, 4.0, 2.7 Hz), 1.21 (1H, ddt, J = 8.2, 2.0, 1.9 Hz), 0.86 (1H, d, J = 8.2 Hz); ^{13}C NMR (200MHz; C_6D_6) δ/ppm 202.7 (CH), 137.8 (CH), 132.1 (CH), 52.3 (CH), 49.6 (CH_2), 44.9 (CH), 43.0 (CH), 27.5 (CH_2); EIMS (70eV): *m/z* (%): 122.1 (35, $[M]^+$), 66.0 (100); HREIMS: calcd for $C_8H_{10}O$ $[M]^{+\cdot}$: 122.0732; found 122.0731.

Exo adduct **3_{CPD}-x:** (20 mg, 16%): R_f = 0.35 (Et_2O : 30 – 40 °C petrol (10:90)); ν_{max}(film)/cm^{-1} 3063, 2971, 2872, 2814, 2712, 1716 and 1570; ^{1}H NMR (800MHz; C_6D_6) δ/ppm 6.41 (1H, d, 2.0 Hz), 5.88 (1H, dd, 5.6 and 3.0 Hz), 5.79 (1H, dd, 5.6 and 3.0 Hz), 2.70 (1H, s) 2.56 (1H, s), 1.82 (1H, ddd, 6.5, 4.2, 2.2 Hz), 1.73 (1H, dt, 11.8, 3.9 Hz), 1.15 (1H, d, 8.8 Hz), 1.04 (1H, d, 8.8 Hz), 0.89 (1H, ddd, 11.8, 6.6, 2.2 Hz); ^{13}C NMR (200MHz; C_6D_6) δ/ppm 201.9 (CH), 138.4 (CH), 135.5 (CH), 51.8 (CH), 45.9 (CH_2), 44.3 (CH), 42.0 (CH), 27.0 (CH_2); EIMS (70eV): *m/z* (%): 122.1 (35, $[M]^+$), 66.0 (100); HREIMS: calcd for $C_8H_{10}O$ $[M]^{+\cdot}$: 122.0732; found 122.0732.

Bicyclo[2.2.1]hept-5-en-2-yl)ethanone (4_{CPD}-n) and (4_{CPD}-x)

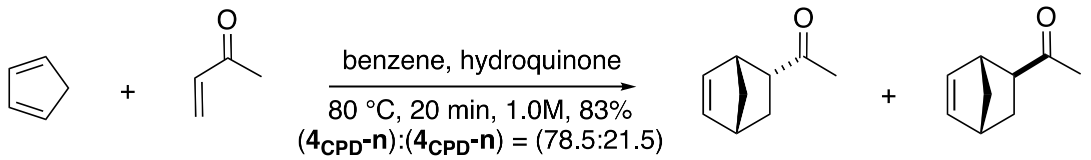

(2) **(4)** **(4_{CPD}-n)** **(4_{CPD}-x)**

Methyl vinyl ketone (**4**) (0.083 mL, 1.0 mmol), cyclopentadiene (0.082 mL, 1.0 mmol) and benzene (0.84 mL) were combined in an ampule. The ampule was sealed and placed in a heating bath at 80 °C for 20 min. The reaction mixture was allowed to cool to ambient temperature then the ampule was cracked. At this stage the *endo:exo* ratio was recorded from equivalent runs conducted in benzene-d_6 as solvent and observed using quantitative ^{1}H NMR spectroscopy at 800 MHz. The solution was then filtered through a short plug of silica eluting with petroleum spirits and then diethyl ether. The solvent was evaporated *in vacuo* and the residue subjected to flash chromatography on silica gel eluting with a 5:95 mixture of Et_2O and 30 – 40 °C petroleum spirits to give the products as colourless oils (total yield 107 mg, 83%).

Endo adduct **4_{CPD}-n:** (90 mg, 66%): R_f = 0.79 (Et_2O : 30 – 40 °C petrol (5:95)); ν_{max}(film)/cm^{-1} 3061, 2971, 2871 and 1708; ^{1}H NMR (800MHz; C_6D_6) δ/ppm 5.99 (1H, dd, 5.7 and 3.2 Hz), 5.76 (1H, dd, 5.6 and 2.8 Hz), 2.83 (1H, s), 2.60 (1H, s), 2.44 - 2.43 (1H, m), 1.71 (3H, s), 1.58 (1H, ddd, 11.5, 4.1 and 2.8), 1.44 (1H, ddd, 11.7, 9.1 and 3.7 Hz), 1.33 (1H, ddd, 8.0, 4.4 and 1.9 Hz),

0.97 (1H, d, 7.9); ^{13}C NMR (200MHz; C_6D_6) δ/ppm 206.0 (C), 137.7 (CH), 131.7 (CH), 52.2 (CH), 50.0 (CH_2), 45.9 (CH), 43.0 (CH), 28.6 (CH_3), 27.5 (CH_2); EIMS (70eV): *m/z* (%):136.1 (22, $[M]^+$), 93.0 (19), 83.9 (35), 71.0 (32), 66.0 (100); HREIMS: calcd for $C_9H_{12}O$ $[M]^+$: 136.0888, found 136.0889.

Exo adduct **4_{CPD}-x:** (23 mg, 17%): R_f = 0.16 (Et_2O : 30 – 40 °C petrol (5:95)); ν_{max}(film)/cm^{-1} 2973, 2943, 2870 and 1710; ^1H NMR (800MHz; C_6D_6) δ/ppm 5.95 (1H, dd, 5.6 and 3.0 Hz), 5.90 (1H, dd, 5.6 and 3.0 Hz), 2.78 (1H, s) 2.64 (1H, s), 1.96 (1H, dd, 8.4 and 4.2 Hz). 1.84 (1H, ddd, 11.4, 8.2 and 4.0 Hz), 1.76 (3H, s), 1.42 (1H, d, 8.4 Hz), 1.29 - 1.28 (1H, m), 0.99 (1H, ddd, 11.3, 9.0 and, 2.7 Hz); ^{13}C NMR (200MHz; C_6D_6) δ/ppm 208.0 (CH), 138.3, (CH), 136.0 (CH), 51.6 (CH), 46.2 (CH_2), 45.7 (CH), 42.1 (CH), 29.2 (CH_3), 29.2 (CH_2); EIMS (70eV): *m/z* (%): 136.1 (22 [M]), 93.1 (25), 66.0 (100); HREIMS: calcd for $C_9H_{12}O$ $[M]^+$: 136.0888 found 136.0888.

Bicyclo[2.2.1]hept-5-ene-2-carboxylic acid (6_{CPD}-n) and (6_{CPD}-x)

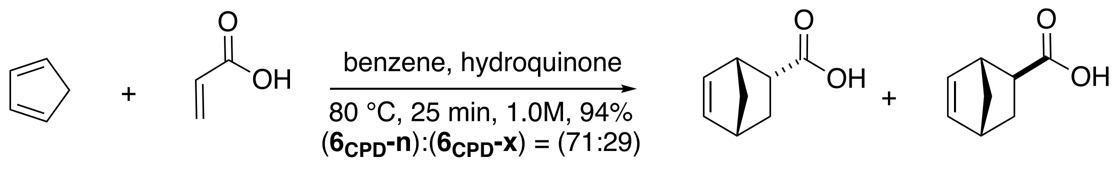

(**2**) (**6**) (**6$_{CPD}$-n**) (**6$_{CPD}$-x**)

Freshly distilled acrylic acid (**6**) (0.137 mL, 2.0 mmol) was added to and ampule followed by a solution of cyclopentadiene (**2**) in benzene (2.0 mL of a 1.0M solution in PhH, 2.0 mmol). The ampule was sealed and placed in a heating bath at 80 °C for 25 min. The reaction mixture was allowed to cool to ambient temperature then the ampule was cracked. At this stage the *endo:exo* ratio was recorded from equivalent runs conducted in benzene-d_6 as solvent and observed using quantitative ^1H NMR spectroscopy at 800 MHz. The solution was then filtered through a short plug of silica eluting with petroleum spirits and then diethyl ether. The solvent was evaporated *in vacuo* to give a clean mixture of **6_{CPD}-n** and **6_{CPD}-x** (71:29, total yield 260 mg, 94%). Analytical samples of both products were obtained by flash chromatography on silica gel eluting with a 40:60 mixture of Et_2O and 30 – 40 °C petroleum spirits.

Endo adduct **6_{CPD}-n**: R_f = 0.37 (Et_2O : 30 – 40 °C petrol (40:60)); ν_{max}(film)/cm^{-1} 3063, 2967, 2871, and 1703; ^1H NMR (800MHz; $CDCl_3$) δ/ppm 6.20 (1H, dd, 5.7 and 3.1 Hz), 5.60 (1H, dd, 5.6 and 2.9 Hz), 3.23 (1H, s), 2.99 (1H, dt, 9.3 and 3.9 Hz), 2.92 (1H, s), 1.92 (1H, ddd, 12.9, 9.3 and 3.8 Hz), 1.45 (1H, ddd, 8.3, 4.2 and 1.9 Hz), 1.40 (1H, ddd, 11.9, 4.2 and 2.9 Hz), 1.29 (1H, d, 8.3 Hz); ^{13}C NMR (200MHz; $CDCl_3$) δ/ppm 181.0 (C), 138.0 (CH), 132.5 (CH), 49.8 (CH_2), 45.8 (CH), 43.3 (CH), 42.6 (CH), 29.2 (CH_2); EIMS (70eV): *m/z* (%): 138.1 (44, $[M]^+$), 91.1 (82), 82.9 (100), 77.0 (75), 65.1 (88); HREIMS: calcd for $C_8H_{10}O_2$ $[M]^+$: 138.0681 found 138.0681.

Exo adduct **6_{CPD}-x**: R_f = 0.41 (Et_2O : 30 – 40 °C petrol (40:60)); ν_{max}(film)/cm^{-1} 3064, 2979, 2953, 2742, and 1698; ^{1}H NMR (800MHz; C_6D_6) δ/ppm 5.85 (1H, dd, 5.7 and 3.1 Hz), 5.80 (1H, dd, 5.5 and 2.9 Hz), 2.30 (1H, s), 2.59 (1H, s), 2.14 (1H, dd, 8.5 and 4.2 Hz), 1.91 (1H, dt, 11.7 and 4.0 Hz), 1.56 (1H, d, 8.8 Hz), 2.30 (1H, ddd, 6.8, 3.7 and 2.0 Hz), 1.15 (1H, ddd, 11.6, 9.0 and 2.6 Hz); ^{13}C NMR (200MHz; C_6D_6) δ/ppm 183.1 (C), 138..1 (CH), 135.9 (CH), 47.1 (CH), 46.6 (CH_2), 43.5 (CH), 42.0 (CH), 30.6 (CH_2); EIMS (70eV): *m/z* (%): 138.1 (4, [M]$^+$), 91.1 (8), 66.1 (100); HREIMS: calcd for $C_8H_{10}O_2$ [M]$^+$: 138.0681 found 138.0678.

Methyl bicyclo[2.2.1]hept-5-ene-2-carboxylate (7_{CPD}-n) and (7_{CPD}-x)

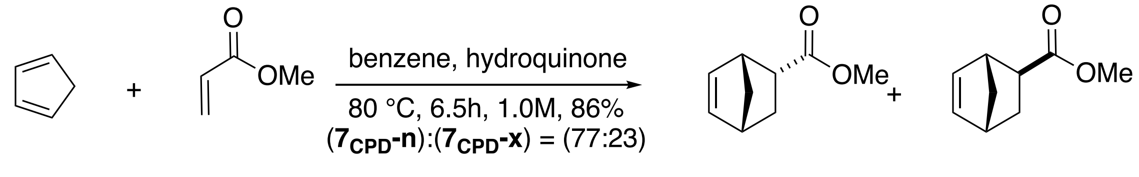

(2) **(7)** **(7_{CPD}-n)** **(7_{CPD}-x)**

Methyl acrylate (**7**) (0.18 mL, 1.0 mmol) was added to an ampule followed a solution of cyclopentadiene (**2**) in benzene (2.0 mL of a 1.0M solution in PhH, 2.0 mmol). The ampule was sealed and placed in a heating bath at 80 °C for 6.5 h. The reaction mixture was allowed to cool to ambient temperature then the ampule was cracked. At this stage the *endo:exo* ratio was recorded from equivalent runs conducted in benzene-d_6 as solvent and observed using quantitative ^{1}H NMR spectroscopy at 800 MHz. The solution was then filtered through a short plug of silica eluting with petroleum spirits and then diethyl ether. The solvent was removed *in vacuo* and the residue subjected to flash chromatography on silica gel eluting with a 2.5:97.5 mixture of Et_2O and 30 – 40 °C petroleum spirits. The products were obtained as colourless and pungent liquids (total yield 261 mg, 86%).

Endo adduct **7_{CPD}-n**: (186 mg, 61%): R_f = 0.18 (Et_2O : 30 – 40 °C petrol (2.5:97.5)); ν_{max}(film)/cm^1 3062, 2976, 2950, 2871 and 1738; ^{1}H NMR (800MHz; C_6D_6) δ/ppm 6.05 (1H, dd, 5.6 and 3.1 Hz), 5.98 (1H, dd, 5.6 and 2.8 Hz), 3.33 (1H, s), 3.10 (1H, s), 2.70 (1H, dt, 8.4 and 3.9 Hz), 2.60 (1H, s), 1.66 (1H, ddd, 11.7, 9.4 and 3.7 Hz), 1.55 (1H, dt, 11.6, 3.7 and 3.2 Hz), 1.29 (1H, d, 8.2 Hz), 0.93 (1H, d, 8.3 Hz); ^{13}C NMR (200MHz; C_6D_6) δ/ppm 174.3 (C), 137.8 (CH), 132.8 (CH), 51.0 (CH), 49.7 (CH_2), 46.0 (CH), 43.4 (CH), 42.9 (CH), 29.5 (CH_2); EIMS (70eV): *m/z* (%): 152.0 (62 [M]$^+$), 121.1 (80), 66.3 (100); HREIMS: calcd for $C_9H_{12}O_2$ [M]$^+$: 152.0837 found 152.0836.

Exo adduct **7_{CPD}-x**: (75 mg, 25%): R_f = 0.23 (Et_2O : 30 – 40 °C petrol (2.5:97.5)); ν_{max}(film)/cm^{-1} 3062, 2977, 2952 and 1733; ^{1}H NMR (800MHz; C_6D_6) δ/ppm 5.90 (1H, dd, 5.6 and 2.9 Hz), 5.86 (1H, dd, 5.6 and 3.0 Hz), 3.37 (1H s), 2.98 (1H, s), 2.65 (1H, s), 2.15 (1H, ddd, 8.5, 4.9 and 1.2 Hz), 1.98 (1H, dt, 11.7 and 3.9 Hz), 1.66 (1H, d, 8.4 Hz), 1.34, (1H, ddd, 6.4, 4.0 and 1.9) 1.19 (1H, ddd,

11.7, 9.0 and 2.6 Hz); ^{13}C NMR (200MHz; C_6D_6) δ/ppm 176.0 (C), 138.1 (CH), 135.9 (CH), 51.2 (CH), 47.0 (CH), 46.6 (CH_2), 43.2 (CH), 42.0 (CH), 30.6 (CH_2); EIMS (70eV): *m/z* (%): 152.0 (8 $[M]^+$), 66.3 (100); HREIMS: calcd for $C_9H_{12}O_2$ $[M]^+$: 152.0837 found 152.0840

Bicyclo[2.2.1]hept-5-ene-2-carboxamide (8_{CPD}-n) and (8_{CPD}-x)

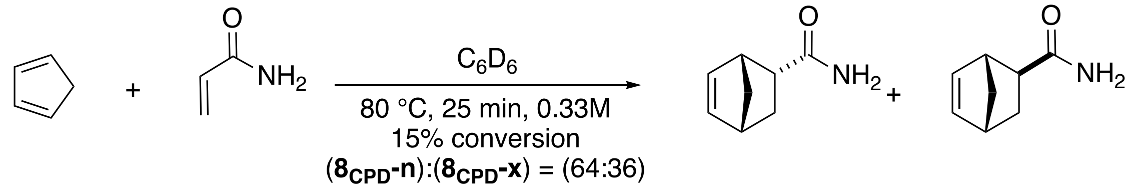

(**2**) (**8**) (**8_{CPD}-n**) (**8_{CPD}-x**)

This reaction was conducted in a J. Young Tap-fitted NMR tube using variable temperature NMR (500 MHz) at 80 °C. Acrylamide (**8**) (23.4 mg, 0.33 mmol), cyclopentadiene (0.082 mL, 1.0 mmol) and benzene-d_6 (0.9 mL) were combined in the NMR tube and inserted into the spectrometer (preheated to 80 °C). ^{1}H NMR spectra were recorded over a two-hour period and a consistent *endo:exo* ratio of 64:36 was observed. At 25 min the conversion was calculated to be 15%. All reaction components are in solution under these conditions. At extended reaction times considerable amounts of polymeric material is formed and the *endo:exo* ratio becomes unreliable. Samples of both products were obtained from a larger scale reaction: Acrylamide (71 mg, 1.0 mmol) was added to an ampule followed by a solution of cyclopentadiene in benzene (3.0 mL of a 1.0M solution in PhH, 3.0 mmol, 3 equiv). The ampule was sealed and placed in a heating bath at 80 °C for 16 h. The reaction mixture was allowed to cool to ambient temperature then the ampule was cracked. The solvent was evaporated *in vacuo* and the residue subjected to flash chromatography on silica gel eluting with a 5:95 mixture of ethyl acetate and diethyl ether to give the *exo* adduct **8_{CPD}-x** as a white crystalline solid (30 mg, 22%) and, after recrystallisation from acetone/ethyl acetate, the *endo* adduct **8_{CPD}-n** as a white crystalline solid (35 mg, 26%).

Endo adduct **8_{CPD}-n**: R_f = 0.09 (EtOAc : Et_2O (5:95)); Mp 158-162 °C (EtOAc/acetone); ν_{max}(film)/cm^{-1} 3360, 3188, 2990, 2959, 2941, 1657 and 1625; ^{1}H NMR (800MHz; $CDCl_3$) δ/ppm 6.26 (1H, dd, 5.5 and 3.1 Hz), 6.03 (1H, dd, 5.5 and 2.7 Hz), 5.35 (2H, br. s), 3.15 (1H, s) 2.93 (1H, s), 2.90 (1H dt, 9.1 and 4.0 Hz), 1.96 (1H, ddd, 12.0, 9.4 and 3.0 Hz), 1.48 (1H, dd, 6.5 and 2.0 Hz), 1.34 (1H, ddd, 11.8, 4.2 and 2.7), 1.31 (1H, d, 8.4 Hz); ^{13}C NMR (200MHz; $CDCl_3$) δ/ppm 176.8 (C), 138.0 (CH), 132.3 (CH), 50.2 (CH_2), 46.3 (CH), 44.6 (CH), 42.8 (CH), 30.1 (CH_2); EIMS (70eV): *m/z* (%): 137.1 (22 $[M]^+$), 97.1 (34), 81.1 (60), 69.1 (100); HREIMS: calcd for $C_8H_{11}NO$ $[M]^+$: 137.0841 found 137.0840.

Exo adduct **8_{CPD}-x** R_f = 0.19 (EtOAc : Et_2O (5:95)); Mp 148-150 °C (EtOAc/Et_2O); ν_{max}(film)/cm^{-1} 3374, 3195, 2978, 2939, 1657 and 1628; ^{1}H NMR (800MHz; $CDCl_3$) δ/ppm 6.15 (1H, dd, 5.5 and

3.0 Hz), 6.11 (1H, dd, 5.3 and 3.0 Hz), 5.58 (1H br. s), 5.48 (1H, br. s), 2.98 (1H, s), 2.92 (1H, s), 2.10 (1H, dd, 8.3 and 3.6 Hz), 1.93 (1H, dt, 11.4 and 4.0 Hz), 1.66 (1H, d, 8.3 Hz), 1.38 (1H, d, 7.7 Hz), 1.35 (1H, dd, 11.4 and 2.3 Hz); ^{13}C NMR (200MHz; $CDCl_3$) δ/ppm 178.2 (C), 138.4 (CH), 136.0 (CH), 47.1 (CH), 46.5 (CH_2), 44.1 (CH), 41.7 (CH), 30.7 (CH_2); EIMS (70eV): *m/z* (%): 137.1 (32 $[M]^+$), 81.1 (60), 69.1 (100); HREIMS: calcd for $C_8H_{11}NO$ $[M]^+$: 137.0841 found 137.0835.

Bicyclo[2.2.1]hept-5-ene-2-carbonitrile (9_{CPD}-n) and (9_{CPD}-x)

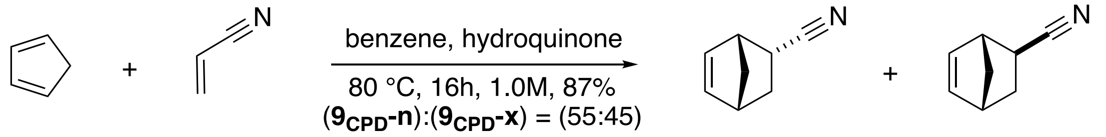

(2) (9) (9_{CPD}-n) (9_{CPD}-x)

Acrylonitrile (**9**) (0.066 mL, 1.0 mmol), cyclopentadiene (**2**) (0.247 mL, 3.0 mmol, 3.0 equiv) and benzene (0.7 mL) were combined in an ampule. The ampule was sealed and placed in a heating bath at 80 °C for 16 h. The reaction mixture was allowed to cool to ambient temperature then the ampule was cracked. At this stage the *endo:exo* ratio was recorded from equivalent runs conducted in benzene-*d*$_6$ as solvent and observed using quantitative ^{1}H NMR spectroscopy at 800 MHz. The solution was then subjected to flash chromatography on silica gel eluting with a 5:95 mixture of Et_2O and 30 – 40 °C petroleum spirits. The products were obtained as colourless and pungent liquids (**9_{CPD}-x** *exo*; 47 mg, 39% **9_{CPD}-n** *endo*; 57 mg, 48%; total yield 104 mg, 87%). ^{1}H NMR spectra were consistent with that reported.

Tetrahydro-4,7-methanoisobenzofuran-1(3*H*)-one (10_{CPD}-n) and (10_{CPD}-x)

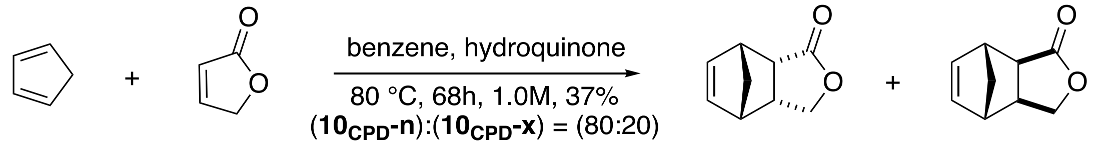

(2) (10) (10_{CPD}-n) (10_{CPD}-x)

Furan-2(5*H*)-one (**10**) (0.070 mL, 1.0 mmol), cyclopentadiene (**2**) (0.247 mL, 3.0 mmol, 1 equiv) and benzene (0.7 mL) were combined in an ampule. The ampule was sealed and placed in a heating bath at 80 °C for 68 h. The reaction mixture was allowed to cool to ambient temperature then the ampule was cracked. At this stage the *endo:exo* ratio was recorded from equivalent runs conducted in benzene-*d*$_6$ as solvent and observed using quantitative ^{1}H NMR spectroscopy at 800 MHz. The solution was then filtered through a short plug of silica eluting with petroleum spirits and then diethyl ether. The solvent was evaporated *in vacuo* and the residue was subjected to flash

chromatography on silica gel eluting with a 60:40 mixture of Et_2O and 30 – 40 °C petroleum spirits. The products were obtained as a white solid (*endo* adduct **10_{CPD}-n**) and a colourless oil (*exo* adduct **10_{CPD}-x**; total yield 60 mg, 37%).

Endo adduct **10_{CPD}-n:** (49.3 mg, 30%): R_f = 0.22 (Et_2O : 30 – 40 °C petrol (60:40)); Mp 91-92 °C (Et_2O/petrol) ν_{max}(KBr)/cm^{-1} 2991, 2978, 2954, and 1757; ^{1}H NMR (800MHz; C_6D_6) δ/ppm 6.13 (1H, dd, 5.6 and 2.8), 5.79 - 5.77 (1H, m), 3.57 (1H, ddd, 9.3, 4.0 and 1.9 Hz), 3.20 (1H, 9.6, 3.1 and 1.5 Hz), 2.98 (1H, s), 2.65 (1H, ddd, 9.4, 3.9 and 1.9 Hz), 2.34 (1H, s), 2.17 - 2.13 (1H, m), 1.21 (1H, d, 8.5 Hz), 0.81 (1H, d, 8.4 Hz); ^{13}C NMR (200MHz; C_6D_6) δ/ppm 176.7 (C), 137.0 (CH), 134.2 (CH), 69.3 (CH_2), 51.7 (CH_2), 47.3 (CH), 46.1 (CH), 46.0 (CH), 40.1 (CH); EIMS (70eV): *m/z* (%): 150.1 (2 $[M]^+$), 105.1 (9), 91.0 (55), 85.0 (47), 66.1 (100); HREIMS: calcd for $C_9H_{10}O_2$ $[M]^+$: 150.0681 found 150.0686.

Exo adduct **10_{CPD}-x:** (10.6 mg, 7%: R_f = 0.43 (Et_2O : 30 – 40 °C petrol (60:40)); ν_{max}(disc)/cm^{-1} 2992, 2953, 2883 and 1752; ^{1}H NMR (800MHz; C_6D_6) δ/ppm 5.74 (1H, dd, 5.7 and 3.8 Hz), 5.66 (1H, dd, 5.7 and 3.2 Hz), 3.63 (1H, dd, 9.21 and 9.21 Hz), 3.22 (1H, dd, 9.6 and 3.5 Hz), 3.05 (1H, s), 2.11 (1H, d, 8.2 Hz), 2.05 (1H, s), 1.60 - 1.57 (1H, m), 1.15 (1H, d, 9.6 Hz), 1.13 - 1.12 (1H, m); ^{13}C NMR (200MHz; C_6D_6) δ/ppm 176.2 (C), 137.6 (CH), 137.5 (CH), 70.8 (CH_2), 48.1 (CH), 47.7 (CH), 46.6 (CH), 43.3 (CH_2), 41.8 (CH); EIMS (70eV): *m/z* (%): 150.1 (1 $[M]^+$), 105.1 (2), 91.0 (10), 85.0 (9), 66.1 (100); HREIMS: calcd for $C_9H_{10}O_2$ $[M]^+$: 150.0681 found 150.0681.

Dihydro-2'*H*-spiro[bicyclo[2.2.1]hept[5]ene-2,3'-furan]-2'-one (11_{CPD}-n) and (11_{CPD}-x)

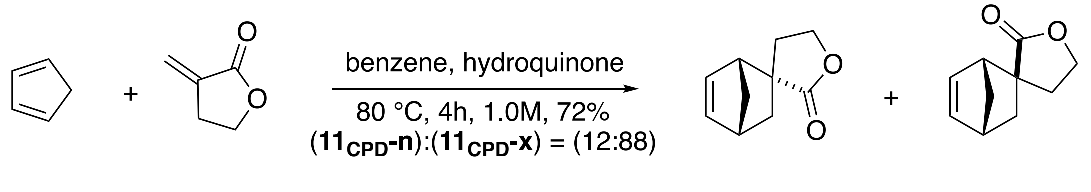

(**2**) (**11**) (**11_{CPD}-n**) (**11_{CPD}-x**)

To 3-methylenedihydrofuran-2(3*H*)-one (**11**) (98 mg, 1.0 mmol) was added to an ampule followed by a solution of cyclopentadiene in benzene (1.0 mL of a 1.0M solution in PhH, 1.0 mmol). The ampule was sealed and placed in a heating bath at 80 °C for 4 h. The reaction mixture was allowed to cool to ambient temperature then the ampule was cracked. At this stage the *endo:exo* ratio was recorded from equivalent runs conducted in benzene-d_6 as solvent and observed using quantitative ^{1}H NMR spectroscopy at 800 MHz. The solution was then directly subjected to flash chromatography on silica gel eluting with a 20:80 mixture of Et_2O and 30 – 40 °C petroleum spirits. The products were obtained as colourless liquids (total yield of 118 mg, 72%). The *exo* adduct **11_{CPD}-x** can be recrystallised from CH_2Cl_2/hexane.

Endo adduct **11_{CPD}-n:** (18 mg, 11%): R_f = 0.10 (Et_2O : 30 – 40 °C petrol (20:80)); ν_{max}(film)/cm^{-1} 2967, 2872, 1767 and 1666; ^1H NMR (800MHz; C_6D_6) δ/ppm 6.17 (1H, dd, 5.6 and 3.0 Hz), 6.03 (1H, dd, 5.6 and 3.0), 3.55 - 3.51 (2H, m), 2.54 (1H, s), 2.35 (1H, s), 1.61 (1H, dd, 11.6 and 2.6 Hz), 1.53 (1H, ddd, 8.8, 12.6 and 8.8 Hz), 1.31 - 1.28 (2H, m), 1.11 (1H, dd, 11.6 and 3.5 Hz), 0.92 (1H, d, 8.6 Hz); ^{13}C NMR (200MHz; C_6D_6) δ/ppm 179.1 (C), 137.7 (CH), 133.0 (CH), 63.9 (CH_2), 50.1 (CH), 49.4 (CH_2), 46.8 (C), 42.8 (CH), 39.7 (CH_2), 37.3 (CH_2); EIMS (70eV): *m/z* (%): 164.1 (5 $[M]^+$), 99.0 (73), 66.1 (100); HREIMS: calcd for $C_{10}H_{12}O_2$ $[M]^+$: 164.0837 found 164.0839.

Exo adduct **11_{CPD}-x:** (100 mg, 61%): R_f = 0.24 (Et_2O : 30 – 40 °C petrol (20:80)); Mp 48-49 °C (CH_2Cl_2/hexane); ν_{max}(KBr)/cm^{-1} 3061, 2975, 2909, and 1761; ^1H NMR (800MHz; C_6D_6) δ/ppm 5.95 (1H, dd, 5.6 and 3.0 Hz), 5.72 (1H, dd, 5.6 and 3.0 Hz), 3.53 - 3.46 (2H, m), 2.60 (1H, s), 2.55 (1H, s), 2.31 (1H, d, 8.2 Hz), 2.09 (1H, dd, 11.5 and 3.7 Hz), 1.34 - 1.29 (2H, m), 1.15 - 1.12 (1H, m), 0.62 (1H, dd, 11.5 and 2.6 Hz); ^{13}C NMR (200MHz; C_6D_6) δ/ppm 181.1 (C), 139.7 (CH), 134.4 (CH), 64.2 (CH_2), 49.3 (CH), 47.6 (C), 47.2 (CH_2), 43.1 (CH), 39.3 (CH_2), 35.1 (CH_2); EIMS (70eV): *m/z* (%): 164.1 (5 $[M]^+$), 99.0 (64), 66.1 (100); HREIMS: calcd for $C_{10}H_{12}O_2$ $[M]^+$: 164.0837 found 164.0833.

Bicyclo[2.2.1]hept-5-ene-2,3-dicarbonitrile (14_{CPD}-n) and (14_{CPD}-x)

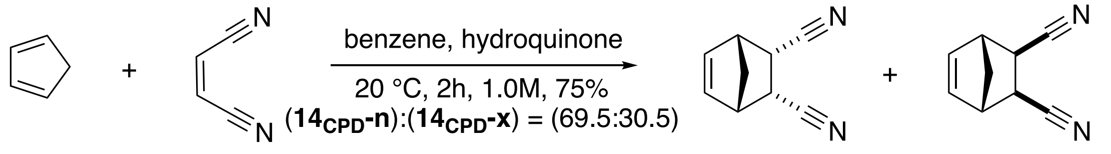

(**2**) (**14**) (**14_{CPD}-n**) (**14_{CPD}-x**)

To maleonitrile (**14**) (8.7 mg, 0.111 mmol) was added a solution of cyclopentadiene (**2**) in benzene (0.111 mL of a 1.0M solution in PhH, 0.111 mmol). The solution was allowed to stand at 20 °C for 2h and then the solvent was evaporated *in vacuo*. The residue was dissolved in $CDCl_3$ and the *endo:exo* ratio recorded using quantitative ^1H NMR spectroscopy at 800 MHz. The solvent was evaporated *in vacuo* and the residue was subjected to flash chromatography on silica gel eluting with a 70:30 mixture of Et_2O and hexane to give the products as white crystalline solids (total yield 12 mg, 75%). Both adducts were recrystallised from chloroform/hexane to give crystals suitable for single crystal X-ray analysis

Endo adduct **14_{CPD}-n:** (8 mg, 50 %): R_f = 0.15 (Et_2O : 30 – 40 °C petrol (70:30)); Mp 162 °C ($CHCl_3$/hexane); ν_{max}(KBr)/cm^{-1} 3014, 1996, 1958, 2243 and 1693; ^1H NMR (800MHz; $CDCl_3$) δ/ppm 6.51 (2H, s), 3.44 (2H, s), 3.28 (2H, s), 1.70 (1H, d, 9.2 Hz), 1.34 (1H, d, 9.2 Hz); ^{13}C NMR (200MHz; $CDCl_3$) δ/ppm 136.5 (CH), 118.2 (C), 48.0 (CH2), 46.8 (CH), 34.1 (CH); EIMS (70eV): *m/z* (%): 143.1 (10 $[M]^+$), 117.0 (24), 104.0 (50), 67.0 (53), 51.0 (58), 39.0 (100); HREIMS: calcd for $C_9H_7N_2$ $[M]^+$: 143.0609 found 143.0608.

Exo adduct **14$_{CPD}$-x:** (4 mg, 25%): R_f = 0.23 (Et_2O : 30 – 40 °C petrol (70:30)); Mp 120 °C ($CHCl_3$/hexane); ν_{max}(KBr)/cm^{-1} 3000, 2981, 2950, 2880, 2240 and 1660; ^{1}H NMR (800MHz; $CDCl_3$) *δ*/ppm 6.24 (2H, s), 3.43 (2H, s), 2.68 (2H, d, 1.8 Hz), 1.96 (1H, d, 10.3 Hz), 1.81 (1H, d, 10.3 Hz); ^{13}C NMR (200MHz; $CDCl_3$) *δ*/ppm 136.9 (CH), 118.7 (C), 48.2 (CH), 46.5 (CH_2), 33.6 (CH); EIMS (70eV): *m/z* (%): 143.1 (15 $[M]^+$), 117.1 (32), 104.0 (62), 66.0 (100); HREIMS: calcd for $C_9H_7N_2$ $[M]^+$: 143.0609 found 143.0604.

2-Methyl-3a,4,7,7a-tetrahydro-1*H*-4,7-methanoisoindole-1,3(2*H*)-dione (15$_{CPD}$)

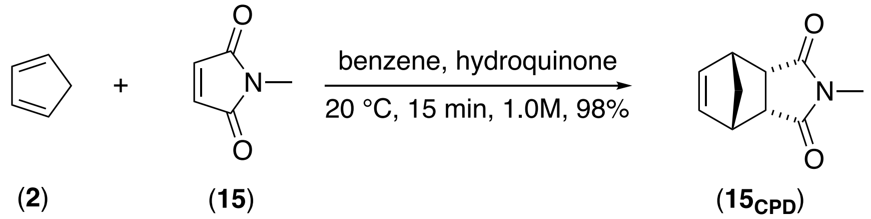

To *N*-methylmaleimide (**15**) (111 mg, 1.0 mmol) was added a solution of cyclopentadiene (**2**) in benzene (1.0 mL of a 1.0M solution in PhH, 1.0 mmol). The solution was allowed to stand at 20 °C for 15min and then solvent evaporated *in vacuo* to give the title compound **15$_{CPD}$** as a white crystalline solid (174 mg, 98%).

Endo adduct **15$_{CPD}$**: Mp 105-106 °C (benzene); ν_{max}(KBr)/cm^{-1} 3009, 2979, 2948, 1767 and 1691; ^{1}H NMR (800MHz; $CDCl_3$) *δ*/ppm 6.05 (2H, s), 3.34 (2H, s), 3.23 (2H, s), 2.77 (3H, s), 1.69 (1H, d, 8.4 Hz), 1.51 (1H, d, 8.4 Hz); ^{13}C NMR (200 MHz; $CDCl_3$) *δ*/ppm 177.8 (C), 134.4 (CH), 52.2 (CH_2), 46.0 (CH), 44.8 (CH), 24.2 (CH_3); EIMS (70eV): *m/z* (%): 177.1 (9 $[M]^+$), 112.0 (35), 91.1 (17), 84.0 (13), 66.1 (100); HREIMS: calcd for $C_{10}H_{11}NO_2$ $[M]^+$: 177.0790 found 177.0792.

Tetrahydro-1,4-methanonaphthalene-5,8-dione (16$_{CPD}$-n)

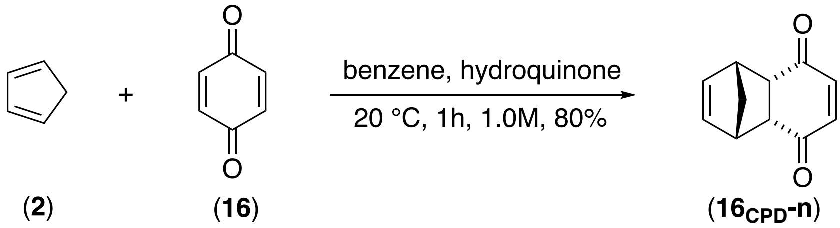

To benzoquinone (**16**) (108 mg, 1.0 mmol) was slowly added a solution of cyclopentadiene (**2**) in benzene (1.0mL of a 1.0M solution in PhH, 1.0 mmol). The solution was allowed to stand at 20 °C for 1h and then solvent evaporated *in vacuo*. The residue was subjected to flash chromatography on silica gel eluting with a 20:80 mixture of ethyl acetate and hexane to give the title compound **16$_{CPD}$-n** as a yellow crystalline solid (140 mg, 80%).

Endo adduct **16$_{CPD}$-n**: R_f = 0.14 (EtOAc : hexane (20:80)); Mp 66 – 67 °C (CH_2Cl_2/hexane) ν_{max}(KBr)/cm^{-1} 2992, 2970, 2943, 2874, 1762, 1670 and 1659; ^{1}H NMR (400MHz; $CDCl_3$) *δ*/ppm 6.55 (2H, s), 6.05 (2H, s), 3.54 (2H, dd, 3.6 and 2.0 Hz), 3.20 (2H, dd, 2.4 and 1.6 Hz), 1.53 (1H, d,

7.6 Hz), 1.42 (1H, d, 9.2 Hz); ^{13}C NMR (100MHz; $CDCl_3$) δ/ppm 199.4 (C), 142.0 (CH), 135.3 (CH), 48.7 (CH), 48.7 (CH_2), 48.3 (CH); EIMS (70eV): *m/z* (%): 174.1 (85 $[M]^+$), 146.1 (36), 117.1 (44), 91.0 (53), 66.0 (100); HREIMS: calcd for $C_{11}H_{10}O_2$ $[M]^+$: 174.0681 found 174.0682.

3 Anisotropic Displacement Ellipsoid Plots for $\mathbf{14_{CPD}}$-n, and $\mathbf{14_{CPD}}$-x

A crystal structure of (1*E*,3*E*)-1,4-diiodo-1,3-butadiene (**20**) was obtained showing identical characteristics to that already published (CCDC 824354, Figure S3).[13]

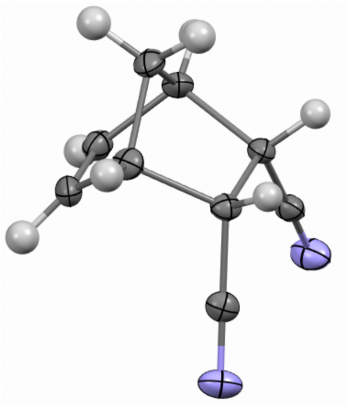

14_{CPD}-n (CCDC 2005761)

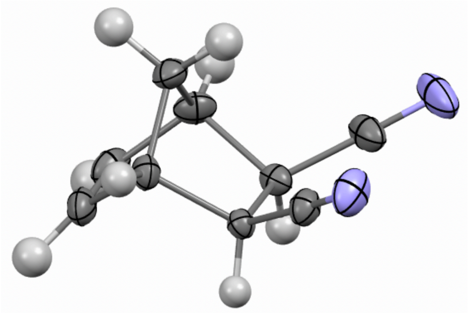

14_{CPD}-x (CCDC 2005762)

Figure S3. Anisotropic displacement ellipsoid plot of **14_{CPD}-n** (CCDC 2005761) and of **14_{CPD}-x** (CCDC 2005762). Ellipsoids show 30% probability levels.

4 References

[1] W. L. F. Armarego, C. Chai, *Purification of Laboratory Chemicals* 7th Edn. Butterworth-Heinemann, Oxford, **2012.**

[2] (a) Mitchenko, S. A.; Ananikov, V. P.; Beletskaya, I. P.; Ustynyuk, Y. A. *Mendeleev Commun.* **1997**, *7*, 130-131; (b) Ananikov, V. P.; Mitchenko, S. A.; Beletskaya, I. P.; *Russ. J. Org. Chem.* **2002**, *38*, 636-650; (c) Ananikov, V. P.; Kashin, A. S.; Hazipov, O. V.; Beletskaya, I. P.; Starikova, Z. A., *Synlett* **2011**, *22*, 2021-2024.

[3] Kitagawa, K.; Inoue, A.; Shinokubo, H.; Oshima, K. *Angew. Chem. Int. Ed.* **2000**, *39*, 2481-2483.

[4] Diels, O.; Alder, K. *Justus Liebigs Ann. Chem.* **1928**, *460*, 98-122.

[5] Petrov, A. A. *Russ. J. Gen. Chem.* **1941**, *11*, 309-313.

[6] Petrov, A. A.; Sopov, N. P. *Russ. J. Gen. Chem.* **1947**, *17*, 2228-2234.

[7] Doucet, J.; Rumpf, P. *B. Soc. Chim. Fr.* **1954**, *1954*, 610-13.

[8] Hall, H. K. *J. Am. Chem. Soc.* **1960**, *82*, 1209-1215.

[9] Ortuno, R. M.; Corbera, J.; Font, J. *Tetrahedron Lett.* **1986**, *27*, 1081-1084.

[10] Paddon-Row, M. N.; Kwan, L. C. H.; Willis, A. C.; Sherburn, M. S. *Angew. Chem. Int. Ed.* **2008**, *47*, 7013-7017.

[11] Asastaseva, A. P.; Vereshchagin, A. N. *Izv. An. SSSR Chim.* **1970**, *7*, 1497-1504.

[12] Rice, L. M.; Reid, E. E.; Grogan, C. H. *J. Org. Chem.* **1954**, *19*, 884-893.

[13] V. P. Ananikov, A. S. Kashin, O. V. Hazipov, I. P. Beletskaya, Z. A. Starikova, *Synlett* **2011**, *22*, 2021–2024.

5 COSY and nOe spectra for compounds 4_{CPD}-n and 4_{CPD}-x

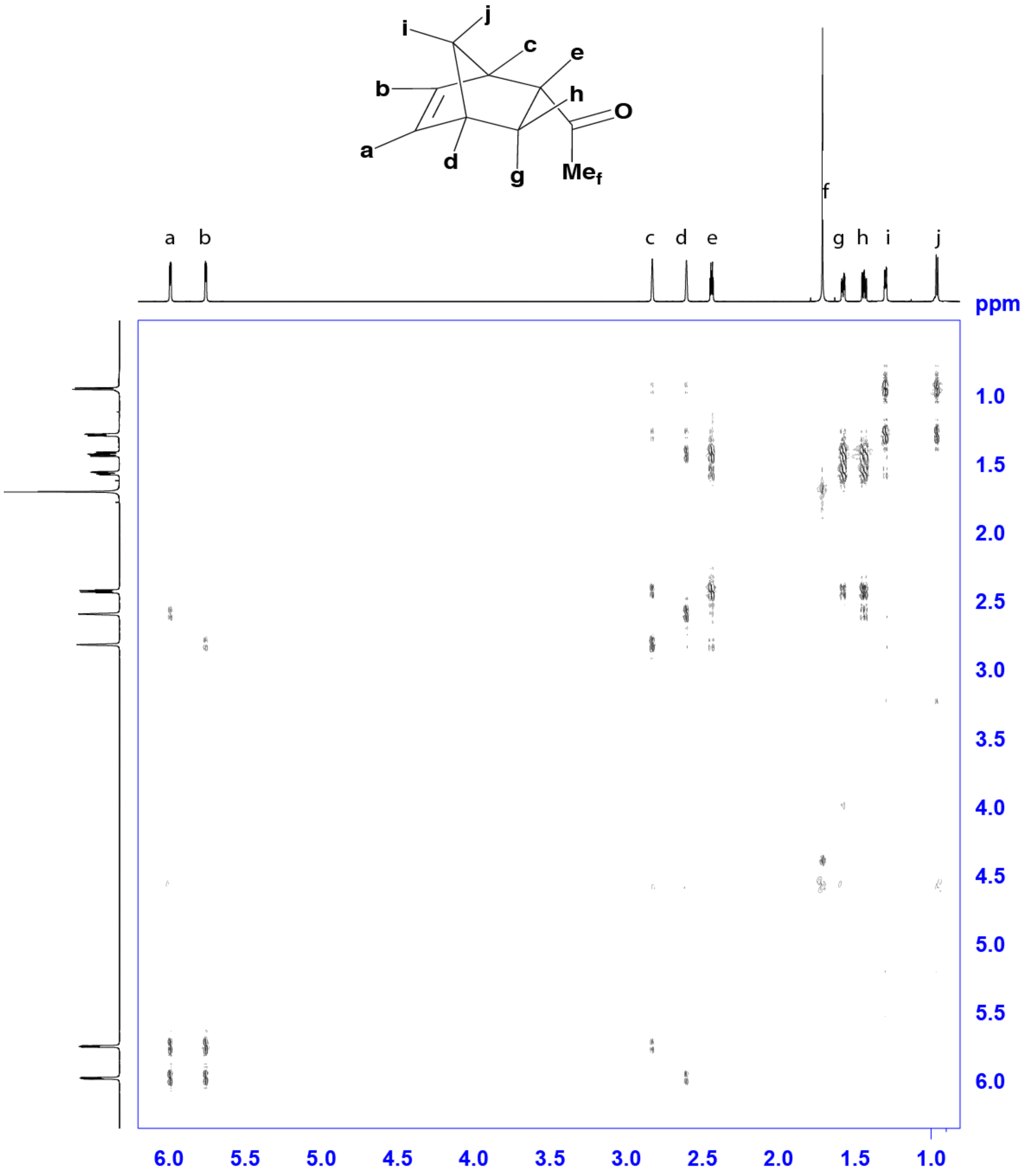

Figure S4. COSY spectrum of *endo* adduct **4$_{CPD}$-n**.

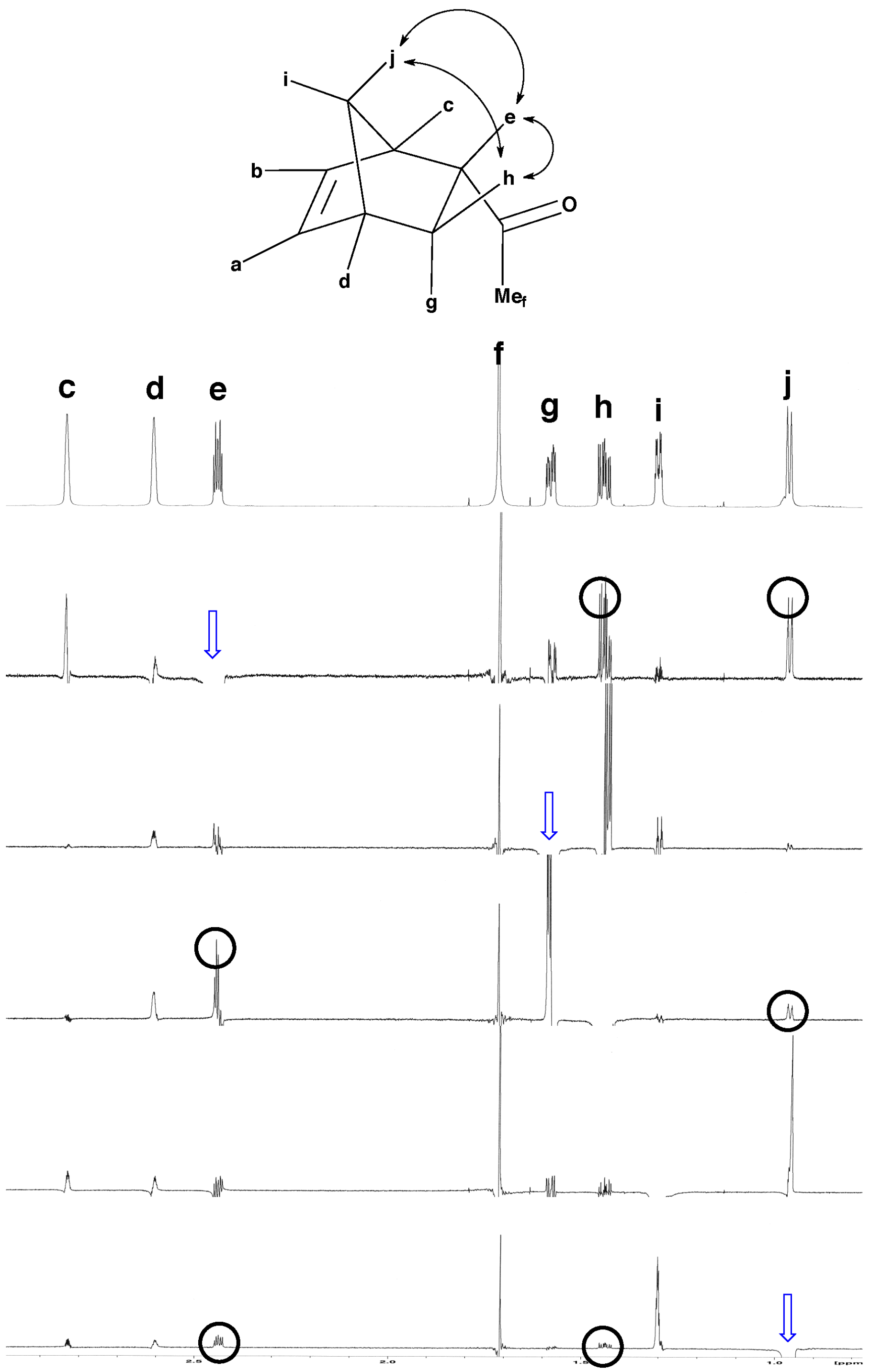

Figure S5. Array of nOe spectra of *exo* adduct **4$_{CPD}$-x**

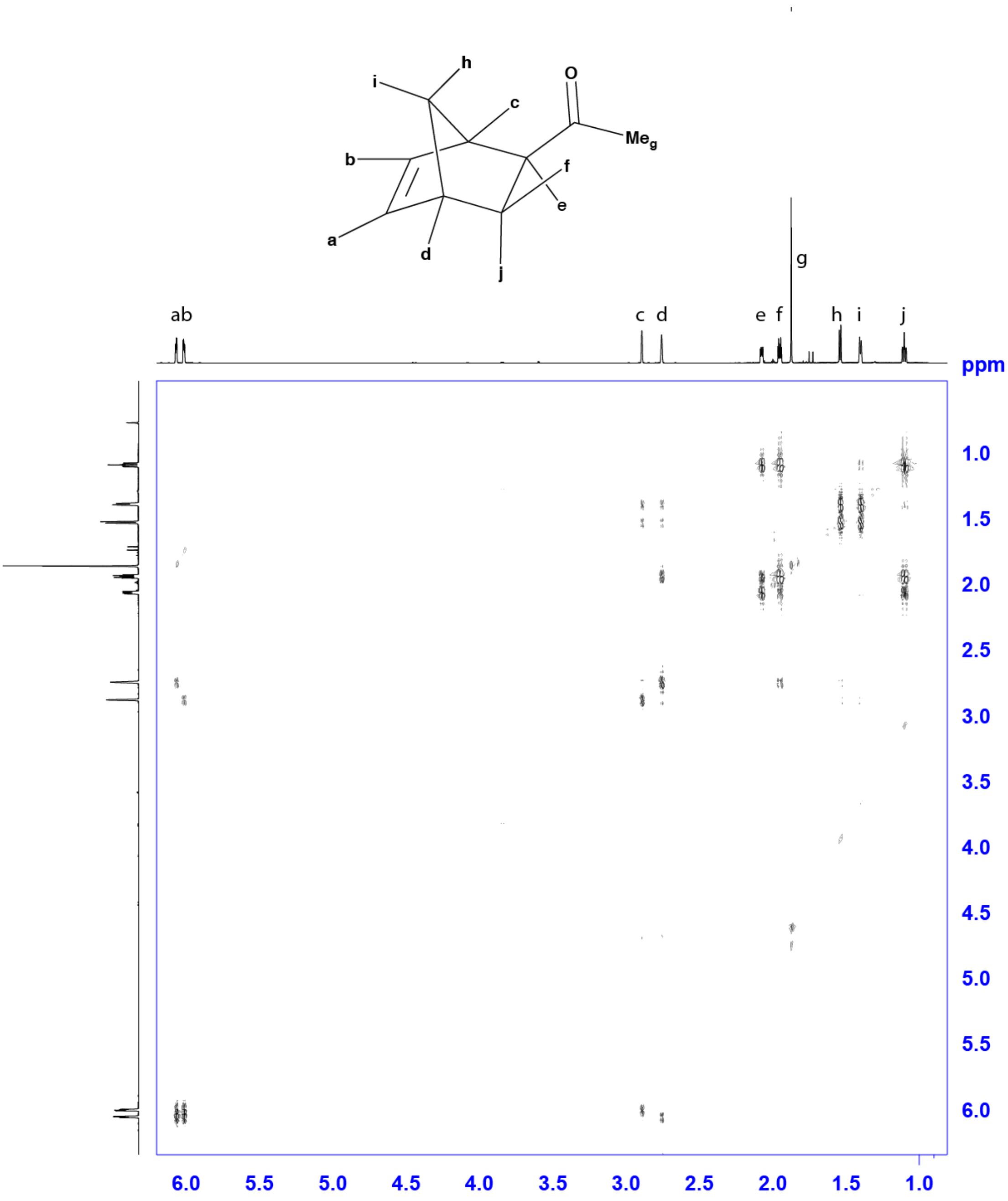

Figure S6. COSY spectrum of *exo* adduct **4$_{CPD}$-x**.

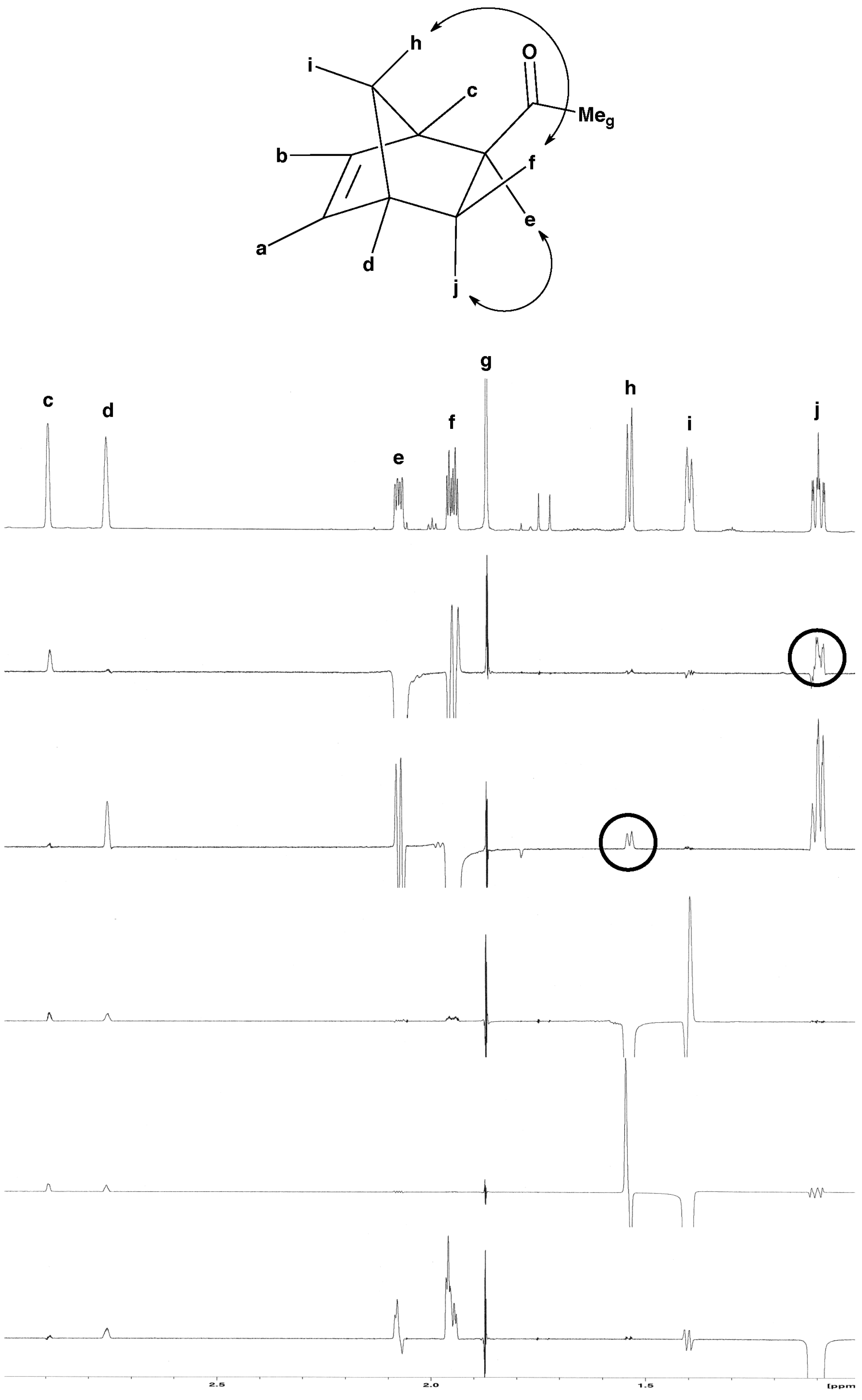

Figure S7 Array of nOe spectra of *exo* adduct $\mathbf{4_{CPD}}$**-x**.

6 ^{1}H and ^{13}C NMR spectra of compounds 1-20

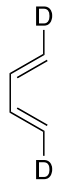

1

800Mz ^{1}H $CDCl_3$

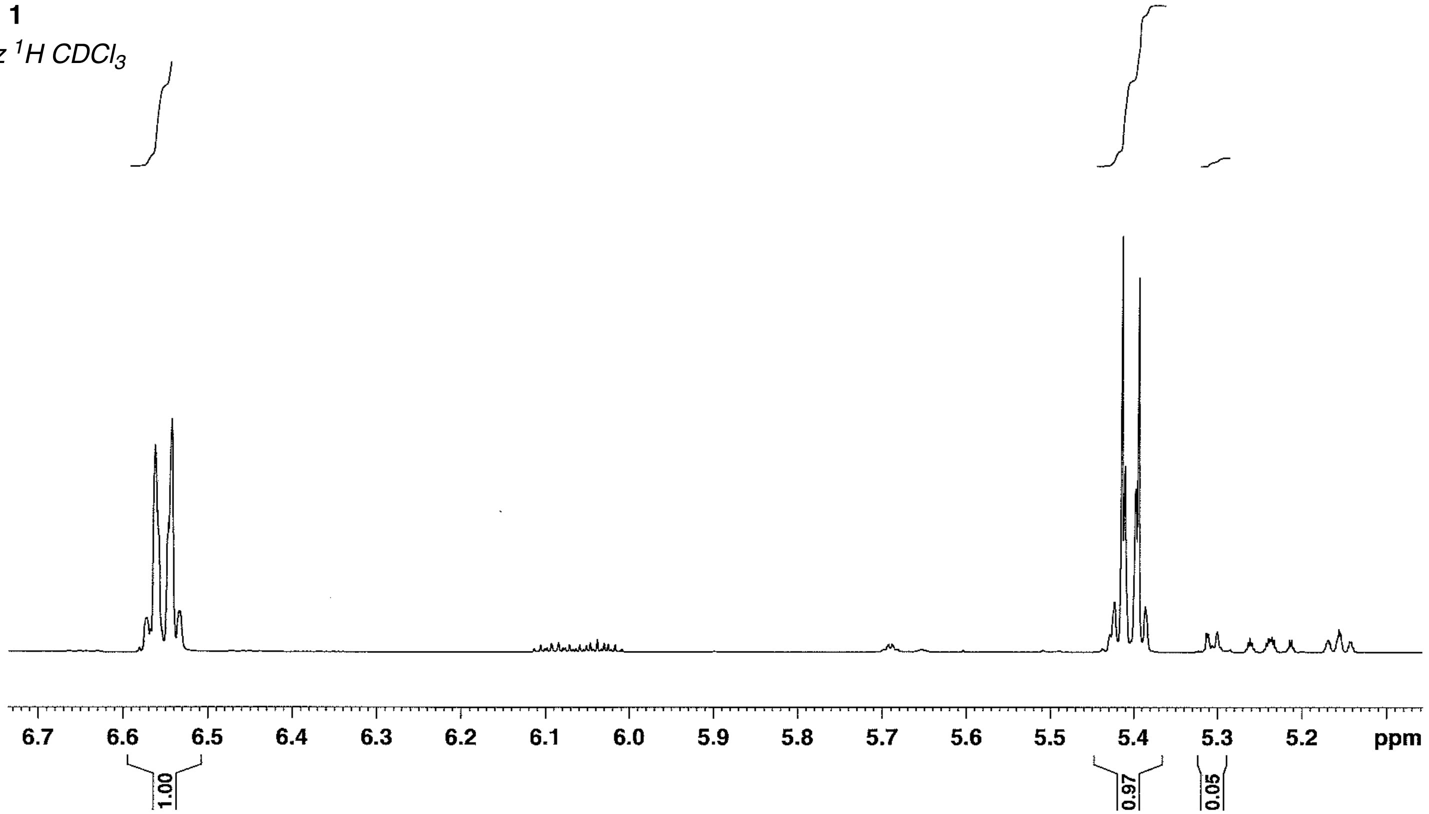

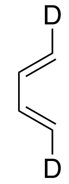

1

200Mz ^{13}C $CDCl_3$

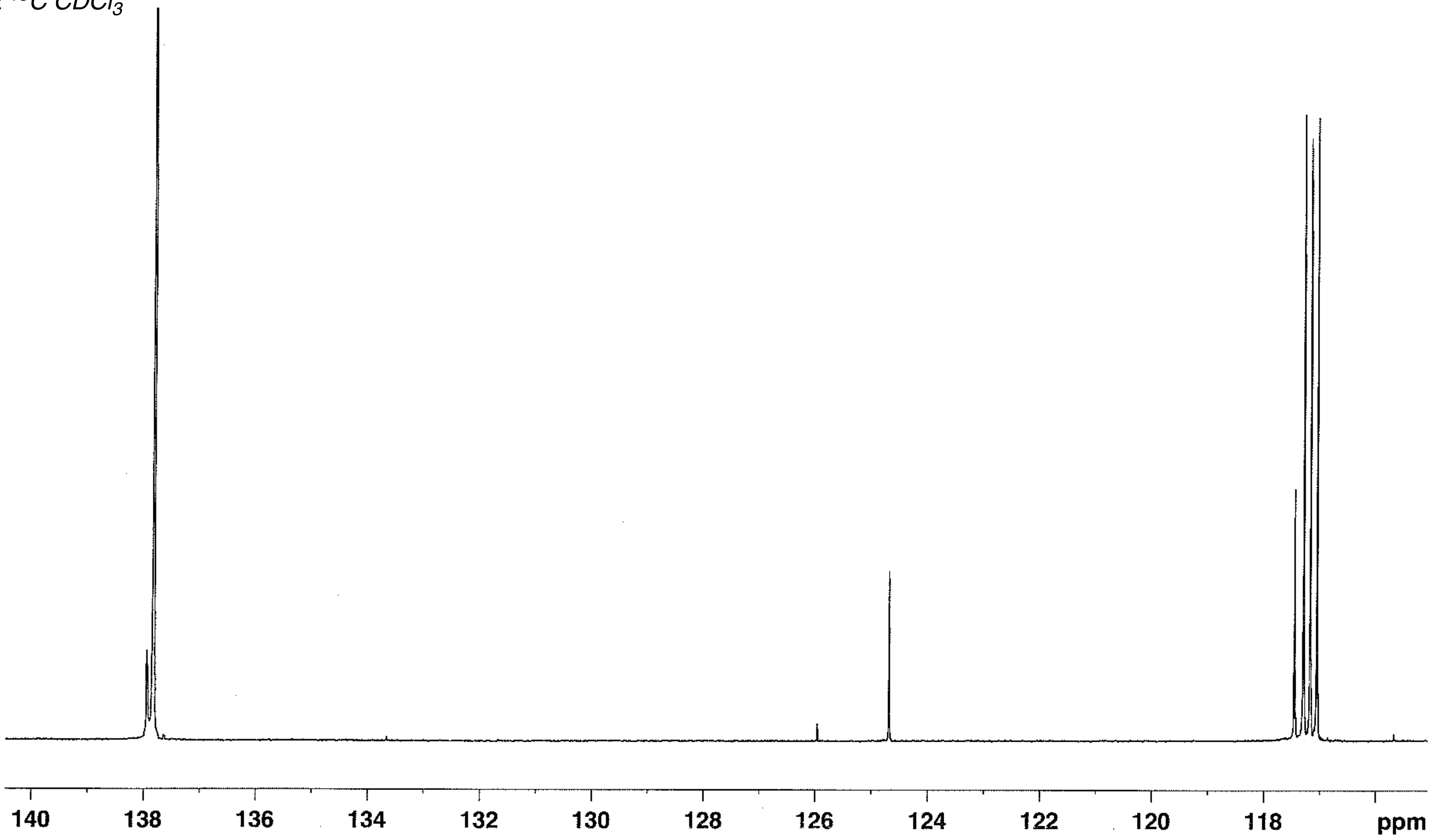

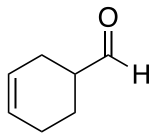

3_{BD}

800Mz ^1H C_6D_6/DMSO-d_6 (49:1)

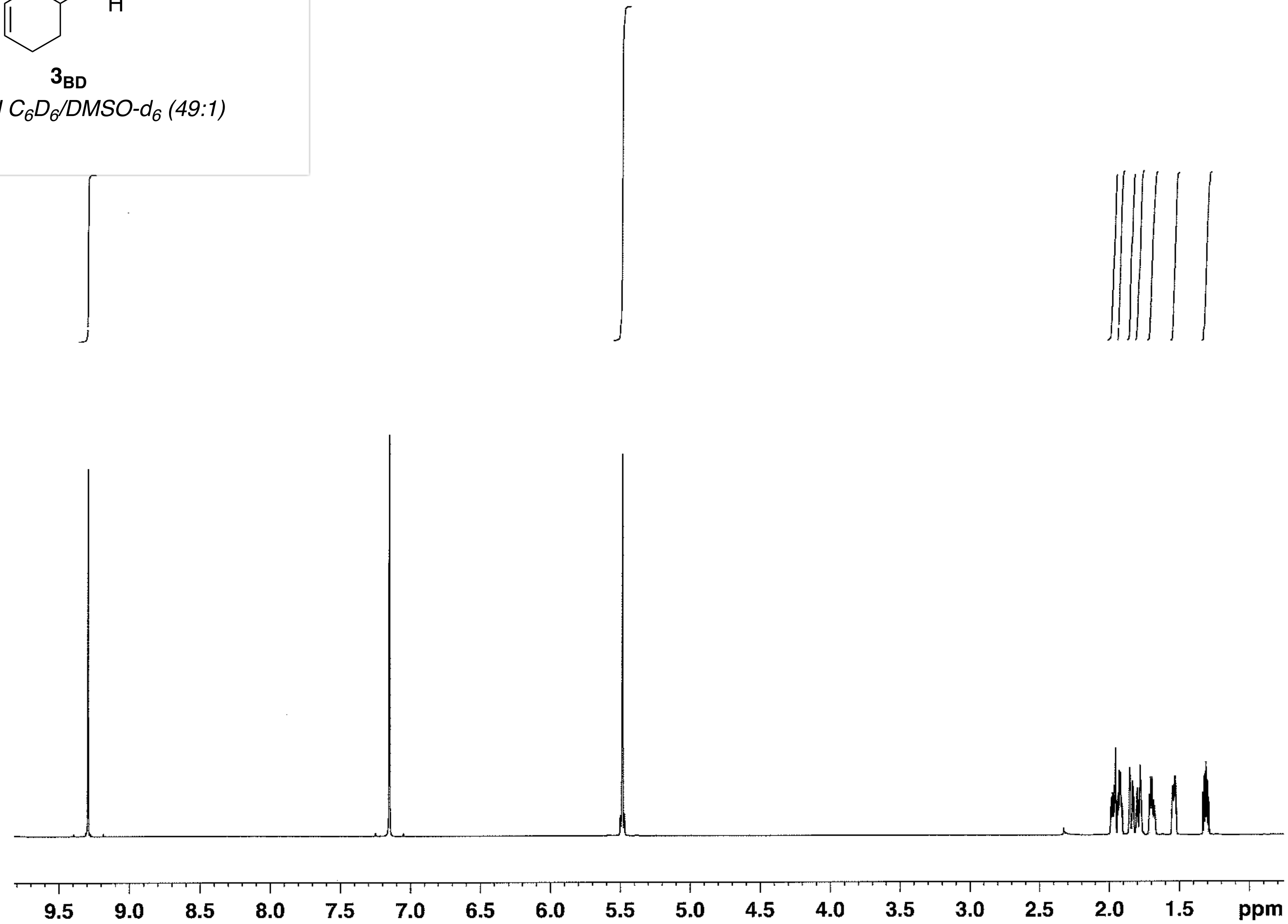

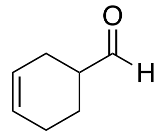

3_{BD}

200Mz ^{13}C C_6D_6/DMSO-d_6 (49:1)

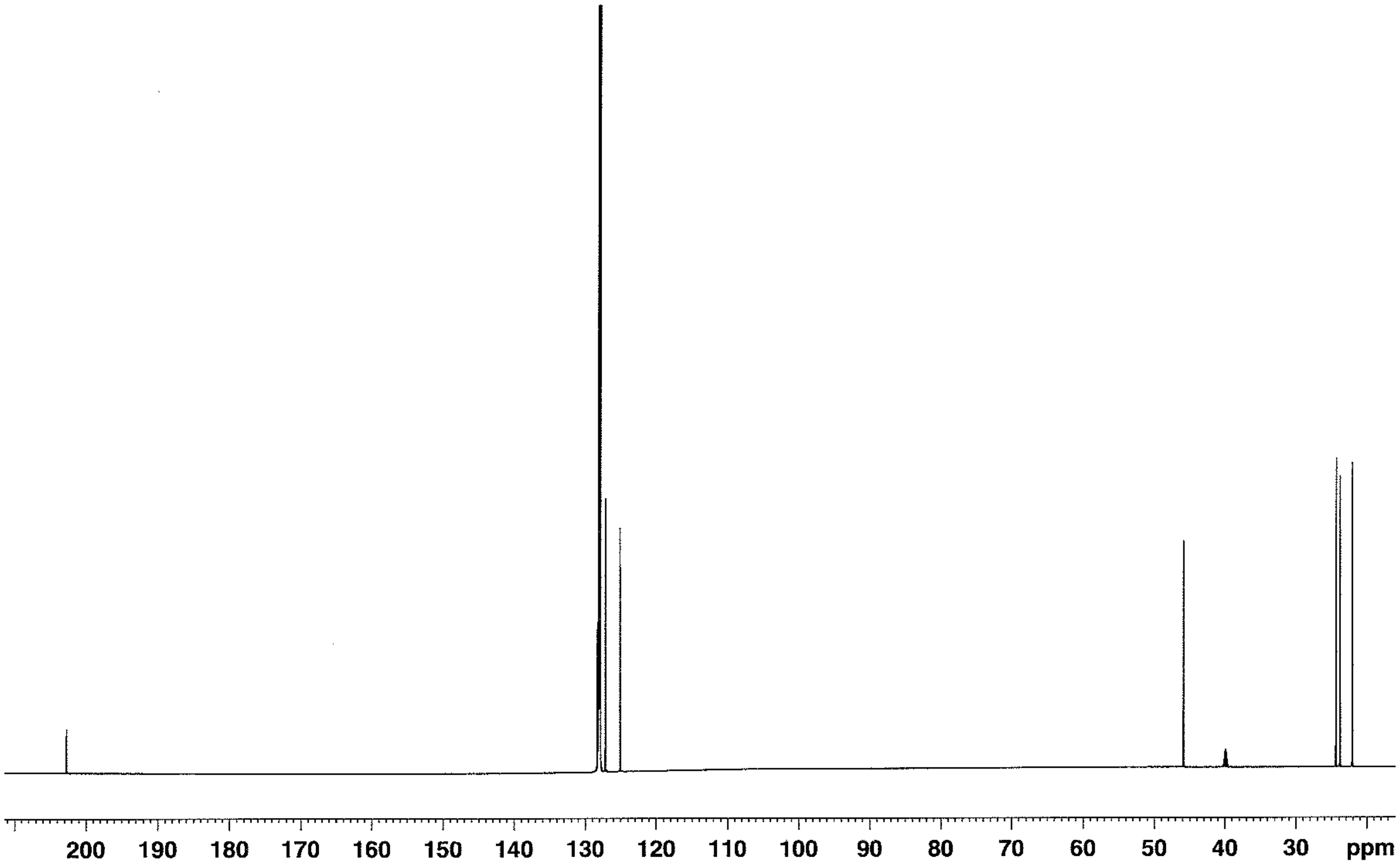

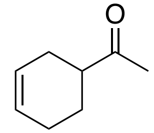

$\mathbf{4_{BD}}$

800Mz ^{1}H C_6D_6/DMSO-d_6 (24:1)

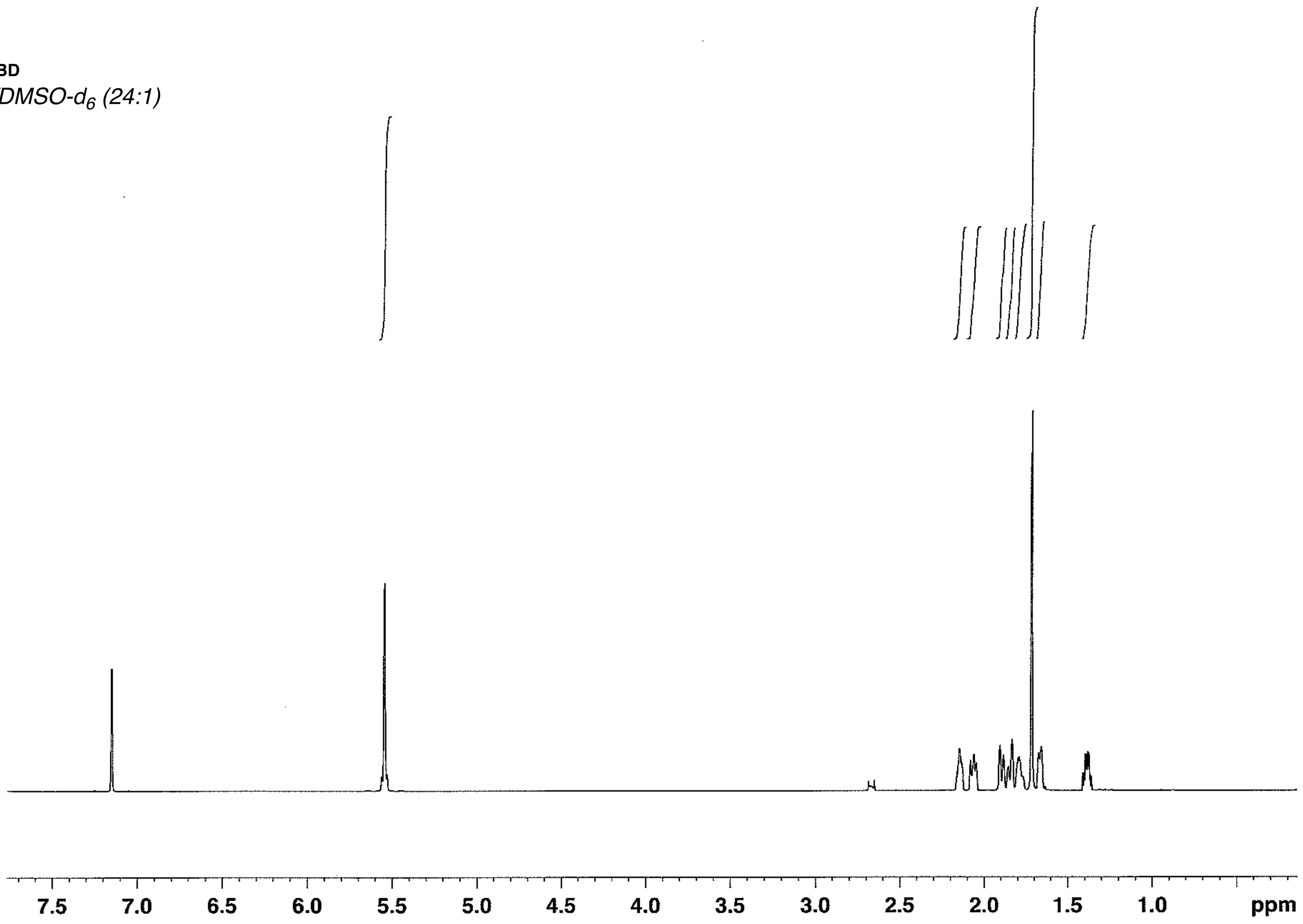

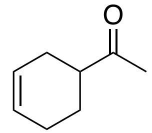

4_{BD}

200Mz ^{13}C C_6D_6/DMSO-d_6 (24:1)

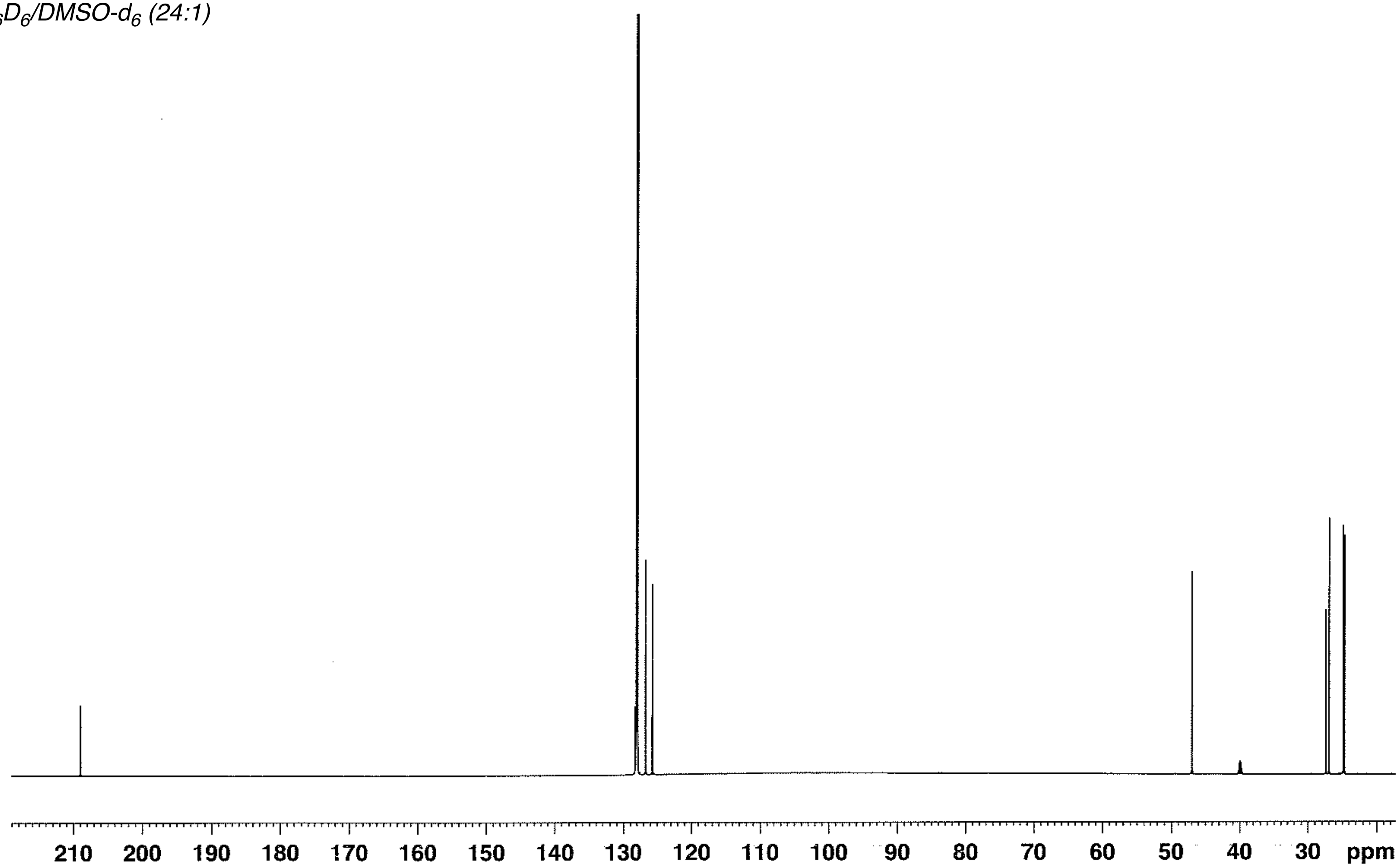

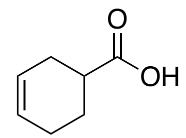

6_{BD}

800Mz ^1H C_6D_6/DMSO-d_6 (49:1)

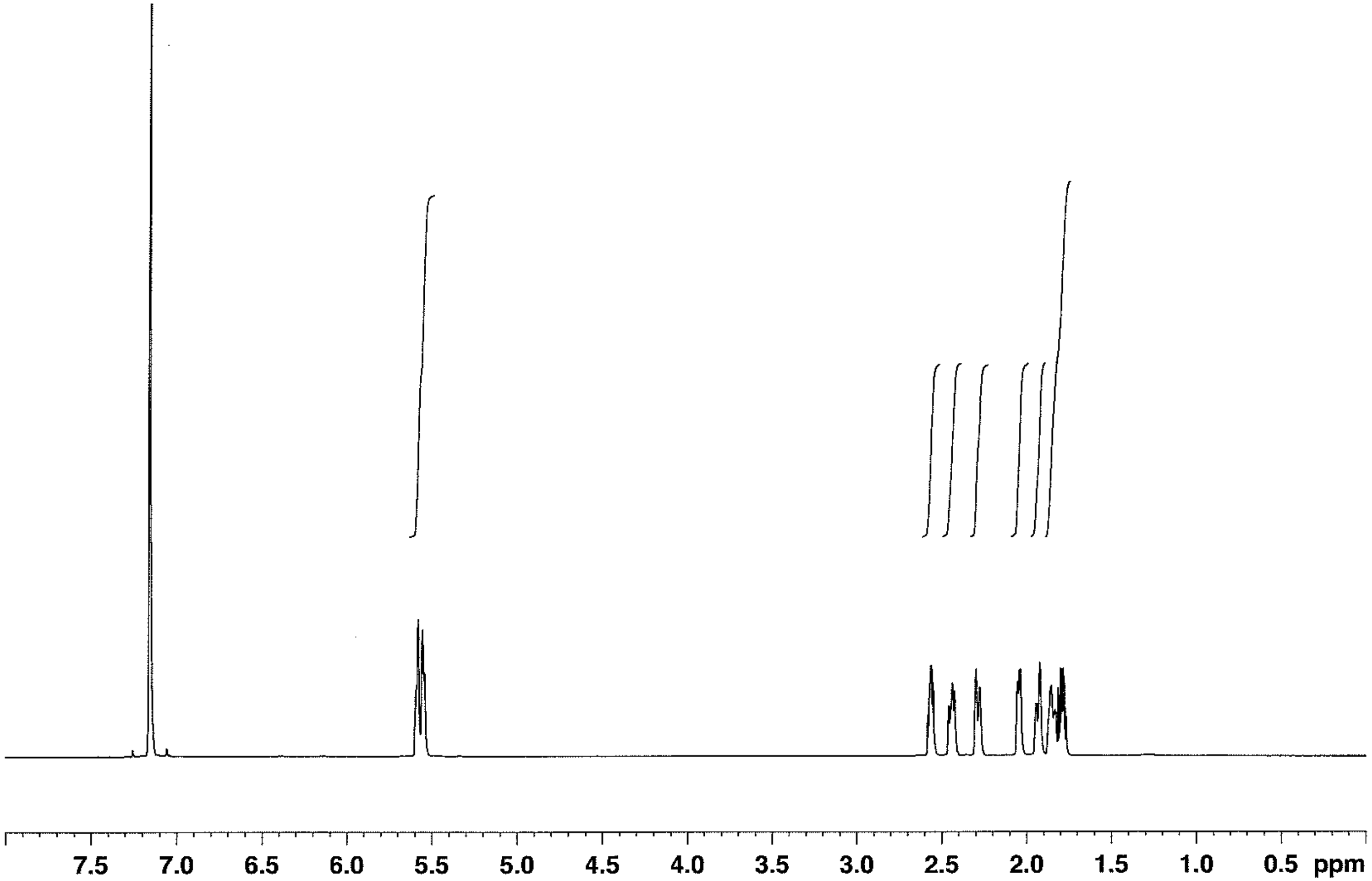

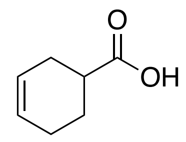

6$_{BD}$

200Mz ^{13}C C_6D_6/DMSO-d_6 (49:1)

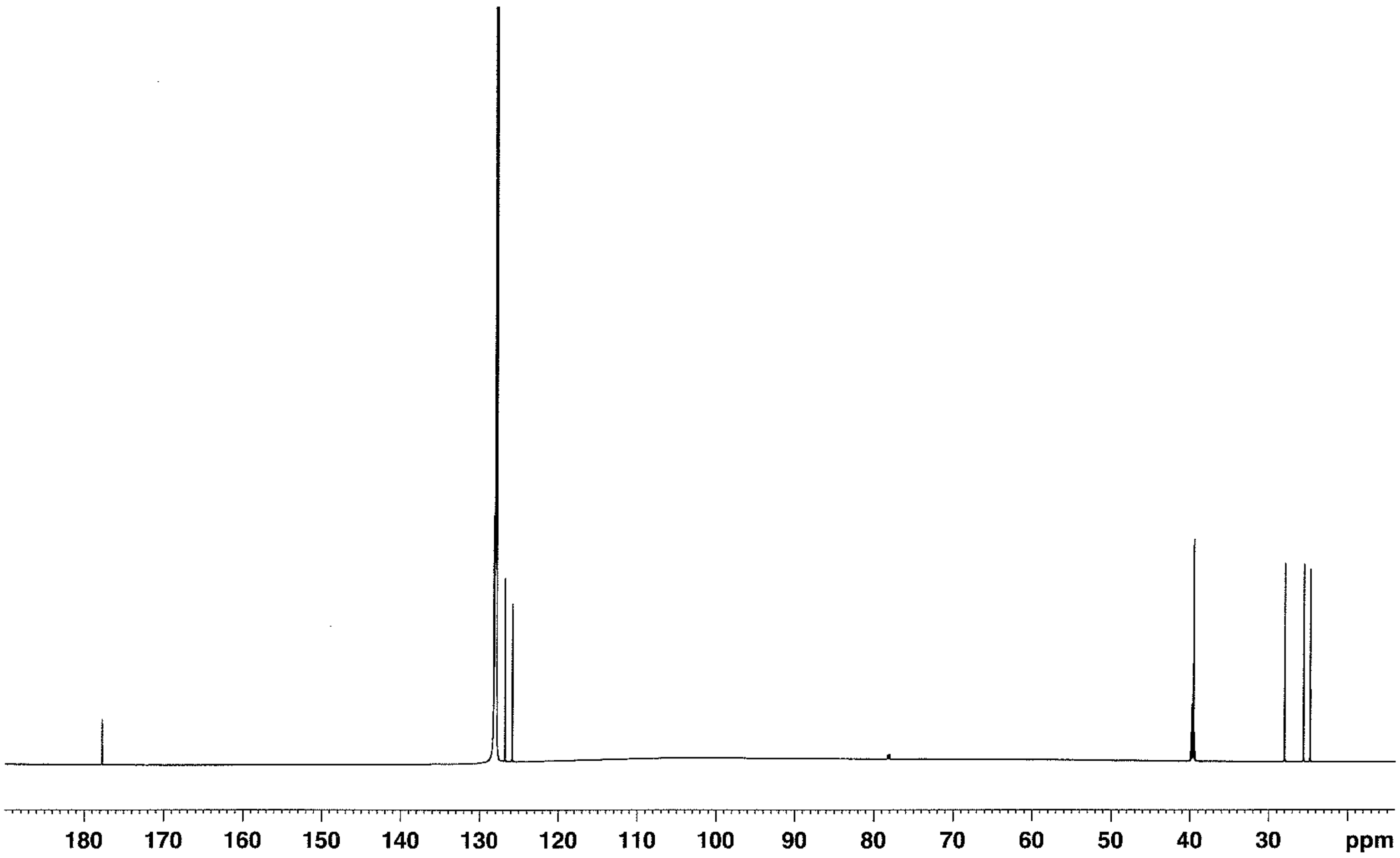

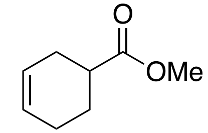

7$_{\mathbf{BD}}$

800Mz ¹H DMSO-d$_6$

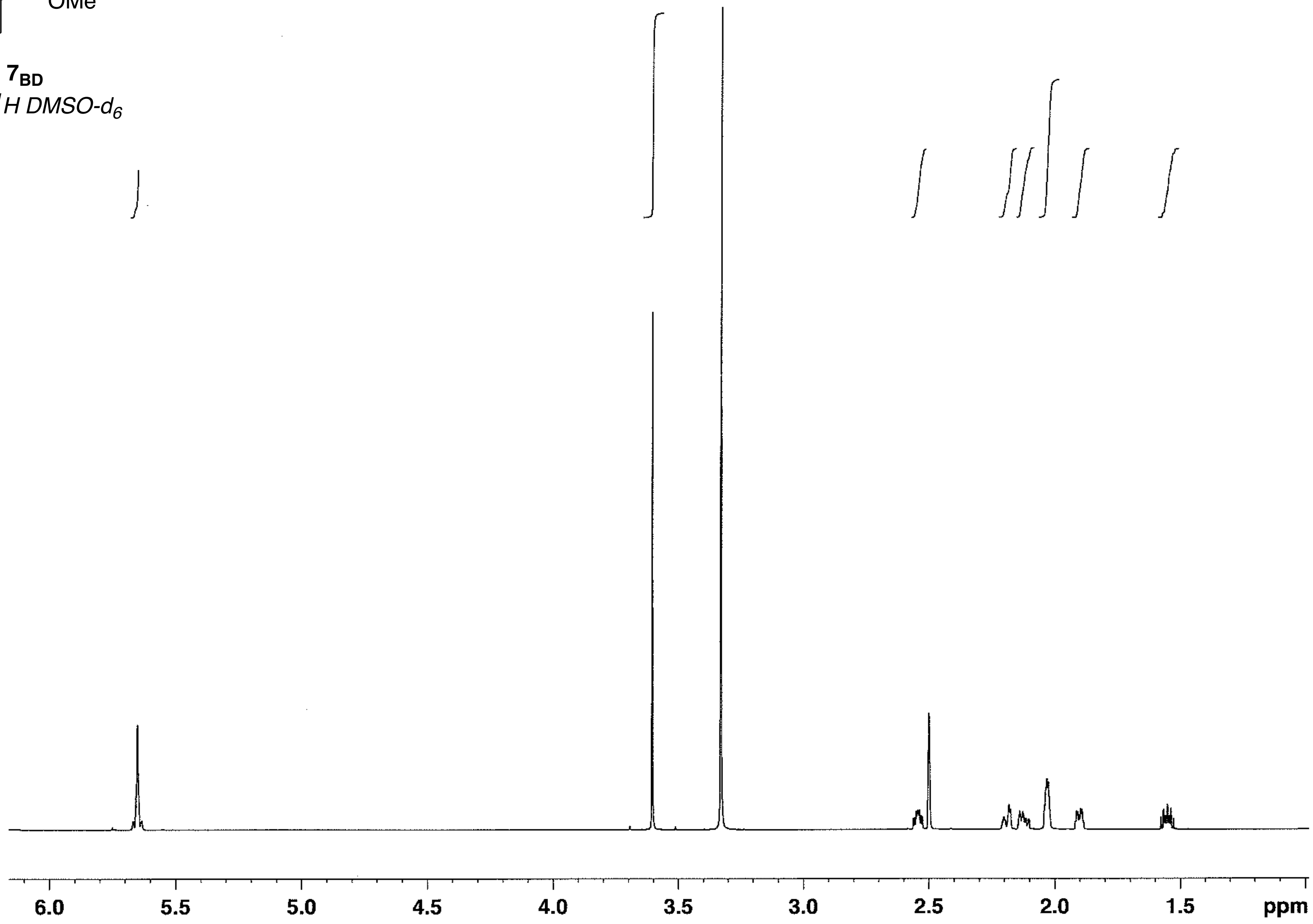

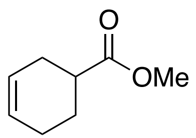

7$_{BD}$

200Mz ^{13}C DMSO-d$_6$

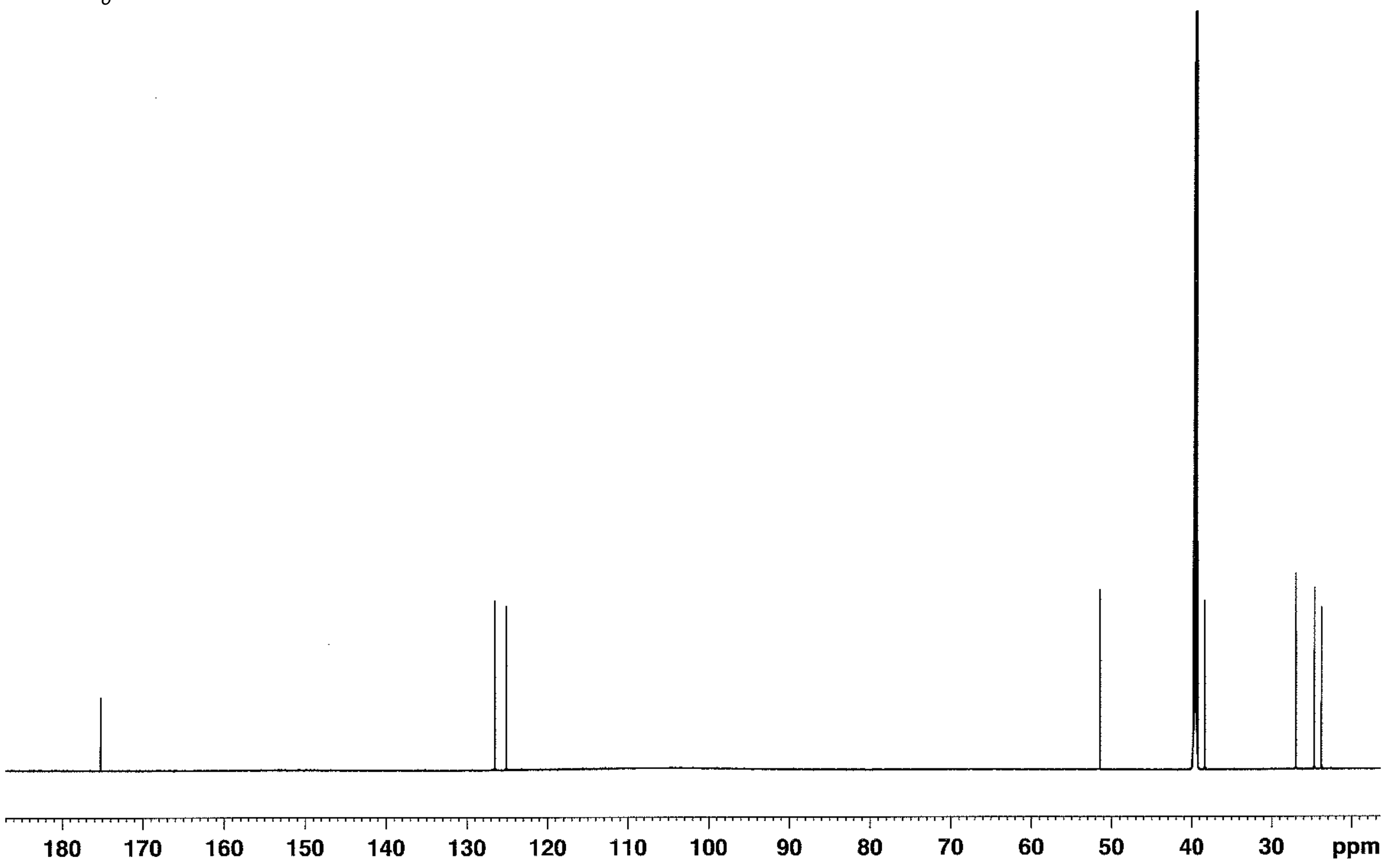

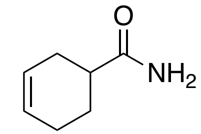

8_{BD}

800Mz ^{1}H acetone-d_6

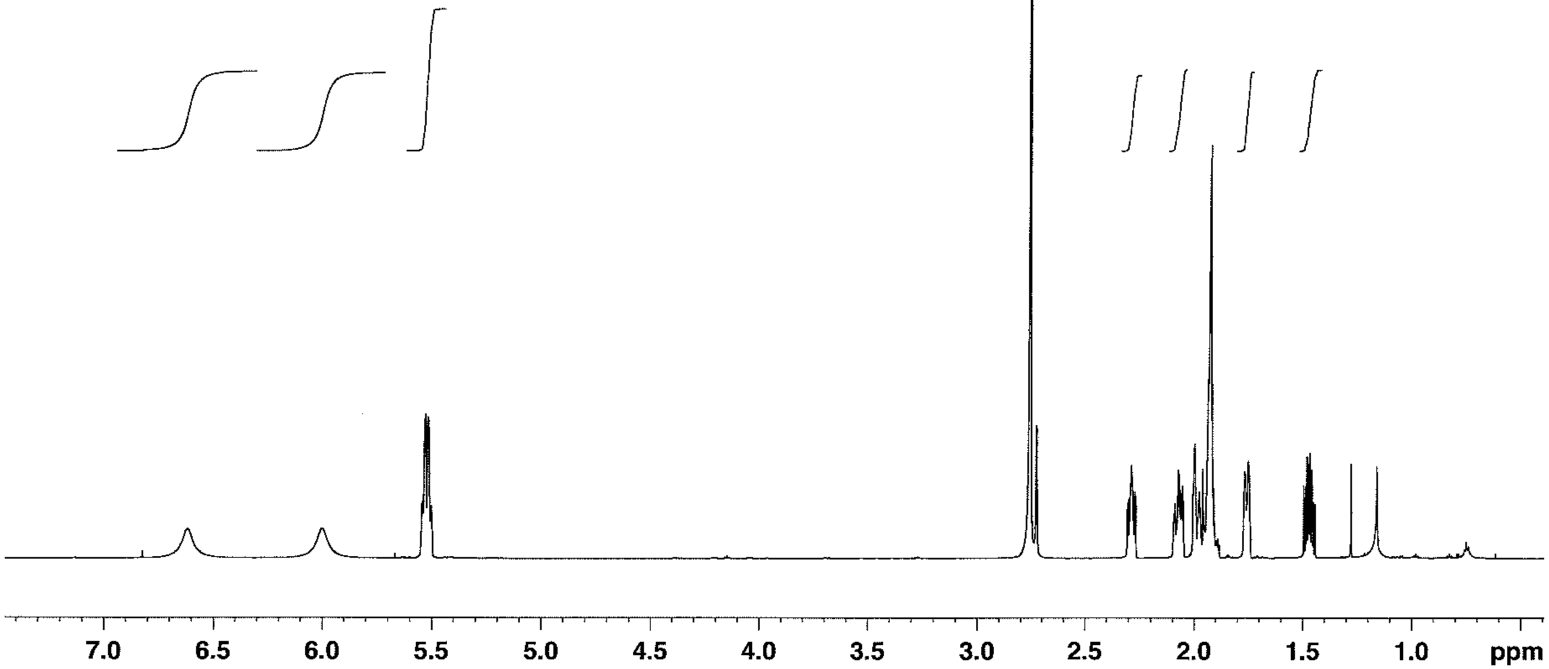

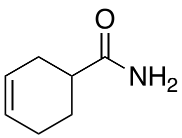

8_{BD}

200Mz ^{13}C acetone-d_6

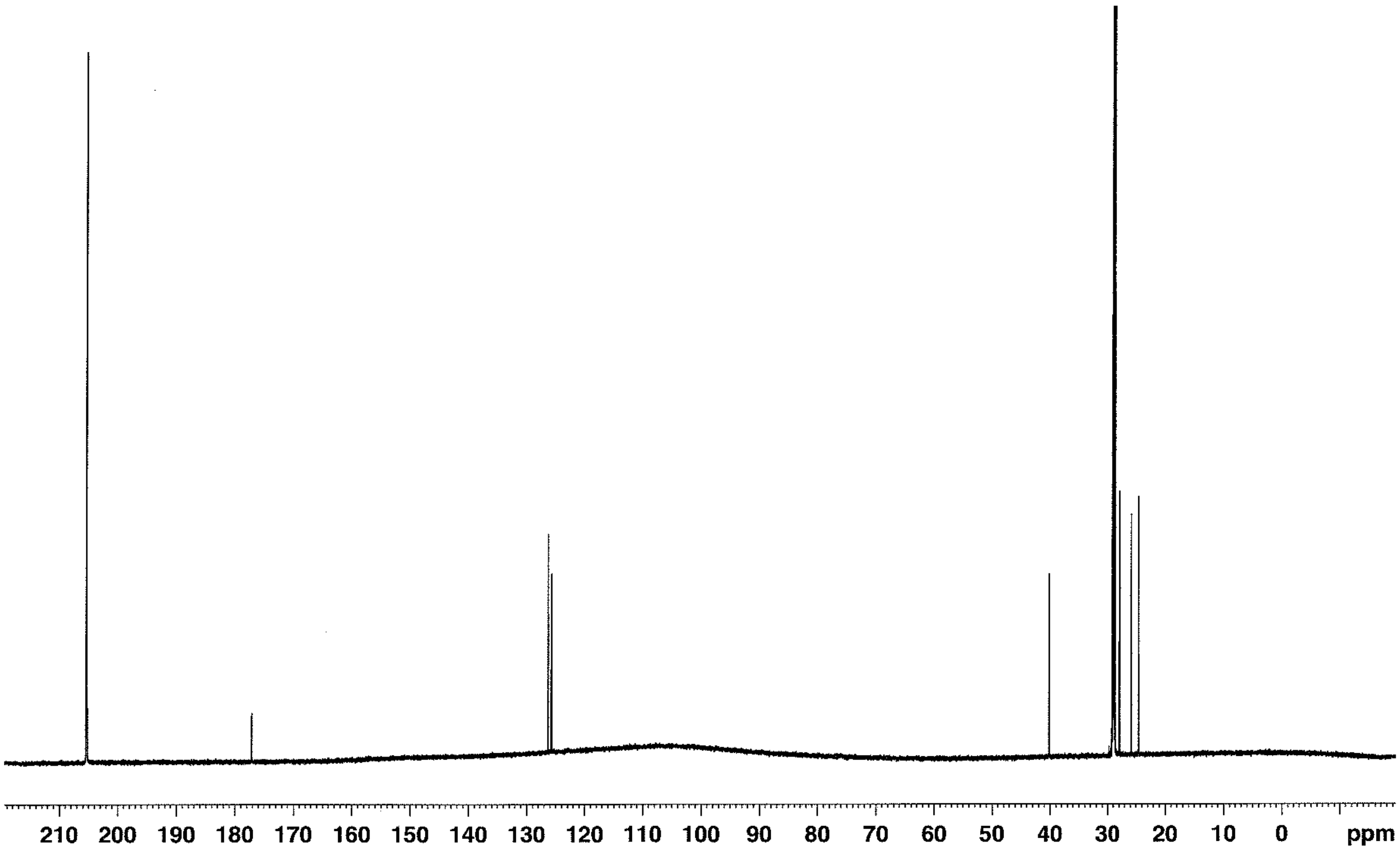

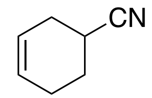

9$_{BD}$

800Mz ^{1}H $CDCl_3$

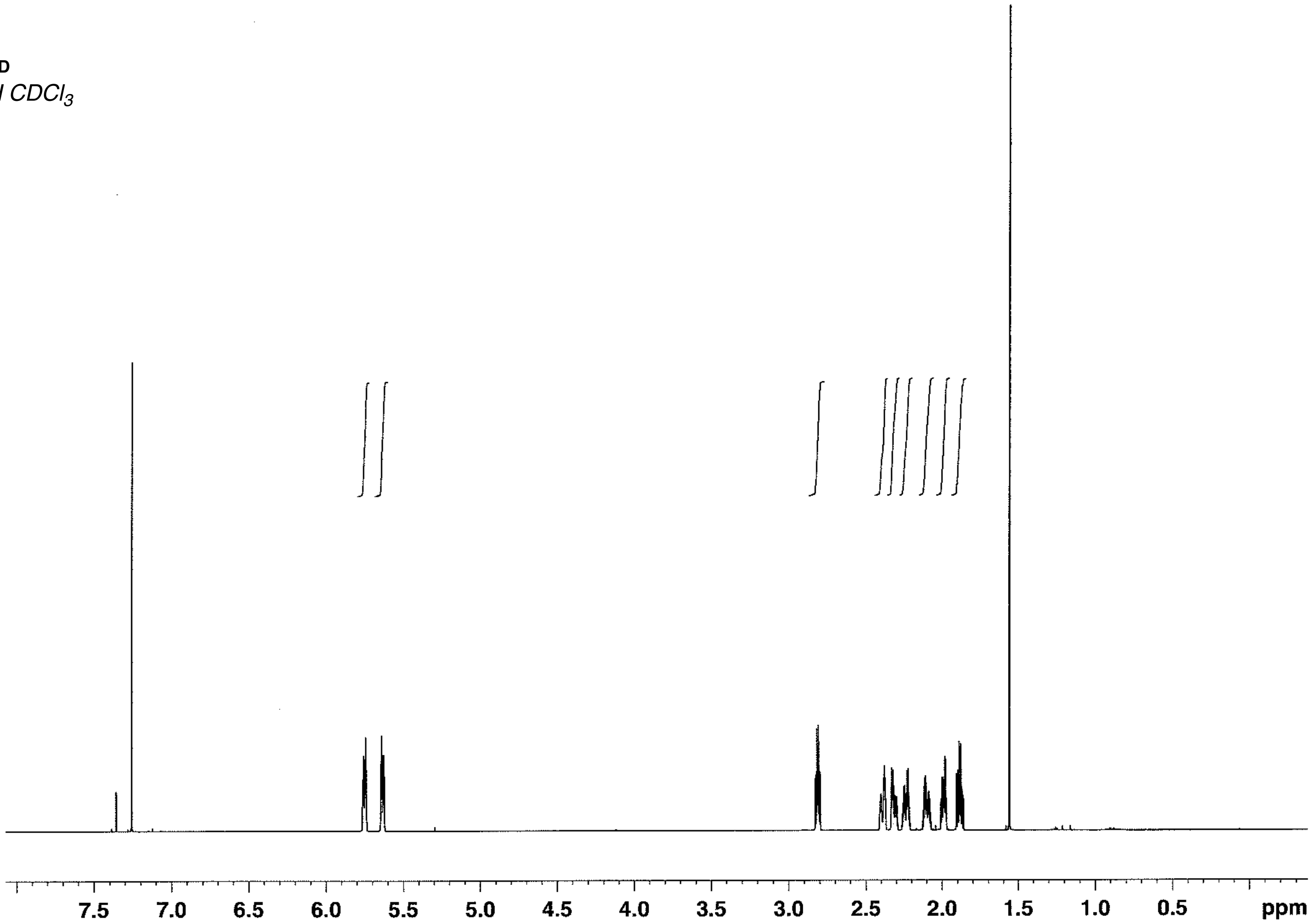

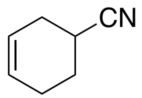

9$_{BD}$

200Mz ^{13}C $CDCl_3$

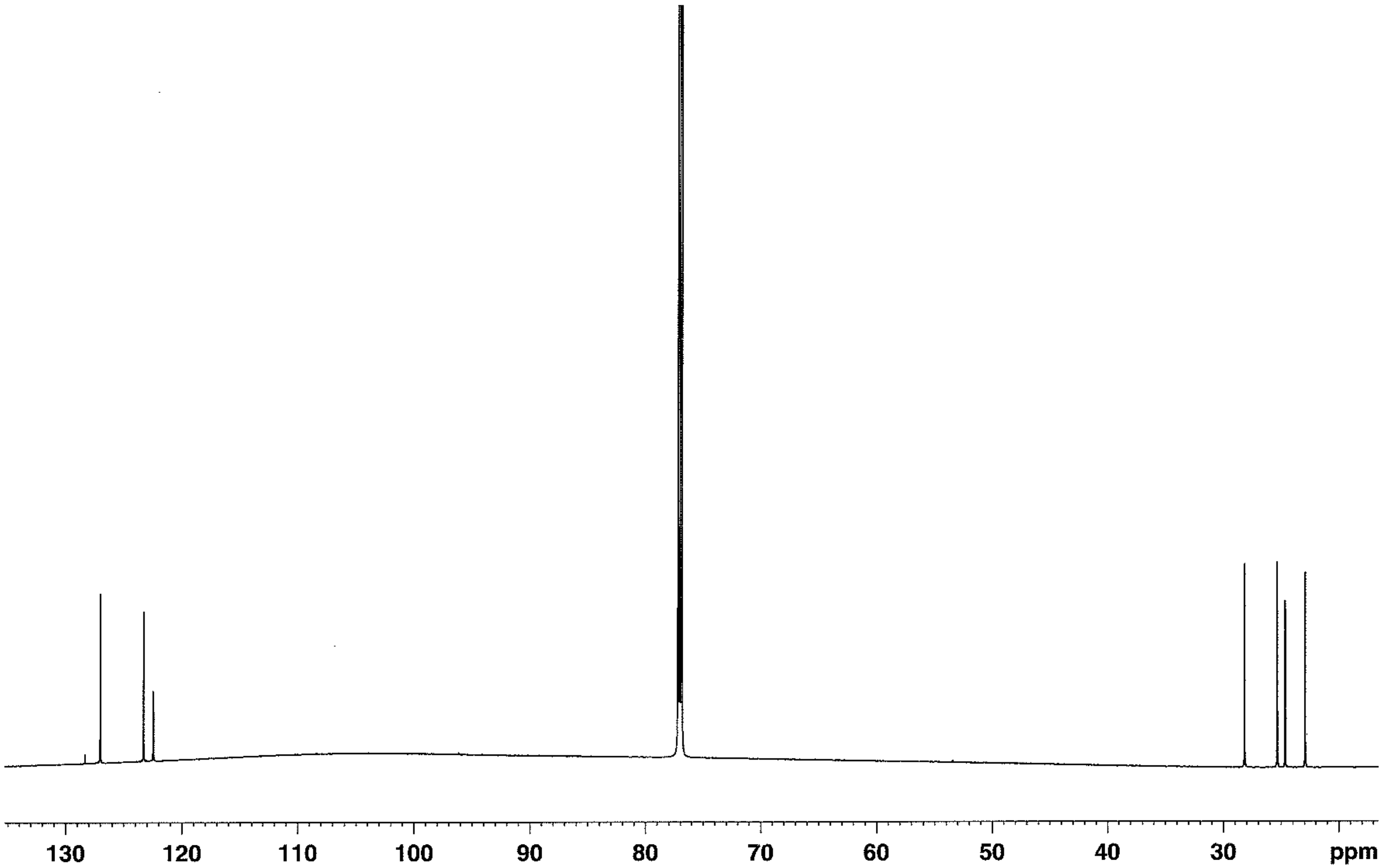

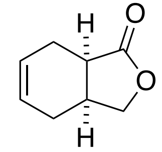

10$_{BD}$
800Mz ^1H $CDCl_3$

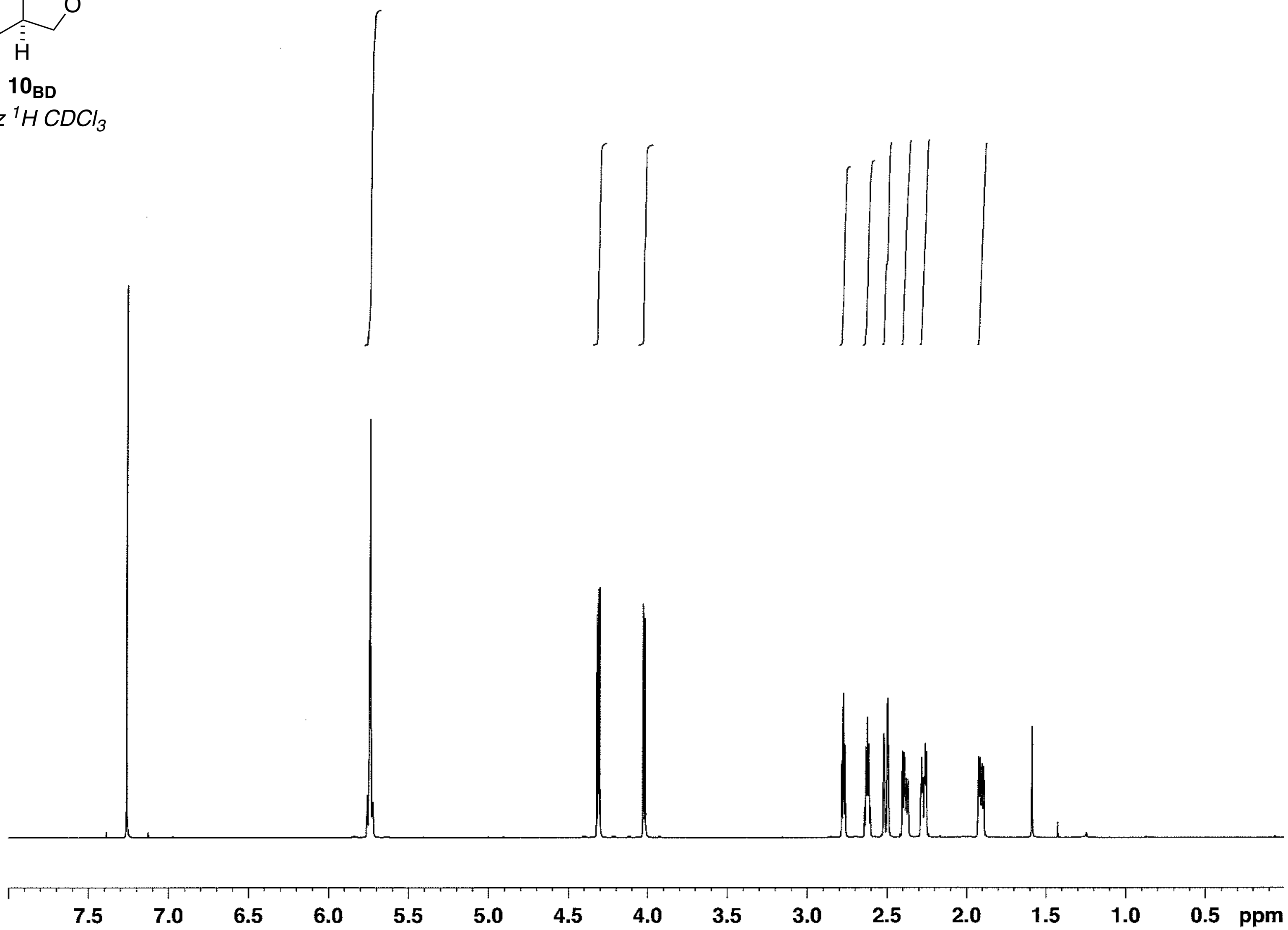

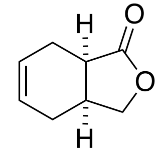

10_{BD}

200Mz ^{13}C $CDCl_3$

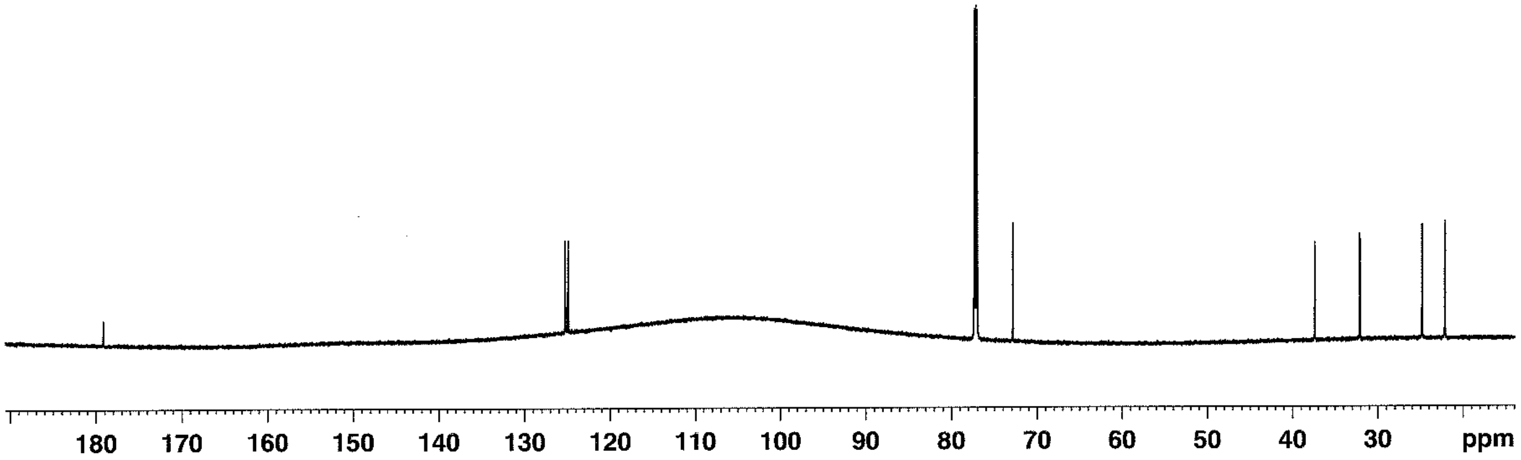

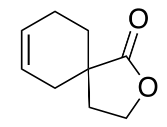

11_{BD}

800Mz ^1H toluene-d_8

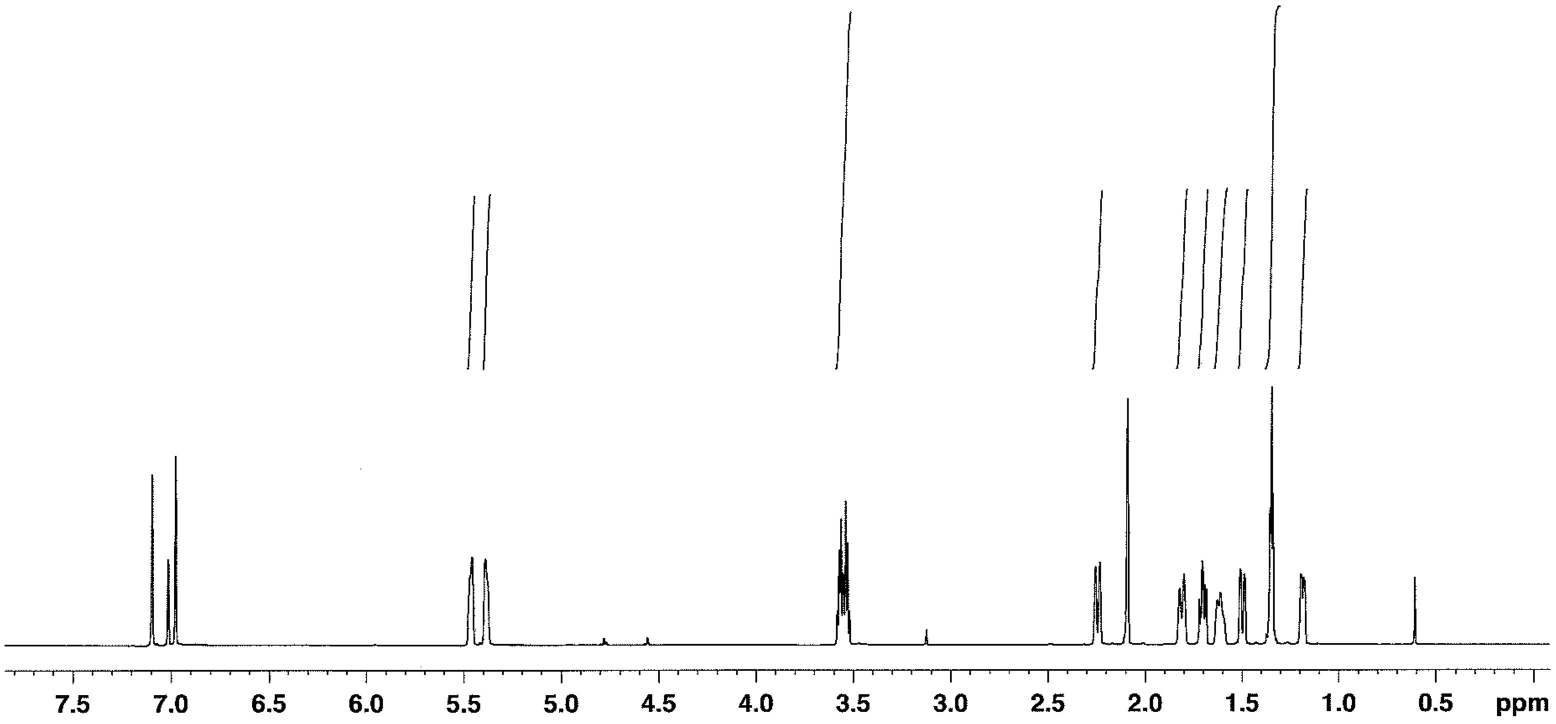

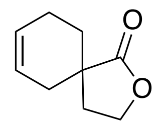

11_{BD}

200Mz ^{13}C toluene-d_8

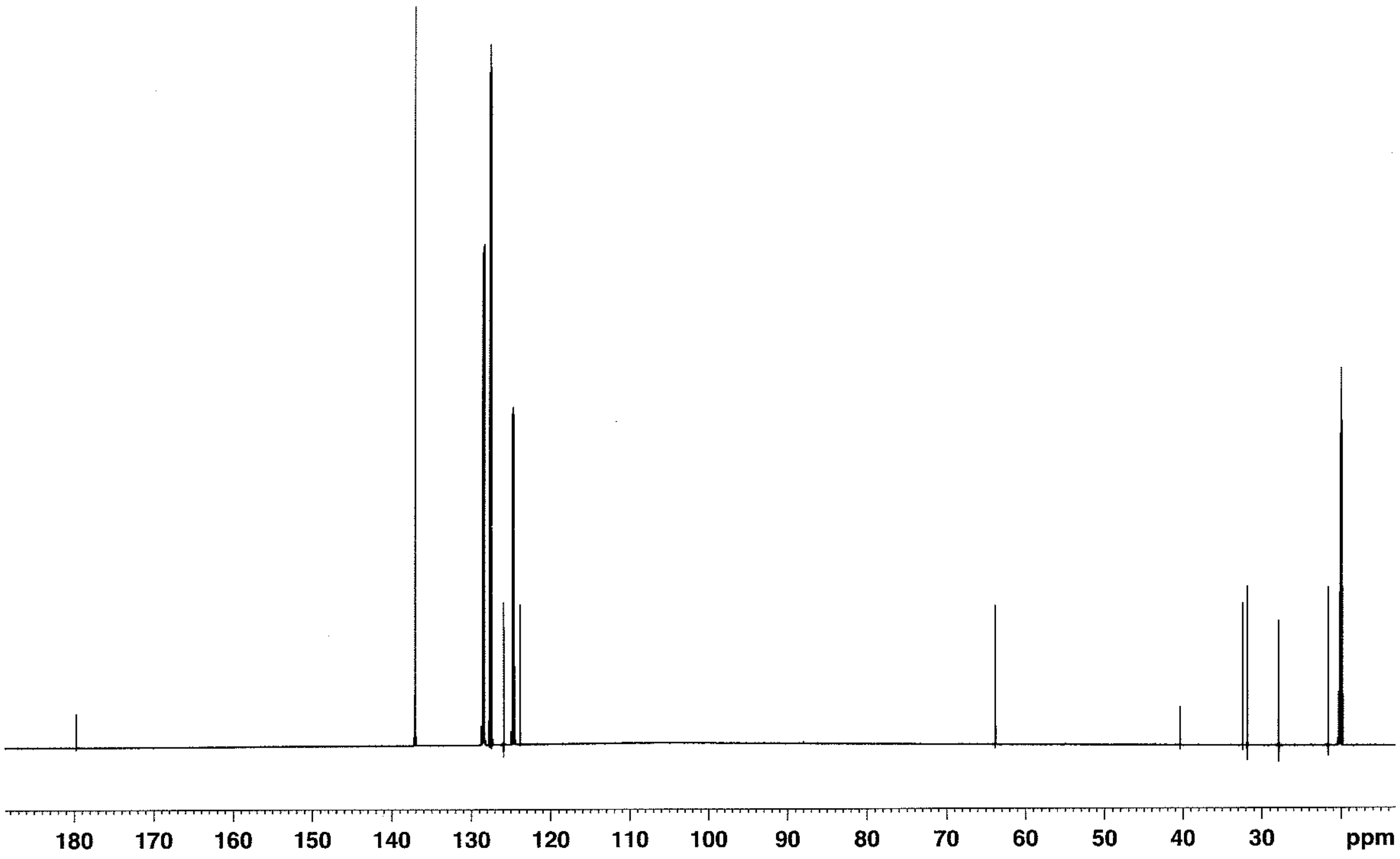

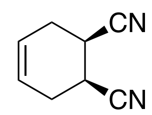

14_{BD}

800Mz ^1H C_6D_6/DMSO-d_6 (49:1)

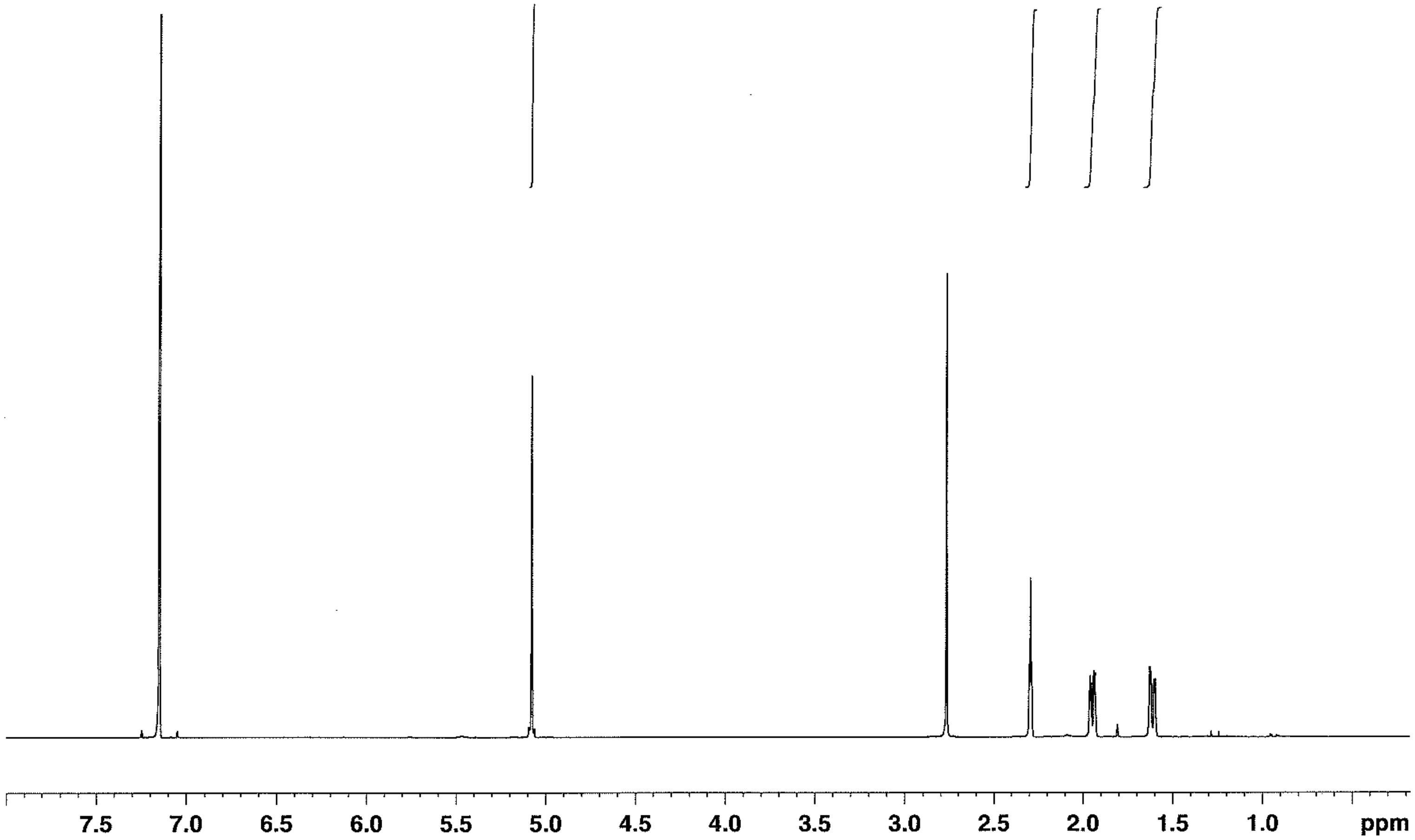

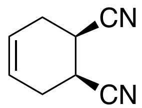

14_{BD}

200Mz ^{13}C C_6D_6/DMSO-d_6 (49:1)

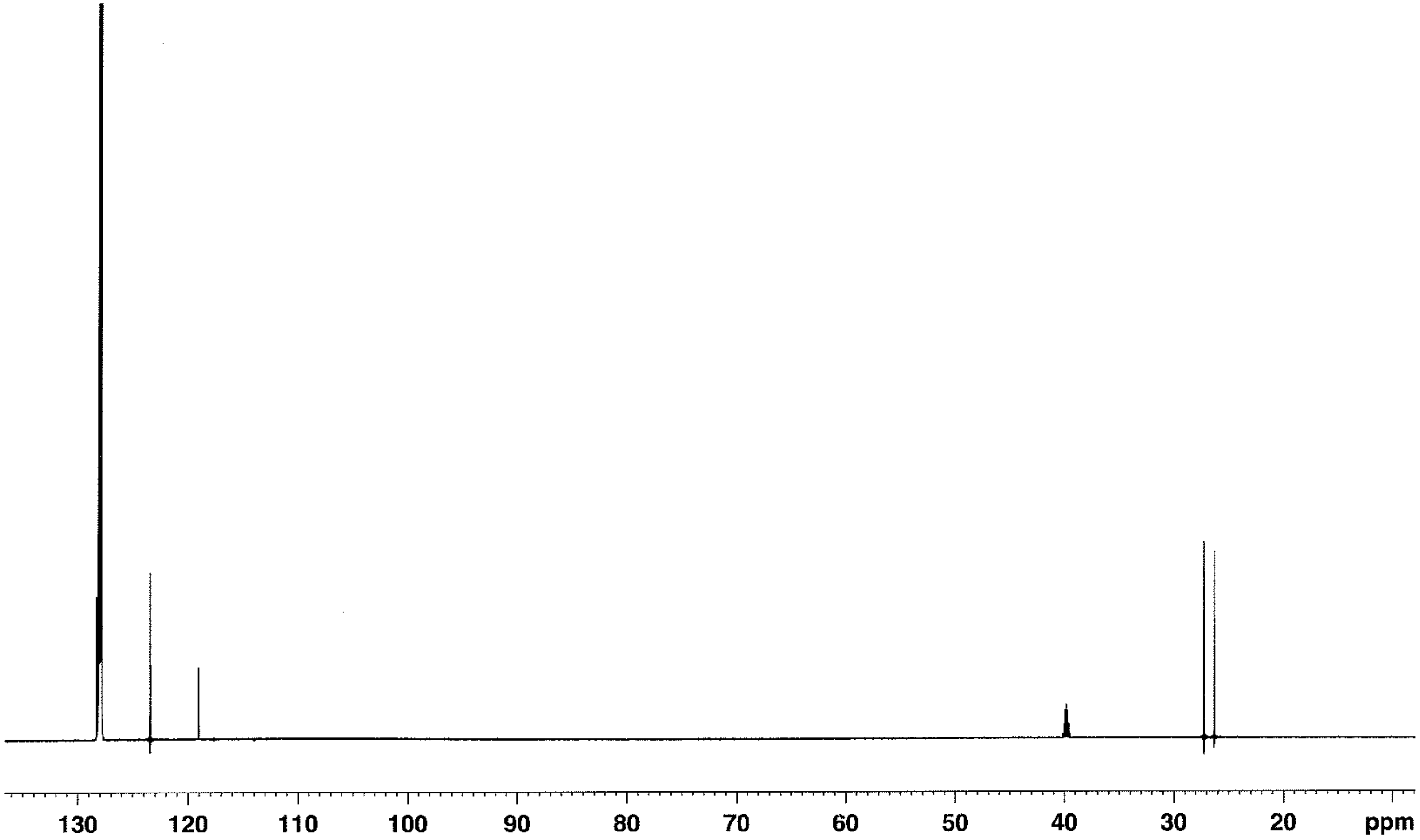

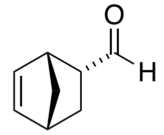

$\mathbf{3_{CPD}}$**-n**

800Mz ^{1}H C_6D_6

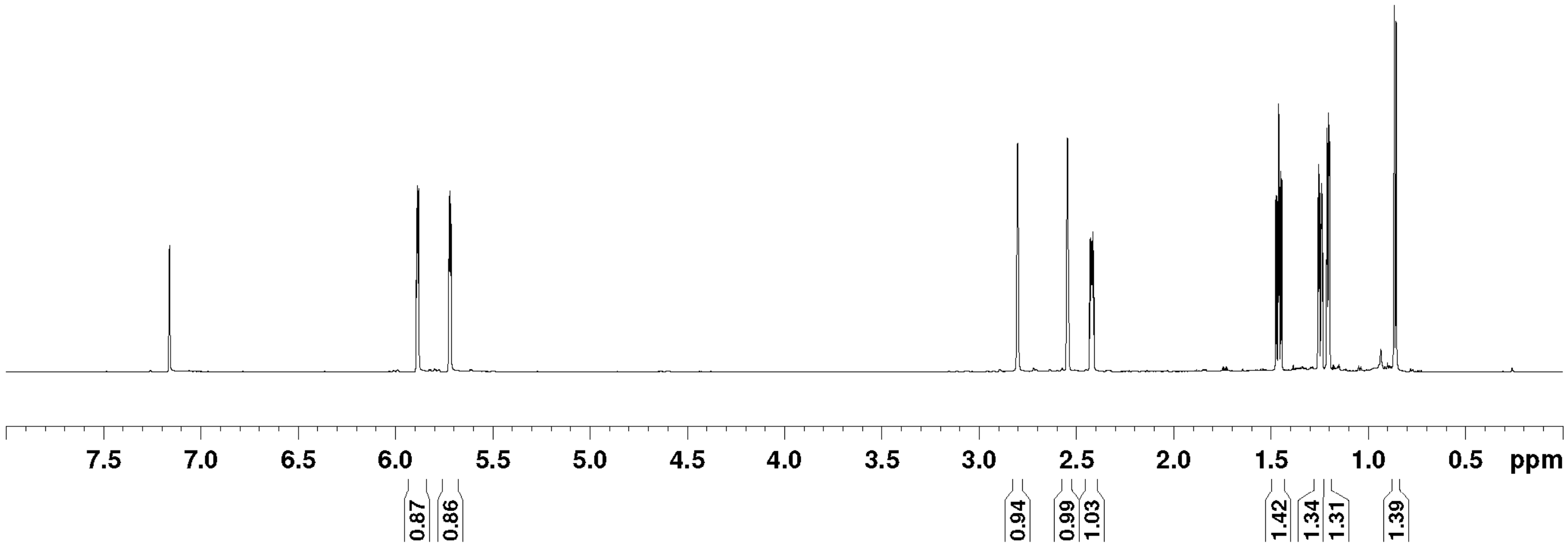

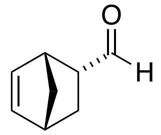

3_{CPD}-n

200Mz ^{13}C C_6D_6

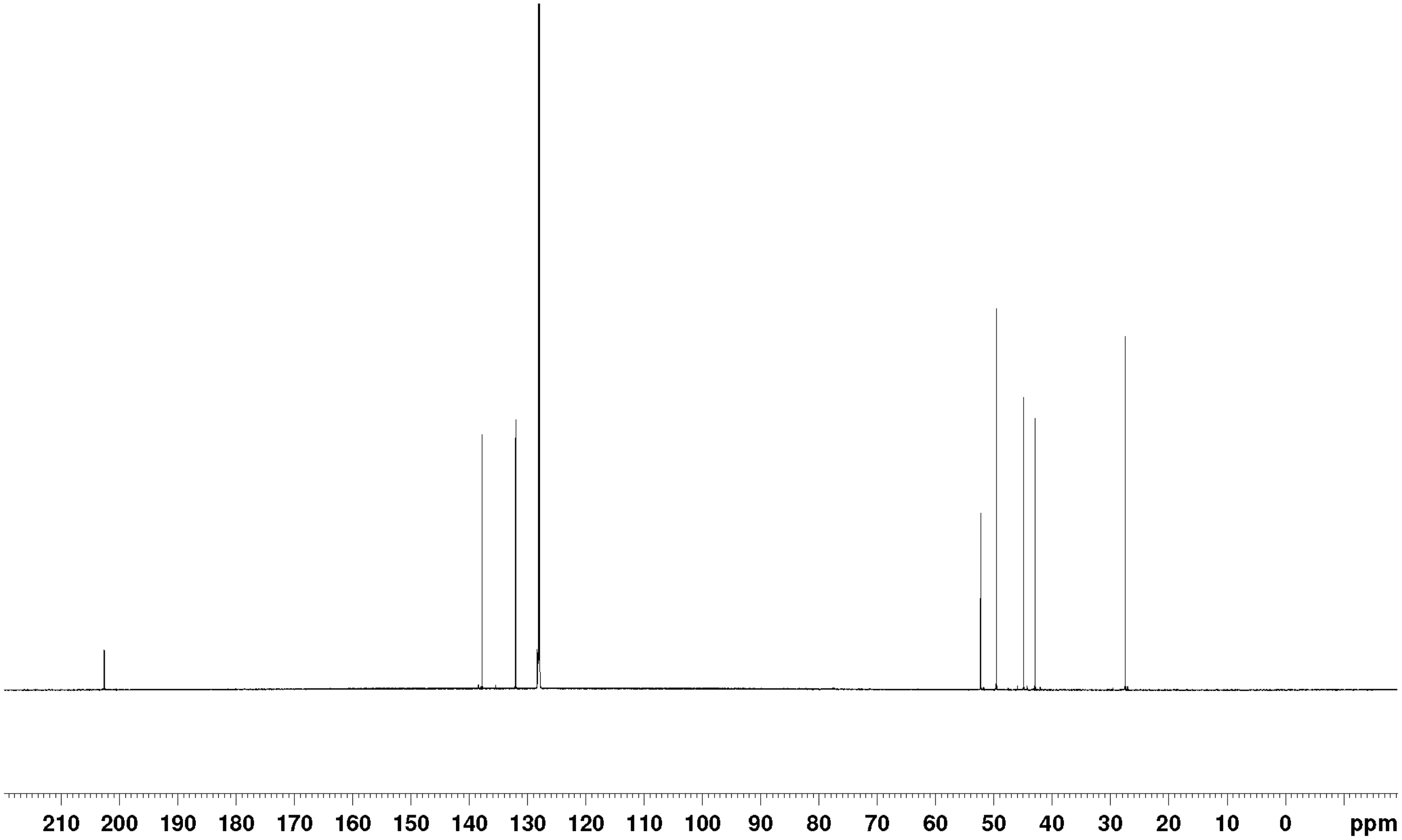

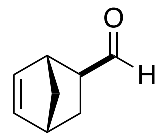

3_{CPD}-x

800Mz ^{1}H C_6D_6

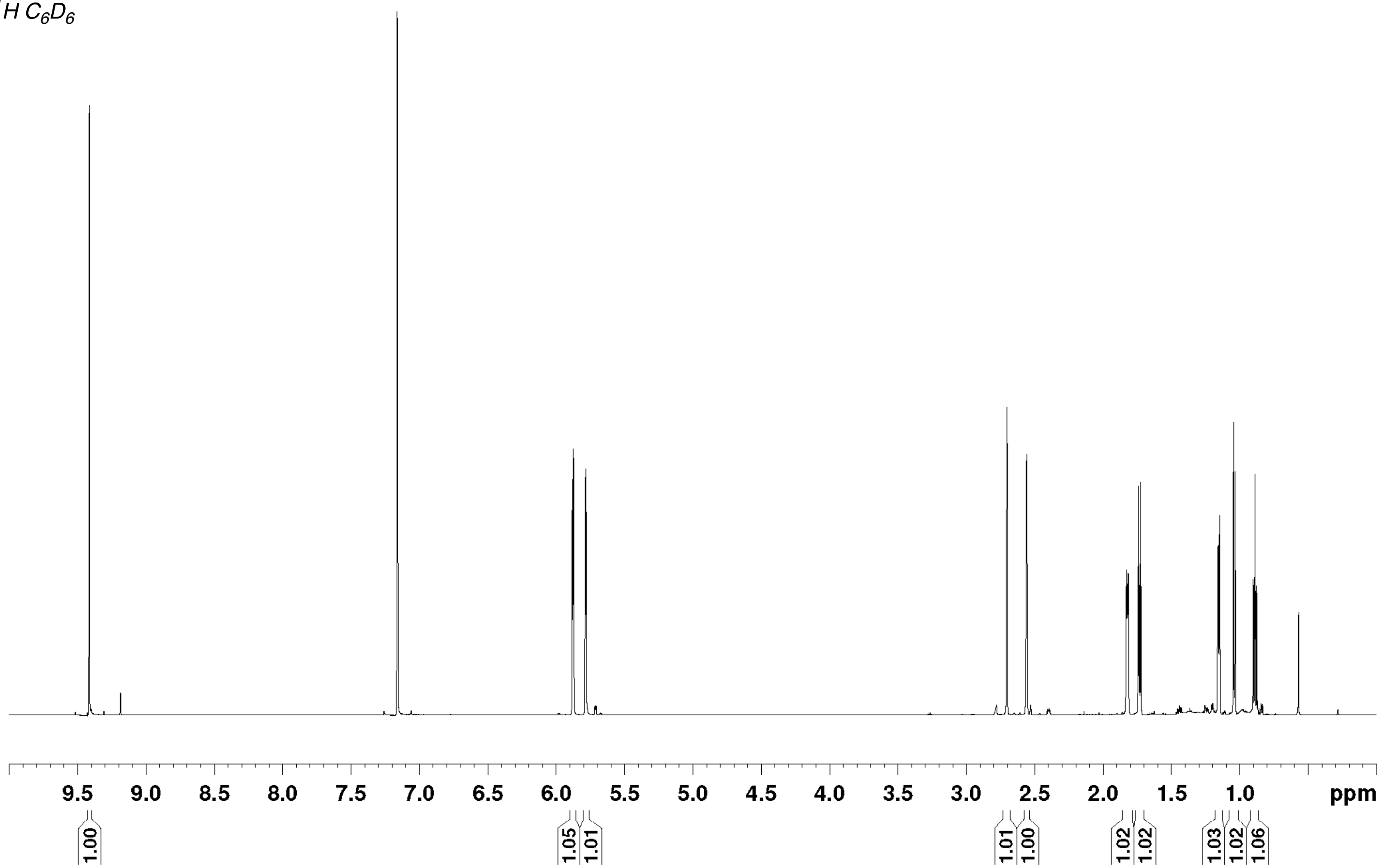

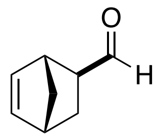

$\mathbf{3_{CPD}}$**-x**

200Mz ^{13}C C_6D_6

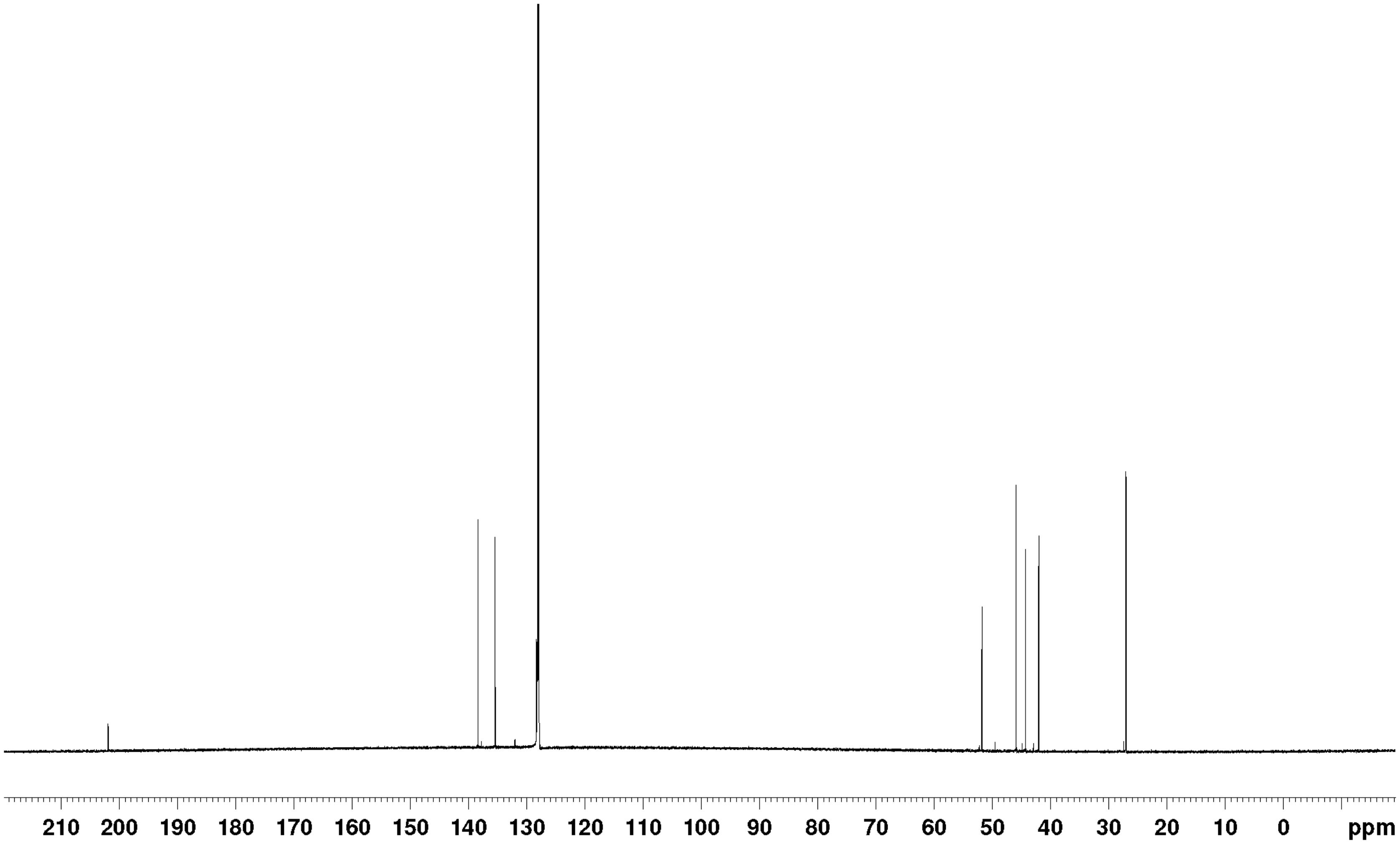

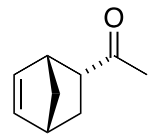

4_{CPD}-n

800Mz ^{1}H C_6D_6

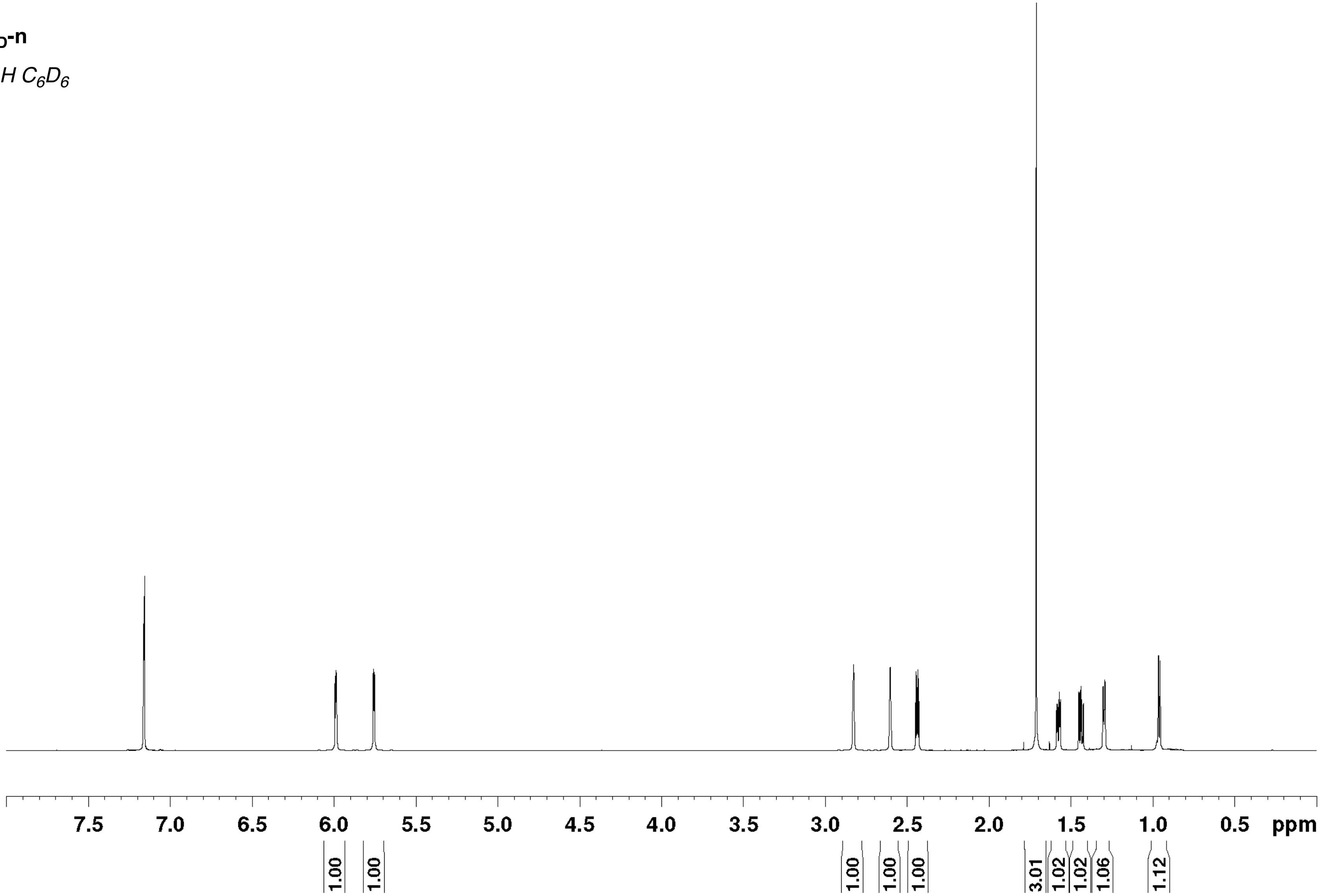

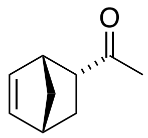

4_{CPD}-n

200Mz ^{13}C C_6D_6

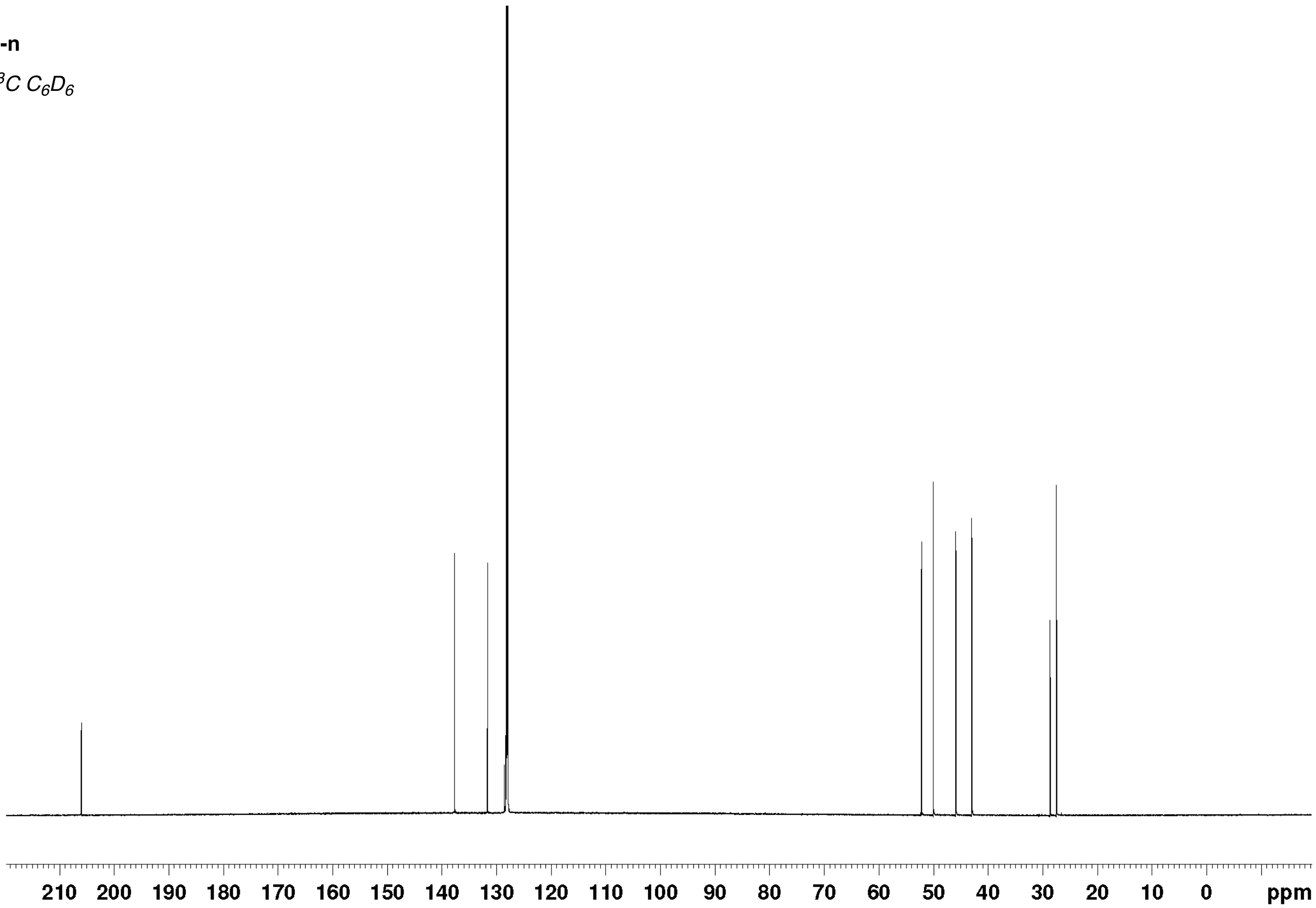

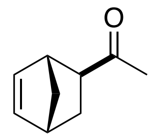

4_{CPD}-x

800Mz ^1H C_6D_6

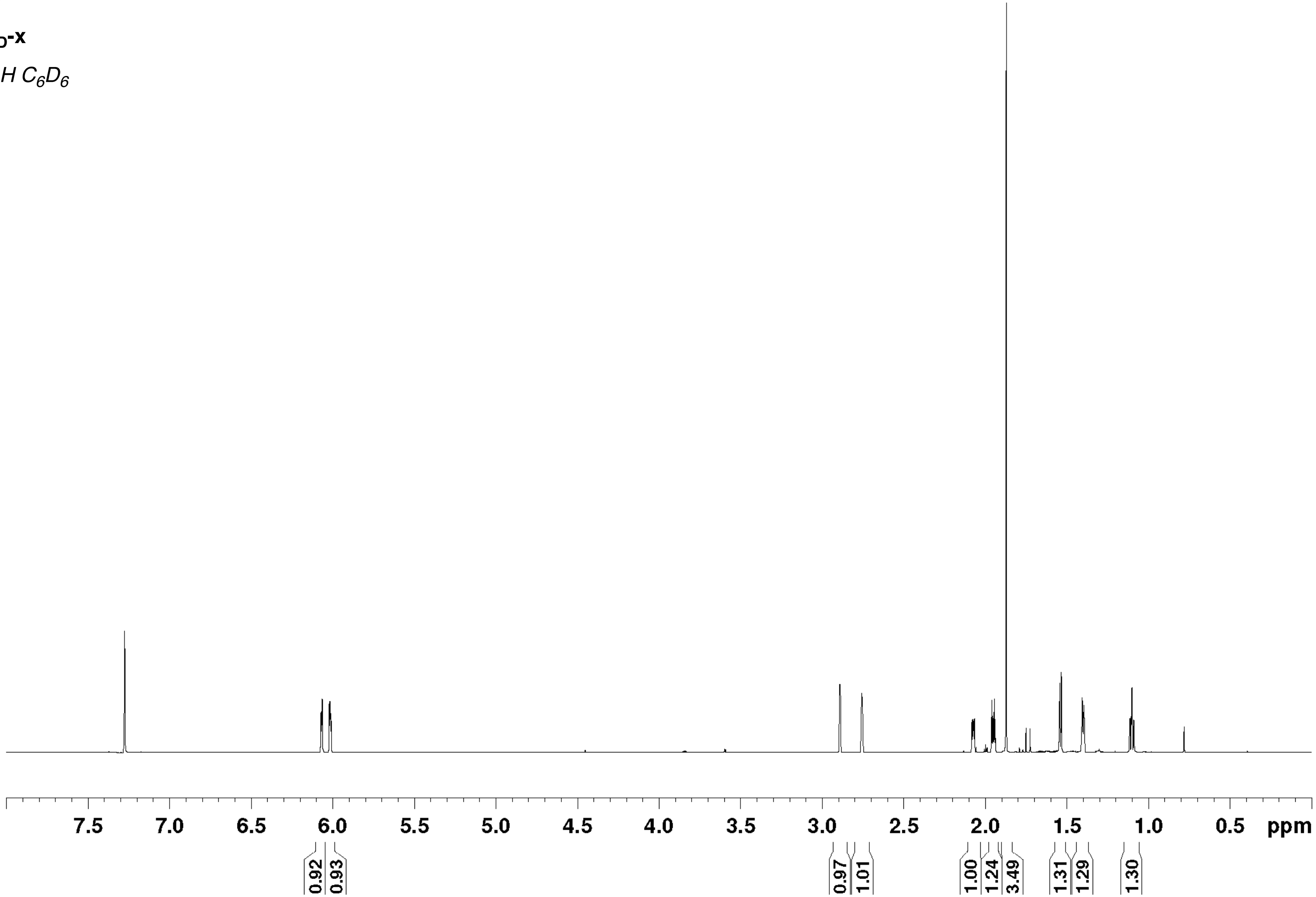

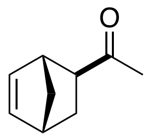

4_{CPD}-x

200Mz ^{13}C C_6D_6

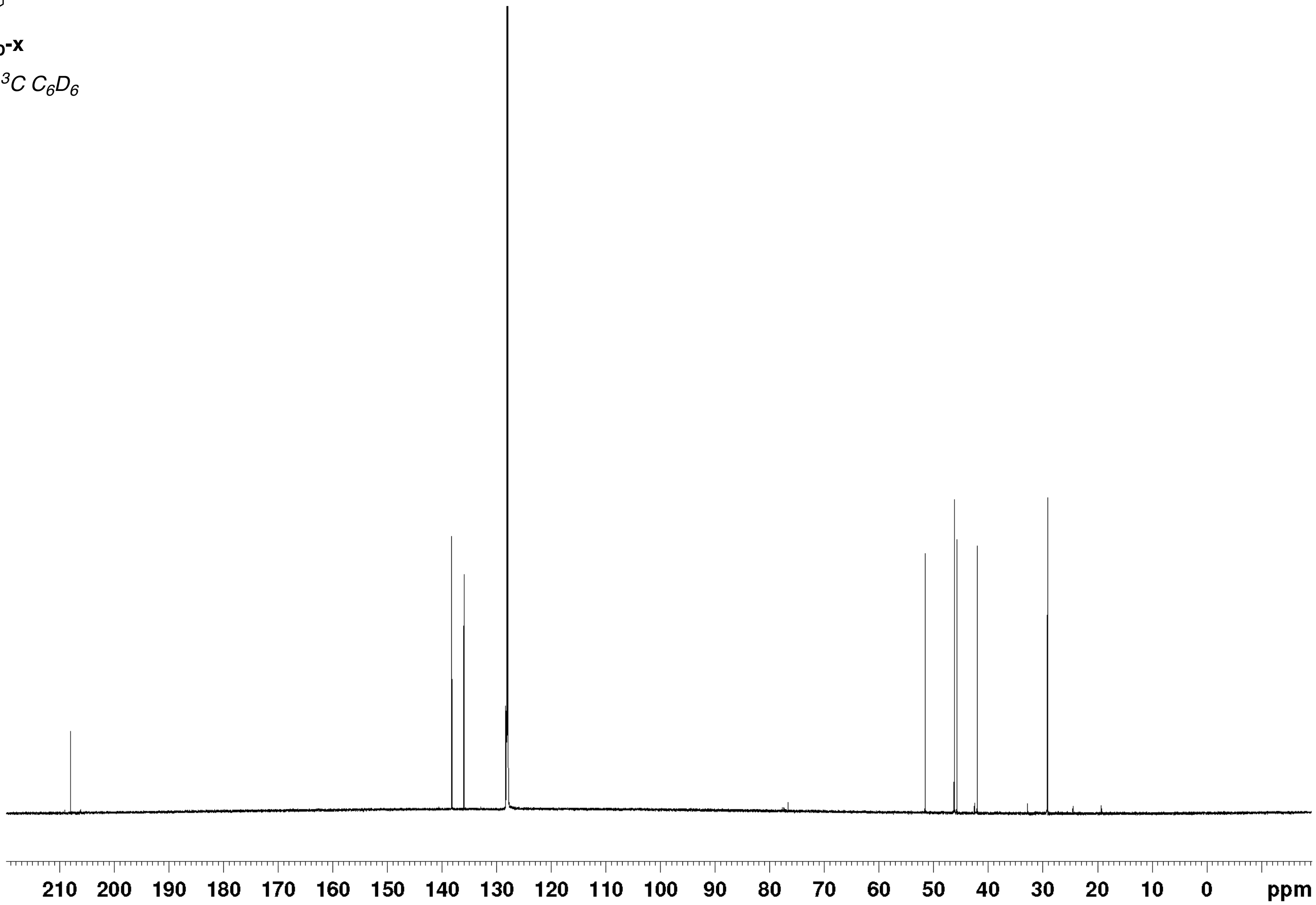

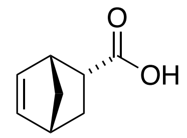

6_{CPD}-n

800Mz ^1H C_6D_6

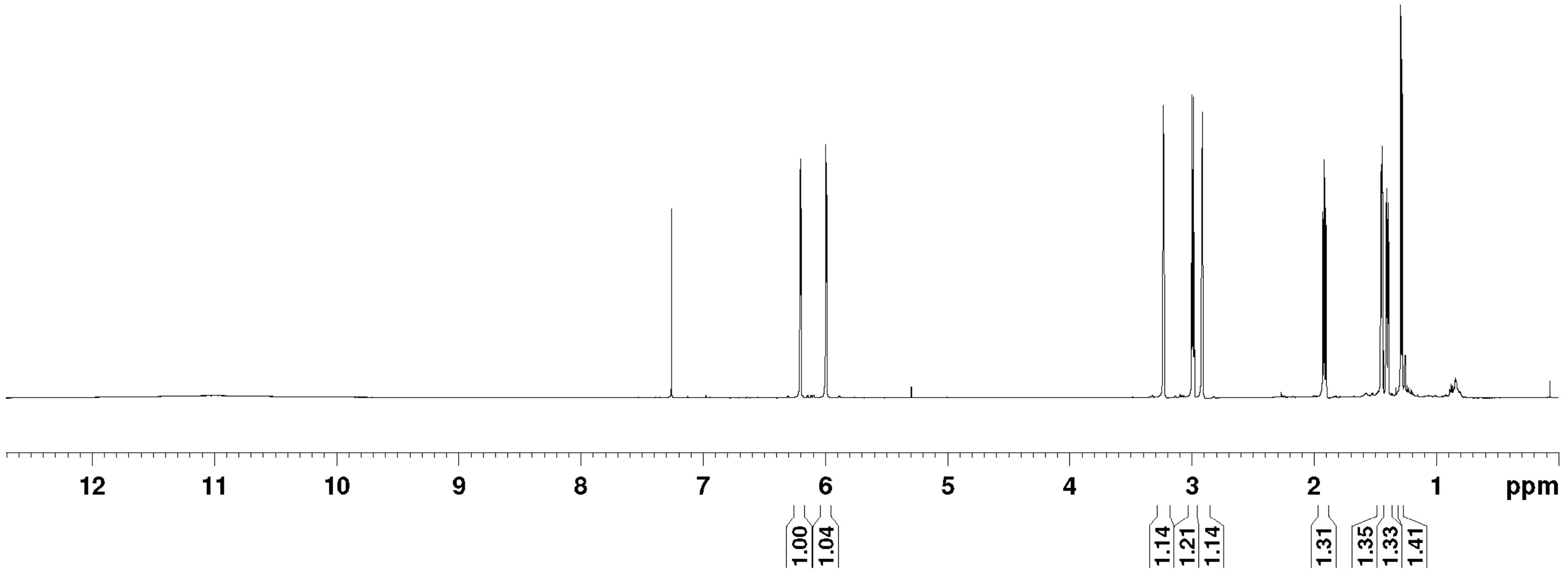

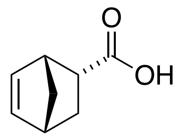

6_{CPD}-n

200Mz ^{13}C C_6D_6

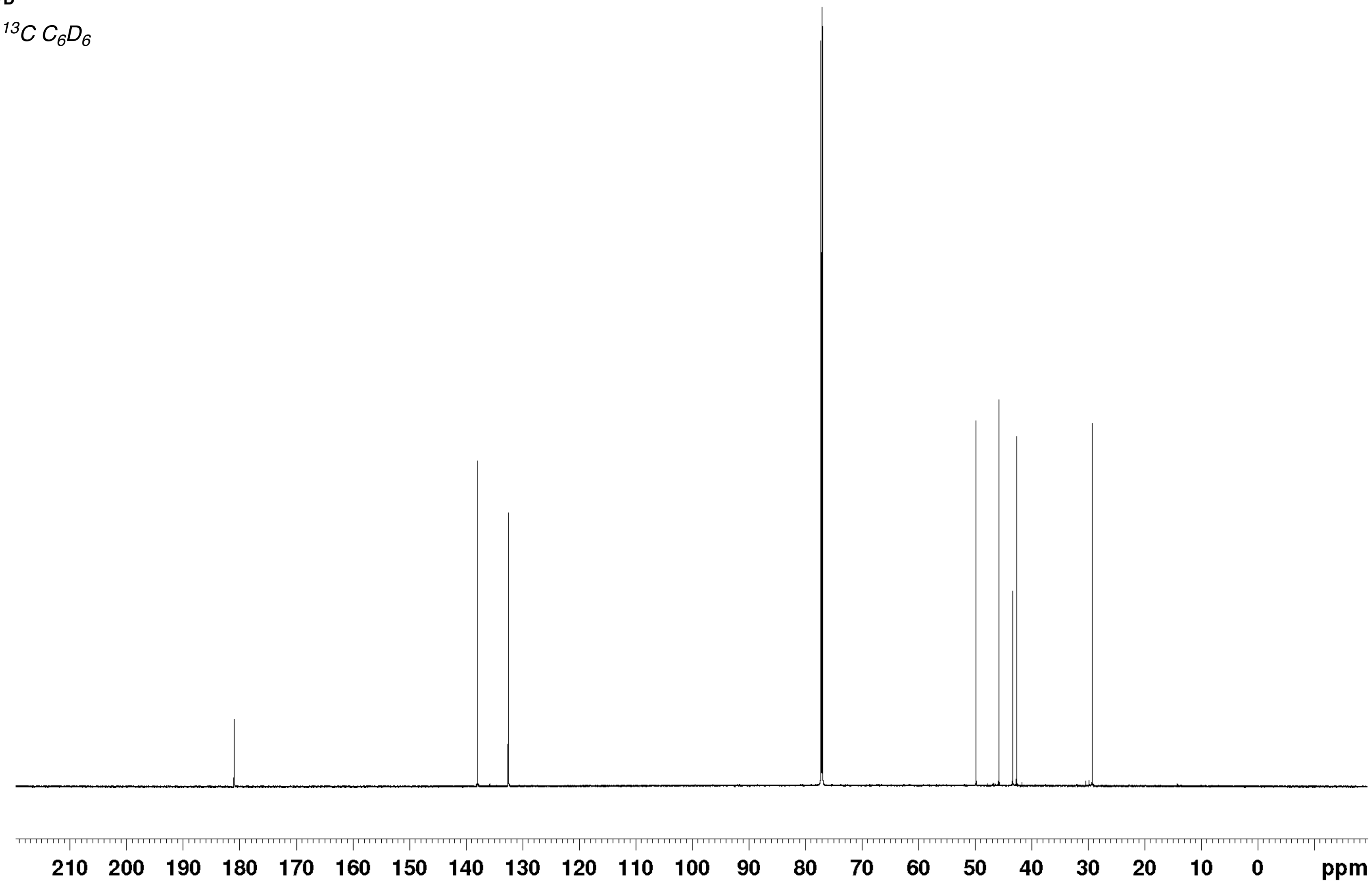

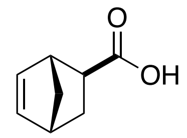

6_{CPD}-x

800Mz [1]*H* C_6D_6

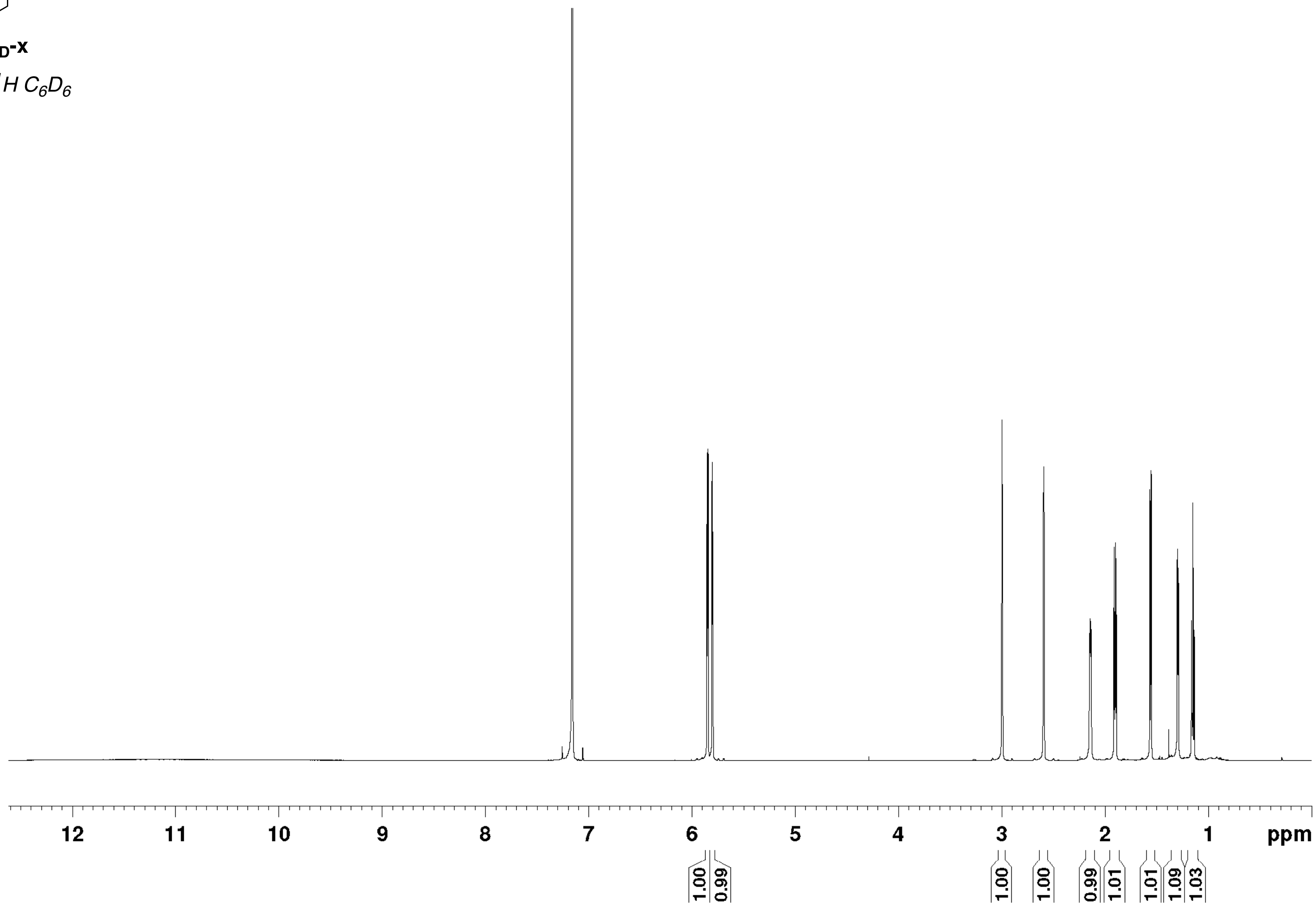

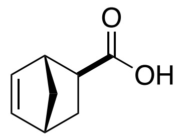

6_{CPD}-x

200Mz ^{13}C C_6D_6

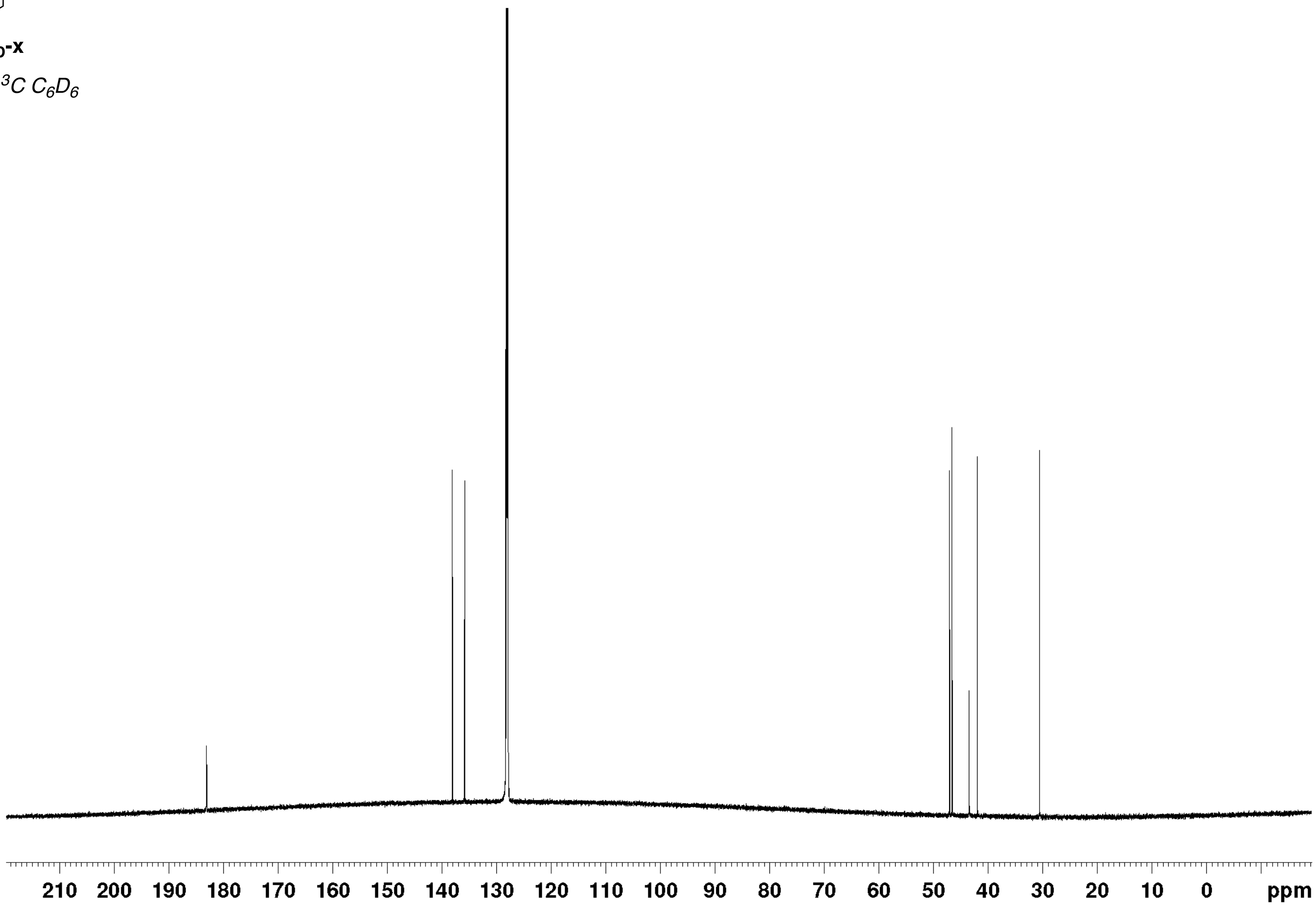

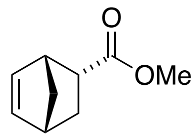

7_{CPD}-n

800Mz ^{1}H C_6D_6

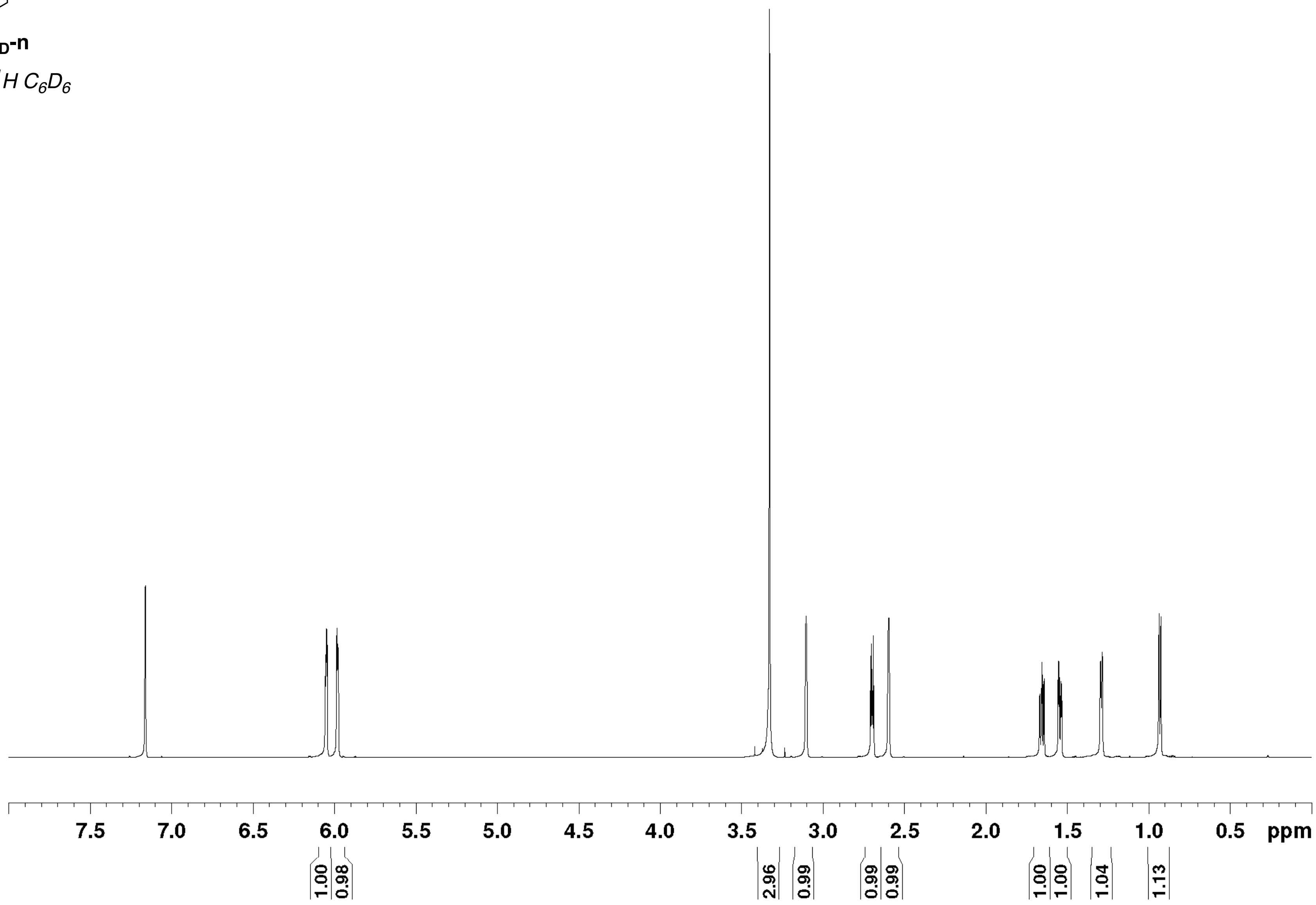

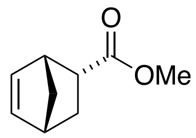

7_{CPD}-n

200Mz ^{13}C C_6D_6

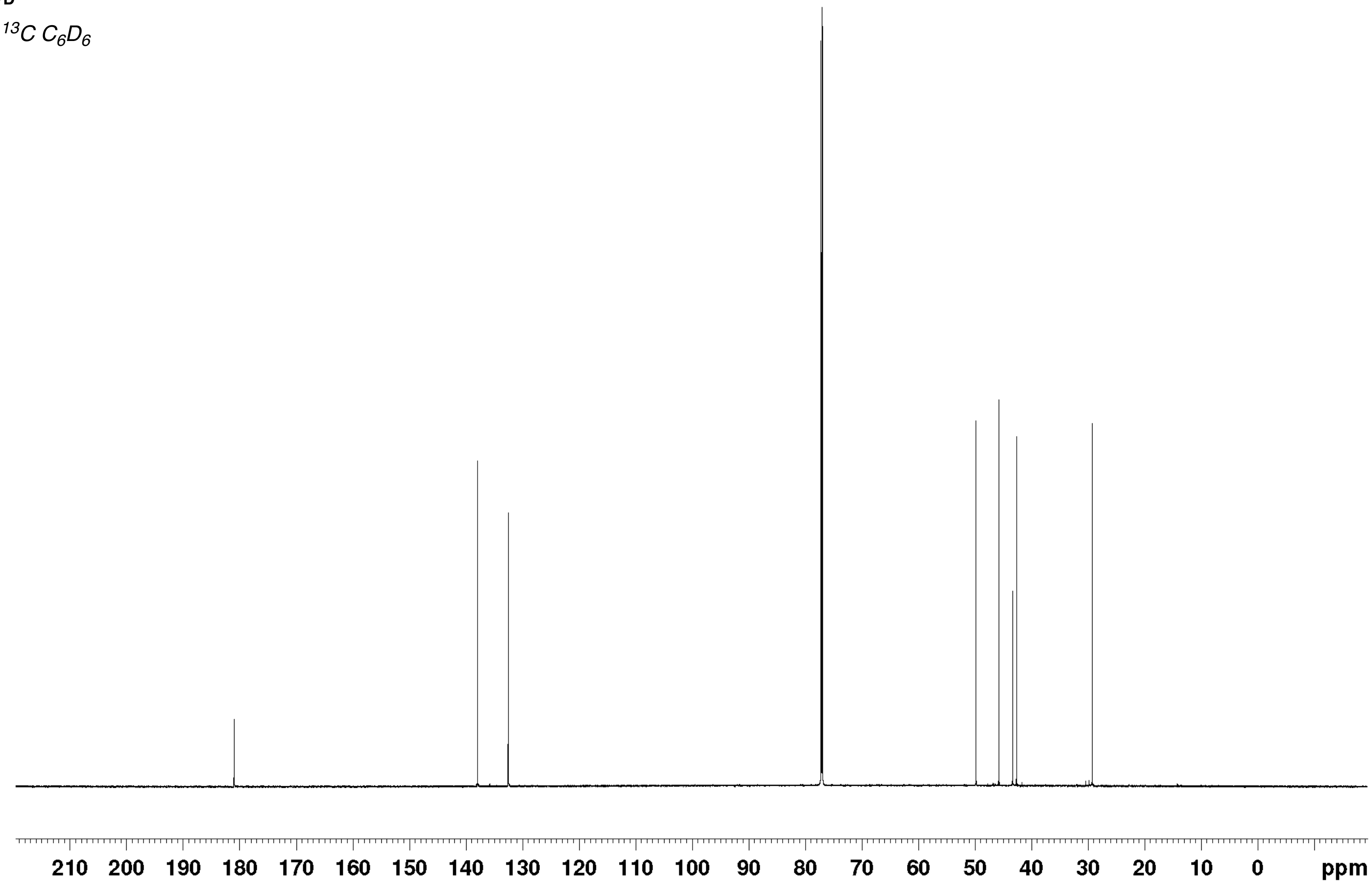

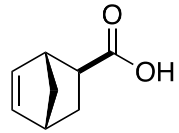

7_{CPD}-x

800Mz ^1H C_6D_6

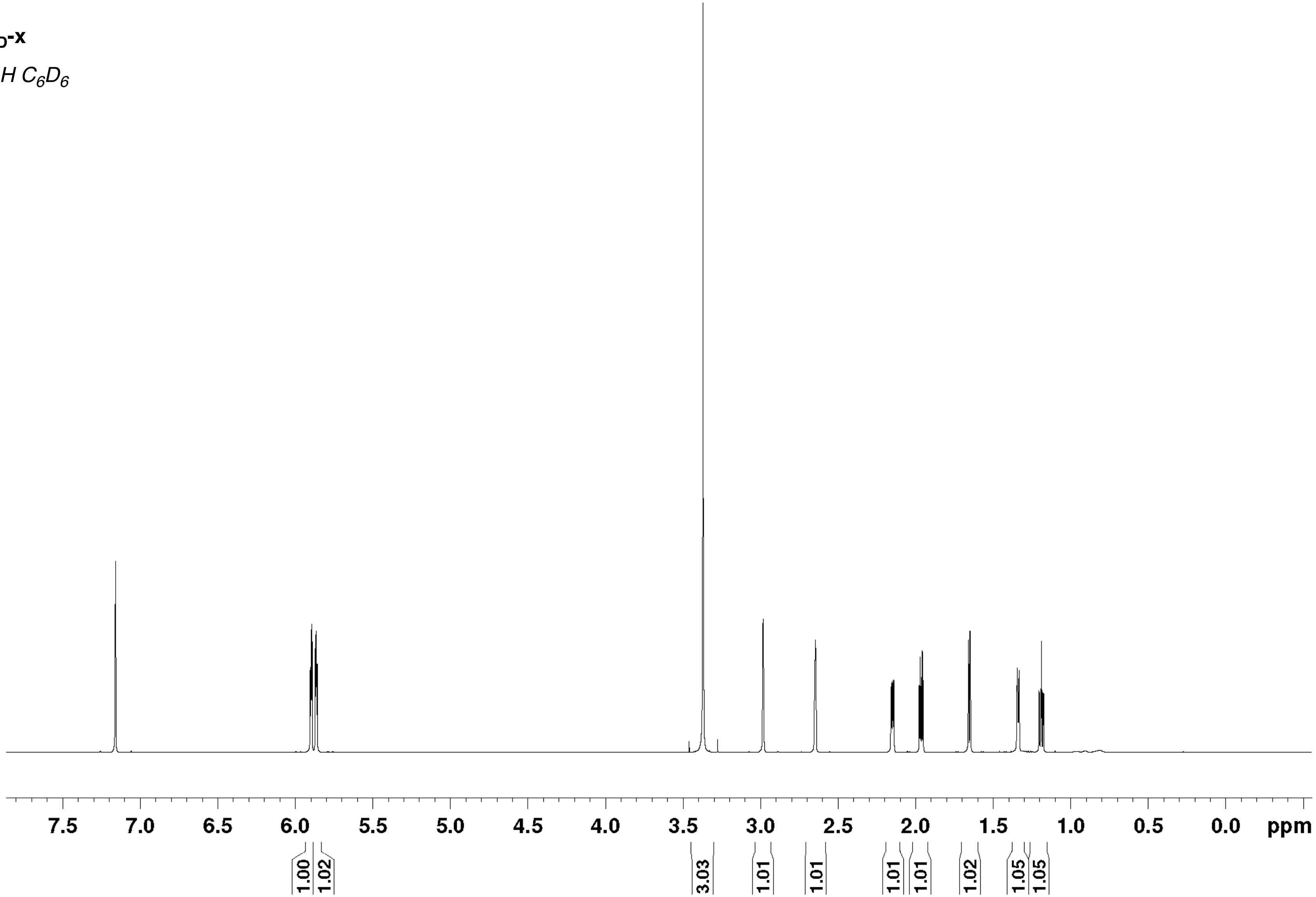

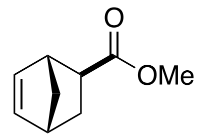

$\mathbf{7_{CPD}}$**-x**

200Mz ^{13}C C_6D_6

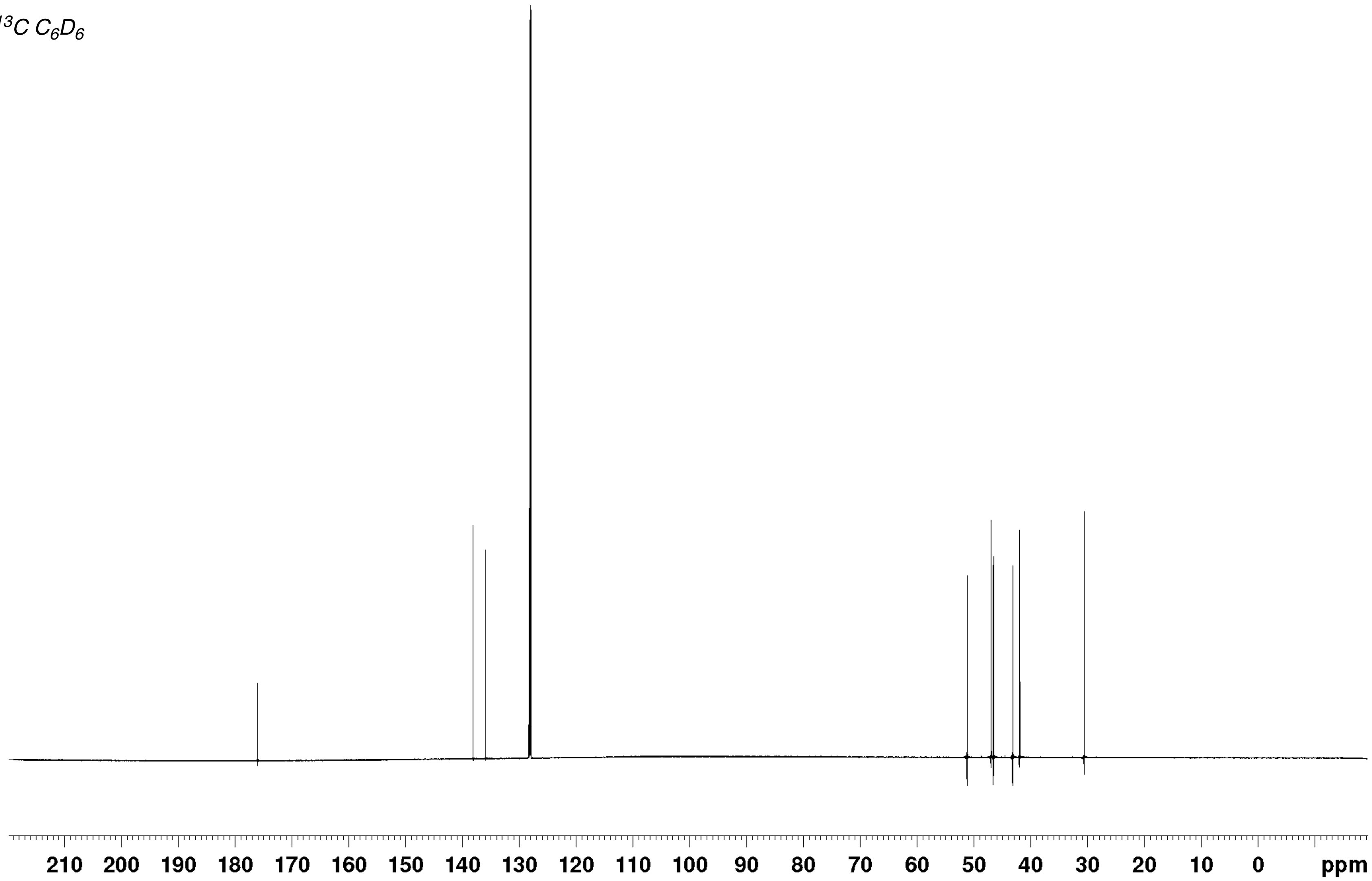

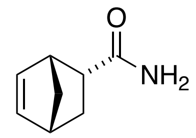

8_{CPD}-n

800Mz ^{1}H C_6D_6

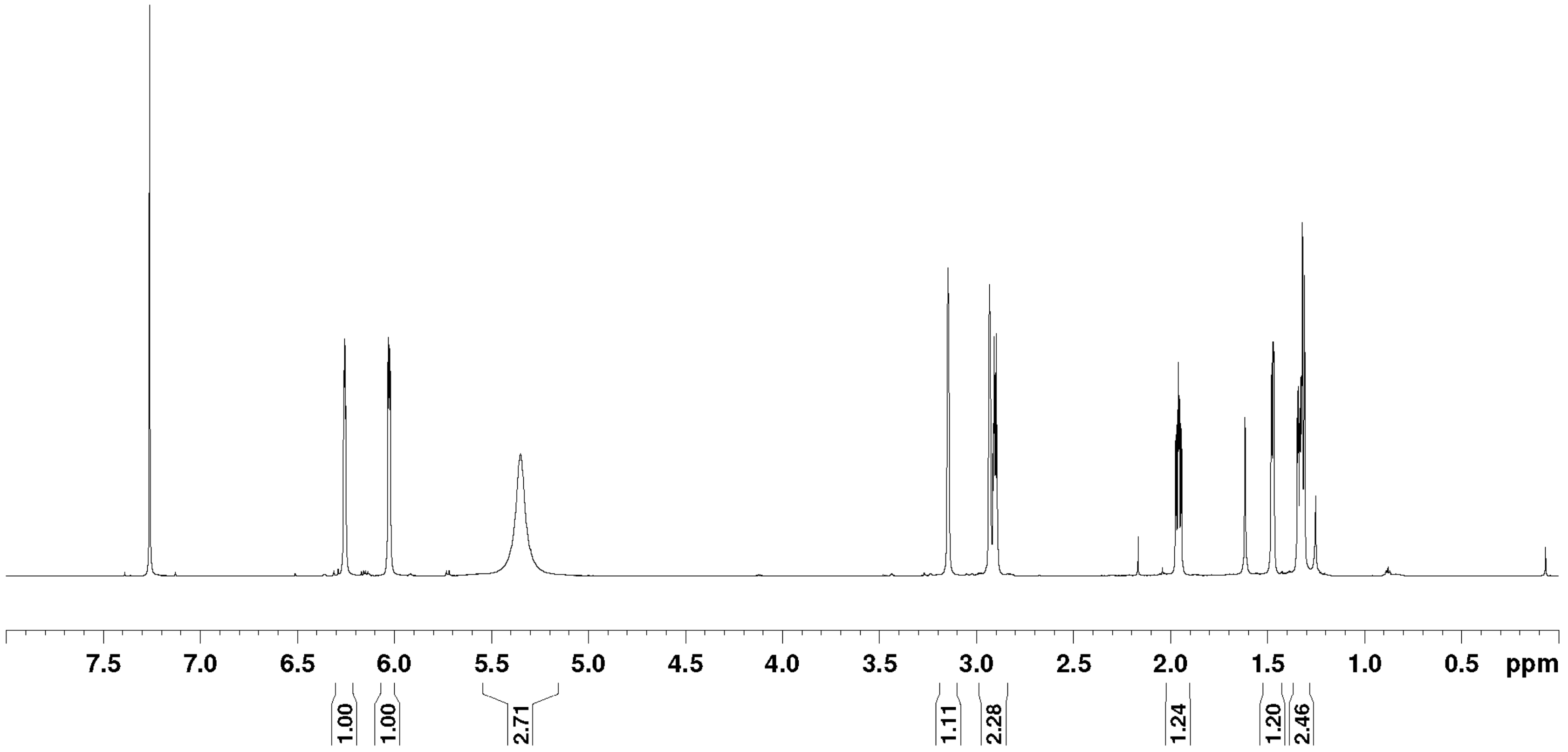

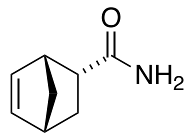

8$_{CPD}$-n

200Mz ^{13}C C_6D_6

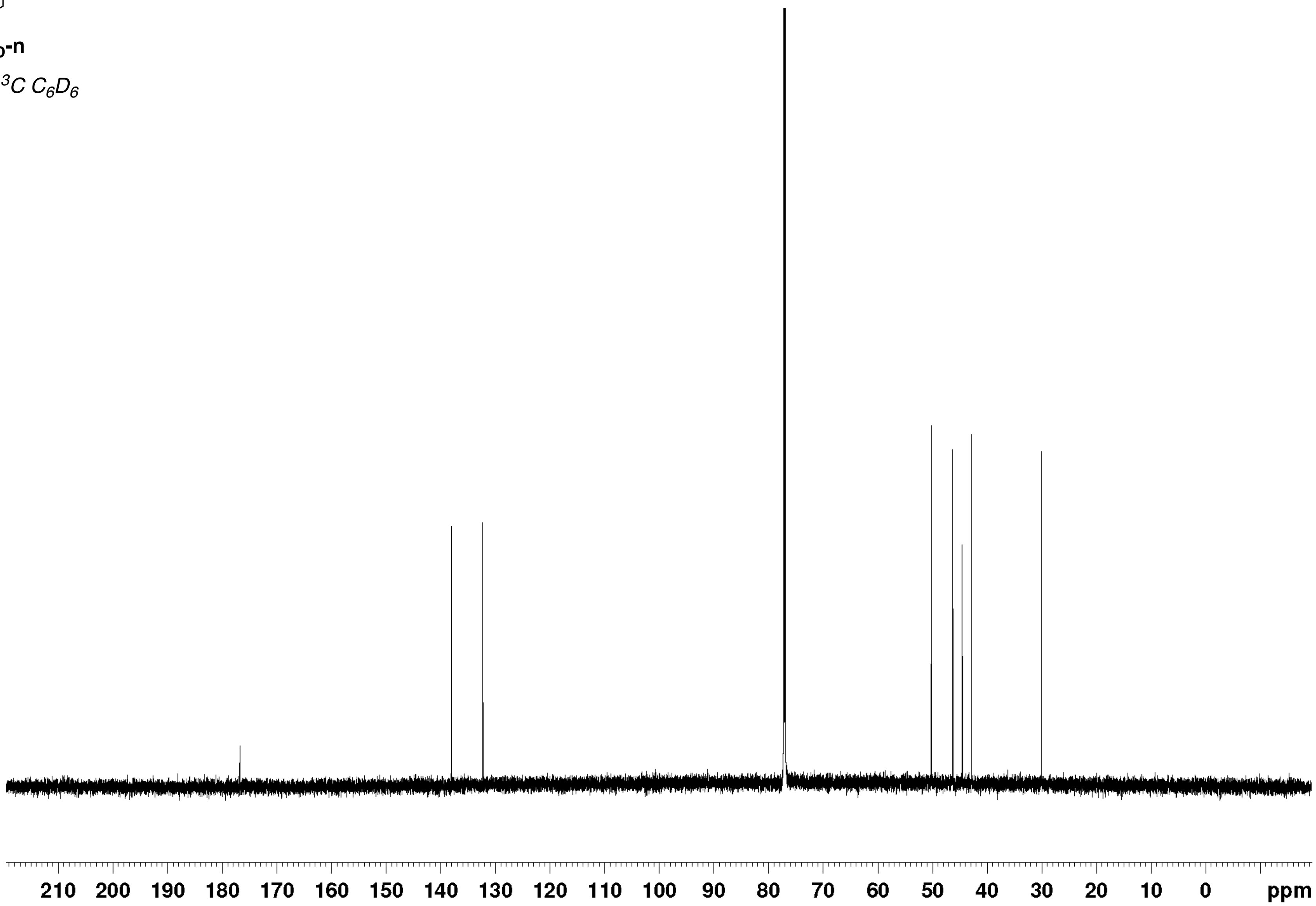

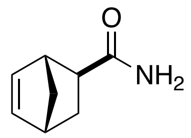

8$_{CPD}$-x

800Mz ^1H C_6D_6

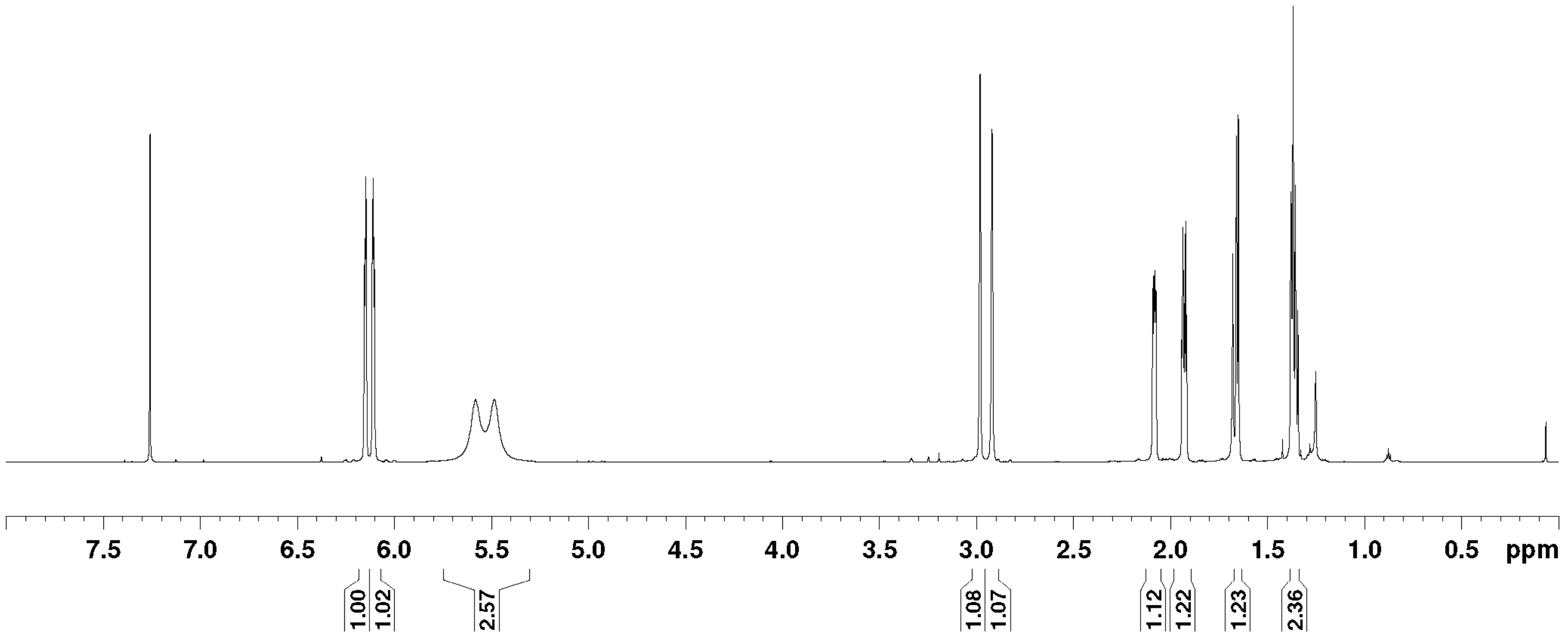

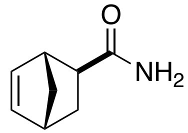

8_{CPD}-x

200Mz ^{13}C C_6D_6

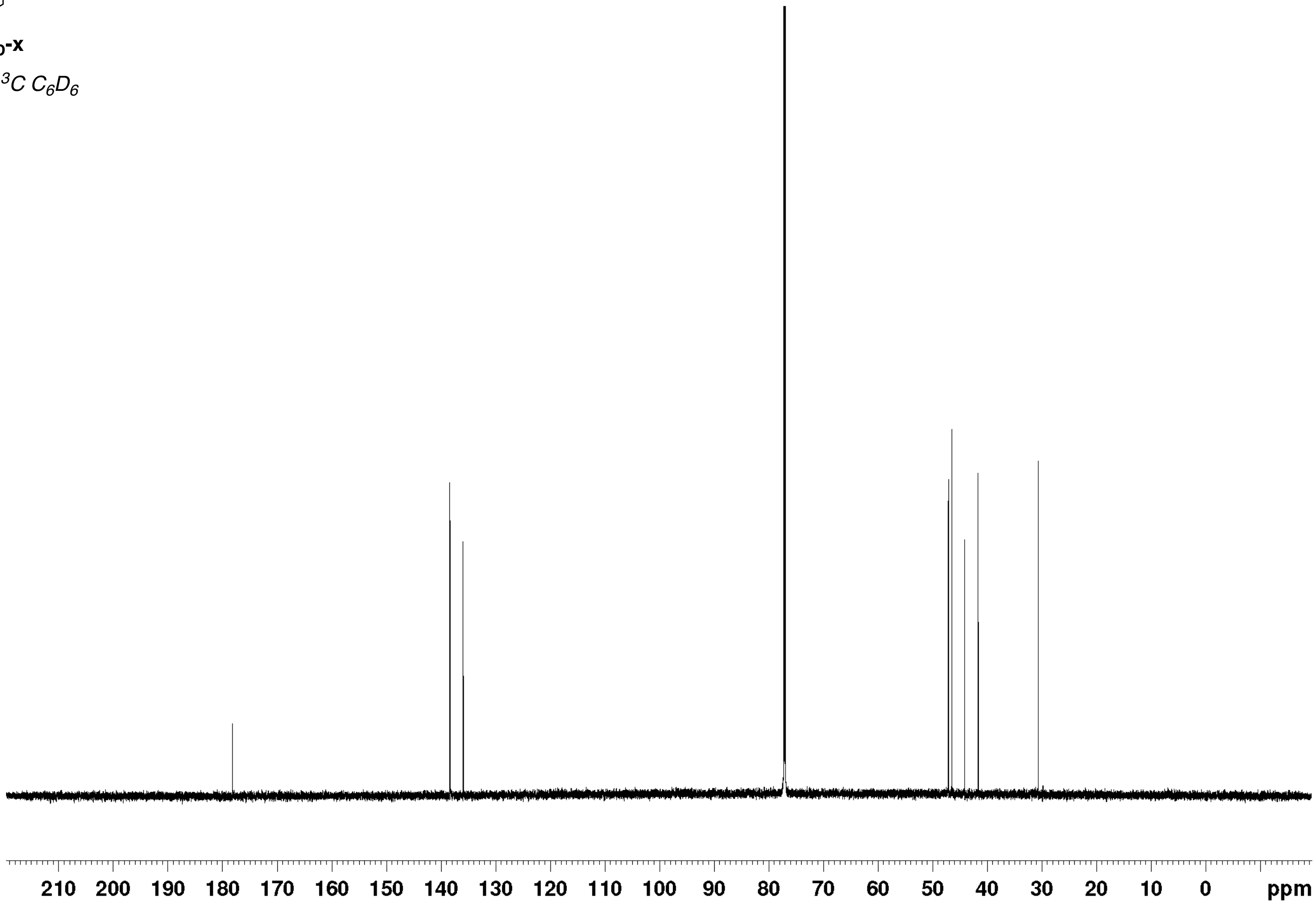

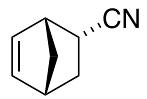

9_{CPD}-n

800Mz ^{1}H C_6D_6

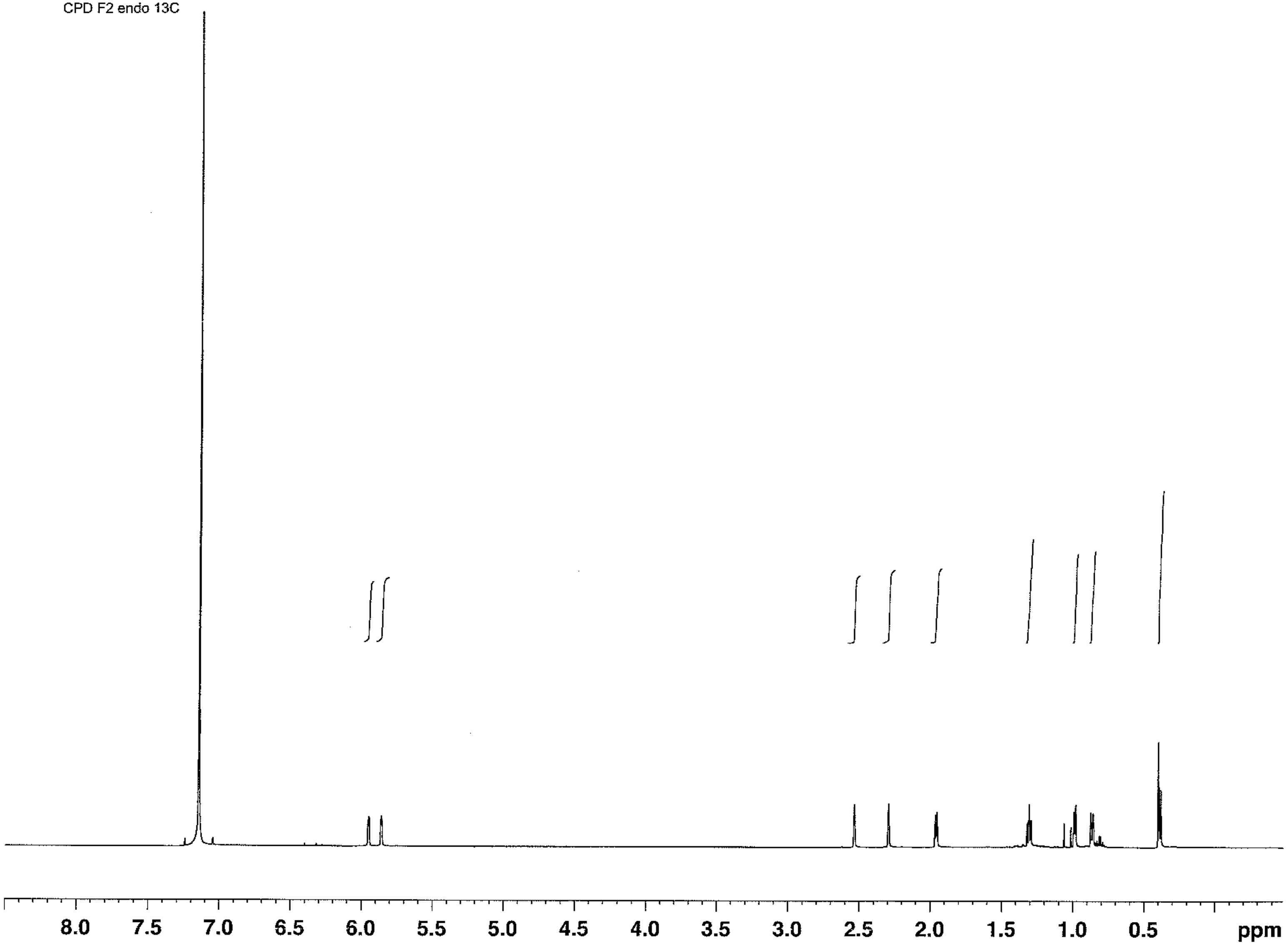

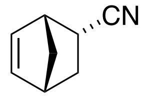

9_{CPD}-n

200Mz ^{13}C C_6D_6

CPD F2 endo 13C

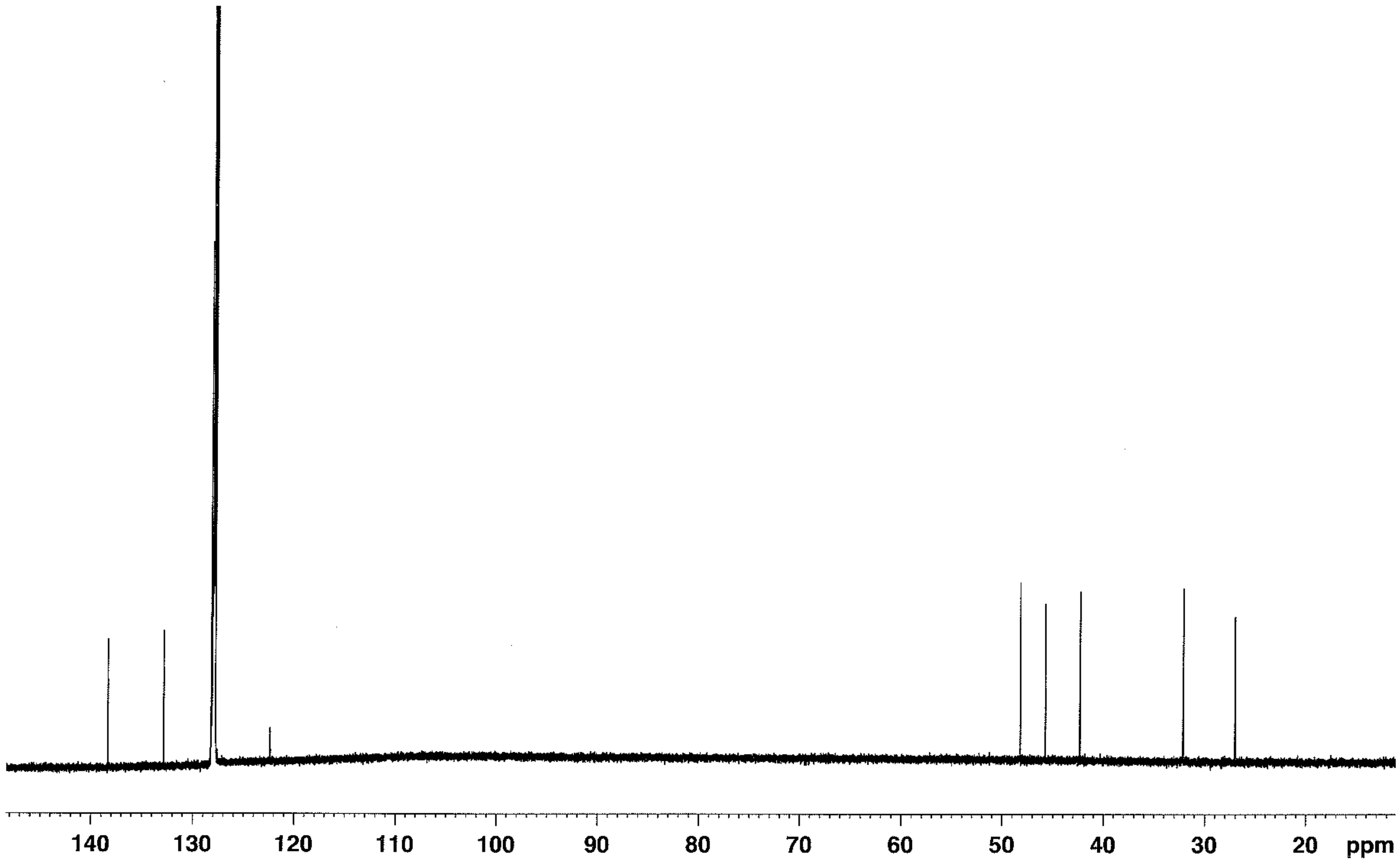

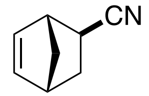

9_{CPD}-x

800Mz ^{1}H C_6D_6

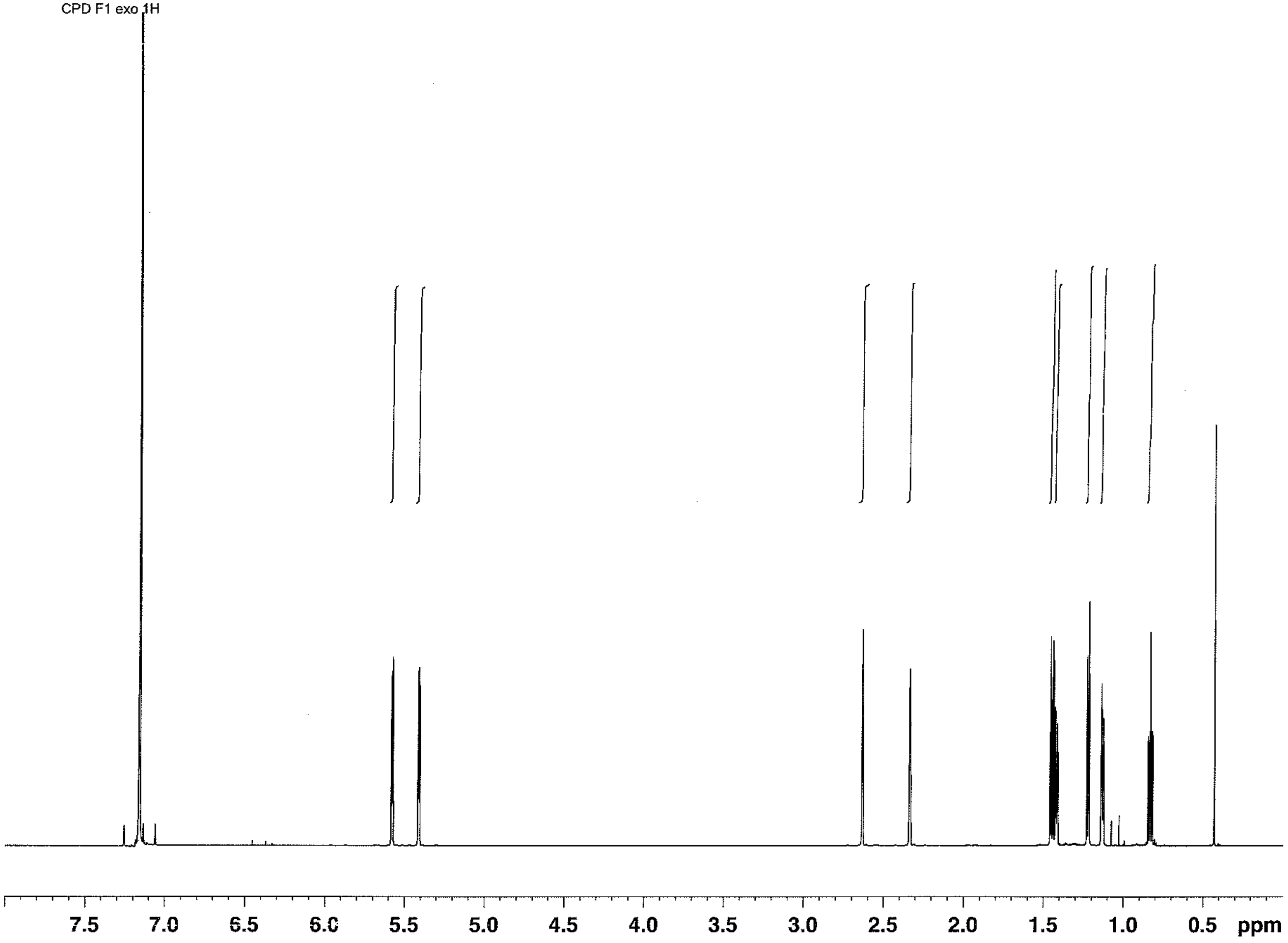

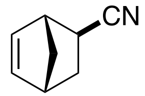

9_{CPD}-x

200Mz ^{13}C C_6D_6

CPD F1 13C

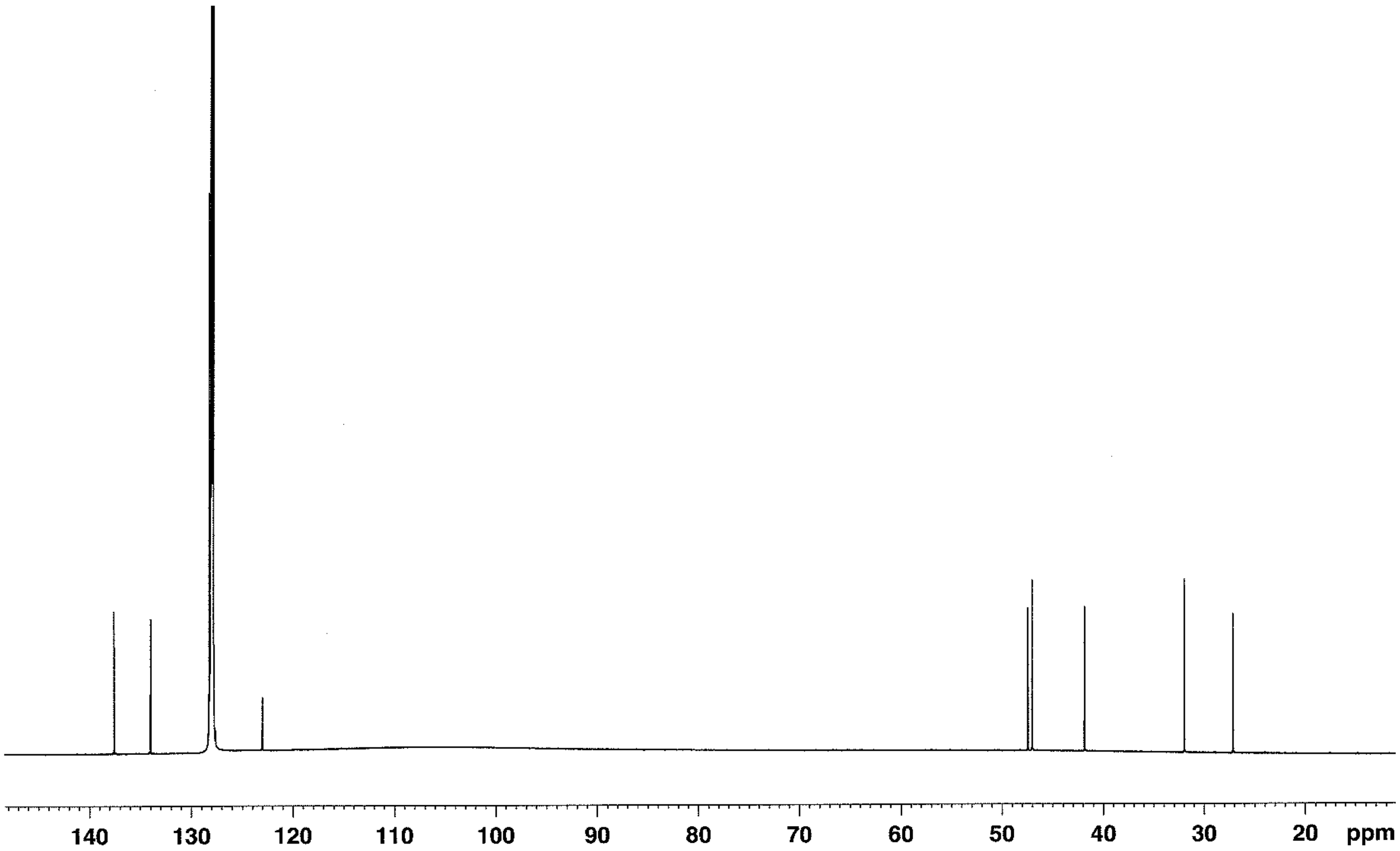

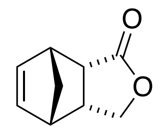

10_{CPD}-n

800Mz ^1H C_6D_6

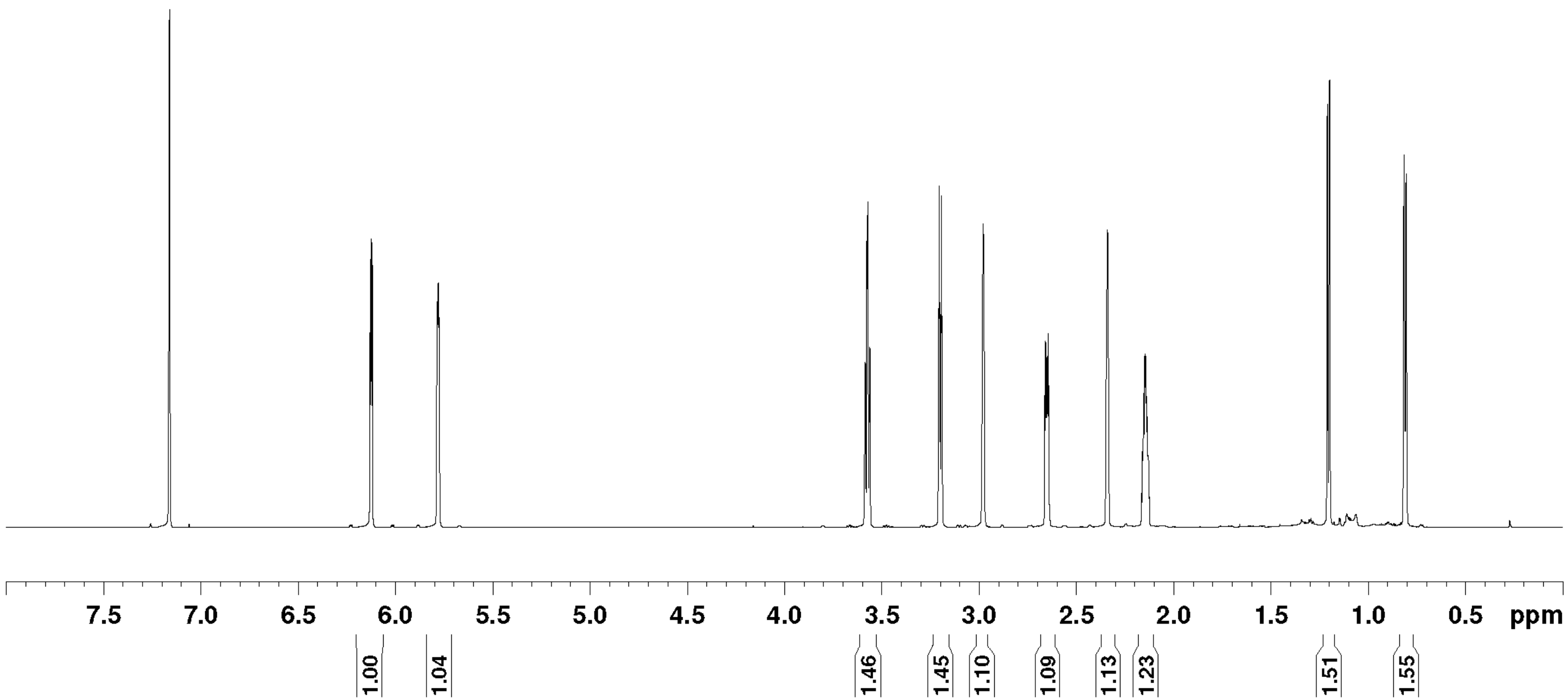

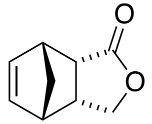

10_{CPD}-n

200Mz ^{13}C C_6D_6

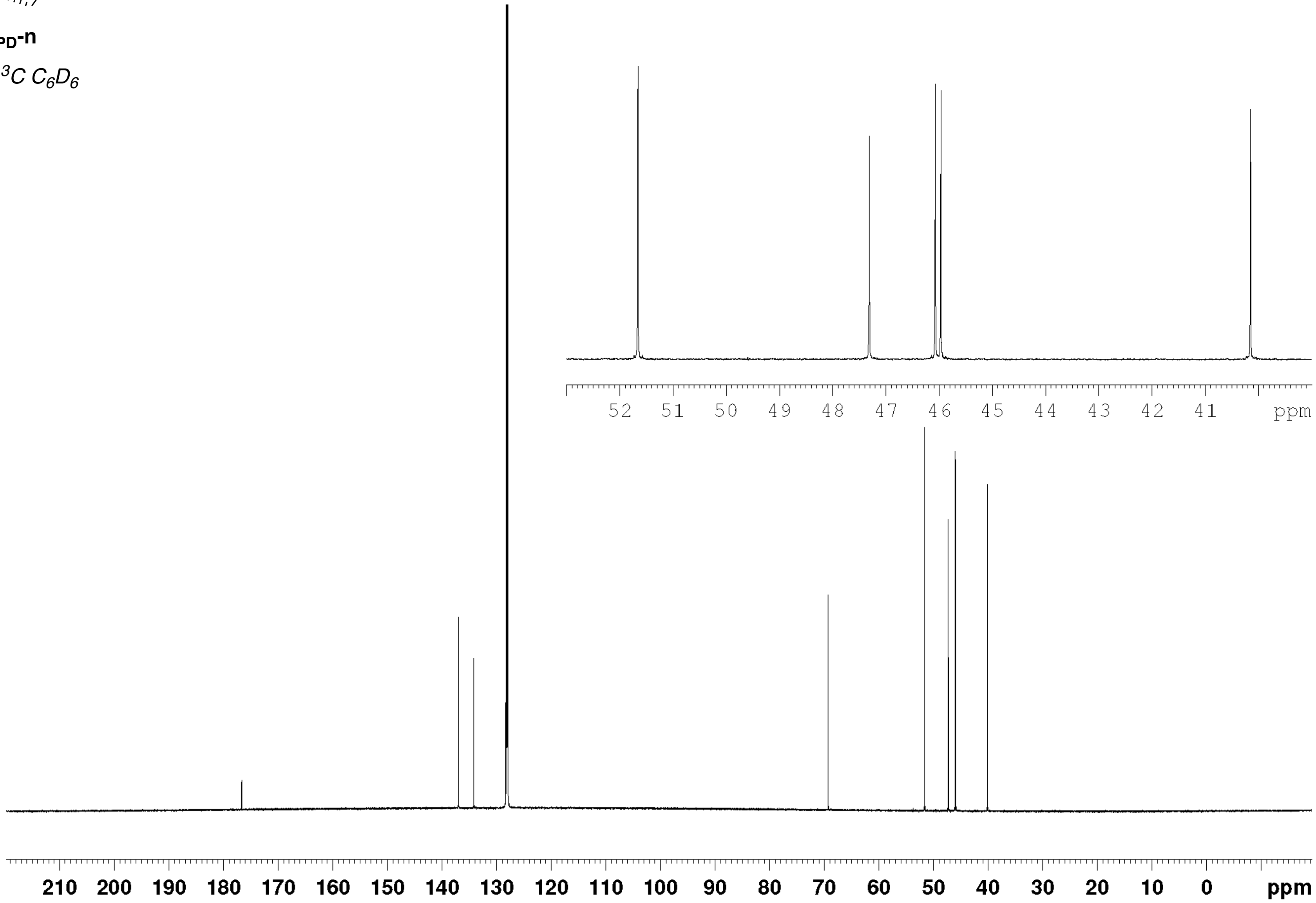

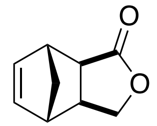

10_{CPD}-x

800Mz ^{1}H C_6D_6

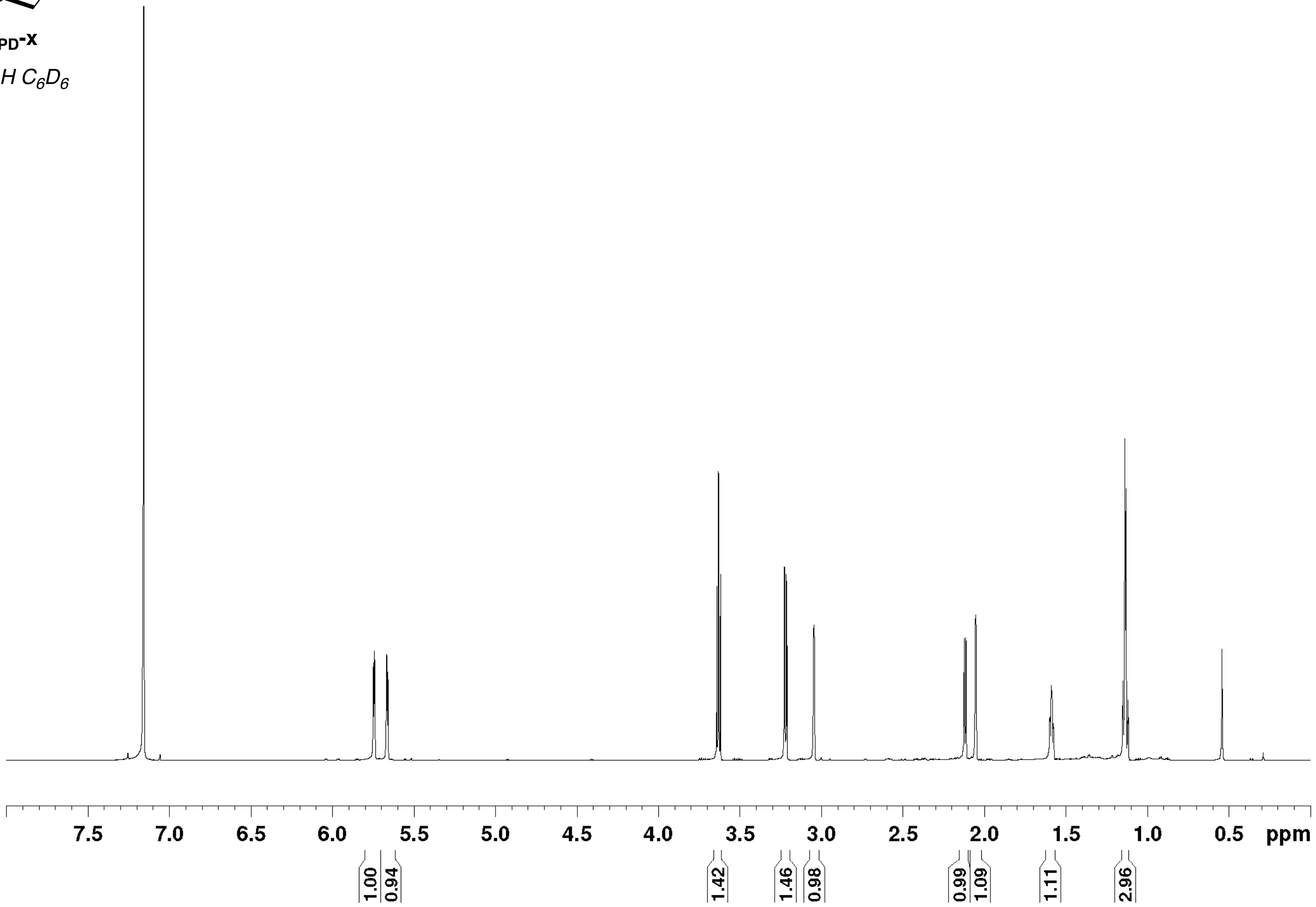

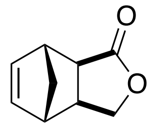

10_{CPD}-x

200Mz ^{13}C C_6D_6

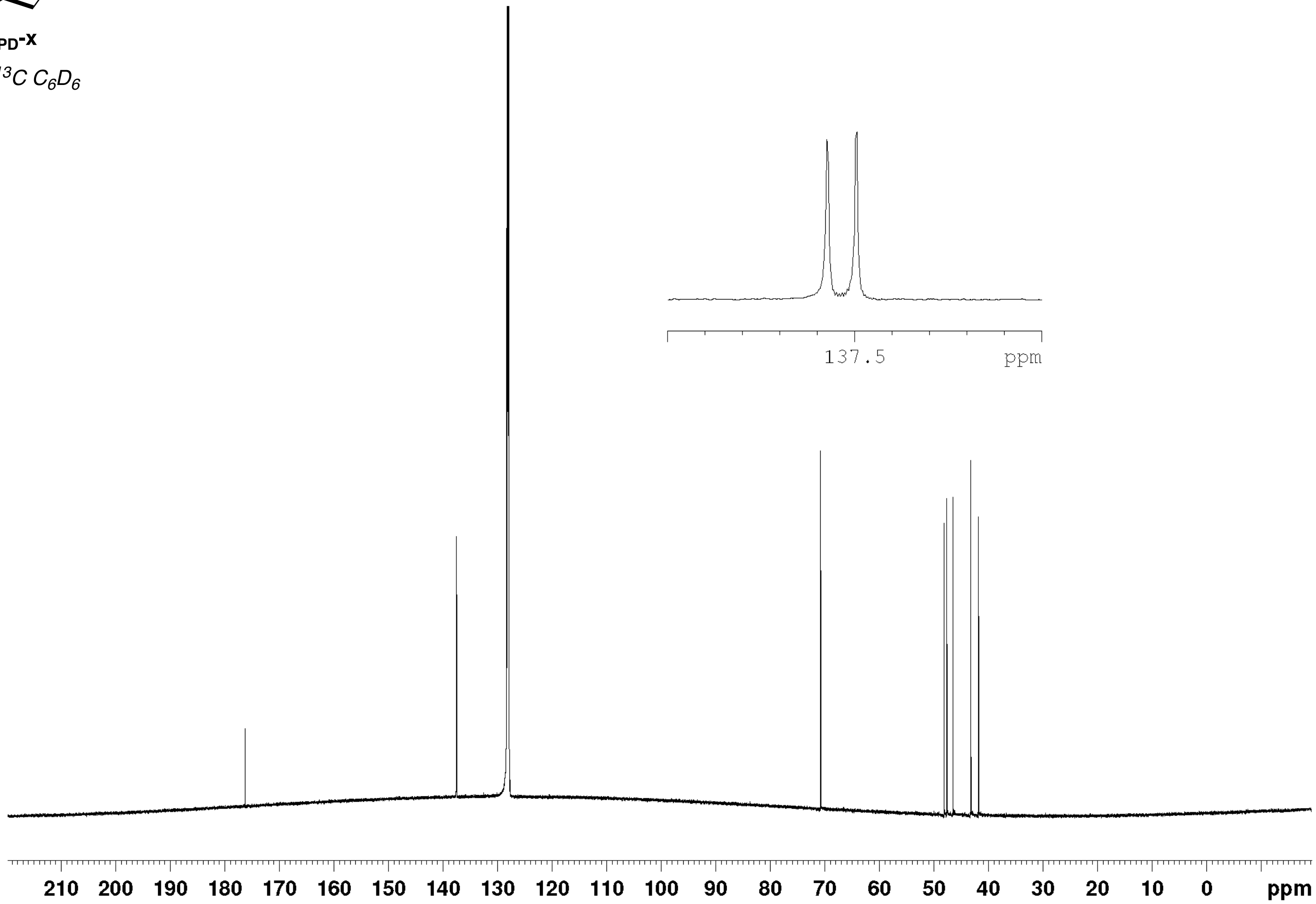

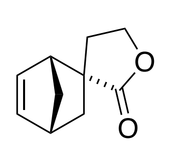

11_{CPD}-n

800Mz ^1H C_6D_6

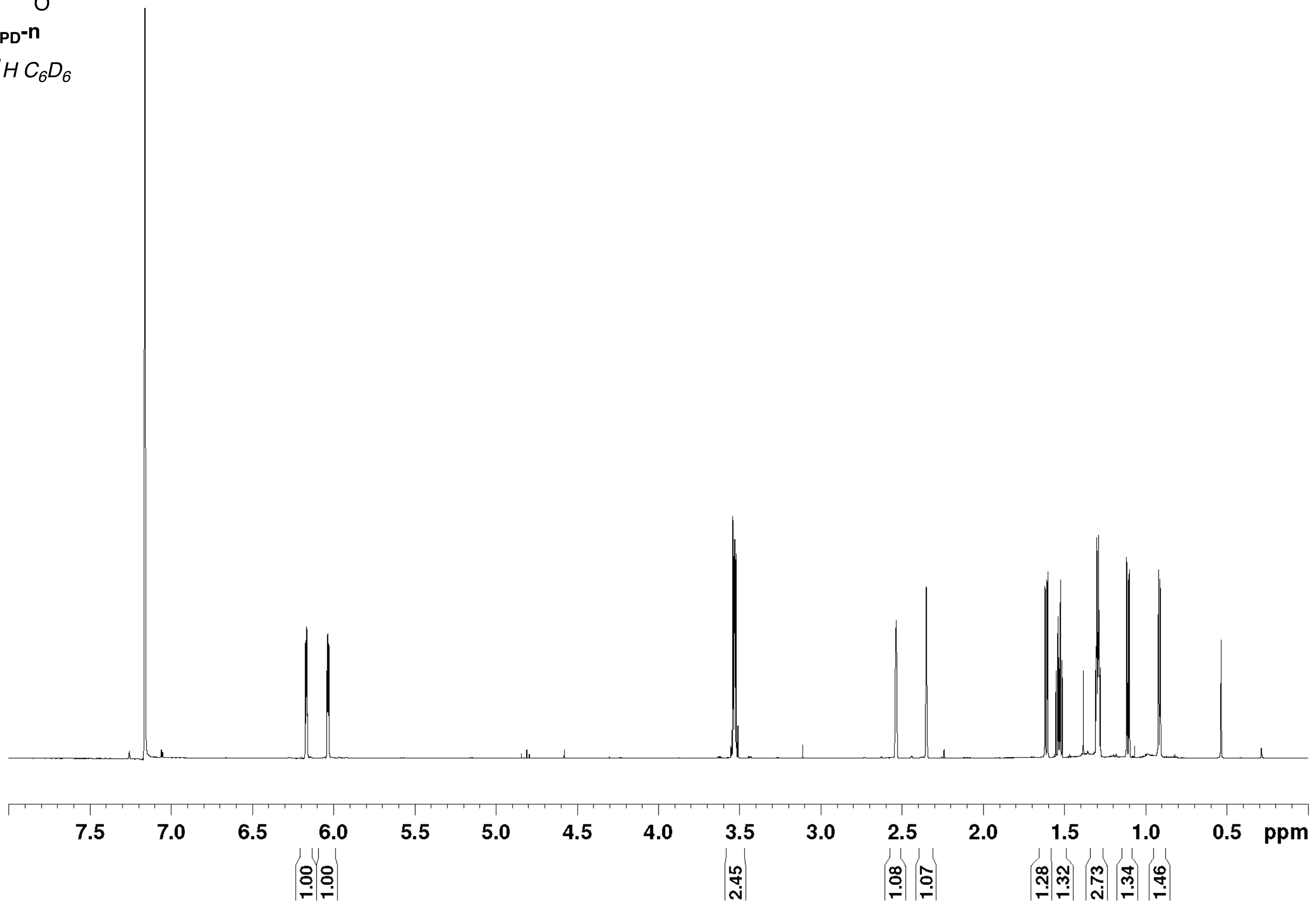

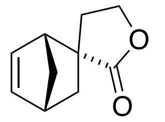

11_{CPD}-n

200Mz ^{13}C C_6D_6

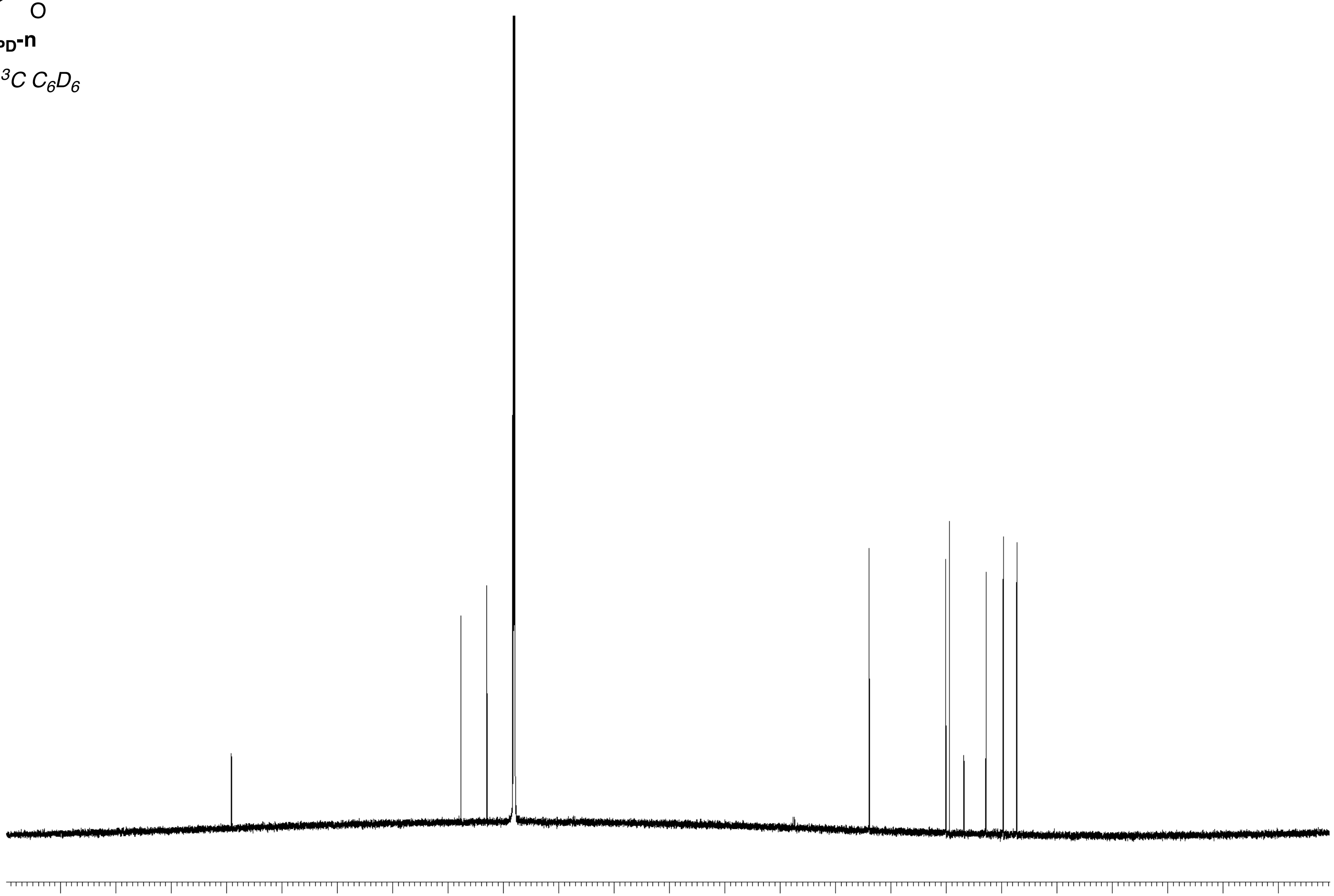

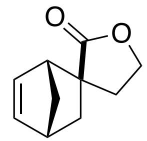

11_{CPD}-x

800Mz ^1H C_6D_6

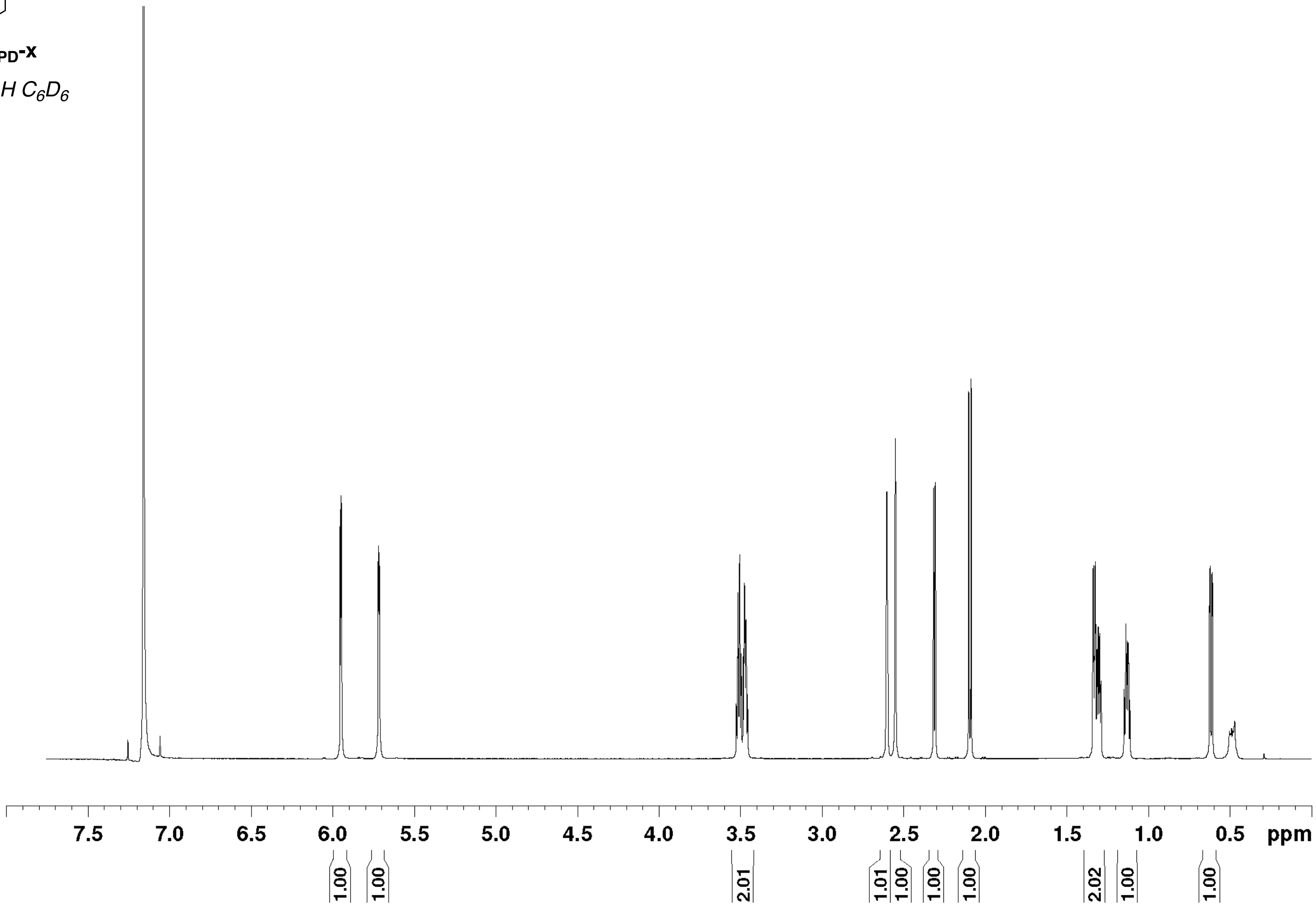

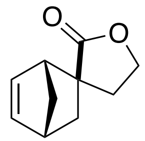

11_{CPD}-x

200Mz ^{13}C C_6D_6

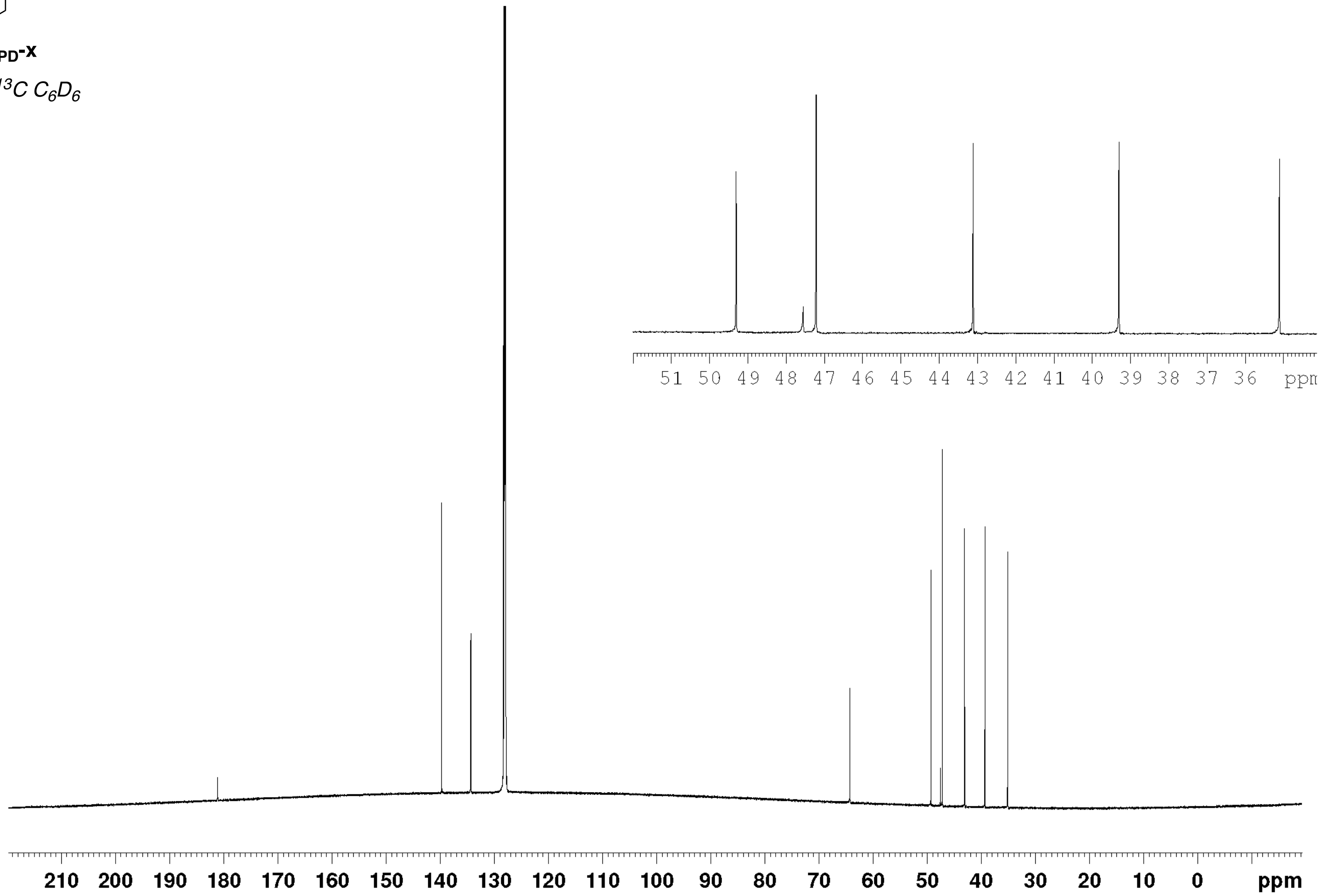

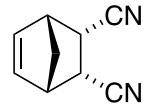

14$_{CPD}$-n

800Mz ^1H C_6D_6

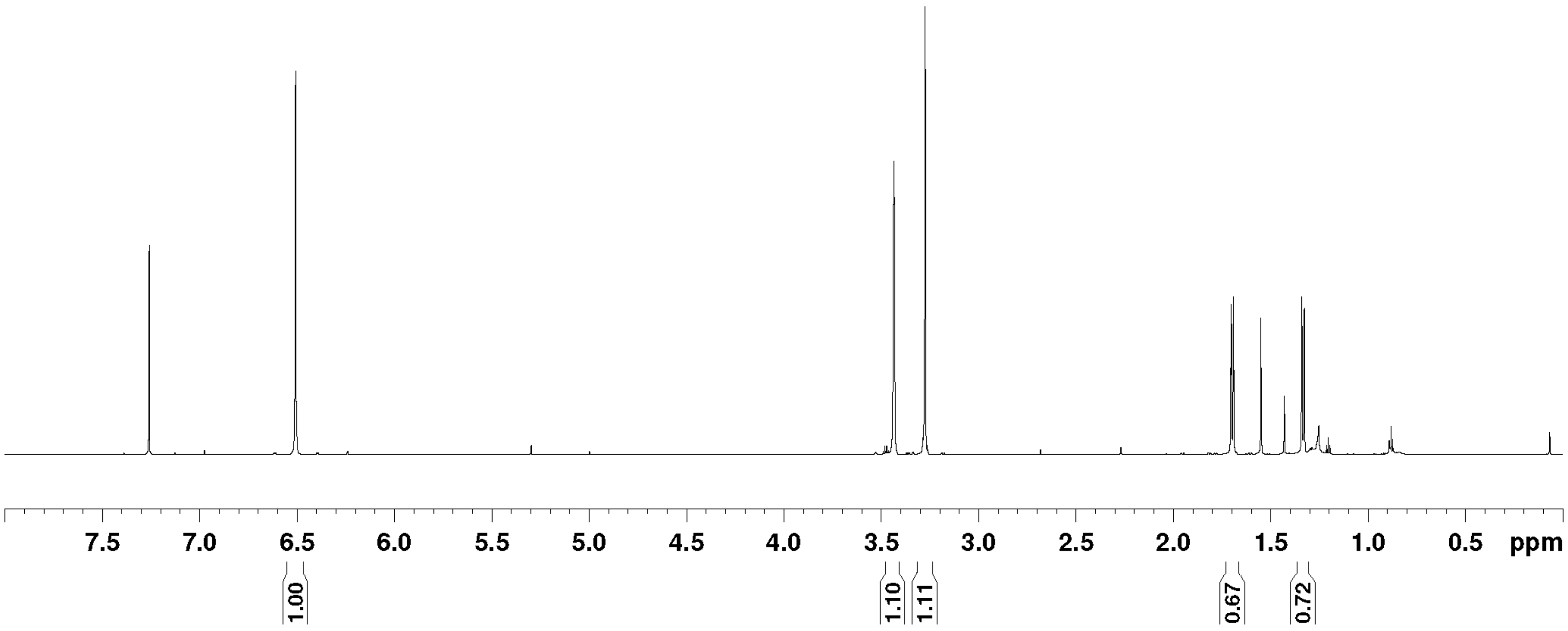

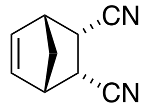

14_{CPD}-n

200Mz ^{13}C C_6D_6

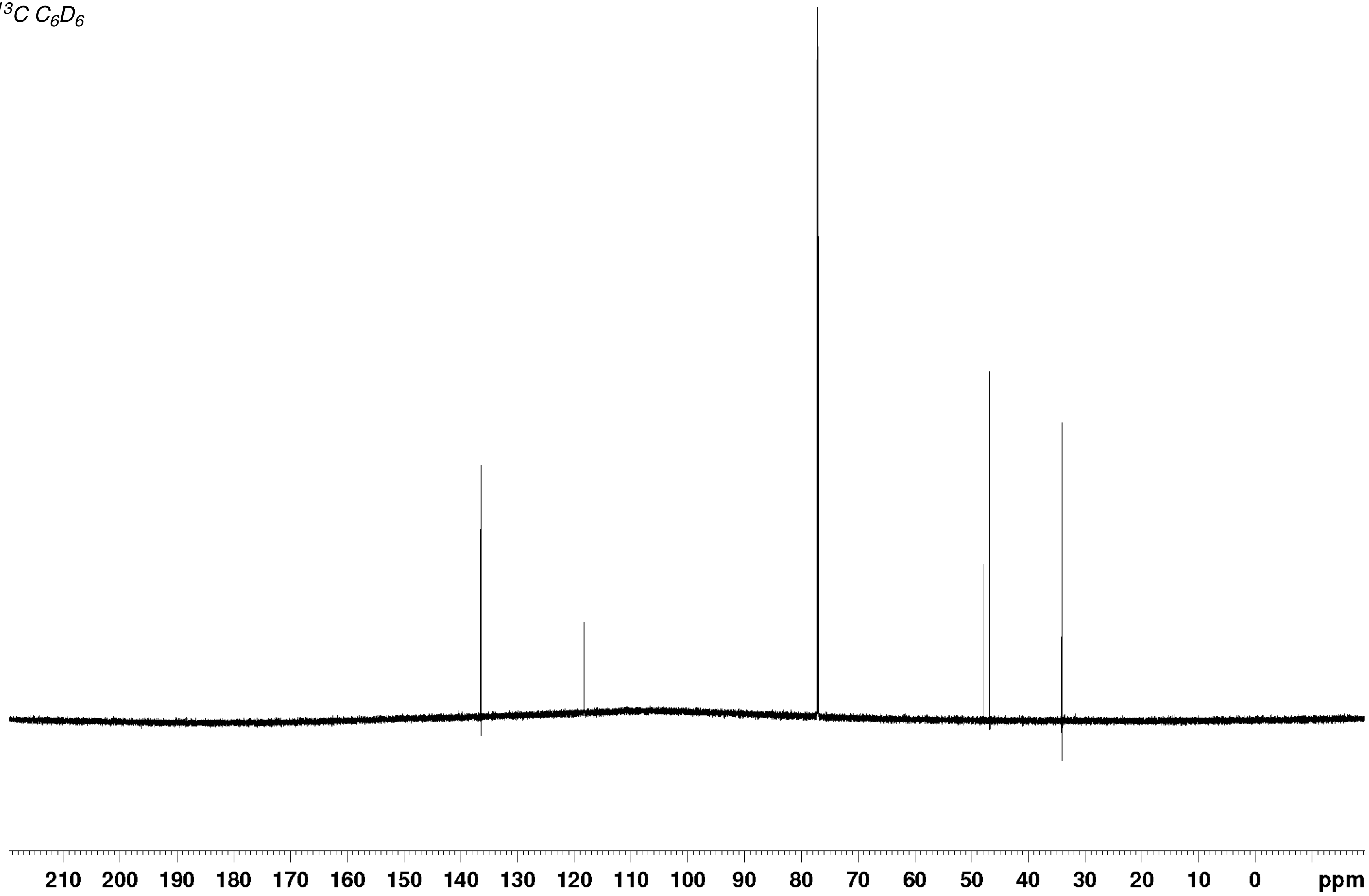

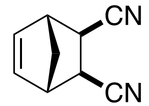

14_{CPD}-x

800Mz ^{1}H C_6D_6

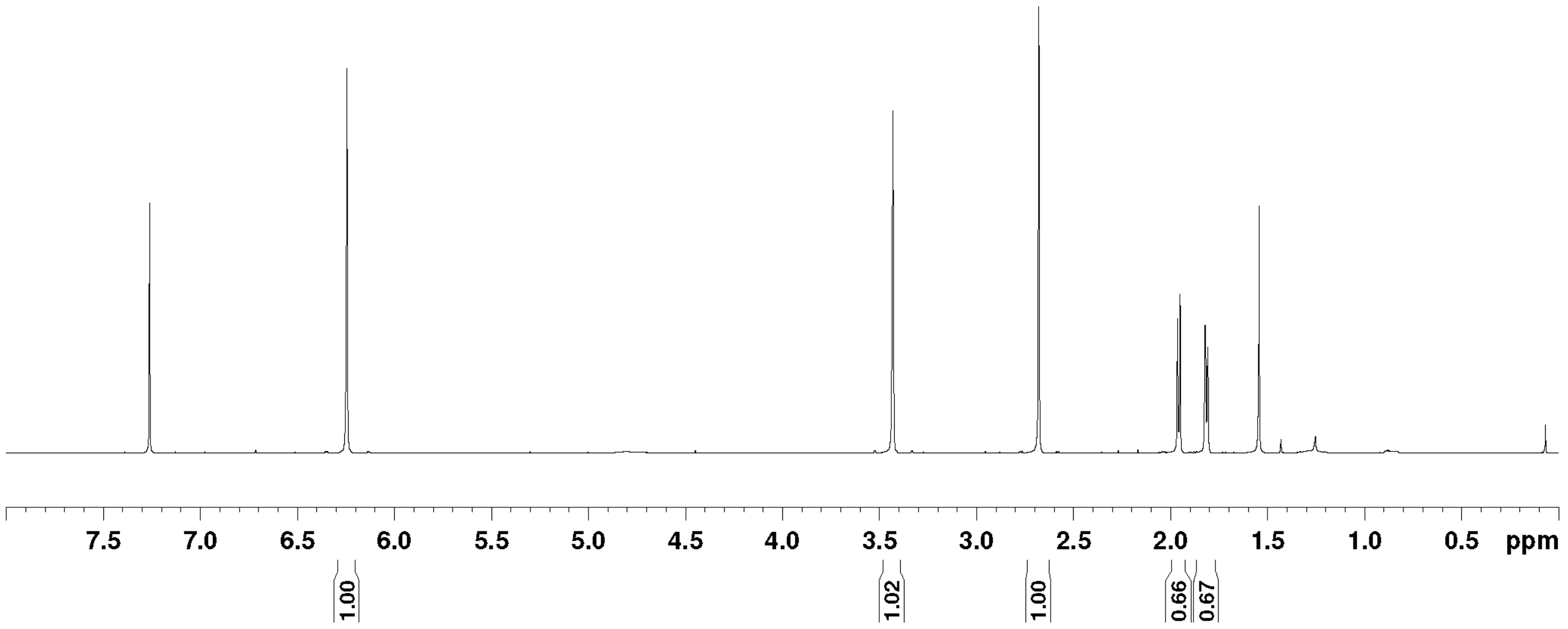

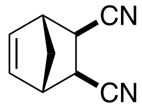

14_{CPD}-x

200Mz ^{13}C C_6D_6

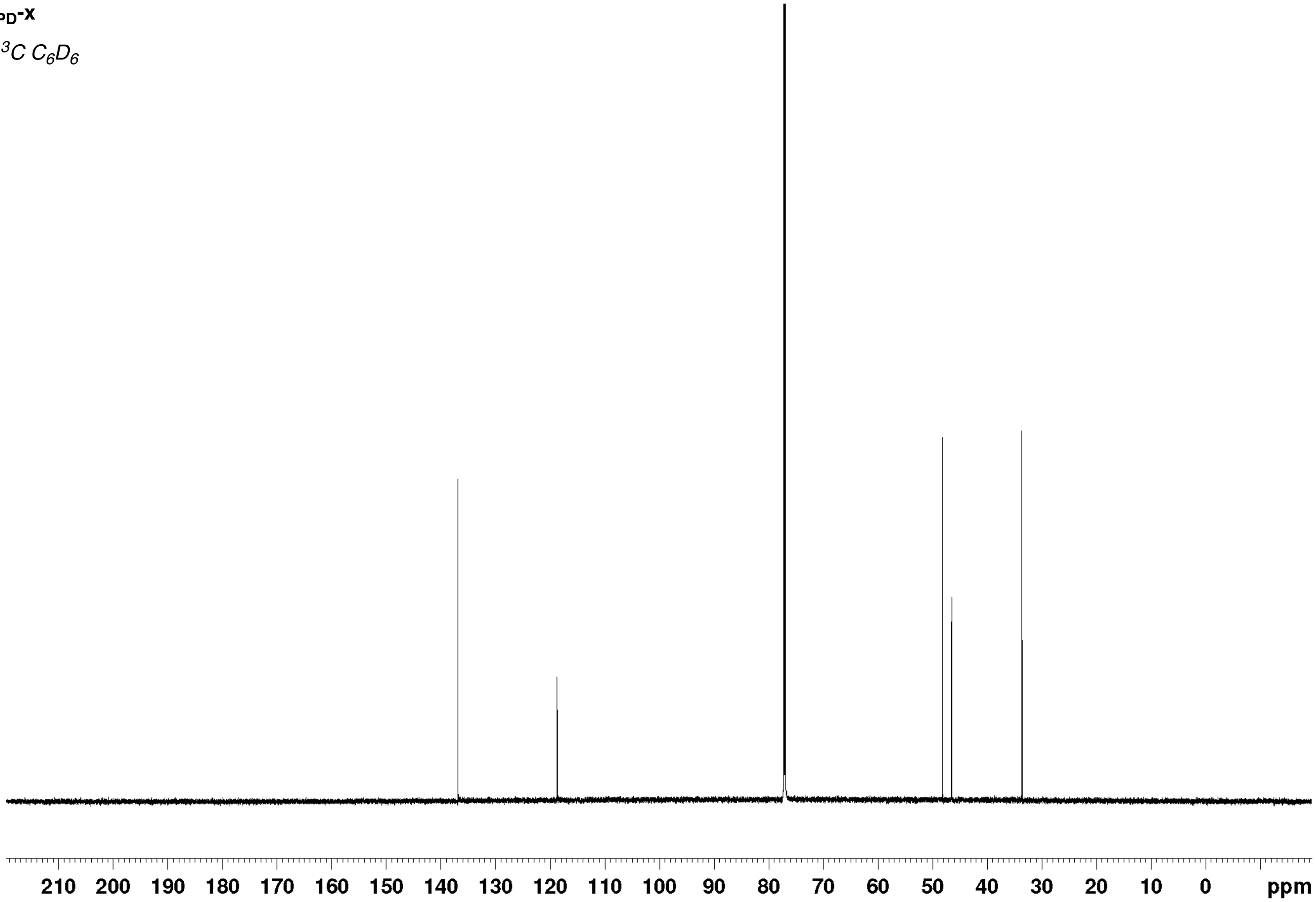

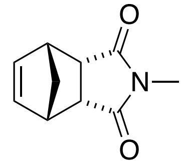

14_{CPD}-n

800Mz ^{1}H C_6D_6

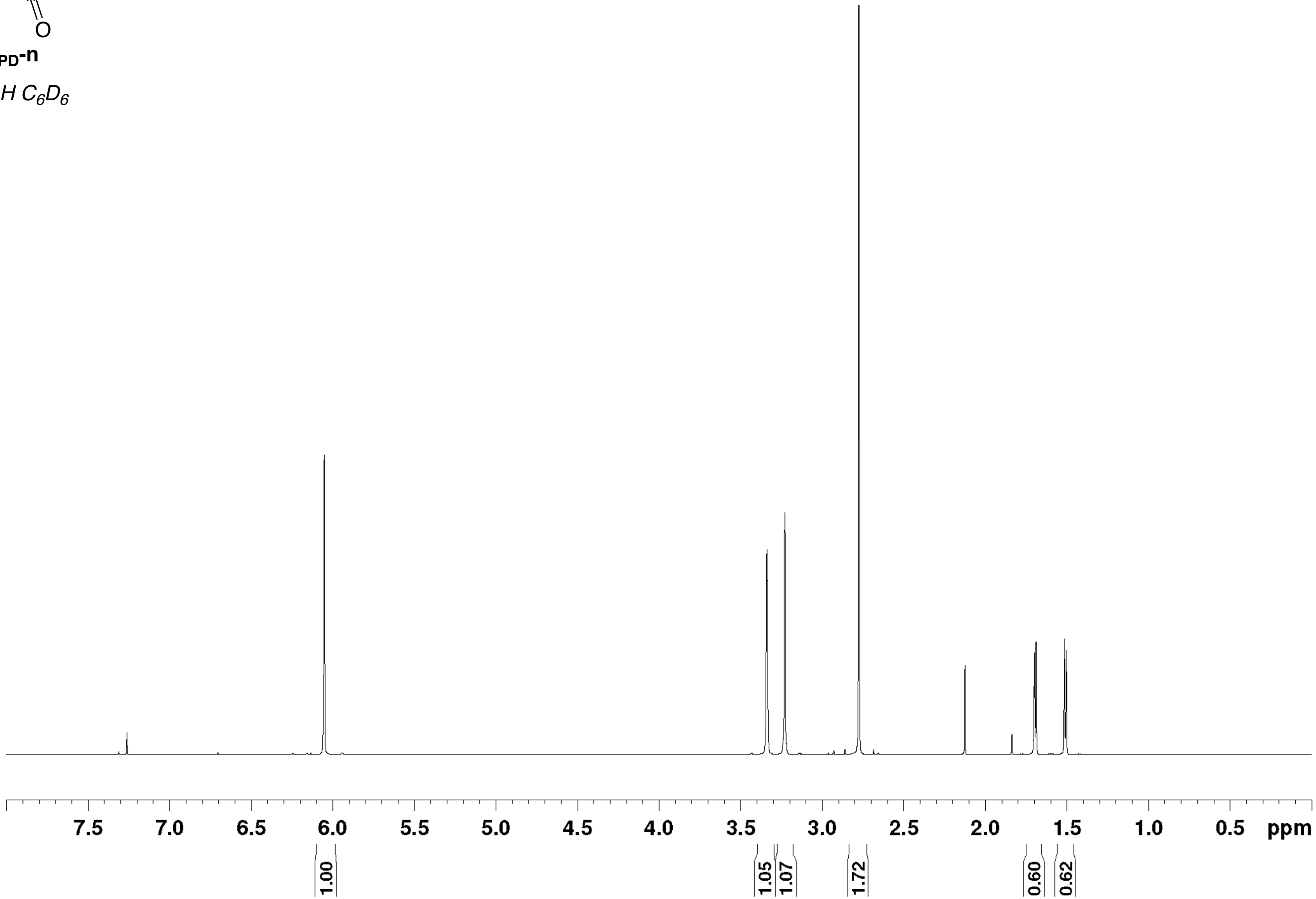

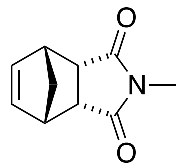

14_{CPD}-n

200Mz ^{13}C C_6D_6

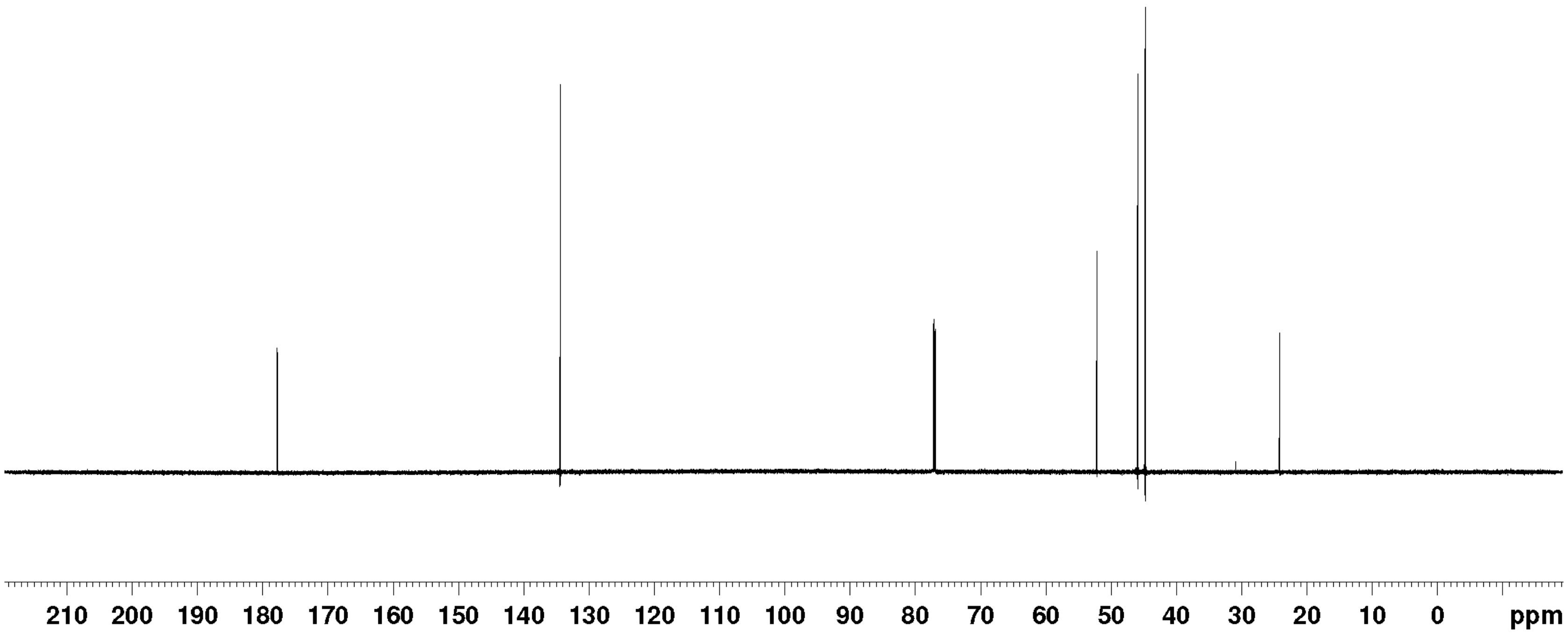

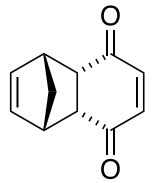

14_{CPD}-n

800Mz ^1H C_6D_6

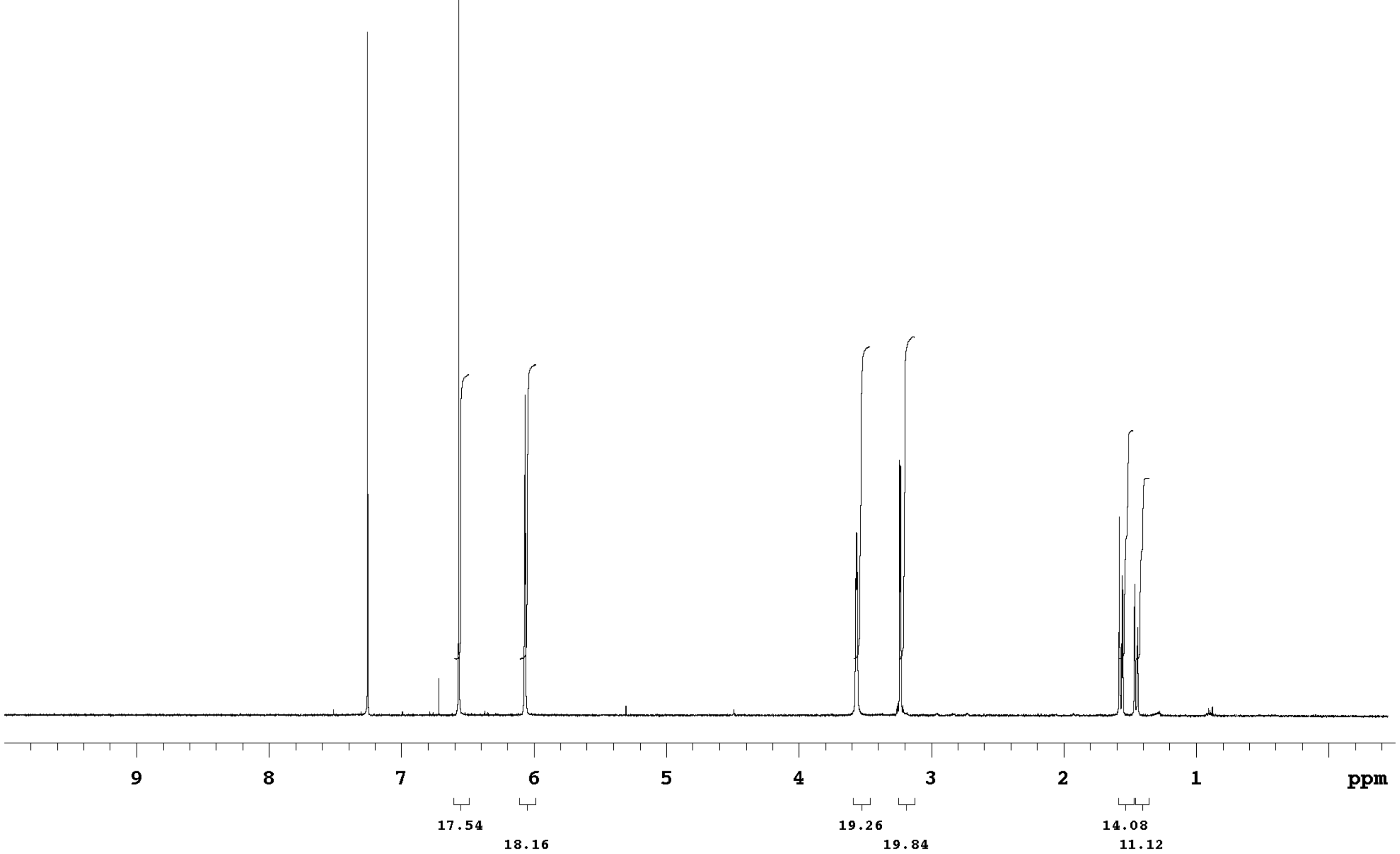

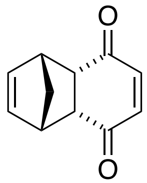

14_{CPD}-n

200Mz ^{13}C C_6D_6

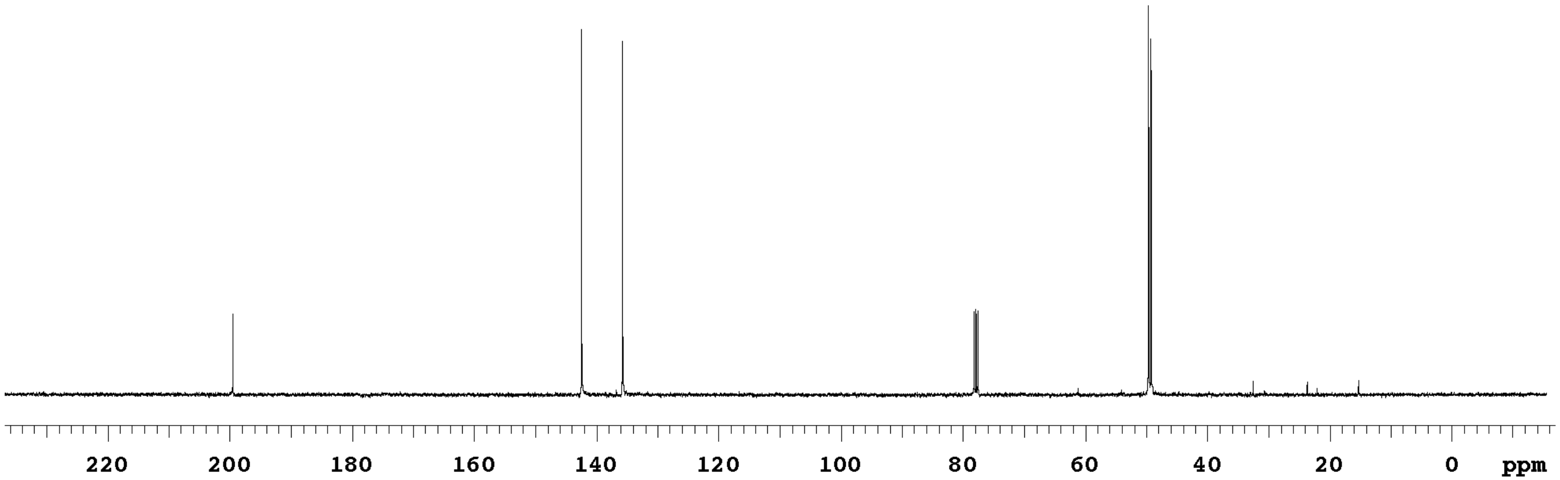

7 Computational Analysis

7.1 Table S1. HOMO-LUMO gap, relative $H^{‡}_{298K}$ and $G^{‡}_{298K}$ (kJ/mol) and Product Distribution (PD), and Charge Transfer properties arising from various stereochemical modes for butadiene with dienophiles.

	Calculated at B3LYP/6-31G(d)		Calculations at CBS-QB3									Charge Transfer calculated at B3LYP/6-31G(d)			
	HOMO-LUMO[a]		Gas phase				Benzene solvent				Exp.	Gas phase		Benzene solvent	
**dienophile**	**(ev)**	**TS mode**	**H‡(rel)**	**G‡(rel)**	**PD[b]**	**%endo[b]**	**H‡(rel)**	**G‡(rel)**	**PD[b]**	**%endo[b,c]**	**%endo**	**APT CT[f]**	**dipole[g]**	**APT CT[f]**	**dipole[g]**
acrolein	*4.4*	*endo s-cis*	0.0	0.0	79.3%	84	0.0	0.0	77.9%	86(76)	64	0.064	3.077	0.0718	3.642
		exo s-cis	4.8	4.1	15.0%		5.7	4.7	11.7%			0.055	3.411	0.0598	3.9597
		endo s-trans	7.4	7.2	4.3%		5.7	5.6	8.3%			0.066	4.034	0.0717	4.781
		exo s-trans	10.6	9.8	1.5%		9.7	9.0	2.1%			0.058	3.929	0.0629	4.6314
methyl vinyl ketone	*4.7*	*endo s-cis*	0.0	0.0	65.0%	66	0.0	0.0	61.7%	64(54)	65	0.048	2.710	0.054	3.2047
		exo s-cis	2.7	1.6	33.4%		3.7	1.4	34.9%			0.042	2.994	0.046	3.4649
		endo s-trans	8.2	9.7	1.3%		6.4	7.8	2.7%			0.052	3.761	0.056	4.4556
		exo s-trans	12.9	13.0	0.3%		11.7	11.1	0.7%			0.045	3.614	0.050	4.2736
methyl vinyl ketone $AlCl_3$	*2.4*	*endo s-cis*	0.0	0.0	99.6%	99	0.0	0.0	98.8%	99	>95[d]	0.136	11.035	0.1184	13.1388
		exo s-cis	16.3	14.1	0.3%		15.4	13.0	0.5%			0.123	12.010	0.0973	14.2743
		endo s-trans	23.4	22.8	0.1%		18.0	17.0	0.5%			0.134	13.502	0.1144	16.126
		exo s-trans	37.6	34.8	0%		31.9	27.1	0.1%			0.101	13.548	0.0801	16.4211
acrylic acid	*4.8*	*endo s-cis*	0.0	0.0	47.8%	53	0.0	0.0	52.6%	60(54)	60	0.063	1.727	0.0712	2.0607
		exo s-cis	1.4	0.4	41.0%		2.5	1.1	33.4%			0.055	1.922	0.059	2.2449
		endo s-trans	5.6	5.5	5.1%		4.8	4.8	7.6%			0.0606	2.6677	0.068	3.1372
		exo s-trans	5.8	5.1	6.1%		6.0	5.2	6.5%			0.055	2.625	0.060	3.0781
methyl acrylate	*5.0*	*endo s-cis*	0.0	0.0	52.7%	59	0.0	0.0	58.0%	67(60)	50	0.050	1.610	0.056	1.921
		exo s-cis	1.5	1.0	36.0%		2.4	1.9	27.4%			0.042	1.645	0.047	1.928
		endo s-trans	5.2	5.3	6.2%		4.2	4.7	8.8%			0.052	2.638	0.059	3.106
		exo s-trans	6.0	5.8	5.1%		5.9	5.7	5.8%			0.044	2.541	0.051	2.983
methyl methacrylate	*5.1*	*endo s-cis*	0.0	0.0	35.6%	57	0.0	0.0	30.1%	59	-	0.018	1.461	0.022	1.7625
		exo s-cis	1.1	0.6	27.4%		1.8	0.7	22.8%			0.014	1.660	0.017	1.9443
		endo s-trans	1.2	1.3	21.1%		0.6	0.1	29.4%			0.018	2.464	0.021	2.9134
		exo s-trans	2.1	2.0	16.0%		2.2	1.3	17.7%			0.016	2.397	0.020	2.8418
acrylamide	*5.3*	*endo s-cis*	1.9	3.1	21.9%	24	1.0	1.8	31.5%	35(39)	46	0.041	3.0667	0.044	3.6005
		exo s-cis	0.0	0.0	75.9%		0.0	0.0	64.2%			0.038	3.306	0.040	3.7799
		endo s-trans	7.6	9.4	1.7%		5.2	7.2	3.5%			0.041	3.927	0.047	4.5904
		exo s-trans	12.4	12.7	0.5%		10.5	11.1	0.7%			0.040	3.8665	0.041	4.5543
acrylonitrile	*4.6*	*endo*	1.6	1.6	33.6%	34	0.8	0.6	43.9%	44(46)	37	0.076	4.493	0.085	5.184
		exo	0.0	0.0	66.4%		0.0	0.0	56.1%			0.061	4.699	0.067	5.388
maleonitrile	*3.2*	*endo*	0.0	0.0	54.3%	54	0.0	0.0	66.4%	66(62)	70	0.116	6.631	0.133	7.574
		exo	0.2	0.5	45.7%		1.6	1.7	33.6%			0.096	7.001	0.107	7.993
maleic anhydride	*3.0*	*endo*	0.0	0.0	96.6%	97	0.0	0.0	97.8%	98	85[e]	0.147	5.217	0.165	5.946
		exo	8.9	8.3	3.4%		10.0	9.4	2.2%			0.123	5.602	0.136	6.367
N-methylmaleimide	*3.5*	*endo*	0.0	0.0	98.7%	99	0.0	0.0	98.8%	99	>99	0.098	2.335	0.111	2.747
		exo	11.9	10.7	1.3%		13.0	10.9	1.2%			0.078	2.432	0.083	2.831
benzoquinone	*2.6*	*endo*	0.0	0.0	96.2%	96	0.0	0.0	97.8%	98	-	0.118	2.634	0.134	3.163
		exo	8.8	8.0	3.8%		10.2	9.4	2.2%			0.093	2.405	0.102	2.873
furan-2(5H)-one	*4.9*	*endo*	0.0	0.0	77.4%	77	0.0	0.0	82.4%	82(74)	73	0.070	4.867	0.075	5.645
		exo	3.6	3.1	22.6%		4.5	3.9	17.6%			0.059	4.990	0.062	5.755
alpha-methylene butyrolactone	*4.9*	*endo*	2.7	2.8	24.4%	24	1.8	2.5	27.5%	35(32)	39	0.037	4.639	0.040	5.380
		exo	0.0	0.0	75.6%		0.0	0.0	72.5%			0.040	4.968	0.043	5.698
alpha-methylene butyrolactone $AlCl_3$	*3.0*	*endo*	0.0	0.0	86.6%	87	0.0	0.0	88.6%	89	-	0.122	13.320	0.109	15.628
		exo	8.5	4.6	13.4%		```	5.1	11.4%			0.122	14.210	0.109	16.646

[a] Calculated at B3LYP/6-31G(d). [b] Calculated from the relative free energies. [c] values in parentheses calculated at 145 °C. [d] Experimental value from the reaction using $MeAlCl_2$. [e] from Stephenson, L. M.; Smith, D. E.; Current, S. P. *J. Org. Chem.* **1982**, *47*, 4170-4171. [f] Atomic Polar Tensor charge transferred from diene to dienophile in TS. [g] Dipole moment (D) in TS.

.2 Table S2. HOMO-LUMO gap, relative $H^{\ddagger}_{298K}$ and $G^{\ddagger}_{298K}$ (kJ/mol) and Product Distribution (PD), and Charge Transfer properties arising from various stereochemical modes for cyclopentadiene with dienophiles.

	**calculated at B3LYP/6-31G(d)**		**Calculations at CBS-QB3**									**Charge Transfer calculated at B3LYP/6-31G(d)**			
			**Gas phase**				**Benzene solvent**				**Exp.**	**Gas phase**		**Benzene solvent**	
**dienophile**	**HOMO-LUMO[a] (ev)**	**TS mode**	**$H^{\ddagger}$(rel)**	**$G^{\ddagger}$(rel)**	**PD[b]**	**%endo[b]**	**$H^{\ddagger}$(rel)**	**$G^{\ddagger}$(rel)**	**PD[b]**	**%endo[b,c]**	**%endo**	**APT CT[h]**	**Dipole[i]**	**APT CT[h]**	**Dipole[i]**
acrolein	*4.0*	*endo s-cis*	0.0	0.0	66.1%	76	0.0	0.0	64.2%	81	73	0.120	3.679	0.136	4.3688
		exo s-cis	2.3	2.9	20.4%		3.4	3.9	13.4%			0.109	3.636	0.122	4.336
		endo s-trans	5.0	4.7	9.8%		3.5	3.4	16.3%			0.140	4.451	0.160	5.2984
		exo s-trans	7.4	7.1	3.7%		6.1	5.9	6.1%			0.121	4.451	0.134	5.3287
methyl vinyl ketone	*4.3*	*endo s-cis*	0.0	0.0	86.0%	87	0.0	0.0	83.4%	86	79	0.1071	3.227	0.121	3.860
		exo s-cis	2.8	4.6	13.1%		3.3	4.5	13.6%			0.0977	3.128	0.1081	3.717
		endo s-trans	8.2	11.3	0.9%		6.1	8.3	2.9%			0.1218	4.103	0.1387	4.888
		exo s-trans	18.1	19.0	0.0%		16.5	16.4	0.1%			0.0977	3.976	0.125	4.781
acrylic acid	*4.4*	*endo s-cis*	0.0	0.0	74.0%	80	0.0	0.0	78.7%	87	71	0.123	2.397	0.140	2.8705
		exo s-cis	2.6	3.4	18.9%		3.5	4.7	12.0%			0.117	2.209	0.131	2.6587
		endo s-trans	6.1	6.4	5.6%		5.1	5.7	8.0%			0.125	3.1508	0.143	3.7358
		exo s-trans	9.5	9.7	1.5%		9.6	10.2	1.3%			0.117	3.036	0.132	3.6229
methyl acrylate	*4.6*	*endo s-cis*	0.0	0.0	63.9%	72	0.0	0.0	64.6%	76	77	0.112	2.094	0.130	2.532
		exo s-cis	2.1	2.2	26.3%		3.1	2.6	22.2%			0.104	1.856	0.119	2.281
		endo s-trans	5.1	5.1	8.3%		4.2	4.2	11.5%			0.116	2.998	0.134	3.561
		exo s-trans	9.3	9.4	1.4%		9.1	8.9	1.7%			0.107	2.864	0.122	3.418
methyl methacrylate	*4.7*	*endo s-cis*	2.5	1.8	25.6%	35	1.7	1.1	27.2%	44	45[e]	0.083	1.815	0.097	2.2126
		exo s-cis	0.0	0.0	52.9%		0.0	0.0	41.6%			0.075	1.712	0.086	2.0929
		endo s-trans	4.7	4.4	9.1%		3.0	2.2	16.8%			0.083	2.768	0.101	3.2987
		exo s-trans	3.9	3.6	12.4%		3.2	2.6	14.4%			0.075	2.742	0.088	3.2993
acrylamide	*4.9*	*endo s-cis*	0.0	0.0	56.9%	59	0.0	0.0	72.6%	76	64	0.103	3.1701	0.116	3.7495
		exo s-cis	0.3	0.8	40.7%		1.7	2.8	23.5%			0.102	3.0633	0.114	3.5576
		endo s-trans	7.7	8.1	2.2%		6.0	7.4	3.7%			0.107	4.374	0.124	5.1107
		exo s-trans	13.2	13.9	0.2%		13.3	14.8	0.2%			0.110	3.7928	0.120	4.5302
acrylonitrile	*4.2*	*endo*	0.6	0.3	47.1%	47	0.0	0.0	60.2%	60	55	0.137	5.058	0.154	5.863
		exo	0.0	0.0	52.9%		0.8	1.0	39.8%			0.124	4.922	0.137	5.712
maleonitrile	*2.8*	*endo*	0.3	0.4	46.3%	46	0.0	0.0	59.4%	59	73	0.187	7.2215	0.214	8.284
		exo	0.0	0.0	53.7%		1.1	1.0	40.6%			0.179	7.172	0.205	8.238
maleic anhydride	*2.6*	*endo*	0.0	0.0	96.8%	97	0.0	0.0	97.0%	97	>99	0.221	5.947	0.257	6.811
		exo	8.6	8.5	3.2%		9.7	8.6	3.0%			0.209	5.879	0.238	6.749
N-methylmaleimide	*3.1*	*endo*	0.0	0.0	99.5%	99.5	0.0	0.0	99.8%	>99	>99	0.166	3.116	0.192	3.645
		exo	12.8	13.3	0.5%		14.2	14.9	0.2%			0.156	2.865	0.178	3.376
benzoquinone	*2.2*	*endo*	0.0	0.0	99.0%	99	0.0	0.0	99.4%	99.5	>99 [f]	0.197	3.126	0.237	3.715
		exo	12.2	11.4	1.0%		13.7	12.7	0.6%			0.181	2.731	0.207	3.290
furan-2(5H)-one	*4.5*	*endo*	0.0	0.0	85.9%	86	0.0	0.0	89.6%	90	80	0.134	5.279	0.152	6.137
		exo	4.0	4.5	14.1%		5.0	5.3	10.4%			0.135	5.208	0.150	6.086
alpha-methylene butyrolactone	*4.5*	*endo*	7.0	6.3	7.3%	7	6.0	5.3	10.5%	11	8 [g]	0.102	4.905	0.115	5.715
		exo	0.0	0.0	92.7%		0.0	0.0	89.5%			0.101	4.917	0.120	5.723
alpha-methylene butyrolactone $AlCl_3$	*2.6*	*endo*	4.7	3.0	23.1%	23	4.4	5.2	11.0%	11(8)[d]	6 [g]	0.177	13.771	0.163	16.224
		exo	0.0	0.0	76.9%		0.0	0.0	89.0%			0.168	14.040	0.147	16.626

[a] Calculated at B3LYP/6-31G(d). [b] Calculated from the relative free energies. [c] value calculated at 80 °C. [d] value in parenthesis calculated in dichloromethane at reflux. [e] reaction reported by Kobuke et al. JACS, **1970**, *92*, 6548-6553, reaction conducted at 100 °C without solvent. [f] reaction reported by Mal et al. *Eur. J. Org. Chem.* **2008**, 3014-3020, reduction conducted in dichloromethane in 0 °C. [g] reaction reported by in Buono et al. *Tetrahedron Letters*, **1990**, *31*, 4863-4866 in dichloromethane at 40 °C (uncatalyzed) and -15 °C ($AlCl_3$ catalysed). [h] from Stephenson, L. M.; Smith, D. E.; Current, S. P. *J. Org. Chem.* **1982**, *47*, 4170-4171. [i] Atomic Polar Tensor charge transferred from diene to dienophile in TS. [g] Dipole moment (D) in TS.

7.3 Geometry Optimisation and Energy Benchmark

The CBS-QB3 procedure employs the reliable B3LYP/6-31G(d) method for transition structure geometry optimisation. This was compared to transition structure geometry optimisations using the dispersion corrected B3LYP-D3/6-311++G(d,p) method for several examples. The results are summarised in Figure S8 and Figure S9. RSMS values range between 0.017 – 0.038 Å, and the bond forming distances are in close agreement. Table S3 compares the CBS-QB3 and CCSD(T) // B3LYP-D3/6-311++G(d,p) computed stereoselectivities in benzene solvent at the relevant experimental temperatures.

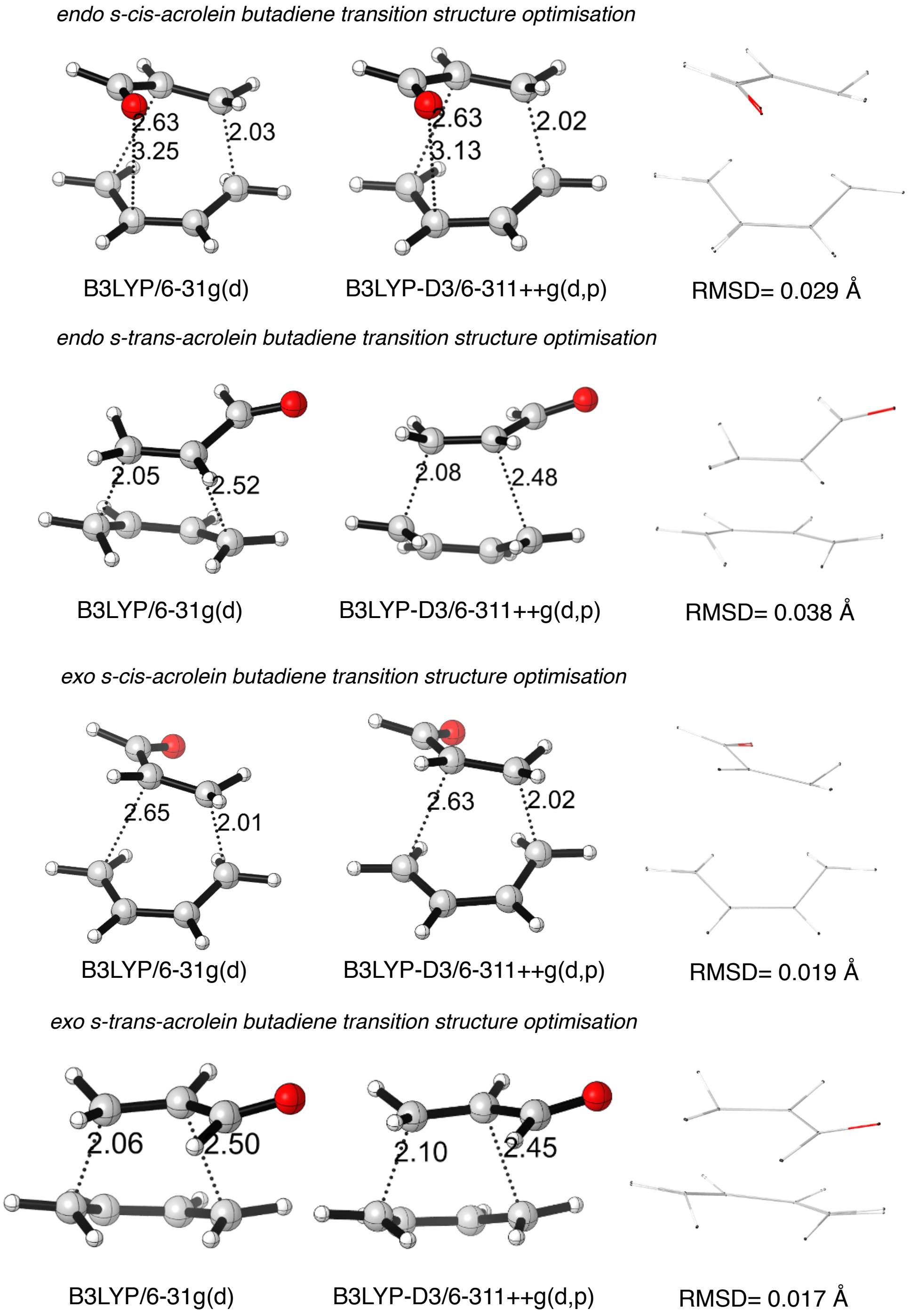

Figure S8. Comparisons of geometry optimisations between B3LYP/6-31g(d) and B3LYP-D3/6-311++g(d,p) in the reaction of butadiene and acrolein.

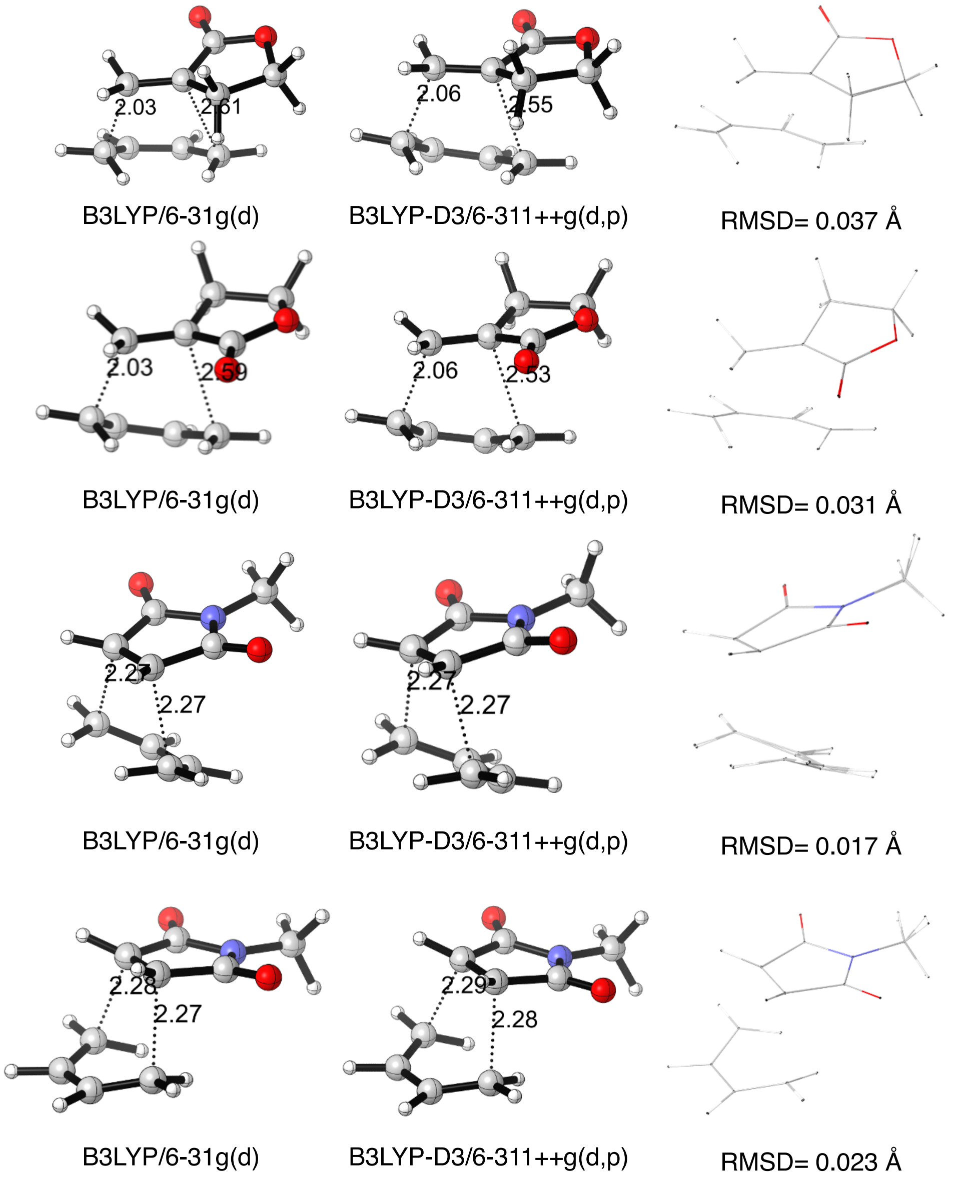

Figure S9. Comparisons of geometry optimisations between B3LYP/6-31g(d) and B3LYP-D3/6-311++g(d,p) in the reaction of butadiene with 3-methylenedihydrofuran-2(3H)-one and N-methylmaleimide.

Table S3. Benchmark of CBS-QB3 energies against CCSD(T)/6-311++G(d.p) in the reaction of butadiene.

Dienophile	Computational Method (benzene phase)	endo:exo (Exp.)	endo:exo (Calc.)	ΔΔG‡ (Calc.) kJmol^{-1} (s-cis endo: s-trans endo): (s-cis exo: s-trans exo)
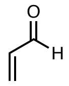	CBS-QB3	64:36	86:14	(0.0:5.6):(4.7:9.0)
	CCSD(T)/6-311++G(d.p) // B3LYP-D3/6-311++G(d.p)	64:36	77:23	(0.0:6.5):(4.3:9.6)
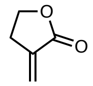	CBS-QB3	39:61	27:73	2.5:0.0
	CCSD(T)/6-311++G(d.p) // B3LYP-D3/6-311++G(d.p)	39:61	36:64	1.9
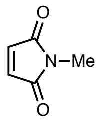	CBS-QB3	>99:1	99:1	0.0:10.9
	CCSD(T)/6-311++G(d.p) // B3LYP-D3/6-311++G(d.p)	>99:1	99.8:0.2	0.0:15.3

7.4 Explanation of how a lower HOMO-LUMO gap leads to increased endo-selectivity

(Based upon a discussion published earlier – see main text, reference 41.)
For normal electron demand Diels-Alder reactions, within the context of PMO theory, the dominant stabilizing interaction in the DA TS is between the diene HOMO and the dienophile LUMO.[1] The TS stabilization energy, ΔE^{SOI}, arising from SOIs is given, to second-order, by[2] the equation

$$\Delta E^{SOI} \approx \frac{|H_{HL}|^2}{E_H - E_L}$$

where H_{HL} is the matrix element of the interaction between the diene HOMO (H) and the dienophile LUMO (L), the magnitude of which depends on the magnitudes of the atomic orbital coefficients of the HOMO and LUMO at the sites where the SOIs occur, and (E_H - E_L) is the diene HOMO - dienophile LUMO energy gap.

If a substituent on a dienophile decreases its LUMO energy, then it decreases the denominator of this equation, thereby increasing the SOI interaction energy. The overall effect of decreasing the HOMO-LUMO gap, therefore, is to enhance endo-selectivity, since SOIs are only possible in the *endo*-TS.

1. Fleming, I. *Frontier Orbitals and Organic Chemical Reactions*; Wiley-Interscience: New York, 1976; Chapter 4.
2. Dewar, M. J. S.; Dougherty, R. C. *The PMO Theory of Organic Chemistry*; Plenum Press: New York, 1975.

7.5 Cartesian Coordinates of Optimised Structures and CBS-QB3 Energies

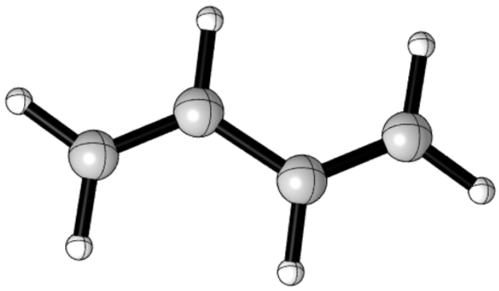

trans-butadiene
CBS-QB3 PCM benzene
Energy (0K) = -155.667847
Enthalpy (298K) = -155.662228
Gibbs free energy (298K) = -155.694305

6	-0.608620	-0.399895	-0.000025
6	-1.845125	0.109668	0.000050
6	0.608648	0.399995	-0.000073
6	1.845091	-0.109733	0.000017
1	-0.476047	-1.480070	-0.000119
1	-2.015449	1.181890	0.000082
1	-2.722671	-0.525770	0.000001
1	0.476210	1.480181	-0.000066

1	2.722752	0.525546	0.000152
1	2.015240	-1.181984	0.000134

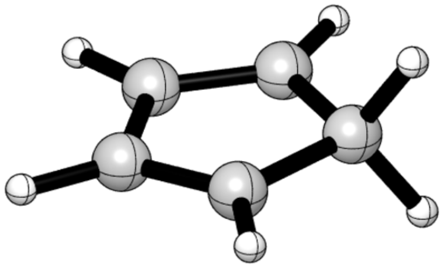

cyclopentadiene
CBS-QB3 PCM benzene
Energy (0K) = -193.713757
Enthalpy (298K) = -193.708623
Gibbs free Energy (298K) = -193.739712

6	0.000000	1.179407	0.281214
6	0.000000	0.734561	-0.989840
6	0.000000	-0.734561	-0.989840
6	0.000000	-1.179407	0.281214
6	0.000000	0.000000	1.215890
1	0.000000	-1.347283	-1.882557
1	0.000000	-2.210205	0.608537
1	0.876476	0.000000	1.878104
1	-0.876476	0.000000	1.878104
1	0.000000	2.210205	0.608537
1	0.000000	1.347283	-1.882557

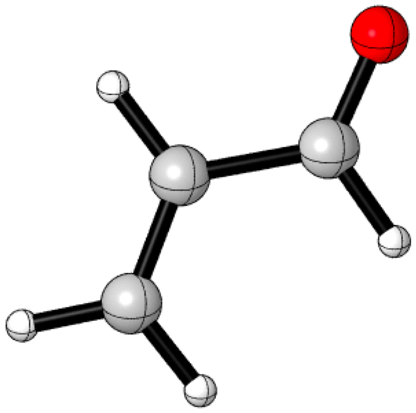

trans-acrolein
CBS-QB3 PCM benzene
Energy (0K) = -191.602481
Enthalpy (298K) = -191.597156
Gibbs free Energy (298K) = -191.628773

6	-0.676156	0.349363	-0.000007
8	-1.792129	-0.121977	0.000005
6	0.562222	-0.447645	-0.000005
6	1.760016	0.141066	-0.000006
1	-0.518386	1.449549	0.000008
1	0.444889	-1.526851	0.000011

1	2.682528	-0.427074	0.000030
1	1.851503	1.223489	0.000023

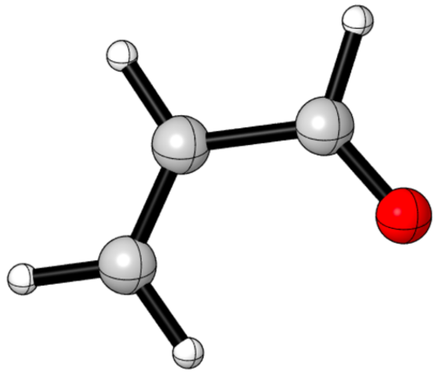

cis-acrolein
CBS-QB3 PCM benzene
Energy (0K) = -191.598908
Enthalpy (298K) = -191.593576
Gibbs free energy (298K) = -191.625359

6	-0.811316	0.461856	0.000018
8	-1.404877	-0.594836	0.000000
6	0.666163	0.598856	-0.000003
6	1.469913	-0.466155	-0.000038
1	-1.370796	1.419337	-0.000068
1	1.063167	1.609626	0.000055
1	2.549219	-0.371746	0.000081
1	1.048863	-1.465873	0.000075

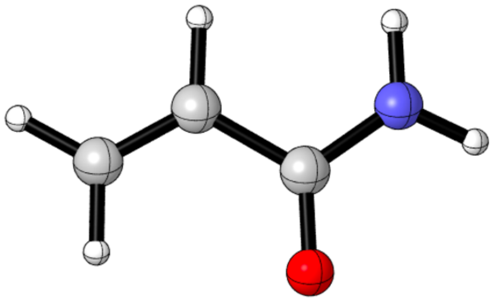

cis-acrylamide
CBS-QB3 PCM benzene
Energy (0K) = -246.909072
Enthalpy (298K) = -246.902355
Gibbs free energy (298K) = -246.937919

6	0.800725	-0.648922	-0.000038
6	1.975553	-0.026104	0.000022
1	0.742685	-1.733991	-0.000098
1	2.011427	1.057749	0.000126
6	-0.478781	0.128935	-0.000015
8	-0.516761	1.350991	0.000006
7	-1.605599	-0.637612	-0.000127
1	-2.502859	-0.179050	0.000363
1	-1.575461	-1.642894	0.000593
1	2.912504	-0.569919	0.000046

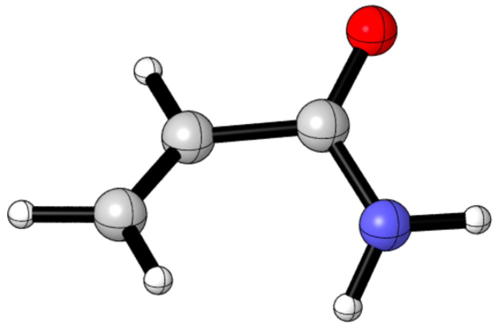

trans-acrylamide
CBS-QB3 PCM benzene
Energy (0K) = -246.906816
Enthalpy (298K) = -246.900313
Gibbs free Energy (298K) = -246.935257

6	-0.848985	-0.587581	0.183235
6	-1.934349	0.114561	-0.136128
1	-0.928689	-1.615035	0.521783
1	-1.877629	1.124124	-0.529764
6	0.566076	-0.129551	0.025921
8	1.468226	-0.938097	-0.138845
7	0.779912	1.219270	0.042522
1	1.736657	1.535049	0.000081
1	0.094894	1.851425	0.421735
1	-2.926879	-0.310248	-0.038898

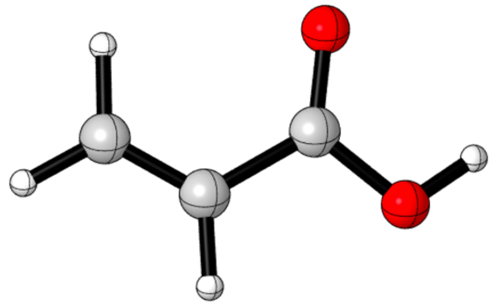

cis-acrylic acid
CBS-QB3 PCM benzene
Energy (0K) = -266.782849
Enthalpy (298K) = -266.776883
Gibbs free energy (298K) = -266.810836

6	-1.968167	0.038139	-0.000025
6	-0.815712	-0.627338	0.000027
1	-1.975905	1.122398	-0.000105
1	-0.770868	-1.709884	0.000125
6	0.478800	0.096789	-0.000020
8	0.623883	1.297007	0.000032
8	1.522060	-0.765728	-0.000020
1	2.330915	-0.231713	-0.000029
1	-2.921205	-0.476570	0.000024

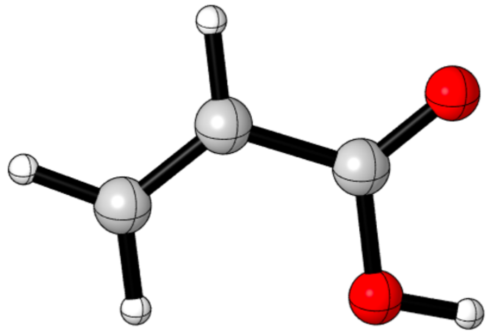

trans-acrylic acid
CBS-QB3 PCM benzene
Energy (0K) = -266.782576
Enthalpy (298K) = -266.776575
Gibbs free energy (298K) = -266.810632

6	-1.922832	0.117851	-0.000138
6	-0.830864	-0.644563	0.000137
1	-1.856631	1.198971	-0.000526
1	-0.886623	-1.726688	0.000584
6	0.553223	-0.120548	0.000017
8	1.538807	-0.822281	-0.000156
8	0.623114	1.231304	0.000120
1	1.563755	1.461889	0.000062
1	-2.913025	-0.322796	0.000069

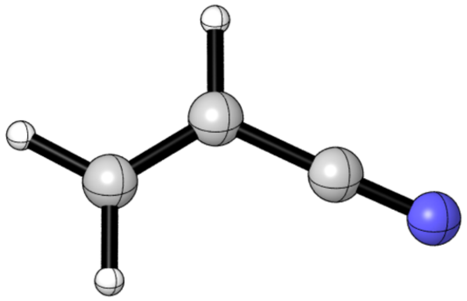

acrylonitrile
CBS-QB3 PCM benzene
Energy (0K) = -170.542412
Enthalpy (298K) = -170.537321
Gibbs free energy (298K) = -170.568279

6	1.318288	0.980434	0.000000
6	0.000000	0.771660	0.000000
1	1.715253	1.987670	0.000000
1	2.026387	0.160727	0.000000
1	-0.702036	1.598699	0.000000
6	-0.579418	-0.532968	0.000000
7	-1.067546	-1.580265	0.000000

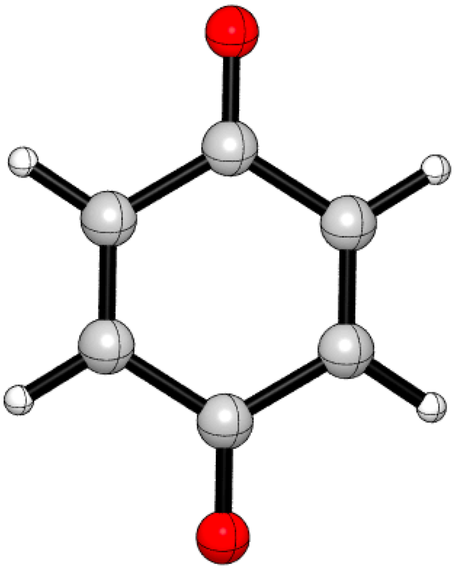

benzoquinone
CBS-QB3 PCM_benzene
Energy (0K) = -380.872165
Enthalpy (298K) = -380.864913
Gibbs free energy (298K) = -380.901463

6	0.000000	0.000000	1.443070
6	0.000000	1.268020	-0.669489
6	0.000000	-1.268020	-0.669489
6	0.000000	0.000000	-1.443070
6	0.000000	-1.268020	0.669489
6	0.000000	1.268020	0.669489
1	0.000000	2.180768	-1.254773
1	0.000000	-2.180768	-1.254773
1	0.000000	-2.180768	1.254773
1	0.000000	2.180768	1.254773
8	0.000000	0.000000	2.663004
8	0.000000	0.000000	-2.663004

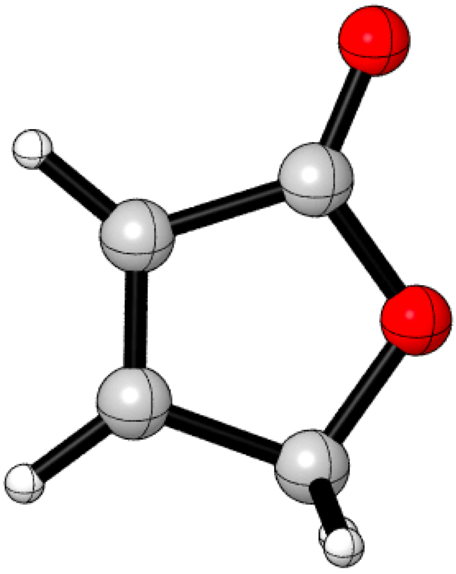

furan-2(5H)-one

CBS-QB3 PCM_Benzene
Energy (0K) = -304.816582
Enthalpy (298K) = -304.810955
Gibbs free energy (298K) = -304.844547

6	-1.317713	0.781830	0.000014
6	-0.054876	1.204111	-0.000069
6	0.841851	0.024404	-0.000093
8	2.040856	-0.037788	0.000130
8	0.047931	-1.099479	-0.000144
6	-1.332564	-0.715756	0.000095
1	-2.217961	1.381331	0.000129
1	0.323026	2.215014	-0.000080
1	-1.817626	-1.132831	0.888123
1	-1.817924	-1.132909	-0.887737

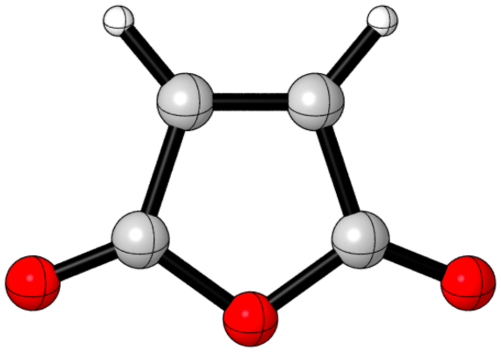

maleic anhydride
CBSQB3 PCM Benzene
Energy (0K) = -378.791493
Enthalpy (298K) = -378.785321
Gibbs free energy (298K) = -378.820596

6	-0.665818	1.257806	-0.000023
6	0.665820	1.257803	0.000009
6	-1.130962	-0.156727	0.000007
6	1.130962	-0.156728	-0.000197
8	2.235969	-0.601827	0.000092
8	-2.235970	-0.601826	0.000031
1	1.355234	2.088681	0.000087
1	-1.355230	2.088685	0.000025
8	-0.000002	-0.970133	0.000016

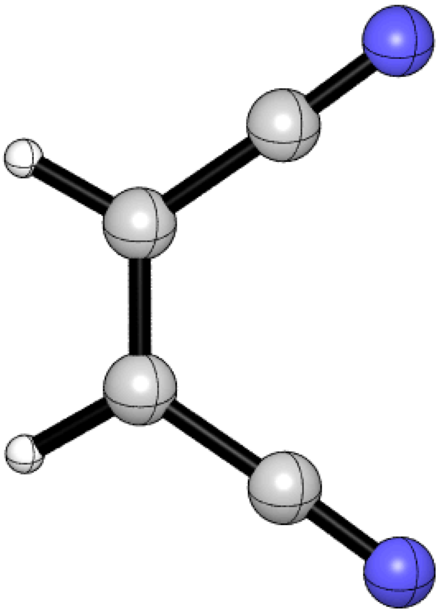

maleonitrile
CBSQB3 PCM Benzene
Energy (0K) = -262.659380
Enthalpy (298K) = -262.652870
Gibbs free energy (298K) = -262.688632

6	0.000042	1.042087	0.671601
6	0.000042	1.042087	-0.671601
6	-0.000027	-0.132020	1.474380
6	-0.000027	-0.132020	-1.474380
7	-0.000027	-1.063647	2.157016
7	-0.000027	-1.063647	-2.157016
1	0.000098	1.985129	-1.205688
1	0.000098	1.985129	1.205688

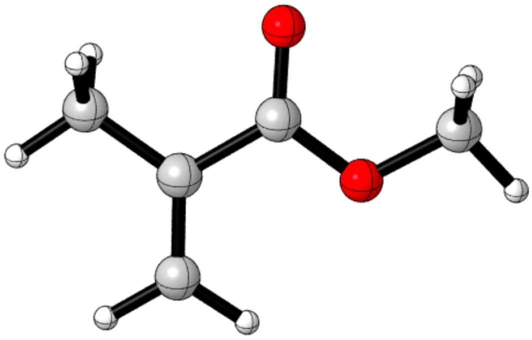

trans-methylmethacrylate
CBSQB3 PCM Benzene
Energy (0K) = -345.225791
Enthalpy (298K) = -345.216708
Gibbs free energy (298K) = -345.258451

6	-0.240900	-0.302520	-0.000272
8	-0.479269	-1.490408	-0.000029
8	-1.205081	0.639410	-0.000043
6	1.144958	0.259829	-0.000045
6	1.357303	1.578459	0.000004

1	2.364514	1.980434	0.000147
1	0.536681	2.283185	-0.000107
6	-2.558884	0.151262	0.000132
1	-3.187566	1.038567	0.000234
1	-2.747867	-0.452249	-0.888771
1	-2.747629	-0.452278	0.889067
6	2.245481	-0.765958	0.000108
1	2.172440	-1.416290	0.876012
1	2.172700	-1.416269	-0.875834
1	3.223786	-0.283544	0.000259

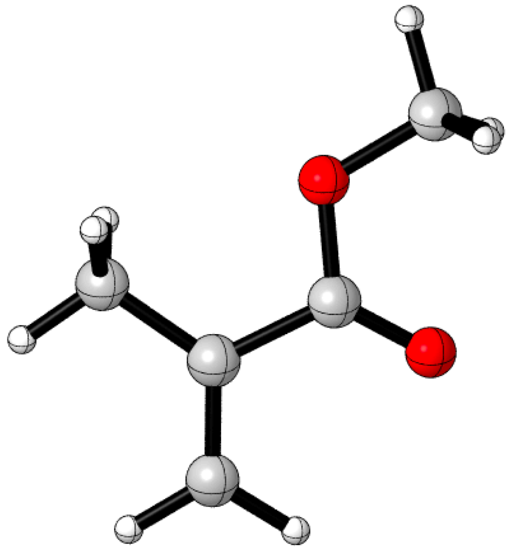

cis-methylmethacrylate
CBSQB3 PCM Benzene
Energy (0K) = -345.225512
Enthalpy (298K) = -345.216427
Gibbs free Energy (298K) = -345.258233

6	0.243887	-0.403557	-0.000126
8	0.629774	-1.550687	-0.000123
8	1.083399	0.653189	0.000188
6	-1.189111	0.034832	-0.000152
6	-2.118912	-0.923012	-0.000301
1	-3.175942	-0.683755	-0.000335
1	-1.833290	-1.967479	-0.000396
6	2.488121	0.339690	0.000396
1	3.000335	1.298833	0.000752
1	2.749816	-0.236250	0.889021
1	2.750180	-0.235776	-0.888430
6	-1.501910	1.507444	-0.000015
1	-1.074124	2.000867	0.876674
1	-1.074035	2.001046	-0.876561
1	-2.580773	1.670113	-0.000053

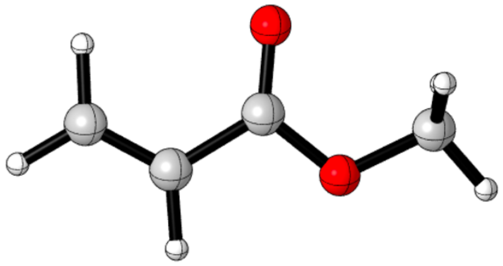

cisoid methyl acrylate
CBSQB3 PCM Benzene
Energy (0K) = -305.994399
Enthalpy (298K) = -305.986772
Gibbs free energy (298K) = -306.024859

6	-0.045075	0.121468	0.000023
8	0.058350	1.326785	0.000091
8	1.012608	-0.717639	-0.000040
6	-1.316953	-0.643437	-0.000030
6	-2.491197	-0.017459	0.000012
1	-1.235453	-1.723784	-0.000101
1	-3.426249	-0.564130	-0.000025
1	-2.535497	1.065877	0.000086
6	2.309058	-0.093083	-0.000046
1	3.026228	-0.910256	-0.000660
1	2.433926	0.527582	-0.888480
1	2.434382	0.526604	0.889014

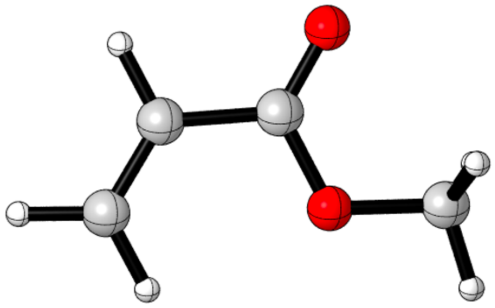

transoid methyl acrylate
CBSQB3 PCM Benzene
Energy (0K) = -305.993666
Enthalpy (298K) = -305.986008
Gibbs free energy (298K) = -306.024272

6	-0.013370	0.485890	0.000047
8	0.576184	1.543602	0.000123
8	0.608097	-0.710596	-0.000037
6	-1.492237	0.376078	0.000027
6	-2.168706	-0.770942	-0.000072
1	-1.995635	1.335996	0.000101
1	-3.252530	-0.781395	-0.000081
1	-1.658589	-1.726302	-0.000145
6	2.046966	-0.669796	-0.000063
1	2.367344	-1.708908	-0.000506

1	2.414601	-0.155027	-0.888790
1	2.414645	-0.155795	0.889096

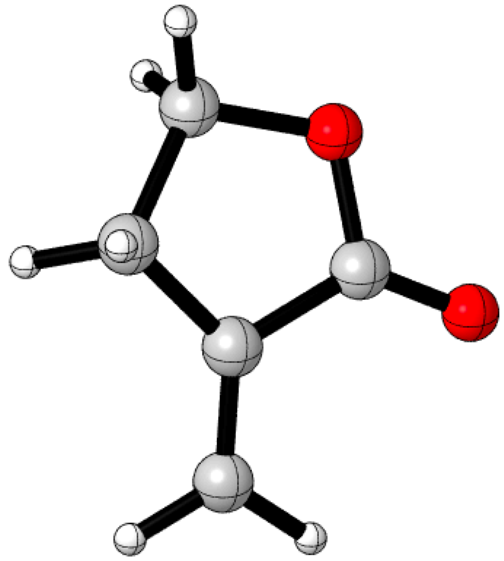

alpha-methylene butyrolactone
CBSQB3 PCM Benzene
Energy (0K) = -344.039024
Enthalpy (298K) = -344.031960
Gibbs free Energy (298K) = -344.069154

6	-0.538311	0.677891	0.023389
6	0.825159	1.291116	0.189589
6	1.754461	0.117323	-0.168591
8	0.968161	-1.088542	0.007782
6	-0.362850	-0.807286	0.030835
1	0.978302	1.596614	1.229344
1	0.991925	2.161892	-0.445543
1	2.077260	0.154201	-1.211365
1	2.627888	0.033247	0.475562
8	-1.202389	-1.667039	0.057905
6	-1.732349	1.246199	-0.113424
1	-1.862817	2.322048	-0.130262
1	-2.615390	0.625195	-0.214022

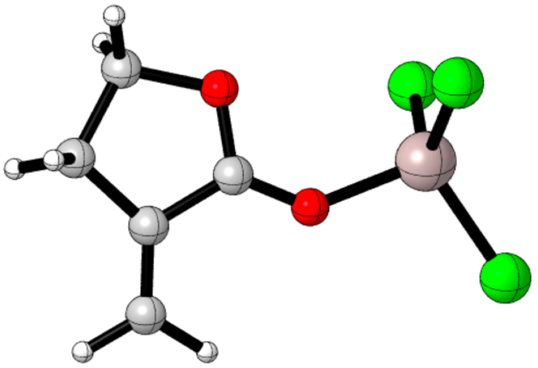

alpha-methylene butyrolactone $AlCl_3$ complex
CBSQB3 PCM Benzene
Energy (0K) = -1965.577361
Enthalpy (298K) = -1965.563409; Gibbs free Energy (298K) = -1965.620423

6	-2.688893	-0.939104	0.029246
6	-3.796881	0.079860	0.148717
6	-3.069715	1.409270	-0.116370
8	-1.631384	1.103309	-0.007890
6	-1.429713	-0.185900	0.019647
1	-4.223679	0.063933	1.154449
1	-4.603345	-0.089720	-0.564499
1	-3.217545	1.793041	-1.124346
1	-3.268222	2.189652	0.612676
6	-2.724232	-2.268777	-0.054937
1	-3.659403	-2.815396	-0.059772
1	-1.804172	-2.836804	-0.124762
8	-0.283470	-0.670037	0.036532
13	1.453410	0.009232	0.003566
17	1.579073	1.125361	-1.813584
17	2.649474	-1.753727	0.018786
17	1.621892	1.189367	1.775558

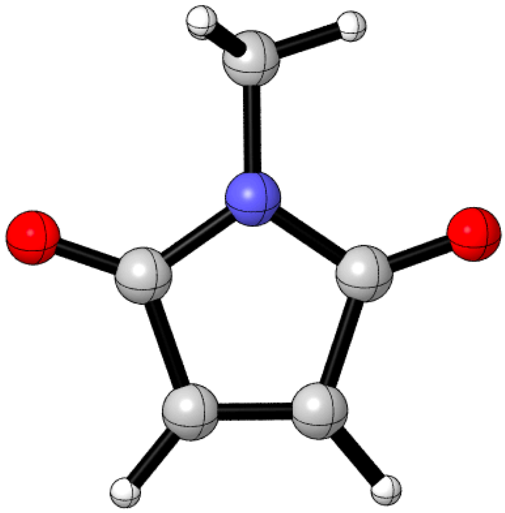

N-methylmaleimide
CBS-QB3 PCM Benzene
Energy (0K) = -398.150341
Enthalpy (298K) = -398.142117
Gibbs free Energy (298K) = -398.182776

6	-0.651168	-1.632863	0.000003
6	0.680693	-1.620321	0.000007
6	-1.144746	-0.212993	0.000003
6	1.149077	-0.192151	0.000006
8	2.285344	0.219663	-0.000014
8	-2.286656	0.183470	-0.000010
1	1.375433	-2.447176	0.000002
7	-0.006163	0.591841	0.000014
6	-0.023333	2.045372	0.000002
1	-0.532397	2.422258	0.888655
1	1.010879	2.385516	-0.000704
1	-0.533621	2.422178	-0.887976
1	-1.329792	-2.472992	-0.000012

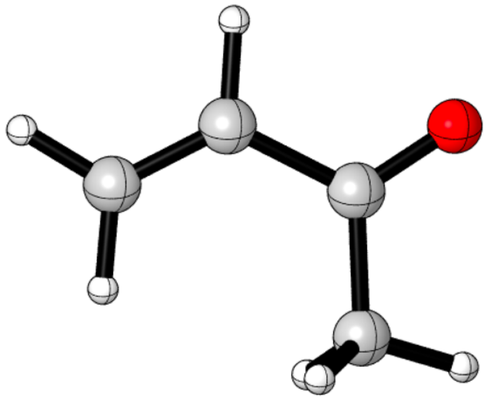

s- trans methyl vinyl ketone
CBS-QB3 PCM Benzene
Energy (0K) = -230.837849
Enthalpy (298K) = -230.831085
Gibbs free Energy (298K) = -230.866728

6	1.939238	0.175078	0.000002
6	0.876937	-0.631447	-0.000001
1	2.947445	-0.223366	-0.000007
1	1.847163	1.255492	0.000003
6	-0.543864	-0.188851	0.000005
8	-1.429371	-1.025279	-0.000001
6	-0.861579	1.294183	-0.000003
1	-0.438118	1.783157	0.881727
1	-0.438030	1.783184	-0.881674
1	-1.942892	1.420932	-0.000052
1	0.995018	-1.710938	-0.000009

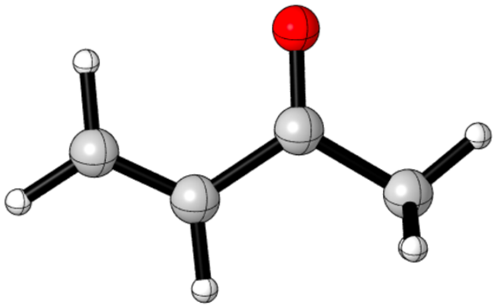

s-cis methyl vinyl ketone
CBS-QB3 PCM Benzene
Energy (0K) = -230.837123
Enthalpy (298K) = -230.830301
Gibbs free Energy (298K) = -230.866150

6	-2.012453	-0.102633	-0.000069
6	-0.803777	-0.663248	0.000095
1	-2.920512	-0.693867	-0.000021
1	-2.108218	0.977931	-0.000275
6	0.442786	0.165008	-0.000041
8	0.406420	1.380570	0.000061
6	1.750409	-0.597351	-0.000129
1	1.808117	-1.244978	0.880910
1	1.806424	-1.248956	-0.878261

1	2.588483	0.097702	-0.002257
1	-0.687434	-1.743042	0.000277

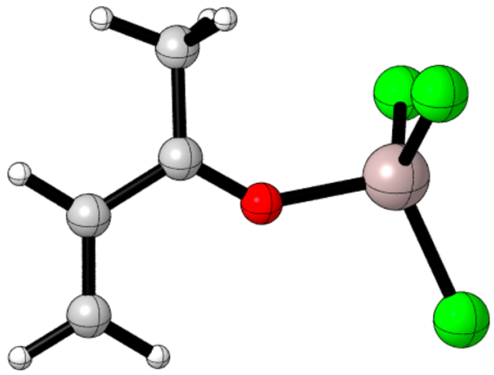

cis-anti-methyl vinyl ketone $AlCl_3$ complex
CBS-QB3 PCM Benzene
Energy (0K) = -1852.374875
Enthalpy (298K) = -1852.362147
Gibbs free Energy (298K) = -1852.415228

6	3.253680	0.269382	0.000050
6	3.359180	1.605191	0.000257
1	4.130674	-0.365822	-0.000005
1	4.327081	2.091291	0.000284
1	2.476600	2.234431	0.000352
6	1.955684	-0.397029	-0.000103
6	1.877045	-1.884614	0.000236
1	2.859166	-2.351641	0.002368
1	1.305826	-2.210928	0.875680
1	1.309795	-2.211102	-0.877770
8	0.912410	0.300749	0.000161
13	-0.935308	0.094217	-0.000008
17	-1.350615	-0.997378	-1.790587
17	-1.664345	2.091612	0.000612
17	-1.351096	-0.998624	1.789698

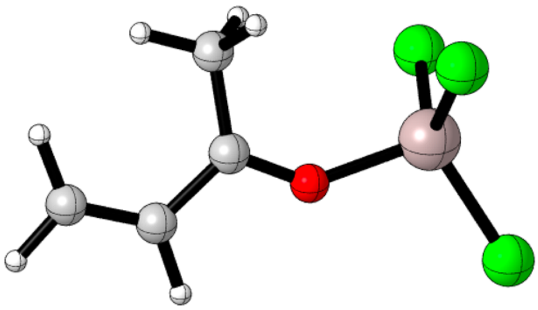

s-trans anti methyl vinyl ketone $AlCl_3$ complex
CBS-QB3 PCM Benzene
Energy (0K) = -1852.374512
Enthalpy (298K) = -1852.361004
Gibbs free Energy (298K) = -1852.416581

6	3.008337	1.008013	0.000002
6	4.300604	0.657051	0.000000
1	2.712282	2.051124	0.000004

1	5.074844	1.414884	0.000000
1	4.633955	-0.373201	-0.000001
6	1.895986	0.068296	0.000002
6	2.054501	-1.413810	0.000000
1	1.540211	-1.817620	-0.877627
1	3.089103	-1.743094	0.000004
1	1.540203	-1.817624	0.877621
8	0.747899	0.577407	0.000002
13	-1.041401	0.056707	0.000000
17	-1.256228	-1.092760	-1.790066
17	-2.110608	1.892101	0.000002
17	-1.256231	-1.092766	1.790062

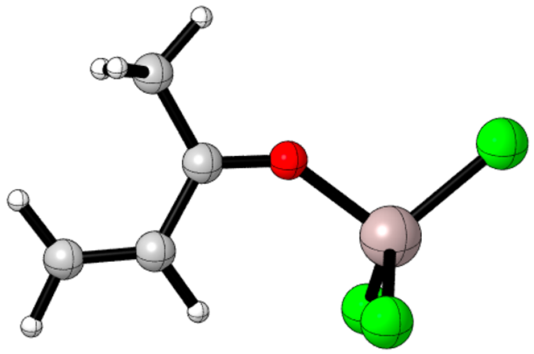

trans-syn-methyl vinyl ketone $AlCl_3$ complex
CBS-QB3 PCM Benzene
Energy (0K) = -1852.373909
Enthalpy (298K) = -1852.360381
Gibbs free Energy (298K) = -1852.415748

6	-2.386801	-0.635322	0.004732
6	-3.700254	-0.894413	-0.004301
1	-1.665651	-1.443886	0.022405
1	-4.060989	-1.915779	0.008088
1	-4.451980	-0.114181	-0.024324
6	-1.838096	0.715368	-0.006990
6	-2.705148	1.934145	-0.005905
1	-3.271572	1.982186	0.928897
1	-2.083357	2.821429	-0.097328
1	-3.425497	1.898451	-0.826100
8	-0.598289	0.892233	-0.008237
13	1.013694	-0.052043	-0.000005
17	0.942733	-1.196554	1.802832
17	2.493659	1.471807	-0.021382
17	0.937086	-1.240441	-1.773855

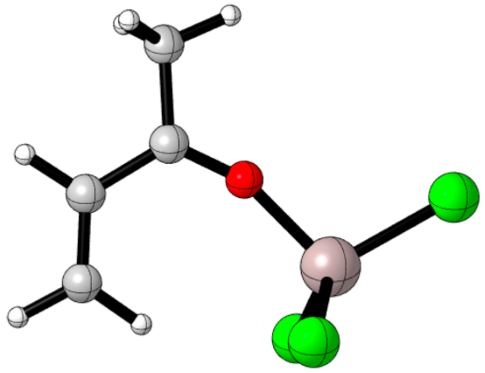

cis-syn-methyl vinyl ketone $AlCl_3$ complex
CBS-QB3 PCM Benzene
Energy (0K) = -1852.371672
Enthalpy (298K) = -1852.357935
Gibbs free Energy (298K) = -1852.414827

6	2.884832	-0.453861	0.000136
6	2.410019	-1.706355	-0.000531
1	3.952890	-0.268608	0.000582
1	3.085725	-2.553249	-0.000572
1	1.350214	-1.927206	-0.001116
6	2.035244	0.732333	0.000099
6	2.693847	2.071326	-0.000042
1	3.343205	2.155968	0.877653
1	3.343351	2.155719	-0.877653
1	1.954832	2.868553	-0.000206
8	0.788354	0.679431	0.000211
13	-0.915473	-0.013039	0.000120
17	-1.001558	-1.178026	-1.789500
17	-2.207760	1.676717	0.000148
17	-1.001244	-1.178560	1.789358

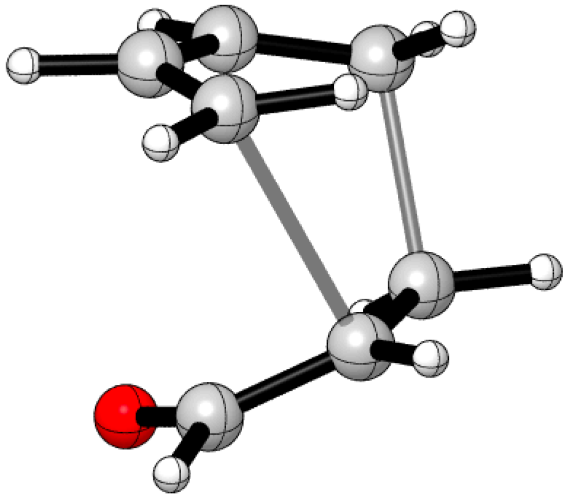

endo s-cis-acrolein butadiene transition structure
CBS-QB3 PCM Benzene
Energy (0K) = -347.243142
Enthalpy (298K) = -347.233894

Gibbs free energy (298K) = -347.275911

6	-1.610645	1.099859	-0.111742
6	-1.564199	-0.091124	-0.833058
6	-1.121701	-1.301628	-0.267137
6	-0.707388	-1.409203	1.032842
6	1.023404	0.562090	0.870829
6	0.312489	1.609869	0.281588
6	1.812244	-0.332116	0.042989
8	1.833393	-0.298872	-1.181326
1	-1.868811	1.065290	0.940163
1	-1.954414	1.994588	-0.619302
1	-1.682136	-0.052311	-1.911097
1	-0.945760	-2.135328	-0.939185
1	-0.246341	-2.321245	1.393447
1	-0.989547	-0.692106	1.789694
1	1.120987	0.479700	1.946833
1	0.037816	2.457133	0.899966
1	0.574442	1.846273	-0.742234
1	2.421392	-1.077493	0.594469

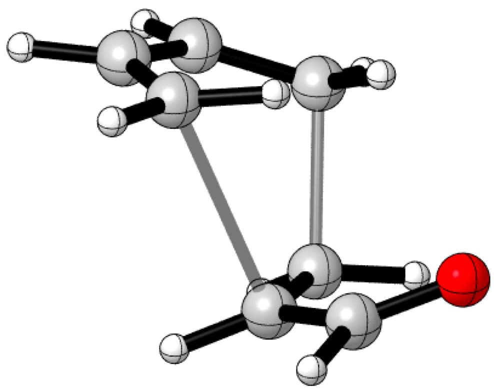

exo s-cis-acrolein butadiene transition structure
CBS-QB3 PCM Benzene
Energy (0K) = -347.241063
Enthalpy (298K) = -347.231715
Gibbs free energy (298K) = -347.274123

6	-1.069461	-1.215435	-0.699186
6	-2.067856	-0.406431	-0.161847
6	-1.870245	0.962432	0.102978
6	-0.727564	1.632524	-0.243174
6	0.903898	-0.052545	0.985082
6	0.358516	-1.312191	0.716795
6	1.982623	0.477534	0.173476
8	2.365682	-0.008872	-0.884451
1	-0.367310	-0.801763	-1.414666
1	-1.293764	-2.264691	-0.859592
1	-2.951326	-0.880832	0.255138
1	-2.600722	1.466014	0.729837
1	-0.557831	2.647608	0.096584
1	-0.070435	1.278108	-1.023596
1	0.641898	0.488982	1.884992

1	0.957433	-1.970233	0.096116
1	-0.202169	-1.807238	1.499846
1	2.459293	1.399690	0.566199

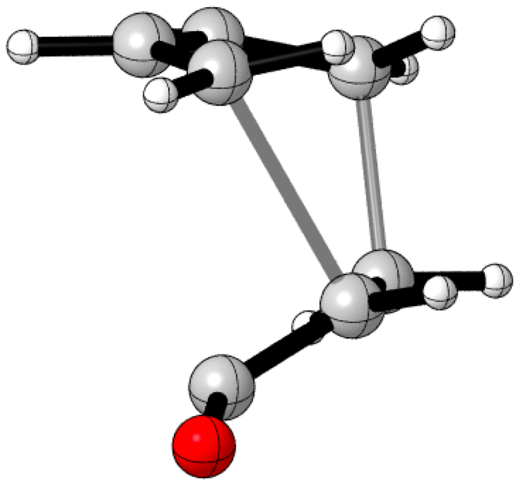

endo s-trans-acrolein butadiene transition structure
CBS-QB3 PCM Benzene
Energy (0K) = -347.241034
Enthalpy (298K) = -347.231737;
Gibbs free energy (298K) = -347.273799

6	2.014915	-0.780115	0.234958
6	1.997724	0.381926	-0.531643
6	1.106127	1.436484	-0.273991
6	0.237011	1.429460	0.788732
6	-0.863599	-0.812323	0.418926
6	0.166290	-1.616978	-0.076589
6	-1.799799	-0.130458	-0.465988
8	-2.890953	0.304980	-0.140410
1	1.840732	-0.720932	1.302501
1	2.687456	-1.580184	-0.055662
1	2.518644	0.380608	-1.484557
1	0.984244	2.196428	-1.040120
1	-0.549083	2.170304	0.868604
1	0.435326	0.860021	1.685220
1	-1.168586	-0.894443	1.457213
1	0.443682	-2.495228	0.495439
1	0.235413	-1.749302	-1.150977
1	-1.452217	-0.055094	-1.520817

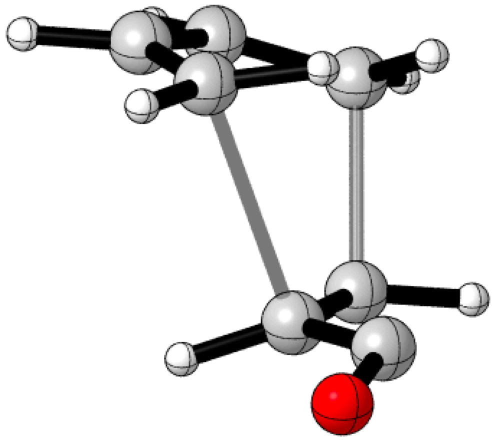

exo s-trans-acrolein butadiene transition structure
CBS-QB3 PCM Benzene
Energy (0K) = -347.239550
Enthalpy (298K) = -347.230220
Gibbs free energy (298K) = -347.272489

6	1.735781	-0.956429	0.627826
6	2.243304	0.115870	-0.100163
6	1.529679	1.315908	-0.257113
6	0.318268	1.539589	0.348704
6	-0.848137	-0.452705	-0.604467
6	0.034373	-1.519398	-0.396308
6	-2.005738	-0.268979	0.260241
8	-2.961844	0.449185	0.027439
1	1.158380	-0.766104	1.524071
1	2.306550	-1.878413	0.660469
1	3.095791	-0.055245	-0.750884
1	1.861525	2.006364	-1.027083
1	-0.294871	2.390370	0.077405
1	0.045834	1.046028	1.270885
1	-0.869361	0.074530	-1.549740
1	-0.239962	-2.256194	0.353506
1	0.555169	-1.930664	-1.251015
1	-1.969487	-0.867288	1.200557

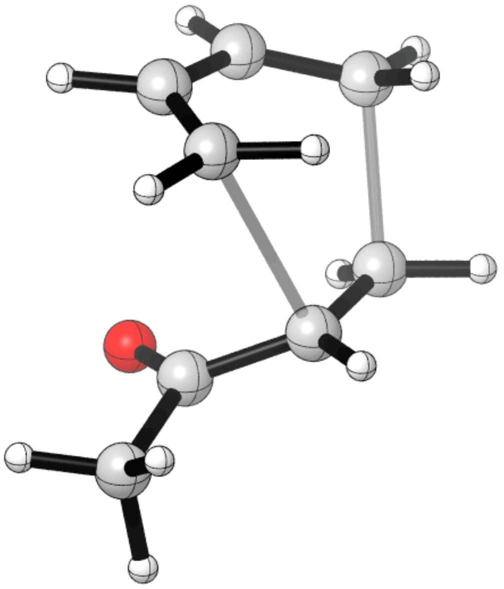

endo s-cis methyl vinyl ketone butadiene transition structure
CBS-QB3 PCM Benzene
Energy (0K) = -386.479997
Enthalpy (298K) = -386.469128
Gibbs free Energy (298K) = -386.515018

6	-2.245120	-0.640932	-0.136010
6	-1.956854	0.421220	0.717779
6	-1.117265	1.484366	0.338258
6	-0.526865	1.560852	-0.895059
6	0.546350	-0.806282	-0.786696
6	-0.502823	-1.644285	-0.403012
6	1.463576	-0.289468	0.229159
8	1.238078	-0.429418	1.428606
6	2.739115	0.391199	-0.242383
1	-2.350754	-0.446953	-1.196882
1	-2.886389	-1.435527	0.229979
1	-2.215435	0.331919	1.767850
1	-0.794640	2.172079	1.113281
1	0.217121	2.320291	-1.105047
1	-0.914680	1.031829	-1.753211
1	0.799855	-0.678550	-1.831983
1	-0.921570	-2.312225	-1.147710
1	-0.451775	-2.039250	0.604046
1	3.538145	-0.356625	-0.289703
1	3.037989	1.150346	0.481469
1	2.636819	0.838001	-1.233148

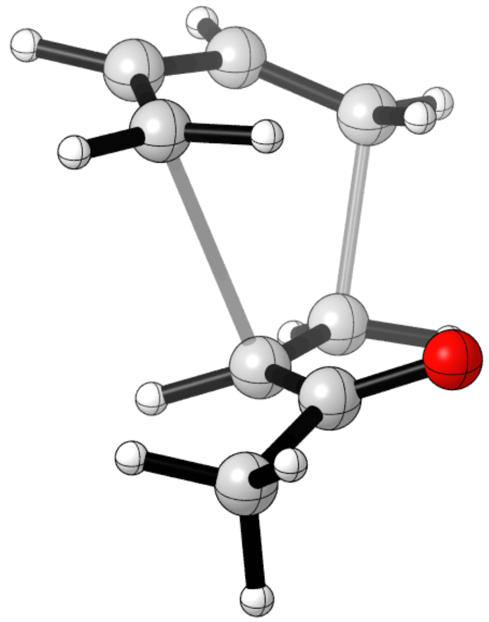

exo s-cis methyl vinyl ketone butadiene transition structure
CBS-QB3 PCM Benzene
Energy (0K) = -386.478744
Enthalpy (298K) = -386.467712
Gibbs free Energy (298K) = -386.514479

6	-1.810360	-1.062791	-0.506757
6	-2.505886	0.046306	-0.032044
6	-1.921271	1.324146	0.039094
6	-0.667603	1.595818	-0.443516
6	0.557019	-0.278925	0.878244
6	-0.321762	-1.364417	0.827073
6	1.681322	-0.197469	-0.055856
8	1.739815	-0.898666	-1.063336
6	2.798996	0.778137	0.273325
1	-1.089175	-0.947102	-1.308056
1	-2.315405	-2.023088	-0.504698
1	-3.443798	-0.113162	0.491894
1	-2.429012	2.081123	0.630356
1	-0.199670	2.554302	-0.250828
1	-0.208937	0.990410	-1.211432
1	0.535815	0.404429	1.716874
1	0.019881	-2.232806	0.274165
1	-0.919841	-1.582365	1.703087
1	3.497113	0.295516	0.966165
1	2.428253	1.683911	0.757632
1	3.343534	1.033338	-0.635850

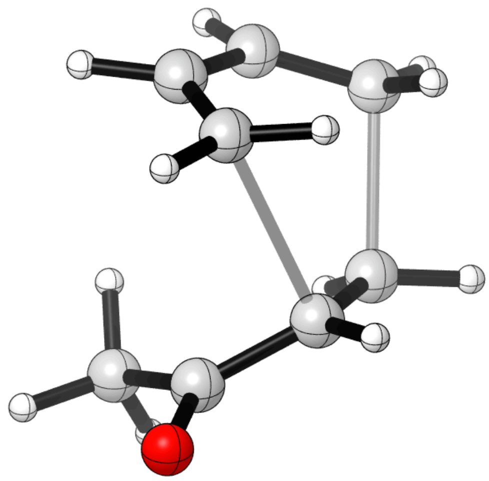

endo s-trans methyl vinyl ketone butadiene transition structure
CBS-QB3 PCM Benzene
Energy (0K) = -386.477415
Enthalpy (298K) = -386.466675
Gibbs free Energy (298K) = -386.512054

6	-2.257279	-0.772594	-0.144900
6	-2.100213	0.328811	0.690417
6	-1.277606	1.413682	0.343752
6	-0.606636	1.477442	-0.854018
6	0.572317	-0.693889	-0.808447
6	-0.359217	-1.605054	-0.302649
6	1.678443	-0.106529	-0.032955
8	2.634212	0.411725	-0.595412
6	1.637011	-0.206576	1.484117
1	-2.291101	-0.626987	-1.217671
1	-2.850546	-1.610933	0.204417
1	-2.437251	0.253792	1.720193
1	-1.024850	2.129212	1.120634
1	0.146080	2.236301	-1.029152
1	-0.970310	0.970289	-1.735746
1	0.721213	-0.631348	-1.881020
1	-0.730984	-2.368348	-0.976932
1	-0.271246	-1.931266	0.725906
1	1.889363	-1.225905	1.794241
1	0.647661	0.024120	1.885895
1	2.377349	0.475510	1.900628

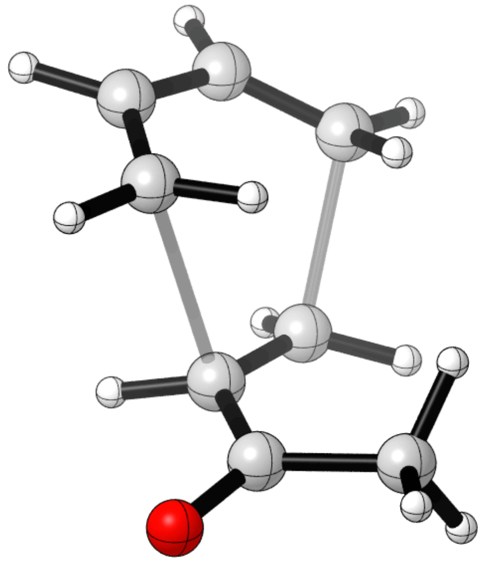

exo s-trans methyl vinyl ketone butadiene transition structure
CBS-QB3 PCM Benzene
Energy (0K) = -386.475587
Enthalpy (298K) = -386.464676
Gibbs free Energy (298K) = -386.510788

6	-1.837321	-1.190122	-0.426740
6	-2.545044	-0.055785	-0.043079
6	-1.981695	1.229473	-0.105161
6	-0.724798	1.460692	-0.609523
6	0.517319	-0.065327	0.875502
6	-0.217035	-1.255499	0.884307
6	1.785872	0.121318	0.151019
8	2.528128	1.057454	0.414433
6	2.182082	-0.888274	-0.919280
1	-1.162152	-1.138405	-1.272182
1	-2.296052	-2.163408	-0.288445
1	-3.457944	-0.182833	0.531377
1	-2.481253	2.029287	0.433954
1	-0.244182	2.424015	-0.490522
1	-0.291602	0.814568	-1.359751
1	0.359827	0.664245	1.659385
1	0.200101	-2.131389	0.400324
1	-0.809442	-1.478190	1.762248
1	2.536449	-1.810347	-0.447644
1	2.994465	-0.467987	-1.510672
1	1.350495	-1.158044	-1.575807

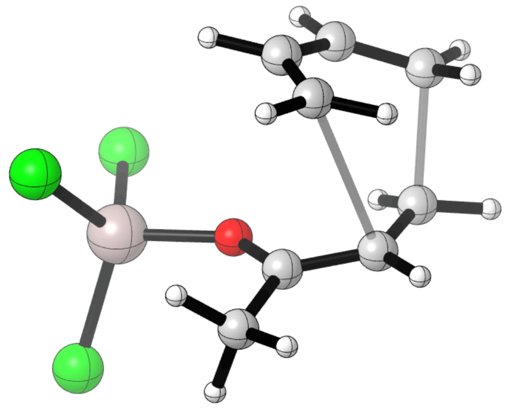

endo s-cis anti methyl vinyl ketone $AlCl_3$ complex butadiene transition structure
CBS-QB3 PCM Benzene
Energy (0K) = -2008.036741
Enthalpy (298K) = -2008.018959
Gibbs free Energy (298K) = -2008.083740

6	3.526859	1.131631	-0.679045
6	2.623609	1.514753	0.297560
6	2.283107	0.731204	1.436211
6	2.797439	-0.497511	1.691463
6	2.085912	-1.341166	-1.057668
6	2.483551	-0.237373	-1.818435
6	0.814640	-1.385836	-0.451465
6	0.305642	-2.617555	0.231778
1	4.320203	0.434742	-0.437911
1	3.761568	1.836581	-1.467730
1	2.035460	2.410970	0.126867
1	1.481666	1.101041	2.066348
1	2.426722	-1.090351	2.518936
1	3.640596	-0.903229	1.148821
1	2.734303	-2.200741	-0.950524
1	3.327039	-0.356782	-2.488171
1	1.715314	0.447283	-2.151411
1	-0.522818	-3.027192	-0.355463
1	-0.096718	-2.365784	1.215588
1	1.080198	-3.376666	0.332293
8	0.053042	-0.353402	-0.544824
13	-1.576368	0.238732	-0.031912
17	-3.037504	-1.033350	-0.950836
17	-1.651870	2.260079	-0.736781
17	-1.625899	0.127679	2.117818

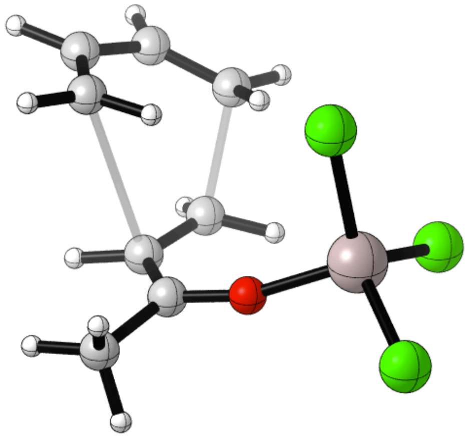

exo s-cis syn methyl vinyl ketone $AlCl_3$ complex butadiene transition structure
CBS-QB3 PCM Benzene
Energy (0K) = -2008.030979
Enthalpy (298K) = -2008.013096
Gibbs free Energy (298K) = -2008.078777

6	2.894690	-1.591950	-0.300492
6	4.011035	-0.967488	0.230126
6	3.970834	0.188250	1.059833
6	2.826289	0.754914	1.517105
6	1.932457	0.907805	-1.335998
6	2.320912	-0.355640	-1.806424
6	0.661797	1.133975	-0.779942
6	0.182068	2.513457	-0.452160
1	1.935399	-1.516171	0.198080
1	3.040709	-2.505038	-0.865673
1	4.988578	-1.290530	-0.116437
1	4.915973	0.680784	1.265690
1	2.850957	1.687739	2.067544
1	1.865082	0.263988	1.448403
1	2.599101	1.754099	-1.427805
1	1.531218	-1.056378	-2.052338
1	3.199548	-0.415172	-2.435678
1	-0.146976	2.560209	0.589577
1	-0.693152	2.739221	-1.069552
1	0.950650	3.263845	-0.629953
8	-0.113787	0.128279	-0.563826
13	-1.752954	-0.246004	0.108559
17	-1.923959	-2.371797	-0.072651
17	-3.196010	0.820122	-1.066653
17	-1.711729	0.404927	2.159022

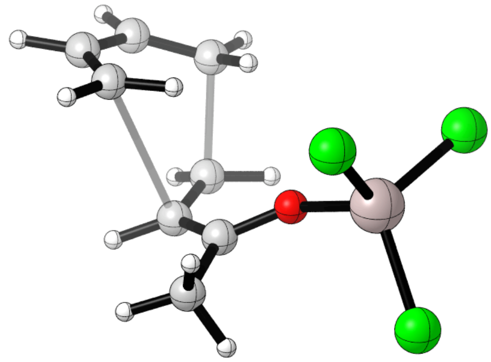

exo s-cis anti methyl vinyl ketone $AlCl_3$ complex butadiene transition structure

CBS-QB3 PCM Benzene

Energy (0K) = -2008.030975

Enthalpy (298K) = -2008.013092

Gibbs free Energy (298K) = -2008.078777

6	-2.894233	1.591976	-0.300314
6	-4.010845	0.967741	0.230014
6	-3.971149	-0.188157	1.059528
6	-2.826906	-0.755205	1.517061
6	-1.932409	-0.907660	-1.336202
6	-2.320819	0.355873	-1.806452
6	-0.661733	-1.133993	-0.780271
6	-0.182233	-2.513527	-0.452352
1	-1.935038	1.515804	0.198380
1	-3.039904	2.505255	-0.865273
1	-4.988235	1.291123	-0.116667
1	-4.916478	-0.680470	1.265044
1	-2.852020	-1.688129	2.067316
1	-1.865519	-0.264571	1.448754
1	-2.599167	-1.753872	-1.427933
1	-1.531093	1.056553	-2.052434
1	-3.199528	0.415541	-2.435590
1	0.146944	-2.560186	0.589349
1	0.692862	-2.739551	-1.069825
1	-0.950986	-3.263794	-0.629917
8	0.114162	-0.128474	-0.564461
13	1.753004	0.245950	0.108546
17	1.710760	-0.404159	2.159260
17	1.924327	2.371666	-0.073321
17	3.196546	-0.820774	-1.065509

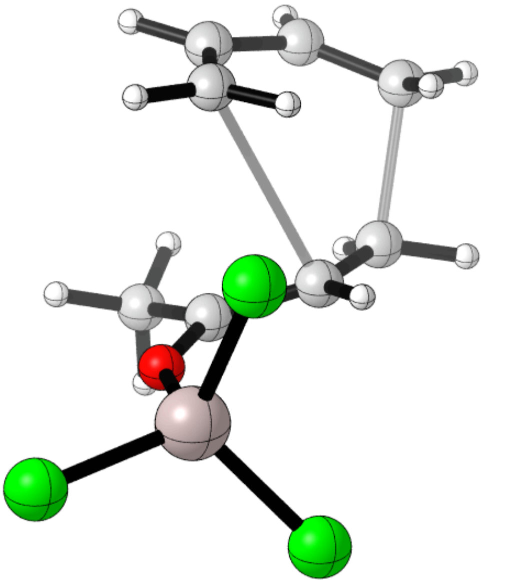

endo s-trans syn methyl vinyl ketone $AlCl_3$ complex butadiene transition structure
CBS-QB3 PCM Benzene
Energy (0K) = -2008.029865
Enthalpy (298K) = -2008.012101
Gibbs free Energy (298K) = -2008.077254

6	3.992231	-0.396910	1.069861
6	4.241646	0.474370	0.017760
6	3.351321	1.492467	-0.408041
6	2.164057	1.761278	0.199108
6	1.337237	-1.002070	0.225225
6	2.577369	-1.647546	0.322629
6	0.733642	-0.579238	-0.979006
6	1.402202	-0.686388	-2.318317
1	3.376099	-0.077027	1.901209
1	4.763880	-1.111476	1.331826
1	5.096204	0.267508	-0.620288
1	3.590299	2.004047	-1.334926
1	1.461087	2.461916	-0.234503
1	1.903127	1.381208	1.176414
1	0.741746	-0.894888	1.124836
1	2.713847	-2.306349	1.172852
1	3.078701	-1.985353	-0.574728
1	1.069417	0.136876	-2.950198
1	1.078304	-1.618287	-2.794154
1	2.489038	-0.690943	-2.256777
8	-0.479308	-0.159674	-1.013635
13	-1.912198	0.052733	0.072766
17	-2.423313	-1.903320	0.795541
17	-3.423507	0.932901	-1.153352
17	-1.298294	1.354470	1.678180

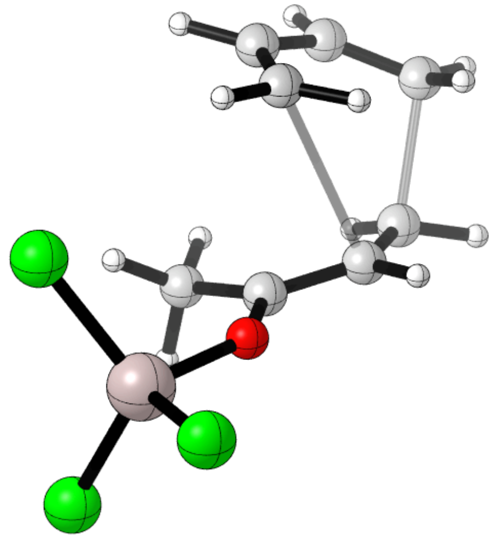

endo s-trans anti methyl vinyl ketone $AlCl_3$ complex butadiene transition structure
CBS-QB3 PCM Benzene
Energy (0K) = -2008.029537
Enthalpy (298K) = -2008.011803
Gibbs free Energy (298K) = -2008.076802

6	4.532165	-0.183028	0.632168
6	4.253137	0.596542	-0.483867
6	3.139502	1.465127	-0.593238
6	2.241919	1.673891	0.408882
6	1.859105	-1.111807	0.929343
6	3.111098	-1.629132	0.571842
6	0.786631	-0.849438	0.047857
6	0.867023	-1.033299	-1.437984
1	4.243276	0.163404	1.616948
1	5.431616	-0.787740	0.616605
1	4.823612	0.408843	-1.389035
1	2.934706	1.890884	-1.570169
1	1.333152	2.237043	0.233165
1	2.435671	1.385439	1.432606
1	1.630303	-0.966822	1.979562
1	3.641753	-2.185742	1.336137
1	3.268623	-2.008116	-0.429323
1	0.385483	-0.192342	-1.940228
1	0.306299	-1.934208	-1.707429
1	1.887729	-1.133913	-1.800556
8	-0.341515	-0.522089	0.567248
13	-1.985869	0.047574	0.077763
17	-1.646059	1.851509	-1.055246
17	-3.026047	0.415600	1.907339
17	-2.887734	-1.496027	-1.109576

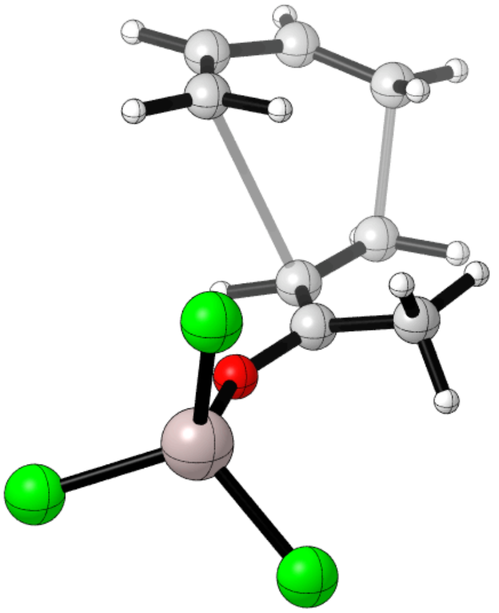

exo s-trans anti methyl vinyl ketone $AlCl_3$ complex butadiene transition structure
CBS-QB3 PCM Benzene
Energy (0K) = -2008.024007
Enthalpy (298K) = -2008.005958
Gibbs free Energy (298K) = -2008.073422

6	-4.363567	0.645739	0.381328
6	-4.633569	-0.633304	-0.092503
6	-3.723395	-1.718316	-0.016653
6	-2.522238	-1.652603	0.615805
6	-1.872350	0.641723	-1.042297
6	-3.052347	1.400780	-0.931055
6	-0.702124	0.835947	-0.282445
6	-0.600429	1.790132	0.869304
1	-3.709423	0.765666	1.236644
1	-5.152981	1.386159	0.317231
1	-5.516550	-0.771779	-0.709672
1	-3.961273	-2.607326	-0.591766
1	-1.799471	-2.454560	0.530230
1	-2.271020	-0.858454	1.305358
1	-1.787073	-0.096431	-1.829685
1	-3.014385	2.350167	-0.408906
1	-3.700903	1.415767	-1.797828
1	0.154587	2.547425	0.638603
1	-0.243410	1.250000	1.750890
1	-1.538787	2.285736	1.107980
8	0.343125	0.166429	-0.609868
13	2.038987	-0.136865	-0.055226
17	3.075201	1.743070	-0.105174
17	2.832648	-1.569898	-1.426759
17	1.863267	-0.915828	1.945986

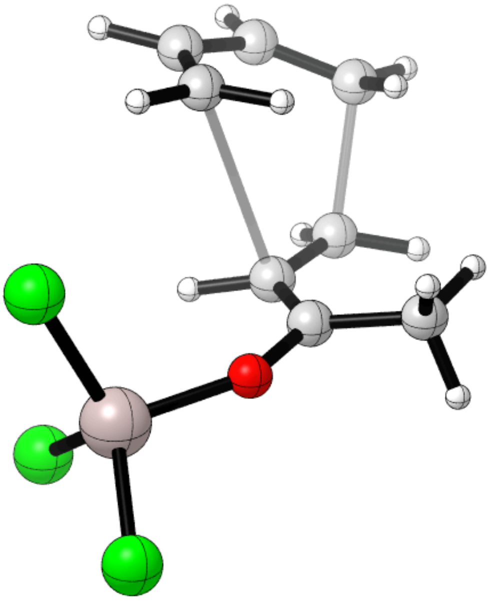

exo s-trans syn methyl vinyl ketone $AlCl_3$ complex butadiene transition structure
CBS-QB3 PCM Benzene
Energy (0K) = -2008.023830
Enthalpy (298K) = -2008.006812
Gibbs free Energy (298K) = -2008.068988

6	-4.234229	-0.392738	-0.000400
6	-4.105942	0.951080	0.325620
6	-3.094155	1.806715	-0.184822
6	-2.192389	1.430890	-1.127863
6	-1.458394	-0.803456	0.651876
6	-2.733136	-1.283941	1.005528
6	-0.635778	-1.364534	-0.341855
6	-1.075125	-2.451087	-1.279823
1	-3.903430	-0.738198	-0.972505
1	-5.081431	-0.934956	0.403904
1	-4.708505	1.334467	1.144061
1	-2.980036	2.776964	0.287351
1	-1.353646	2.064321	-1.387465
1	-2.309878	0.528398	-1.712610
1	-1.012831	-0.020033	1.250949
1	-3.037790	-2.265471	0.661002
1	-3.076558	-1.054166	2.006081
1	-0.476823	-3.345532	-1.084285
1	-0.852460	-2.139536	-2.303205
1	-2.129627	-2.705896	-1.198995
8	0.582469	-0.994739	-0.501324
13	1.887232	0.117480	0.060128
17	1.603743	1.936266	-1.055379
17	3.717860	-0.875080	-0.423824
17	1.672758	0.443091	2.175972

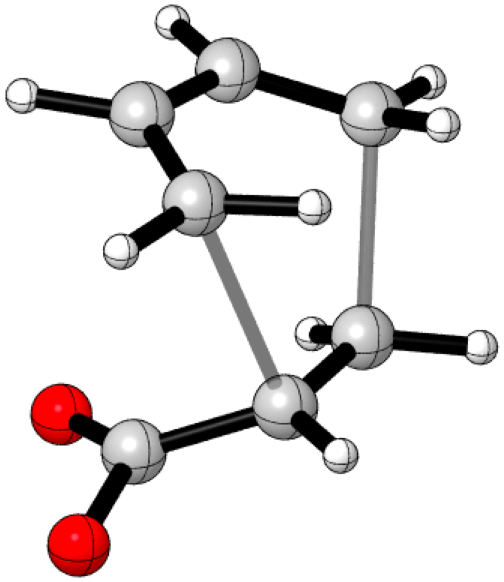

endo s-cis-acrylic acid butadiene transition structure

CBS-QB3 PCM Benzene

Energy (0K) = -422.424547

Enthalpy (298K) = -422.414503;

Gibbs free energy (298K) = -422.458553

6	-2.259485	-0.678008	-0.095893
6	-1.991195	0.415680	0.720524
6	-1.138672	1.457140	0.317085
6	-0.534039	1.478986	-0.914932
6	0.556295	-0.762301	-0.771141
6	-0.444351	-1.629315	-0.333362
6	1.516694	-0.239206	0.196102
8	1.422667	-0.322208	1.406317
8	2.582656	0.370385	-0.396401
1	-2.346988	-0.533180	-1.165962
1	-2.881094	-1.477378	0.292900
1	-2.261333	0.361119	1.770461
1	-0.805997	2.159954	1.074181
1	0.237161	2.205571	-1.140732
1	-0.953118	0.962115	-1.765987
1	0.807756	-0.671922	-1.818796
1	-0.853363	-2.335439	-1.046442
1	-0.372034	-1.984668	0.686360
1	3.154952	0.670565	0.324384

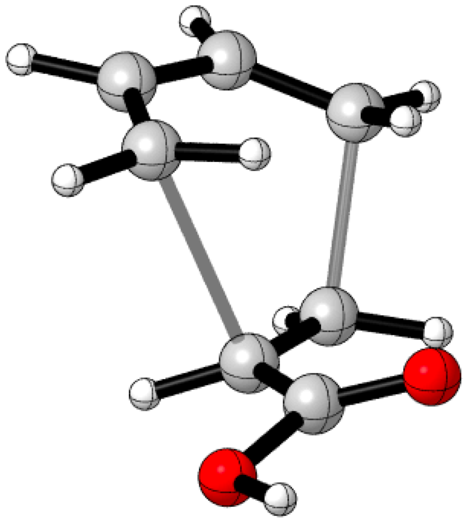

exo s-cis-acrylic acid butadiene transition structure
CBS-QB3 PCM Benzene
Energy (0K) = -422.423703
Enthalpy (298K) = -422.413565;
Gibbs free energy (298K) = -422.458123

6	-1.791754	-1.090617	-0.537275
6	-2.497105	0.010924	-0.062345
6	-1.917877	1.289020	0.024229
6	-0.651911	1.558410	-0.430881
6	0.555544	-0.235387	0.879918
6	-0.278099	-1.352459	0.813797
6	1.705092	-0.144397	-0.013959
8	1.892368	-0.826507	-1.005972
8	2.584134	0.826051	0.357541
1	-1.069678	-0.967810	-1.335990
1	-2.281295	-2.058823	-0.530975
1	-3.435615	-0.157794	0.457682
1	-2.429472	2.038563	0.621364
1	-0.173215	2.505197	-0.210774
1	-0.195232	0.971297	-1.214713
1	0.524220	0.445055	1.717747
1	0.092838	-2.208702	0.261937
1	-0.884336	-1.588504	1.678603
1	3.296418	0.812198	-0.298334

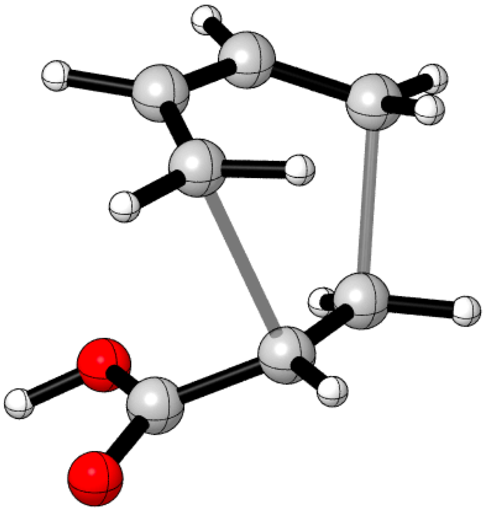

endo s-trans-acrylic acid butadiene transition structure
CBS-QB3 PCM Benzene
Energy (0K) = -422.422709
Enthalpy (298K) = -422.412669;
Gibbs free energy (298K) = -422.456726

6	-2.242073	-0.735590	-0.087604
6	-2.017032	0.374157	0.719105
6	-1.189509	1.433622	0.313017
6	-0.579710	1.460343	-0.917762
6	0.573463	-0.715053	-0.804786
6	-0.373302	-1.614076	-0.310447
6	1.634832	-0.138085	0.020364
8	2.637019	0.407882	-0.397305
8	1.414181	-0.285134	1.359267
1	-2.322129	-0.607775	-1.160291
1	-2.835316	-1.554658	0.304607
1	-2.295150	0.320818	1.767285
1	-0.876438	2.150290	1.065770
1	0.177523	2.200858	-1.145582
1	-0.996564	0.942490	-1.769416
1	0.768489	-0.651634	-1.866922
1	-0.767986	-2.355123	-0.996103
1	-0.273614	-1.945570	0.713860
1	2.171564	0.126427	1.799777

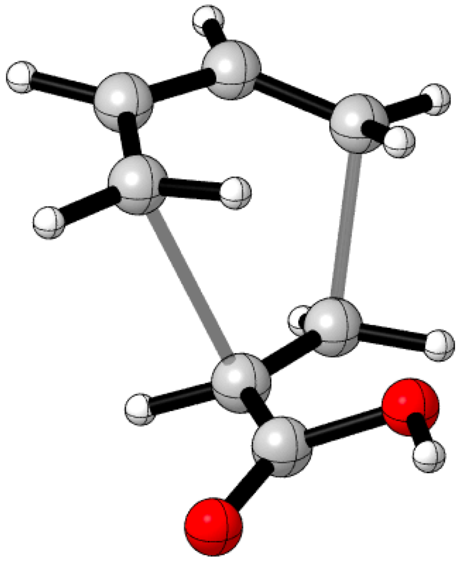

exo s-trans-acrylic acid butadiene transition structure
CBS-QB3 PCM Benzene
Energy (0K) = -422.422320
Enthalpy (298K) = -422.412217;
Gibbs free energy (298K) = -422.456572

6	-1.758591	-1.137275	-0.507622
6	-2.494115	-0.041164	-0.068856
6	-1.946973	1.252359	-0.017177
6	-0.680692	1.535951	-0.465401
6	0.554969	-0.139066	0.910956
6	-0.219769	-1.299879	0.857554
6	1.765864	0.042119	0.113820
8	2.646692	0.848599	0.330994
8	1.825682	-0.799956	-0.963580
1	-1.039675	-1.019875	-1.309214
1	-2.216999	-2.119697	-0.465168
1	-3.428597	-0.218595	0.455509
1	-2.479192	2.006269	0.555841
1	-0.223443	2.497823	-0.266276
1	-0.206555	0.941559	-1.233343
1	0.470887	0.545361	1.742112
1	0.186452	-2.155984	0.333104
1	-0.826718	-1.540787	1.720874
1	2.660697	-0.603490	-1.412390

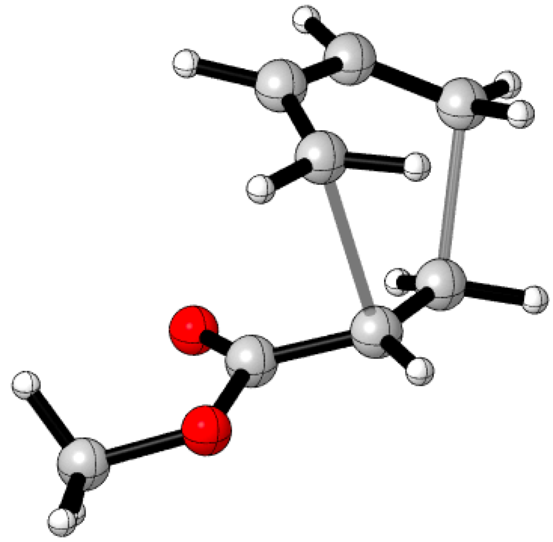

endo s-cis-methyl acrylate butadiene transition structure
CBS-QB3 PCM Benzene
Energy (0K) = -461.635555
Enthalpy (298K) = -461.623796
Gibbs free Energy (298K) = -461.672031

6	-2.737444	-0.453513	0.036493
6	-2.296961	0.643405	0.769359
6	-1.351281	1.547621	0.258342
6	-0.819344	1.422608	-1.001625
6	0.008921	-0.906217	-0.780453
6	-1.057637	-1.626554	-0.243418
6	1.091192	-0.466470	0.101678
8	1.060895	-0.480025	1.317396
8	2.174326	-0.038435	-0.600768
6	3.290955	0.402764	0.186692
1	-2.868728	-0.357883	-1.034548
1	-3.423367	-1.151591	0.504263
1	-2.510836	0.680889	1.832901
1	-0.893475	2.246676	0.950928
1	0.016409	2.037518	-1.313481
1	-1.348669	0.917918	-1.796823
1	0.208088	-0.902110	-1.843184
1	-1.587409	-2.311712	-0.894812
1	-0.969031	-1.938952	0.789076
1	3.641882	-0.395402	0.842966
1	3.018216	1.266721	0.795986
1	4.064748	0.673749	-0.528709

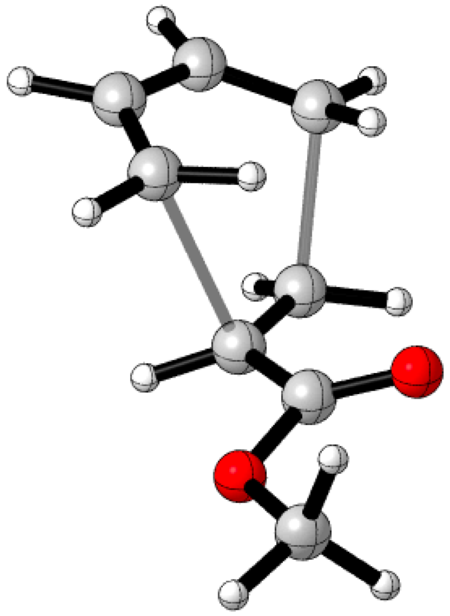

exo s-cis-methyl acrylate butadiene transition structure
CBS-QB3 PCM Benzene
Energy (0K) = -461.634662
Enthalpy (298K) = -461.622877
Gibbs free Energy (298K) = -461.671324

6	-2.324173	-0.916739	-0.619251
6	-2.909464	0.246084	-0.128725
6	-2.183410	1.439269	0.024962
6	-0.873976	1.562304	-0.369130
6	0.058251	-0.375099	0.911101
6	-0.887881	-1.391339	0.775001
6	1.263674	-0.398946	0.081521
8	1.418698	-1.069717	-0.922976
8	2.217567	0.444051	0.552714
6	3.439258	0.490111	-0.202442
1	-1.563065	-0.857845	-1.388279
1	-2.922507	-1.820907	-0.659249
1	-3.880322	0.176137	0.353203
1	-2.623313	2.227164	0.629870
1	-0.294154	2.436488	-0.097513
1	-0.461542	0.952646	-1.160381
1	0.068314	0.274215	1.773783
1	-0.592617	-2.265234	0.205528
1	-1.553710	-1.584052	1.605954
1	3.252191	0.836736	-1.220379
1	3.905057	-0.495810	-0.243561
1	4.081882	1.191924	0.324896

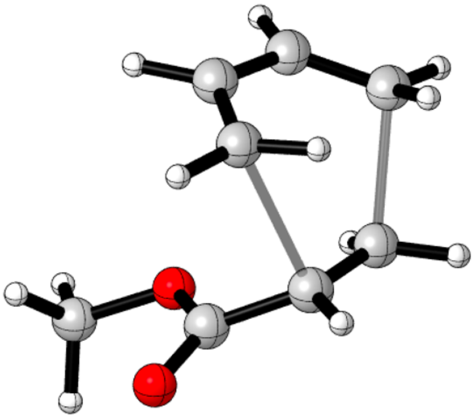

endo s-trans-methyl acrylate butadiene transition structure
CBS-QB3 PCM Benzene
Energy (0K) = -461.633886
Enthalpy (298K) = -461.622169
Gibbs free Energy (298K) = -461.670247

6	2.527057	-0.074528	-0.758327
6	2.045323	1.124952	-0.246626
6	1.324230	1.182363	0.956964
6	1.078251	0.067191	1.721320
6	0.014000	-1.327249	-0.002895
6	0.830066	-1.217440	-1.129815
6	-1.254406	-0.605678	0.146569
8	-2.122482	-0.876997	0.952691
8	-1.378012	0.416532	-0.742238
6	-2.594153	1.174851	-0.653218
1	2.898184	-0.832494	-0.079607
1	3.017433	-0.070800	-1.725901
1	2.023435	1.996346	-0.893994
1	0.788972	2.098926	1.184836
1	0.386624	0.107317	2.554225
1	1.734499	-0.790952	1.701922
1	0.104724	-2.178542	0.658663
1	1.427859	-2.079562	-1.402880
1	0.471883	-0.625175	-1.960222
1	-2.678547	1.656804	0.322590
1	-3.462210	0.531989	-0.806416
1	-2.531107	1.923105	-1.440675

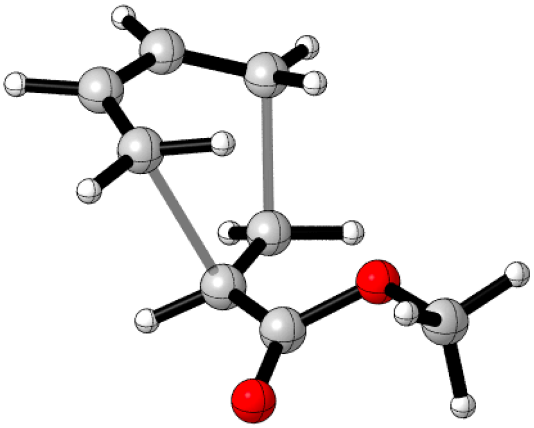

exo s-trans-methyl acrylate butadiene transition structure
CBS-QB3 PCM Benzene
Energy (0K) = -461.633288
Enthalpy (298K) = -461.621532
Gibbs free Energy (298K) = -461.669865

6	-1.923929	-1.339576	-0.375913
6	-2.805741	-0.265777	-0.313491
6	-2.375095	1.062030	-0.473775
6	-1.073502	1.382813	-0.772460
6	0.030708	0.197867	1.106257
6	-0.627850	-1.029260	1.206526
6	1.353425	0.345635	0.492612
8	2.113677	1.273687	0.678092
8	1.653064	-0.686328	-0.347396
6	2.948098	-0.628944	-0.968499
1	-1.088432	-1.314040	-1.064742
1	-2.304747	-2.336225	-0.178901
1	-3.800563	-0.432606	0.089260
1	-3.054215	1.856084	-0.176627
1	-0.731062	2.409958	-0.732225
1	-0.430947	0.694896	-1.303116
1	-0.249600	1.027589	1.738612
1	-0.070858	-1.924285	0.958684
1	-1.355160	-1.148241	1.999376
1	3.736655	-0.634642	-0.214419
1	3.045171	0.271795	-1.576608
1	3.013149	-1.517869	-1.592398

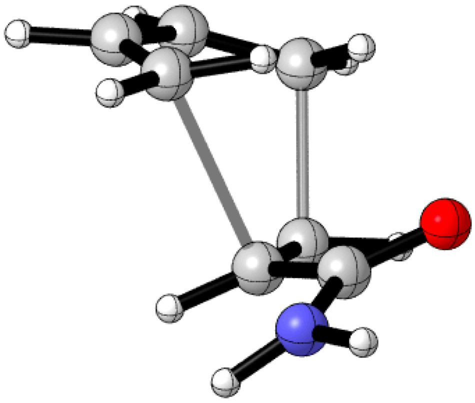

exo s-cis-acrylamide butadiene transition structure

cbsqbe
Energy (0K) = -402.548446
Enthalpy (298K) = -402.537796;
Gibbs free energy (298K) = -402.583041

6	-1.826036	-1.094334	-0.485221
6	-2.519791	0.030288	-0.050820
6	-1.921904	1.300957	-0.010190
6	-0.645472	1.527510	-0.465133
6	0.553592	-0.184797	0.867152
6	-0.282388	-1.300511	0.866779
6	1.716941	-0.160287	-0.044152
8	1.800618	-0.888130	-1.032430
7	2.689964	0.757358	0.251952
1	-1.099688	-1.010816	-1.284589
1	-2.319219	-2.059578	-0.434610
1	-3.459828	-0.108047	0.475665
1	-2.423527	2.081378	0.555577
1	-0.156111	2.477880	-0.285354
1	-0.199881	0.911158	-1.232593
1	0.528950	0.515354	1.692184
1	0.077524	-2.177977	0.342221
1	-0.883364	-1.495208	1.745461
1	3.539556	0.727089	-0.289241
1	2.711237	1.229342	1.140572

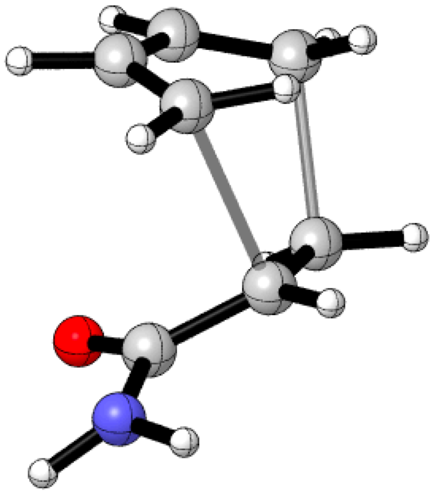

endo s-cis-acrylamide butadiene transition structure
CBS-QB3 PCM Benzene
Energy (0K) = -402.548020
Enthalpy (298K) = -402.537440;
Gibbs free energy (298K) = -402.582369

6	-2.284168	-0.680631	-0.106612
6	-2.017509	0.406550	0.716655
6	-1.161515	1.445582	0.318375
6	-0.551956	1.462446	-0.913329
6	0.555763	-0.709456	-0.776603
6	-0.432138	-1.606803	-0.376342
6	1.531118	-0.230009	0.227329
8	1.349783	-0.349226	1.435746
7	2.656500	0.379024	-0.271300
1	-2.370858	-0.531687	-1.176047
1	-2.893076	-1.492124	0.277335
1	-2.282700	0.343576	1.767328
1	-0.820680	2.140691	1.078745
1	0.218373	2.191554	-1.135178
1	-0.989218	0.968775	-1.769518
1	0.807999	-0.612489	-1.826376
1	-0.833278	-2.293303	-1.112584
1	-0.364761	-1.983722	0.635794
1	3.397005	0.580808	0.381829
1	2.899860	0.302497	-1.245040

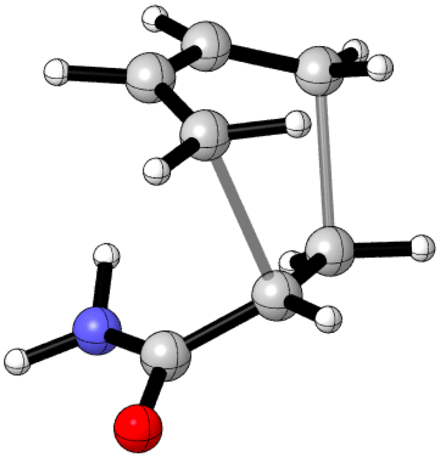

endo s-trans-acrylamide butadiene transition structure
CBS-QB3 PCM Benzene
Energy (0K) = -402.546190
Enthalpy (298K) = -402.535833;
Gibbs free energy (298K) = -402.580303

6	2.267765	0.754153	-0.001114
6	2.033590	-0.430236	0.685705
6	1.208422	-1.439122	0.162268
6	0.592875	-1.321387	-1.064651
6	-0.586919	0.724191	-0.751948
6	0.336967	1.640700	-0.250987
1	2.354067	0.738273	-1.080010
1	2.837442	1.541966	0.479710
1	2.319597	-0.493493	1.732107
1	0.896895	-2.239836	0.826206
1	-0.155664	-2.040035	-1.376323
1	1.030930	-0.735902	-1.860122
1	-0.815143	0.720779	-1.809762
1	0.734282	2.386132	-0.929598
1	0.247063	1.976050	0.774315
6	-1.667975	0.116058	0.064123
8	-2.710579	-0.284259	-0.439737
7	-1.460843	0.075342	1.423947
1	-2.127500	-0.471870	1.947812
1	-0.519782	0.098473	1.785549

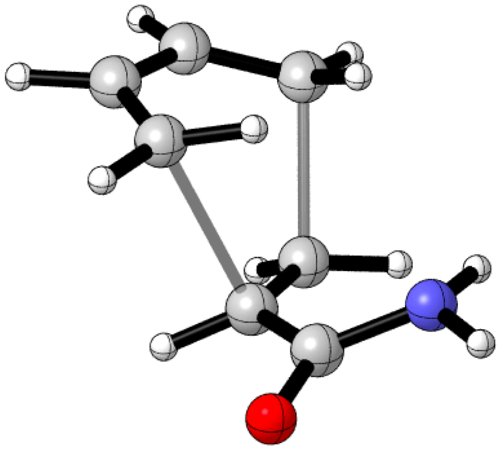

exo s-trans-acrylamide butadiene transition structure
CBS-QB3 PCM Benzene
Energy (0K) = -402.544332
Enthalpy (298K) = -402.533801;
Gibbs free Energy (298K) = -402.578811

6	-1.831196	-1.149275	-0.465011
6	-2.531687	-0.013281	-0.076472
6	-1.940376	1.259999	-0.080994
6	-0.656894	1.473402	-0.527590
6	0.538189	-0.097039	0.878960
6	-0.245226	-1.251221	0.917213
1	-1.117500	-1.085977	-1.277705
1	-2.308714	-2.118587	-0.367028
1	-3.469146	-0.138195	0.457627
1	-2.445591	2.056857	0.457430
1	-0.165138	2.425183	-0.366536
1	-0.207982	0.843897	-1.282695
1	0.453894	0.631847	1.672117
1	0.126763	-2.170508	0.477022
1	-0.864575	-1.414754	1.789357
6	1.796733	0.050492	0.110112
8	2.606062	0.935883	0.362149
7	1.995851	-0.812426	-0.944625
1	2.911944	-0.789115	-1.366056
1	1.489321	-1.679190	-1.015663

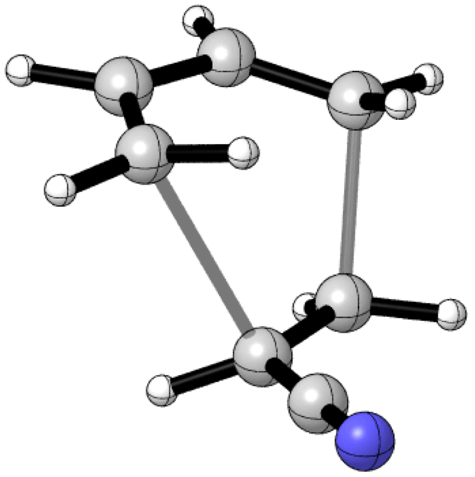

exo acrylonitrile butadiene transition structure
CBS-QB3 PCM Benzene
Energy (0K) = -326.179628
Enthalpy (298K) = -326.170553;
Gibbs free Energy (298K) = -326.212212

6	-1.339452	-1.073265	-0.779268
6	-2.144292	-0.149837	-0.116124
6	-1.693778	1.141298	0.207384
6	-0.461500	1.612330	-0.170044
6	0.929372	-0.270125	0.797636
6	0.194659	-1.430452	0.518504

6	2.090839	0.055690	0.053791
7	3.033194	0.323765	-0.566240
1	-0.658484	-0.733397	-1.550762
1	-1.732884	-2.069648	-0.951309
1	-3.053311	-0.503979	0.361081
1	-2.276000	1.716407	0.921659
1	-0.079312	2.544939	0.228438
1	0.059438	1.228572	-1.034735
1	0.805070	0.252003	1.735555
1	0.643134	-2.165327	-0.140183
1	-0.395092	-1.849760	1.322664

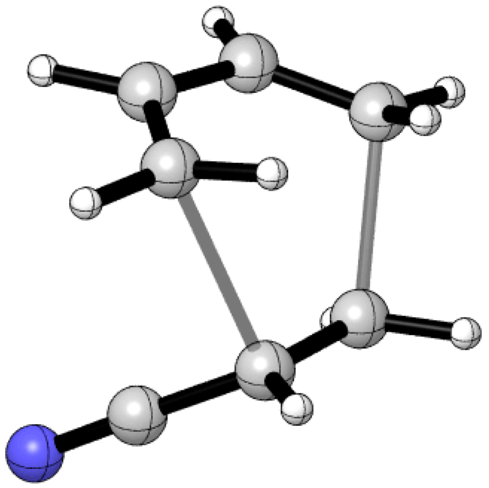

endo acrylonitrile butadiene transition structure
CBS-QB3 PCM Benzene
Energy (0K) = -326.179360
Enthalpy (298K) = -326.170274;
Gibbs free Energy (298K) = -326.211981

6	-1.887454	0.828560	-0.123286
6	-1.703440	-0.383474	-0.779722
6	-0.953404	-1.427511	-0.214029
6	-0.389229	-1.336392	1.035347
6	0.917508	0.736621	0.655065
6	0.015039	1.632062	0.068606
6	1.913892	0.090024	-0.122902
7	2.735497	-0.416607	-0.764252
1	-2.011731	0.836314	0.952810
1	-2.418904	1.624496	-0.633860
1	-1.947787	-0.447630	-1.835709
1	-0.658953	-2.249411	-0.858954
1	0.310346	-2.088742	1.379684
1	-0.799580	-0.692330	1.799971
1	1.103365	0.769346	1.720898
1	-0.350258	2.443531	0.686337
1	0.147548	1.881338	-0.975889

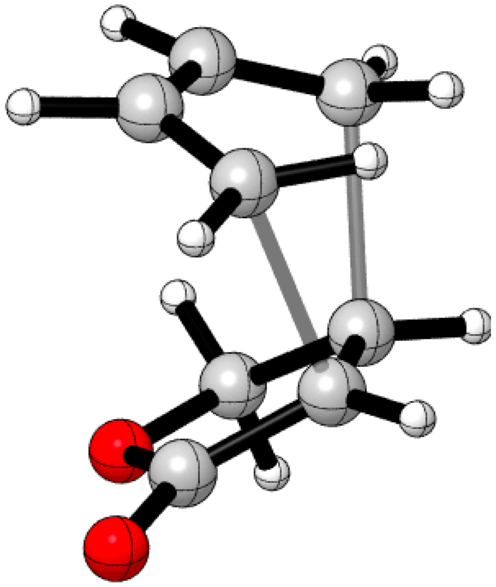

endo furan-2(5H)-one butadiene transition structure
CBS-QB3 PCM Benzene
Energy (0K) = -460.453866
Enthalpy (298K) = -460.444088
Gibbs free Energy (298K) = -460.487642

1	-2.494905	-1.789123	0.259872
6	-2.084651	-0.789060	0.356503
6	-2.008481	0.017024	-0.773426
1	-2.239042	-0.341023	1.330474
6	-0.070038	-1.140389	0.875271
6	-1.394340	1.278686	-0.744118
1	-2.219221	-0.421937	-1.743805
6	0.579627	0.073705	1.100241
1	-0.387834	-1.782451	1.686224
6	-0.890728	1.819482	0.417382
1	-1.136264	1.741925	-1.691261
6	1.566009	0.298433	0.030627
1	0.678796	0.577177	2.048761
1	-0.267744	2.705056	0.388878
1	-1.322439	1.580601	1.379381
8	2.375589	1.177498	-0.118668
8	1.437751	-0.725410	-0.881376
6	0.593735	-1.758476	-0.342038
1	-0.089157	-2.077372	-1.128250
1	1.224296	-2.605984	-0.052576

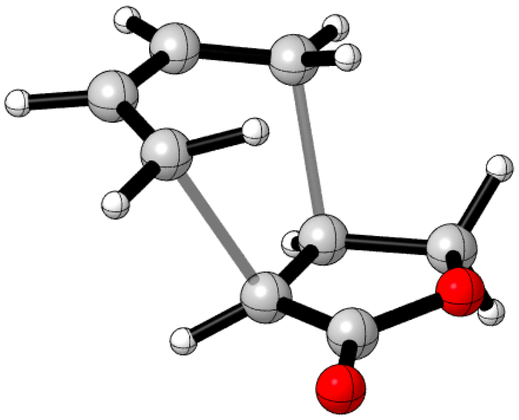

exo furan-2(5H)-one butadiene transition structure
CBS-QB3 PCM Benzene
Energy (0K) = -460.452196
Enthalpy (298K) = -460.442365
Gibbs free Energy (298K) = -460.486183

1	-0.854193	2.625734	-0.236104
6	-1.106601	1.592348	-0.441246
6	-2.280764	1.046428	0.027579
1	-0.560583	1.142434	-1.258423
6	0.068853	-1.026912	0.764161
6	-2.540392	-0.331427	-0.028987
1	-2.938132	1.663972	0.632842
6	0.411291	0.321757	0.884561
1	-0.392661	-1.582746	1.567578
6	-1.602302	-1.231831	-0.516312
1	-3.395410	-0.712309	0.521657
6	1.614910	0.581672	0.077694
1	0.145912	0.979029	1.694800
1	-1.816358	-2.294478	-0.467998
1	-0.966013	-0.944539	-1.344713
8	2.249993	1.592858	-0.076862
8	1.953091	-0.587108	-0.573128
6	1.180766	-1.676712	-0.041062
1	0.853066	-2.296723	-0.876530
1	1.825135	-2.278313	0.608480

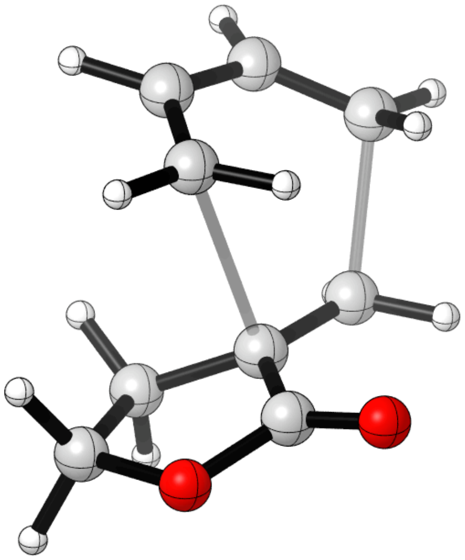

exo 3-methylenedihydrofuran-2(3H)-one butadiene transition structure
CBS-QB3 PCM Benzene
Energy (0K) = -499.680853
Enthalpy (298K) = -499.669547
Gibbs free Energy (298K) = -499.716737

6	2.454181	0.917666	0.186967
6	2.685464	-0.450458	0.054846
6	1.856744	-1.264017	-0.738157
6	0.786241	-0.767957	-1.438665
6	-0.340296	0.313757	0.631176
6	0.672850	1.130381	1.130963
6	-1.360275	0.810738	-0.302534
8	-1.388070	1.845856	-0.927023
8	-2.377311	-0.104078	-0.382991
6	-2.037008	-1.309212	0.342044
1	2.096560	1.469518	-0.674274
1	3.107455	1.492459	0.834676
1	3.390238	-0.933455	0.724940
1	1.985740	-2.340589	-0.672010
1	0.105034	-1.437786	-1.950619
1	0.707113	0.273625	-1.713028
1	0.600970	2.185265	0.888679
1	1.046294	0.914138	2.124755
6	-0.865650	-0.942956	1.268168
1	-1.221087	-0.734357	2.284533
1	-0.123439	-1.741848	1.340330
1	-2.930554	-1.637085	0.871742
1	-1.754779	-2.071764	-0.388457

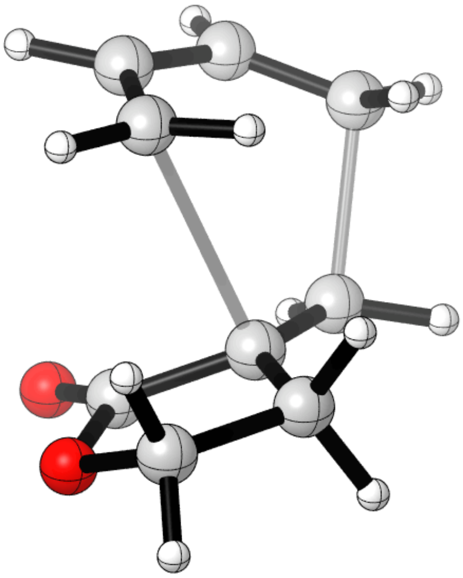

endo 3-methylenedihydrofuran-2(3H)-one butadiene transition structure
CBS-QB3 PCM Benzene
Energy (0K) = -499.680144
Enthalpy (298K) = -499.668849
Gibbs free Energy (298K) = -499.715822

6	-2.446311	0.984480	-0.165364
6	-2.633578	-0.372955	0.084644
6	-1.777130	-1.103471	0.926749
6	-0.738999	-0.525902	1.608870
6	0.398175	0.635429	-0.427981
6	-0.686132	1.097519	-1.169943
6	1.022043	-0.662391	-0.725664
8	0.640668	-1.544191	-1.457706
8	2.220365	-0.749219	-0.060271
6	2.399130	0.385697	0.815613
1	-2.092573	1.629304	0.631088
1	-3.120798	1.477211	-0.857640
1	-3.312551	-0.929409	-0.553903
1	-1.847020	-2.186158	0.903603
1	-0.026654	-1.140881	2.145902
1	-0.724293	0.529766	1.837721
1	-0.782212	2.168598	-1.314398
1	-0.979551	0.493503	-2.019473
6	1.384495	1.453443	0.364089
1	3.436281	0.707873	0.729829
1	2.213883	0.055570	1.840846
1	1.864732	2.200296	-0.278650
1	0.952332	1.990509	1.212807

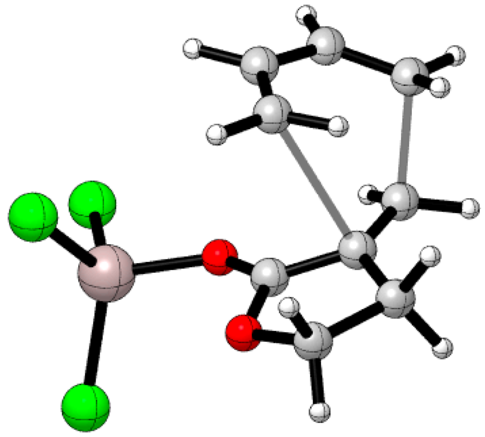

endo alpha-methylene butyrolactone $AlCl_3$ complex butadiene transition structure
CBS-QB3 PCM Benzene
Energy (0K) = -2121.230830
Enthalpy (298K) = -2121.212384
Gibbs free Energy (298K) = -2121.279507

6	-3.504532	-1.704226	-0.681999
6	-2.532631	-2.344992	0.080986
6	-1.925078	-1.772078	1.223132
6	-2.280168	-0.561810	1.735735
6	-2.062123	0.745706	-0.790481
6	-2.506239	-0.264850	-1.646784
6	-0.712523	0.854468	-0.357222
8	-0.466888	1.977156	0.304217
6	-1.723898	2.695607	0.524191
1	-4.198954	-1.023352	-0.202903
1	-3.926339	-2.246674	-1.520667
1	-2.084529	-3.249986	-0.317275
1	-1.052488	-2.271837	1.629465
1	-1.704640	-0.115272	2.537711
1	-3.205912	-0.070909	1.469988
1	-3.357495	-0.039636	-2.280416
1	-1.746136	-0.875118	-2.117240
6	-2.764784	2.022282	-0.392094
1	-1.531459	3.739900	0.291609
1	-1.958840	2.594482	1.584002
1	-2.977702	2.638441	-1.271272
1	-3.713294	1.861358	0.127210
8	0.224297	0.042867	-0.600158
13	1.960095	-0.158320	-0.068097
17	3.061979	1.537278	-0.767716
17	2.557765	-1.989222	-1.003942
17	1.909029	-0.316529	2.074587

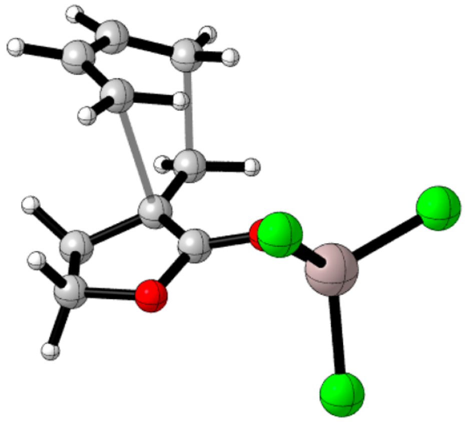

exo alpha-methylene butyrolactone $AlCl_3$ complex butadiene transition structure
CBS-QB3 PCM Benzene
Energy (0K) = -2121.228009
Enthalpy (298K) = -2121.209511
Gibbs free Energy (298K) = -2121.277572

6	3.101141	-1.994198	-0.723017
6	4.013449	-1.454298	0.182253
6	3.621110	-0.734267	1.335335
6	2.319669	-0.532708	1.688007
6	1.868706	0.575786	-0.929140
6	2.310811	-0.487196	-1.725246
6	0.545895	0.708033	-0.432137
8	0.328469	1.875411	0.156609
6	1.594645	2.605401	0.264598
1	2.148256	-2.347570	-0.346307
1	3.492531	-2.581555	-1.546059
1	5.066845	-1.448326	-0.081201
1	4.400145	-0.238808	1.906511
1	2.063183	0.104054	2.526193
1	1.503792	-1.095057	1.256321
1	1.539470	-1.110762	-2.164205
1	3.151340	-0.287559	-2.379309
6	2.575914	1.881453	-0.672256
1	2.717894	2.434466	-1.606947
1	3.560928	1.753259	-0.215872
1	1.385236	3.637160	-0.005687
1	1.892183	2.551263	1.312686
8	-0.388059	-0.138192	-0.529646
13	-2.129474	-0.240445	0.025840
17	-2.094446	-0.083377	2.165693
17	-2.738829	-2.181079	-0.641747
17	-3.194370	1.349717	-0.932071

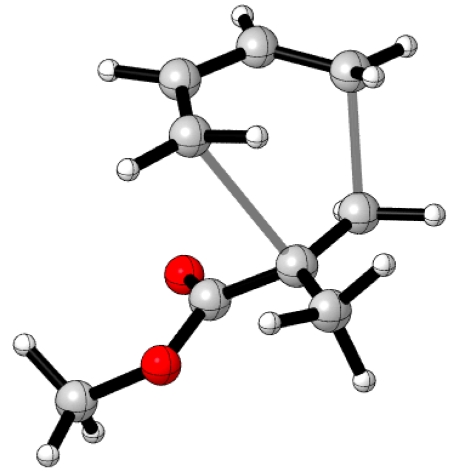

endo s-cis-methyl methacrylate butadiene transition structure
CBS-QB3 PCM Benzene
Energy (0K) = -500.865281
Enthalpy (298K) = -500.851990
Gibbs free Energy (298K) = -500.903259

6	-2.730092	0.193178	-0.381472
6	-2.384447	-1.138418	-0.159173
6	-1.440650	-1.509648	0.814472
6	-0.826006	-0.596409	1.629816
6	0.026454	1.017160	-0.189310
6	-1.052026	1.089126	-1.080496
6	1.048971	0.002823	-0.489676
8	0.945209	-0.872796	-1.329386
8	2.172087	0.155374	0.264107
6	3.220420	-0.792717	0.015552
1	-2.799214	0.869033	0.463791
1	-3.436438	0.415127	-1.174556
1	-2.673866	-1.881897	-0.895078
1	-1.064920	-2.527734	0.792063
1	0.002858	-0.888003	2.264025
1	-1.265542	0.365988	1.844791
1	-1.546856	2.049919	-1.180281
1	-0.972125	0.514890	-1.993468
1	3.523441	-0.769946	-1.032446
1	2.894647	-1.804146	0.266759
1	4.045887	-0.491367	0.657428
6	0.326973	2.152977	0.759602
1	0.873686	1.821609	1.641695
1	0.939848	2.917855	0.267535
1	-0.597364	2.639627	1.084094

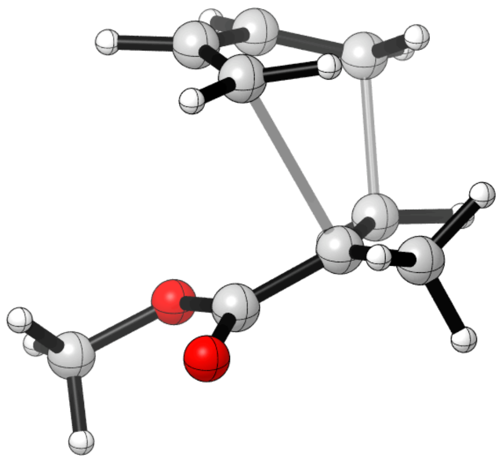

endo s-trans-methyl methacrylate butadiene transition structure
CBS-QB3 PCM Benzene
Energy (0K) = -500.865127
Enthalpy (298K) = -500.851766
Gibbs free Energy (298K) = -500.903238

6	-2.506080	-0.315713	-0.728137
6	-2.058716	-1.427621	-0.018166
6	-1.294514	-1.302881	1.154734
6	-0.980443	-0.087263	1.704779
6	-0.002596	1.068673	-0.358089
6	-0.824745	0.614689	-1.398119
6	1.262944	0.384519	-0.043920
8	2.151691	0.845530	0.648114
8	1.371696	-0.831895	-0.644710
6	2.580212	-1.554061	-0.368151
1	-2.828831	0.565809	-0.185597
1	-3.049641	-0.479858	-1.652767
1	-2.100516	-2.401239	-0.496749
1	-0.796219	-2.191138	1.531179
1	-0.262521	-0.012248	2.512879
1	-1.584607	0.790564	1.531654
1	-1.407592	1.366090	-1.922181
1	-0.459183	-0.188516	-2.021236
1	2.670315	-1.764175	0.699347
1	3.453694	-0.985330	-0.690852
1	2.501829	-2.482232	-0.930880
6	-0.142619	2.475112	0.171958
1	0.277031	2.572390	1.173000
1	0.390515	3.185611	-0.470982
1	-1.192032	2.782450	0.194632

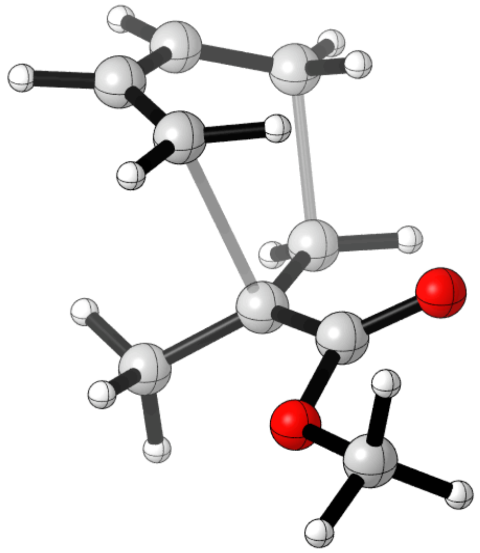

exo s-cis-methyl methacrylate butadiene transition structure
CBS-QB3 PCM Benzene
Energy (0K) = -500.864620
Enthalpy (298K) = -500.851288
Gibbs free Energy (298K) = -500.902998

6	2.379809	1.149120	-0.370900
6	2.893391	-0.145898	-0.348082
6	2.120701	-1.251960	-0.748474
6	0.841042	-1.123256	-1.222724
6	-0.053222	0.163867	0.840213
6	0.872830	1.206965	0.979388
6	-1.244753	0.446520	0.025732
8	-1.361186	1.381973	-0.748031
8	-2.238393	-0.458618	0.218440
6	-3.432297	-0.248607	-0.552309
1	1.701143	1.431277	-1.167654
1	3.010640	1.960702	-0.023692
1	3.825524	-0.333580	0.176435
1	2.499642	-2.245029	-0.524292
1	0.225365	-1.999359	-1.391031
1	0.480756	-0.202393	-1.656918
1	0.555813	2.190260	0.653838
1	1.498768	1.195136	1.864042
1	-3.217320	-0.305948	-1.620799
1	-3.868117	0.726778	-0.330851
1	-4.112897	-1.045037	-0.258660
6	-0.013542	-1.005974	1.791803
1	1.020871	-1.325736	1.948505
1	-0.417618	-0.723914	2.772304
1	-0.589700	-1.854654	1.427631

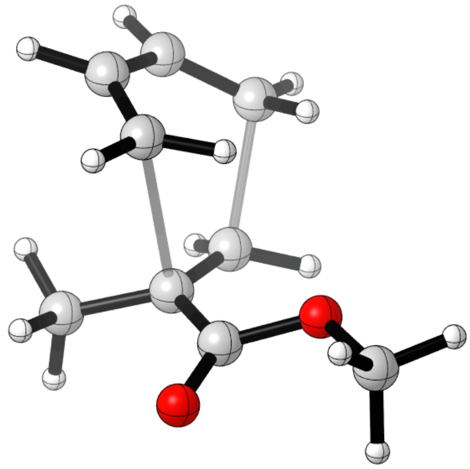

exo s-trans-methyl methacrylate butadiene transition structure
CBS-QB3 PCM Benzene
Energy (0K) = -500.864503
Enthalpy (298K) = -500.851154; Gibbs free Energy (298K) = -500.902759

6	1.915821	-1.438875	-0.649222
6	2.742386	-0.655550	0.153541
6	2.237603	0.051130	1.260559
6	0.921499	-0.016999	1.639414
6	-0.032374	0.708222	-0.637060
6	0.586133	-0.179071	-1.529778
6	-1.366228	0.412014	-0.092091
8	-2.126977	1.230019	0.386924
8	-1.687670	-0.912111	-0.177109
6	-2.993259	-1.267941	0.305745
1	1.109145	-1.995141	-0.186340
1	2.350807	-1.932830	-1.511787
1	3.741557	-0.414659	-0.196864
1	2.877996	0.796561	1.723266
1	0.530209	0.647625	2.400775
1	0.296190	-0.858376	1.380404
1	-0.006049	-0.996276	-1.920900
1	1.285278	0.251933	-2.237412
1	-3.769015	-0.763844	-0.273075
1	-3.104105	-0.998459	1.357228
1	-3.067604	-2.346175	0.179639
6	0.426837	2.141976	-0.568522
1	1.518610	2.189549	-0.526528
1	0.109854	2.693975	-1.462137
1	0.013808	2.653412	0.299691

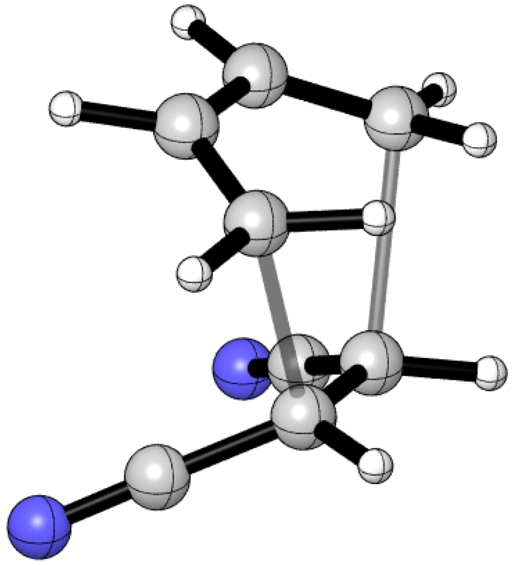

endo maleonitrile butadiene transition structure
CBS-QB3 PCM Benzene
Energy (0K) = -418.302683
Enthalpy (298K) = -418.291922
Gibbs free Energy (298K) = -418.338112

6	-0.340537	1.523371	1.428611
6	0.820176	1.724298	0.699878
6	0.820176	1.724298	-0.699878
6	-0.340537	1.523371	-1.428611
6	-0.978881	-0.503646	-0.705829
6	-0.978881	-0.503646	0.705829
1	-1.294202	1.871531	1.054301
1	-0.287646	1.398365	2.503774
1	1.774721	1.668075	1.212448
1	1.774721	1.668075	-1.212448
1	-0.287646	1.398365	-2.503774
1	-1.294202	1.871531	-1.054301
1	-1.933397	-0.368971	-1.198625
1	-1.933397	-0.368971	1.198625
6	-0.038629	-1.279906	1.447655
6	-0.038629	-1.279906	-1.447655
7	0.709679	-1.907671	2.065964
7	0.709679	-1.907671	-2.065964

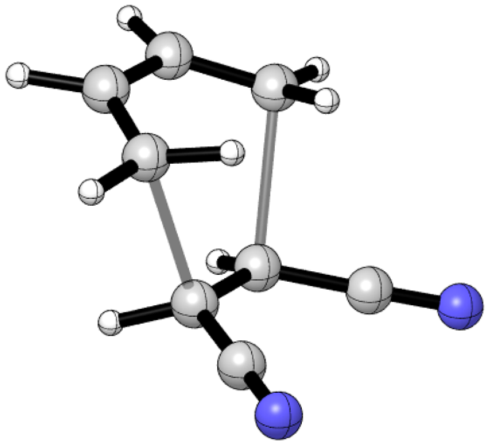

exo maleonitrile butadiene transition structure
CBS-QB3 PCM Benzene
Energy (0K) = -418.302072
Enthalpy (298K) = -418.291316
Gibbs free Energy (298K) = -418.337470

6	-0.508424	1.276664	1.430372
6	-0.003760	2.340254	0.699701
6	-0.003760	2.340254	-0.699701
6	-0.508424	1.276664	-1.430372
6	0.788275	-0.409974	-0.706715
6	0.788275	-0.409974	0.706715
1	-1.344853	0.699034	1.060712
1	-0.375800	1.246750	2.505573
1	0.583457	3.095630	1.212812
1	0.583457	3.095630	-1.212812
1	-0.375800	1.246750	-2.505573
1	-1.344853	0.699034	-1.060712
1	1.652046	0.010226	-1.202711
1	1.652046	0.010226	1.202711
6	0.123836	-1.432381	-1.446865
6	0.123836	-1.432381	1.446865
7	-0.416344	-2.242718	-2.069946
7	-0.416344	-2.242718	2.069946

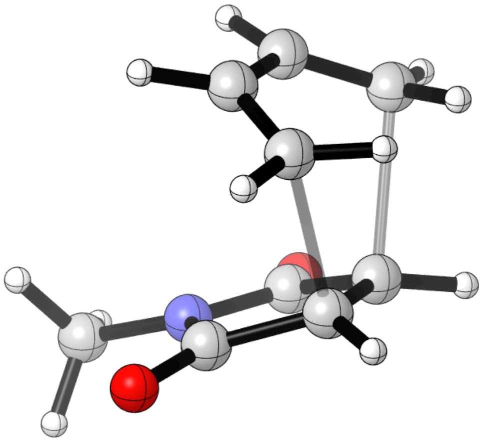

endo N-methylmaleimide butadiene transition structure
CBS-QB3 PCM Benzene
Energy (0K) = -553.800672

Enthalpy (298K) = -553.788266
Gibbs free Energy (298K) = -553.838187

6	1.998854	1.434126	0.005736
6	1.808834	0.695496	1.158130
6	1.806495	-0.708830	1.154035
6	1.993643	-1.441679	-0.002653
6	0.287962	-0.695190	-1.294473
6	0.290269	0.696414	-1.293295
6	-0.824995	-1.153319	-0.412707
6	-0.819683	1.159466	-0.411010
8	-1.220471	2.285968	-0.212355
8	-1.236486	-2.276013	-0.213522
1	2.622245	1.062002	-0.796203
1	1.849510	2.507033	0.018430
1	1.449058	1.202927	2.047698
1	1.444915	-1.220141	2.040675
1	1.841508	-2.514275	0.004697
1	2.618965	-1.067503	-0.802174
1	0.632778	-1.327645	-2.098343
1	0.639097	1.329876	-2.094604
7	-1.358823	0.002379	0.167060
6	-2.481231	-0.000802	1.089409
1	-2.785180	1.033762	1.239800
1	-3.311329	-0.573945	0.672851
1	-2.195030	-0.442476	2.045738

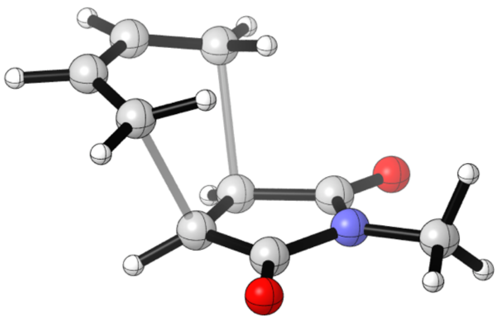

exo N-methylmaleimide butadiene transition structure
CBS-QB3 PCM Benzene
Energy (0K) = -553.795884
Enthalpy (298K) = -553.783306
Gibbs free Energy (298K) = -553.834026

6	-1.723258	-1.441945	-0.578217
6	-2.847766	-0.705696	-0.259481
6	-2.849792	0.698530	-0.261803
6	-1.727546	1.436946	-0.582807
6	-0.270639	0.695670	1.003941
6	-0.269484	-0.696608	1.003893
6	0.979702	1.156621	0.327947
6	0.983365	-1.152745	0.328114
8	1.404238	-2.279656	0.182161
8	1.392209	2.286637	0.183285
1	-1.006146	-1.070213	-1.298311

1	-1.711104	-2.515989	-0.437674
1	-3.690038	-1.214249	0.200219
1	-3.693648	1.206183	0.195976
1	-1.717959	2.511332	-0.444772
1	-1.008065	1.064834	-1.300364
1	-0.741333	1.328060	1.739261
1	-0.737315	-1.328945	1.741105
7	1.627037	0.001683	-0.130642
6	2.882896	-0.001183	-0.861400
1	3.589423	-0.678437	-0.380168
1	2.735994	-0.322522	-1.895087
1	3.274505	1.014772	-0.850378

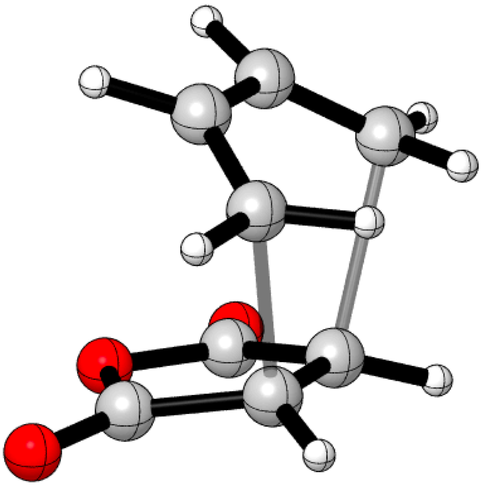

endo maleic anhydride butadiene transition structure
CBS-QB3 PCM Benzene
Energy (0K) = -534.441197
Enthalpy (298K) = -534.430785
Gibbs free Energy (298K) = -534.476190

6	1.684300	-1.434405	0.323966
6	1.811337	-0.700619	-0.843575
6	1.811417	0.700436	-0.843509
6	1.684430	1.434129	0.324103
6	-0.273070	0.697444	1.135628
6	-0.273195	-0.697392	1.135652
6	-1.116259	1.140860	0.004800
6	-1.116432	-1.140719	0.004845
8	-1.488746	-2.228339	-0.321693
8	-1.475280	0.000093	-0.722104
8	-1.488400	2.228532	-0.321760
1	2.091581	-1.065149	1.255931
1	1.540354	-2.507064	0.274655
1	1.696175	-1.210572	-1.794499
1	1.696332	1.210496	-1.794385
1	1.540647	2.506814	0.274880
1	2.091674	1.064751	1.256036
1	-0.156191	1.332495	2.000176
1	-0.156336	-1.332453	2.000198

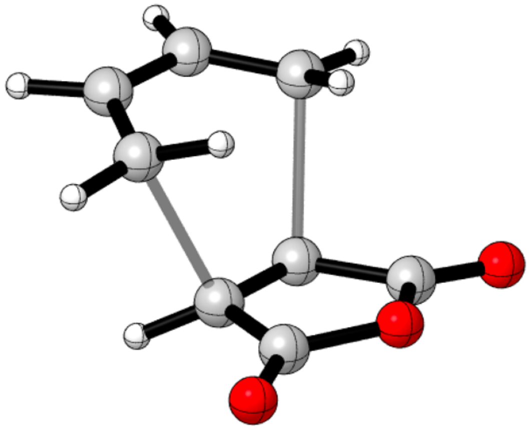

exo maleic anhydride butadiene transition structure
CBS-QB3 PCM Benzene
Energy (0K) = -534.437477
Enthalpy (298K) = -534.426988
Gibbs free Energy (298K) = -534.472625

6	1.470714	1.436731	-0.498538
6	2.545485	0.700958	-0.032540
6	2.545467	-0.700992	-0.032572
6	1.470674	-1.436716	-0.498601
6	-0.145087	-0.697541	0.894657
6	-0.145083	0.697506	0.894688
6	-1.293017	-1.138665	0.071161
6	-1.293004	1.138675	0.071205
8	-1.727058	2.230858	-0.144071
8	-1.873998	0.000019	-0.499338
8	-1.727084	-2.230835	-0.144153
1	0.863919	1.066999	-1.314798
1	1.440399	2.510757	-0.360759
1	3.319501	1.210458	0.533236
1	3.319470	-1.210539	0.533178
1	1.440336	-2.510748	-0.360878
1	0.863890	-1.066930	-1.314847
1	0.220349	-1.333478	1.684271
1	0.220359	1.333409	1.684328

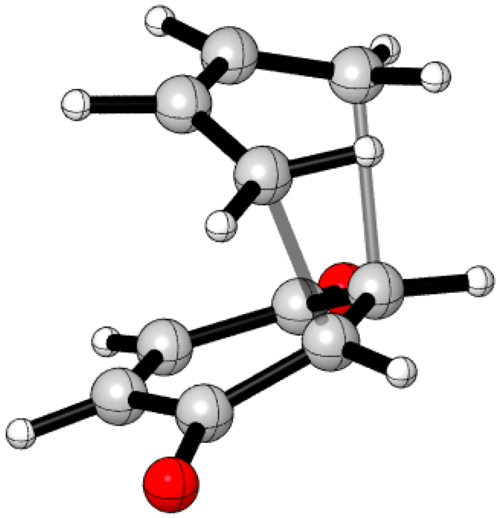

endo benzoquinone butadiene transition structure
CBS-QB3 PCM Benzene
Energy (0K) = -536.520361
Enthalpy (298K) = -536.508888
Gibbs free Energy (298K) = -536.556527

6	-1.788913	-0.260894	1.425563
6	-1.900024	0.916095	0.699176
6	-1.900024	0.916095	-0.699176
6	-1.788913	-0.260894	-1.425563
6	0.136719	-1.118553	-0.703366
6	0.136719	-1.118553	0.703366
6	0.982022	-0.160776	-1.450795
8	1.240463	-0.287723	-2.639276
1	-2.226535	-1.177701	1.053549
1	-1.644366	-0.225870	2.498582
1	-1.775193	1.863138	1.214333
1	-1.775193	1.863138	-1.214333
1	-1.644366	-0.225870	-2.498582
1	-2.226535	-1.177701	-1.053549
1	-0.069444	-2.041406	-1.232638
1	-0.069444	-2.041406	1.232638
6	1.535176	0.976966	-0.670308
6	1.535176	0.976966	0.670308
1	2.001955	1.766595	-1.249738
1	2.001955	1.766595	1.249738
6	0.982022	-0.160776	1.450795
8	1.240463	-0.287723	2.639276

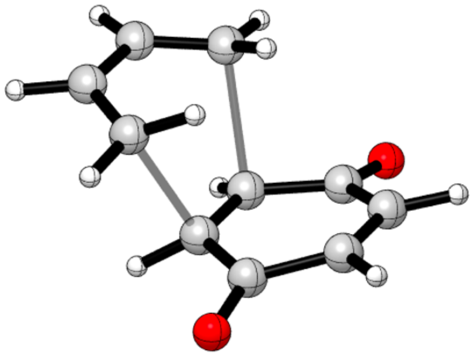

exo benzoquinone butadiene transition structure
CBS-QB3 PCM Benzene
Energy (0K) = -536.516572
Enthalpy (298K) = -536.504997
Gibbs free Energy (298K) = -536.552953

6	-0.552872	1.564729	1.429347
6	-0.123424	2.661424	0.699786
6	-0.123424	2.661424	-0.699786
6	-0.552872	1.564729	-1.429347
6	0.865195	-0.017020	0.703200
6	0.865195	-0.017020	-0.703200
6	0.194764	-1.106511	1.449610
8	0.362043	-1.286979	2.647011
1	-1.358963	0.944424	1.058072

1	-0.406432	1.528542	2.501942
1	0.419373	3.449320	1.213142
1	0.419373	3.449320	-1.213142
1	-0.406432	1.528542	-2.501942
1	-1.358963	0.944424	-1.058072
1	1.683470	0.448260	1.236398
1	1.683470	0.448260	-1.236398
6	-0.704271	-2.000611	0.669740
6	-0.704271	-2.000611	-0.669740
1	-1.310149	-2.686785	1.251771
1	-1.310149	-2.686785	-1.251771
6	0.194764	-1.106511	-1.449610
8	0.362043	-1.286979	-2.647011

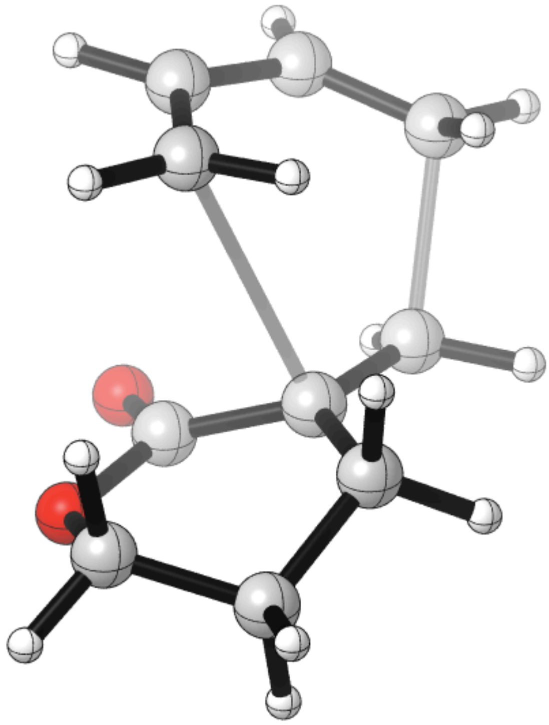

endo 3-methylenetetrahydro-2H-pyran-2-one butadiene transition structure

CBS-QB3 PCM Benzene

Energy (0K) = -538.904507 -538.905633

Enthalpy (298K) = -538.892142 -538.893294

Gibbs free energy (298K) = -538.941717 -538.942559

6	-2.574362	1.090799	-0.024680
6	-2.834458	-0.267450	0.175195
6	-2.016490	-1.079070	0.982087
6	-0.936119	-0.593556	1.664842
6	0.209587	0.646863	-0.435845
6	-0.914253	1.183053	-1.085301
6	0.590835	-0.720000	-0.829501
8	-0.064797	-1.413342	-1.583509
8	1.770181	-1.236370	-0.364186
6	2.546329	-0.512627	0.609655
1	-2.185223	1.674575	0.802882
1	-3.257656	1.646263	-0.658753

1	-3.554715	-0.758651	-0.471134
1	-2.162917	-2.152987	0.930075
1	-0.255300	-1.267486	2.171583
1	-0.827807	0.455254	1.895061
1	-0.947215	2.264320	-1.180983
1	-1.270149	0.638507	-1.949217
6	1.161920	1.538416	0.342068
1	2.125945	-0.695460	1.605557
1	1.144100	2.547256	-0.081079
1	0.849620	1.646300	1.388937
6	2.584974	0.973951	0.304337
1	3.025524	1.119652	-0.687323
1	3.227487	1.483031	1.028322
1	3.537450	-0.965157	0.570491

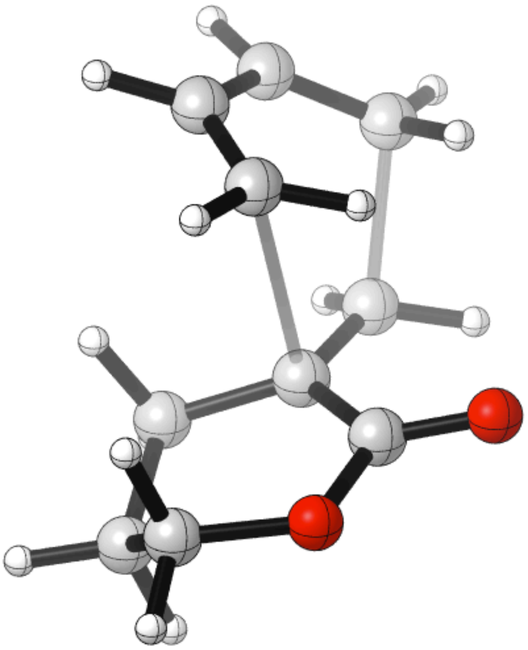

exo 3-methylenetetrahydro-2H-pyran-2-one butadiene transition structure
CBS-QB3 PCM Benzene
Energy (0K) = -538.903814 -538.906527
Enthalpy (298K) = -538.891430 -538.894196
Gibbs free energy (298K) = -538.941326 -538.943691

6	2.670147	0.606020	0.405653
6	2.806961	-0.710791	-0.045650
6	1.961636	-1.241381	-1.037726
6	0.984002	-0.503247	-1.651110
6	-0.145483	0.227286	0.708695
6	0.977026	0.754887	1.371151
6	-0.900562	1.141998	-0.158994
8	-0.541227	2.274318	-0.428110
8	-2.084265	0.713607	-0.689732
6	-2.471867	-0.669499	-0.570207
1	2.396243	1.366987	-0.317443
1	3.369952	0.957170	1.157013
1	3.446781	-1.393901	0.504656

1	2.008789	-2.310055	-1.227351
1	0.286075	-0.975941	-2.332534
1	0.987595	0.576341	-1.654412
1	1.033780	1.836641	1.409787
1	1.271710	0.252848	2.286271
6	-0.715164	-1.109661	1.143490
1	-3.531586	-0.685643	-0.824948
1	-1.927312	-1.252993	-1.321222
6	-2.210170	-1.205010	0.825267
1	-0.556073	-1.235662	2.219044
1	-0.171883	-1.931621	0.662073
1	-2.561023	-2.238435	0.896372
1	-2.788269	-0.612747	1.542008

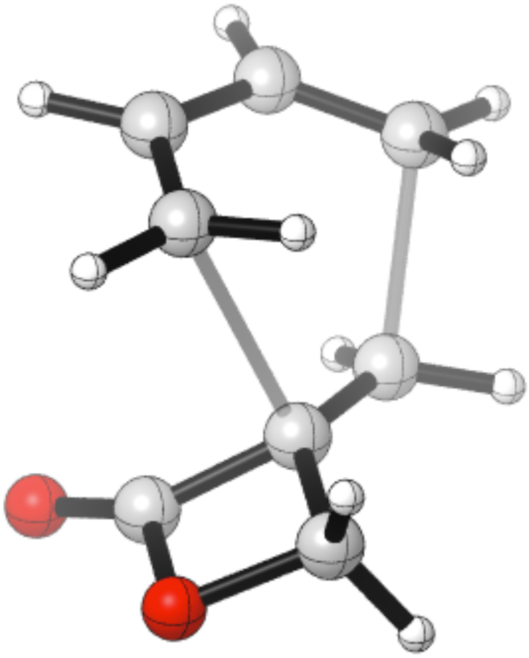

endo 3-methyleneoxetan-2-one butadiene transition structure
CBS-QB3 PCM Benzene
Energy (0K) = -460.424177
Enthalpy (298K) = -460.413774
Gibbs free energy (298K) = -460.458843

6	-2.348688	0.968067	-0.105184
6	-2.487343	-0.407482	-0.235281
6	-1.654107	-1.312878	0.444320
6	-0.682073	-0.897385	1.319365
6	0.546951	0.662679	-0.145448
6	-0.427277	1.325202	-0.874159
6	1.477950	-0.423302	-0.519692
8	1.562599	-1.323693	-1.301246
8	2.435640	-0.035140	0.417182
1	-2.057708	1.396595	0.845863
1	-2.968422	1.617295	-0.714086
1	-3.094973	-0.791931	-1.049009
1	-1.659775	-2.349348	0.122908
1	0.044910	-1.598317	1.712565

1	-0.761617	0.041765	1.847958
1	-0.530880	2.396591	-0.741997
1	-0.638675	0.964250	-1.872360
6	1.606743	1.103501	0.847588
1	2.107192	2.046608	0.618942
1	1.381106	1.036750	1.912677

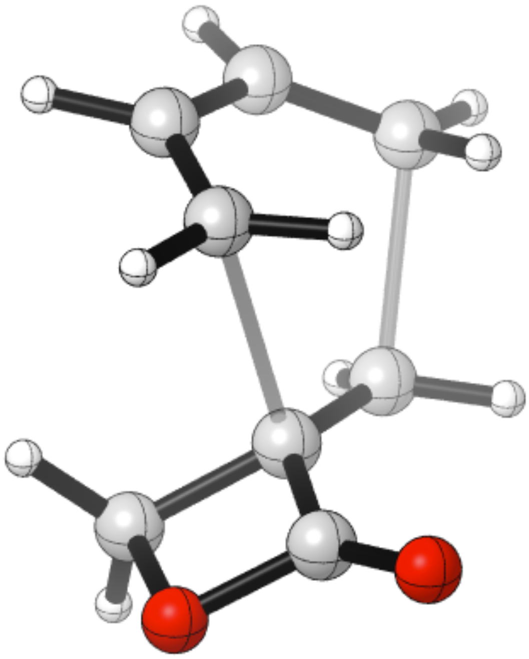

exo 3-methyleneoxetan-2-one butadiene transition structure
CBS-QB3 PCM Benzene
Energy (0K) = -460.423955
Enthalpy (298K) = -460.413598
Gibbs free energy (298K) = -460.458546

6	2.283827	1.090365	-0.127006
6	2.606832	-0.236409	0.116466
6	1.809821	-1.291387	-0.360249
6	0.674910	-1.072719	-1.105133
6	-0.493845	0.332079	0.488473
6	0.389792	1.353136	0.798456
6	-1.706564	0.316646	-0.359834
8	-2.144431	0.867953	-1.326800
8	-2.321493	-0.679440	0.389113
1	1.830364	1.366104	-1.070595
1	2.878581	1.873524	0.329942
1	3.361562	-0.464154	0.863465
1	1.998523	-2.288606	0.025998
1	0.005331	-1.892700	-1.339520
1	0.550810	-0.188902	-1.714787
1	0.246728	2.307556	0.305009
1	0.782736	1.408427	1.805802
6	-1.179908	-0.736527	1.321316
1	-1.493512	-0.426714	2.320162
1	-0.742933	-1.733746	1.341087

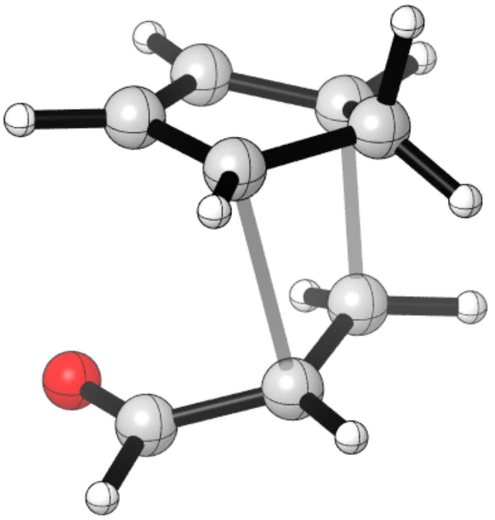

endo s-cis acrolein cyclopentadiene transition structure
CBS-QB3 PCM Benzene
Energy (0K) = -385.297728
Enthalpy (298K) = -385.288744
Gibbs free Energy (298K) = -385.330378

6	-1.303380	0.560975	-0.806394
6	-0.913533	-0.747175	-1.162932
6	-0.699596	-1.503192	0.002496
6	-1.020279	-0.735935	1.104091
6	1.006460	0.949103	0.655546
6	0.406924	1.510391	-0.480906
6	1.959908	-0.123544	0.515324
8	2.255761	-0.654410	-0.552647
1	-1.780072	1.226722	-1.517129
1	-0.691807	-1.078278	-2.168392
1	-0.260450	-2.490910	0.027978
1	-0.950880	-1.048556	2.137095
1	0.842988	1.359754	1.644691
1	-0.032243	2.498504	-0.392907
1	0.894862	1.317341	-1.428500
6	-1.771344	0.475352	0.637801
1	-2.846051	0.236427	0.632333
1	-1.637751	1.375122	1.236310
1	2.424363	-0.476694	1.459541

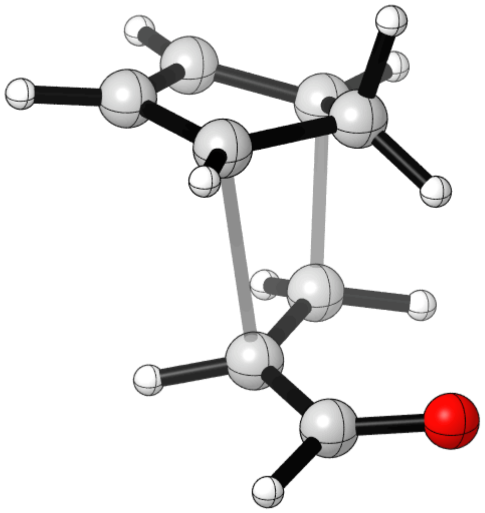

exo s-cis acrolein cyclopentadiene transition structure
CBS-QB3 PCM Benzene
Energy (0K) = -385.296431
Enthalpy (298K) = -385.287475
Gibbs free Energy (298K) = -385.328902

6	-1.165088	0.763473	0.812667
6	-2.100737	0.460860	-0.200162
6	-1.788106	-0.788800	-0.755273
6	-0.728625	-1.343809	-0.052540
6	0.942934	0.437067	-0.970261
6	0.386444	1.491238	-0.229891
1	-1.332237	1.551415	1.537963
1	-2.847113	1.145636	-0.582225
1	-2.248883	-1.215457	-1.636717
1	-0.286761	-2.313315	-0.238701
1	0.951613	1.821024	0.635929
1	-0.121156	2.286612	-0.760835
6	-0.524220	-0.554808	1.200495
1	-1.135996	-1.005854	1.997296
1	0.503554	-0.501368	1.556983
1	0.639076	0.265571	-1.995607
6	2.084022	-0.318261	-0.501561
8	2.591209	-0.236658	0.614053
1	2.508494	-1.022760	-1.247347

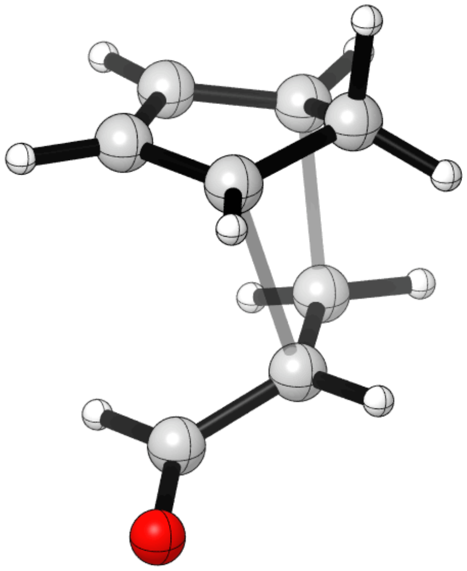

endo s-trans acrolein cyclopentadiene transition structure
CBS-QB3 PCM Benzene
Energy (0K) = -385.296413
Enthalpy (298K) = -385.287404
Gibbs free Energy (298K) = -385.329083

6	-1.730484	0.648316	-0.176277
6	-1.583953	-0.411054	-1.096628
6	-0.812222	-1.422443	-0.511242
6	-0.527824	-1.069988	0.804153
6	1.000445	0.813762	0.333287
6	0.047659	1.599382	-0.334947
6	1.982984	0.017074	-0.380730
8	2.988846	-0.473955	0.105157
1	-2.456190	1.441226	-0.315833
1	-1.889779	-0.379012	-2.134537
1	-0.418599	-2.289104	-1.025658
1	0.033477	-1.668426	1.508564
1	1.199777	0.967330	1.388072
1	-0.291325	2.508647	0.149610
1	0.126737	1.690366	-1.412765
6	-1.456828	0.044434	1.188830
1	-2.395538	-0.403375	1.550461
1	-1.095528	0.727625	1.955007
1	1.757543	-0.120527	-1.462857

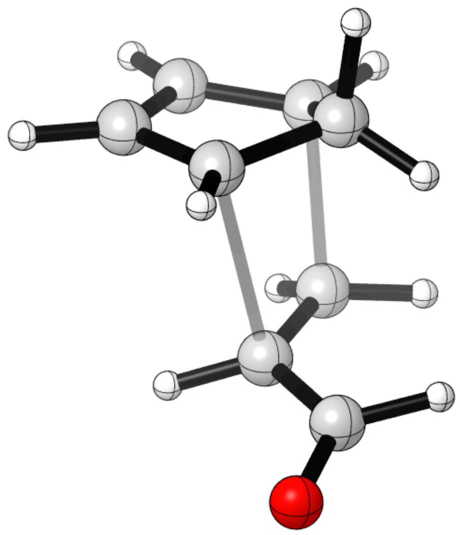

exo s-trans acrolein cyclopentadiene transition structure
CBS-QB3 PCM Benzene
Energy (0K) = -385.295439
Enthalpy (298K) = -385.286424
Gibbs free Energy (298K) = -385.328152

6	-1.563588	0.761473	0.484508
6	-2.108825	0.057954	-0.610156
6	-1.421922	-1.154701	-0.764021
6	-0.501558	-1.286611	0.267451
6	0.976527	0.516183	-0.642208
6	0.112975	1.571246	-0.307517
1	-2.060413	1.615065	0.931332
1	-2.838429	0.456022	-1.303767
1	-1.523675	-1.830731	-1.602800
1	0.160636	-2.127739	0.419518
1	0.372290	2.173281	0.559741
1	-0.345128	2.141890	-1.105086
6	-0.850012	-0.280365	1.324032
1	-1.595803	-0.733514	1.995167
1	-0.025475	0.077046	1.937157
1	0.980507	0.098381	-1.641502
6	2.112635	0.164762	0.189551
8	3.028062	-0.577503	-0.129094
1	2.113607	0.650675	1.193155

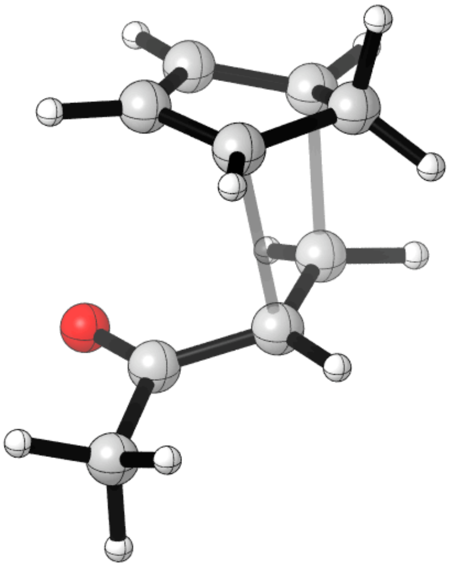

endo s-cis methyl vinyl ketone cyclopentadiene transition structure
CBS-QB3 PCM Benzene
Energy (0K) = -424.535054
Enthalpy (298K) = -424.524349
Gibbs free Energy (298K) = -424.570296

6	1.899811	0.636110	0.186347
6	1.495168	-0.178280	1.264640
6	0.860958	-1.327423	0.764985
6	0.914935	-1.309595	-0.615831
6	-0.680614	0.768645	-0.793625
6	0.265216	1.665677	-0.279231
6	-1.693618	0.204764	0.083778
8	-1.679469	0.399470	1.300002
1	2.628386	1.429370	0.311110
1	1.544838	0.100352	2.308479
1	0.328529	-2.051781	1.365960
1	0.514885	-2.067972	-1.275212
1	-0.753863	0.585019	-1.858345
1	0.760897	2.338363	-0.971260
1	0.043317	2.094307	0.690358
6	1.910788	-0.268887	-1.035345
1	2.902689	-0.742420	-1.098657
1	1.715800	0.217447	-1.989945
6	-2.800552	-0.629221	-0.544311
1	-3.112459	-1.407207	0.154066
1	-3.666364	0.015458	-0.730521
1	-2.503454	-1.077447	-1.494477

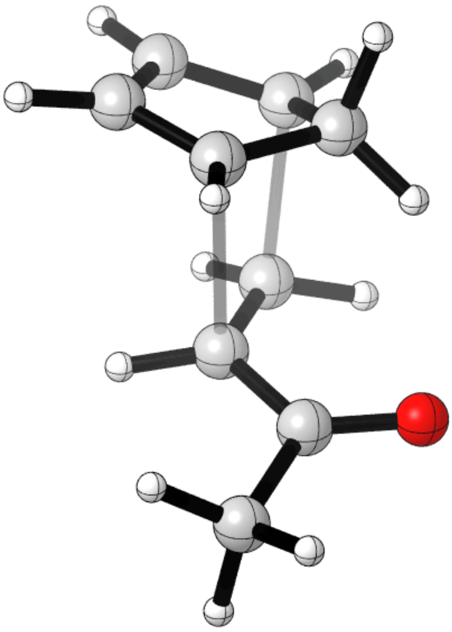

exo s-cis methyl vinyl ketone cyclopentadiene transition structure
CBS-QB3 PCM Benzene
Energy (0K) = -424.533671
Enthalpy (298K) = -424.523088
Gibbs free Energy (298K) = -424.568584

6	1.787790	-0.886136	0.195360
6	2.434218	0.145706	-0.518618
6	1.793486	1.361595	-0.244536
6	0.801852	1.147193	0.704373
6	-0.659276	-0.174865	-0.924214
6	0.193275	-1.286891	-0.971718
1	2.227926	-1.868290	0.323756
1	3.200882	-0.002231	-1.268567
1	1.983869	2.298210	-0.752603
1	0.167710	1.910303	1.135321
1	-0.129081	-2.164734	-0.421342
1	0.715538	-1.501468	-1.895298
6	1.011550	-0.207043	1.305873
1	1.683233	-0.100331	2.172004
1	0.108797	-0.717112	1.638322
1	-0.591709	0.589472	-1.688062
6	-1.828148	-0.137828	-0.049715
8	-2.027281	-0.965934	0.838700
6	-2.840748	0.969660	-0.301833
1	-3.503538	0.669259	-1.120771
1	-3.447820	1.119064	0.591096
1	-2.361557	1.906971	-0.593298

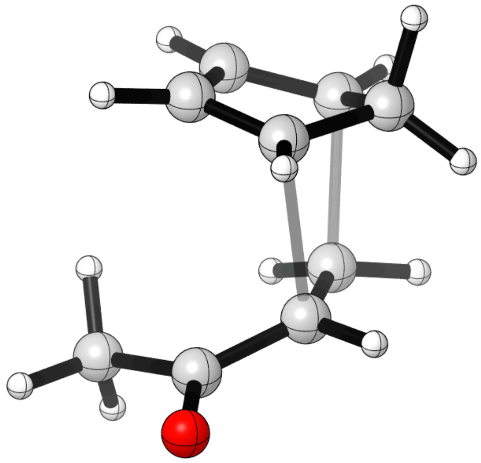

endo s-trans methyl vinyl ketone cyclopentadiene transition structure
CBS-QB3 PCM Benzene
Energy (0K) = -424.532511
Enthalpy (298K) = -424.522020
Gibbs free Energy (298K) = -424.567108

6	-1.937256	0.329226	0.651946
6	-1.605587	1.244500	-0.368162
6	-0.951226	0.559172	-1.400679
6	-0.918638	-0.797313	-1.094540
6	0.652221	-0.697716	0.767042
6	-0.161195	0.061463	1.621976
6	1.828038	-0.196199	0.044362
8	2.607911	-0.967879	-0.501795
1	-2.624632	0.574871	1.453123
1	-1.712117	2.319548	-0.298119
1	-0.460478	1.020598	-2.247537
1	-0.500107	-1.572505	-1.721690
1	0.626622	-1.779737	0.828074
1	-0.617277	-0.445954	2.465268
1	0.106139	1.089232	1.833822
6	-1.924700	-1.038566	-0.004607
1	-2.907809	-1.194153	-0.475244
1	-1.728611	-1.885976	0.649323
6	2.092858	1.301965	0.023053
1	2.458918	1.626134	1.002815
1	2.858251	1.513090	-0.723040
1	1.190733	1.878698	-0.194783

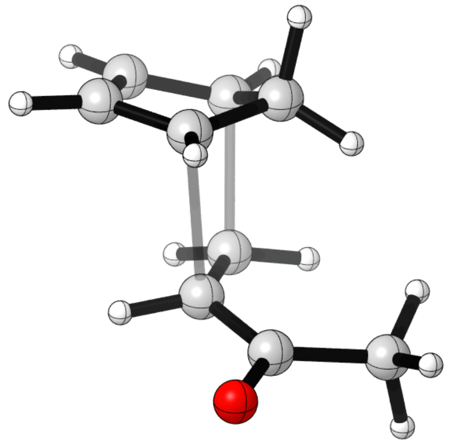

exo s-trans methyl vinyl ketone cyclopentadiene transition structure
CBS-QB3 PCM Benzene
Energy (0K) = -424.528752
Enthalpy (298K) = -424.518093
Gibbs free Energy (298K) = -424.564045

6	1.731990	-1.015872	0.140305
6	2.469018	0.022475	-0.468315
6	1.909103	1.253657	-0.105013
6	0.870044	1.031432	0.793008
6	-0.615579	0.066685	-0.912518
6	0.090379	-1.136792	-1.070320
1	2.089895	-2.038369	0.180061
1	3.242697	-0.117852	-1.212391
1	2.170792	2.212441	-0.533253
1	0.268451	1.797374	1.262430
1	-0.321136	-2.045155	-0.642963
1	0.614654	-1.304622	-2.002337
6	1.011518	-0.370674	1.307695
1	1.710920	-0.353537	2.157976
1	0.098344	-0.855181	1.642192
1	-0.409156	0.892089	-1.582174
6	-1.882114	0.241375	-0.191207
8	-2.500744	1.298108	-0.260063
6	-2.490868	-0.918670	0.589852
1	-3.073708	-0.515590	1.418505
1	-3.178413	-1.454128	-0.073327
1	-1.768331	-1.644020	0.964861

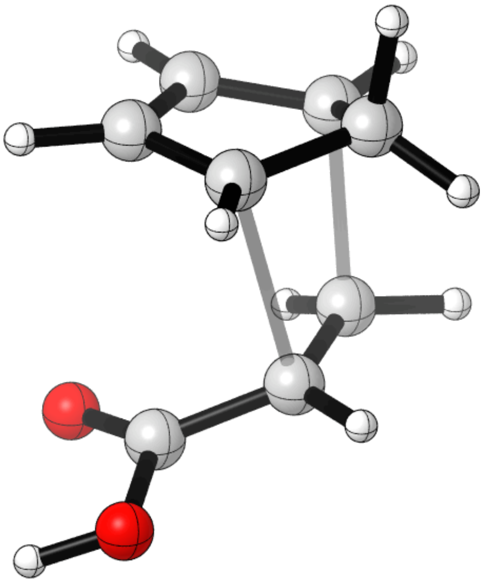

endo s-cis acrylic acid cyclopentadiene transition structure
CBS-QB3 PCM Benzene
Energy (0K) = -460.480327
Enthalpy (298K) = -460.470458
Gibbs free Energy (298K) = -460.514467

6	1.927424	0.601091	0.298678
6	1.541785	-0.386713	1.226813
6	0.862202	-1.411154	0.552966
6	0.861025	-1.134845	-0.806260
6	-0.668417	0.837463	-0.651302
6	0.226853	1.660822	0.040677
6	-1.727295	0.152332	0.070568
8	-1.847668	0.092615	1.281723
8	-2.621969	-0.459474	-0.760417
1	2.652800	1.370270	0.537890
1	1.626854	-0.298004	2.301759
1	0.329358	-2.222053	1.030473
1	0.424154	-1.757021	-1.575496
1	-0.743227	0.859915	-1.729502
1	0.706233	2.464767	-0.506679
1	-0.008112	1.893369	1.071467
6	1.890346	-0.071653	-1.062018
1	2.862177	-0.562728	-1.223701
1	1.700819	0.579559	-1.913687
1	-3.277502	-0.877253	-0.183711

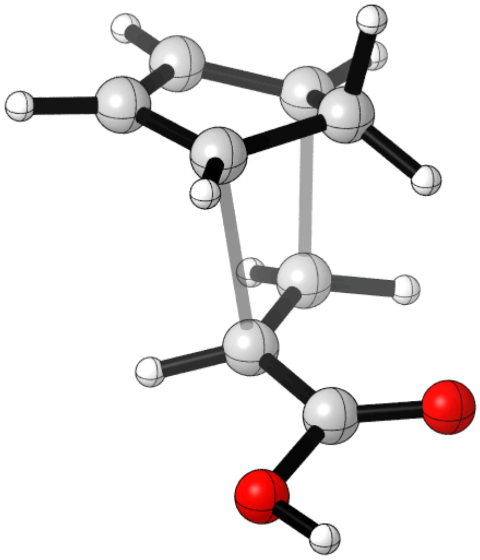

exo s-cis acrylic acid cyclopentadiene transition structure
CBS-QB3 PCM Benzene
Energy (0K) = -460.478869
Enthalpy (298K) = -460.469118
Gibbs free Energy (298K) = -460.512692

6	1.746153	-0.914831	0.267025
6	2.436774	0.069011	-0.471554
6	1.812471	1.307679	-0.277345
6	0.778345	1.154018	0.639403
6	-0.650598	-0.153598	-0.931596
6	0.154100	-1.299567	-0.933875
1	2.153144	-1.903181	0.446712
1	3.224252	-0.129142	-1.187605
1	2.035440	2.214727	-0.824077
1	0.139499	1.946836	1.004517
1	-0.207339	-2.152901	-0.370366
1	0.690660	-1.552410	-1.838712
6	0.956391	-0.167808	1.322791
1	1.614964	-0.019969	2.192696
1	0.042956	-0.648290	1.667423
1	-0.573839	0.585694	-1.715520
6	-1.835207	-0.076879	-0.089829
8	-2.147227	-0.847733	0.803068
8	-2.619644	0.997110	-0.394818
1	-3.375342	0.955463	0.208821

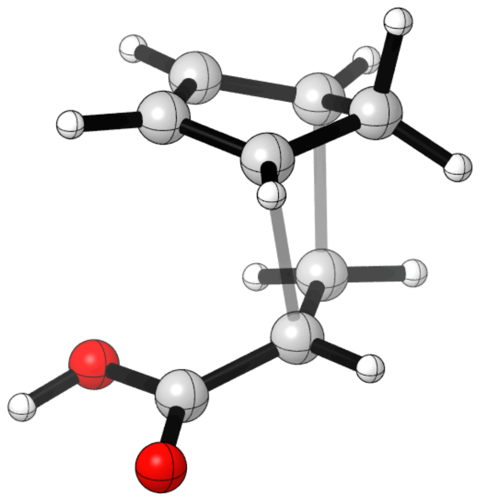

endo s-trans acrylic acid cyclopentadiene transition structure

CBS-QB3 PCM Benzene

Energy (0K) = -460.478338

Enthalpy (298K) = -460.468523

Gibbs free Energy (298K) = -460.512314

6	1.915543	0.520700	0.477552
6	1.539307	-0.683713	1.104505
6	0.880368	-1.497892	0.174332
6	0.884415	-0.865693	-1.063122
6	-0.662465	0.945427	-0.475612
6	0.173142	1.570540	0.459306
6	-1.799351	0.107789	-0.116544
8	-2.658291	-0.288174	-0.881965
8	-1.838927	-0.213059	1.211727
1	2.616093	1.216266	0.925063
1	1.613255	-0.882005	2.165783
1	0.356479	-2.414913	0.407753
1	0.468277	-1.269035	-1.976252
1	-0.680576	1.271720	-1.506466
1	0.645886	2.502135	0.167959
1	-0.096465	1.516964	1.505302
6	1.910923	0.229920	-1.011948
1	2.888795	-0.201748	-1.274902
1	1.734990	1.081207	-1.667176
1	-2.620288	-0.773187	1.324020

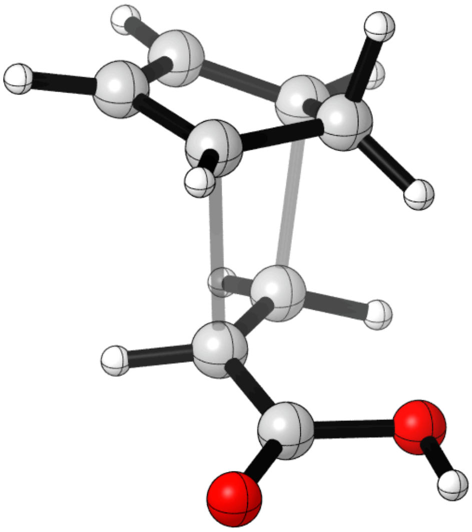

exo s-trans acrylic acid cyclopentadiene transition structure
CBS-QB3 PCM Benzene
Energy (0K) = -460.476617
Enthalpy (298K) = -460.466806
Gibbs free Energy (298K) = -460.510586

6	1.702965	-0.969595	0.234275
6	2.444858	0.013451	-0.453879
6	1.874088	1.269186	-0.212035
6	0.820388	1.121676	0.684097
6	-0.640405	-0.026851	-0.948024
6	0.095538	-1.219245	-0.992928
1	2.063107	-1.982598	0.372123
1	3.231920	-0.188614	-1.169257
1	2.141838	2.187835	-0.717629
1	0.210202	1.924880	1.074560
1	-0.312074	-2.083622	-0.482001
1	0.632875	-1.454715	-1.902377
6	0.944282	-0.231646	1.318911
1	1.612800	-0.140917	2.189212
1	0.015729	-0.688621	1.651085
1	-0.493032	0.738372	-1.696382
6	-1.877226	0.147543	-0.197143
8	-2.640225	1.089099	-0.301269
8	-2.148279	-0.876408	0.673340
1	-2.982261	-0.640646	1.104454

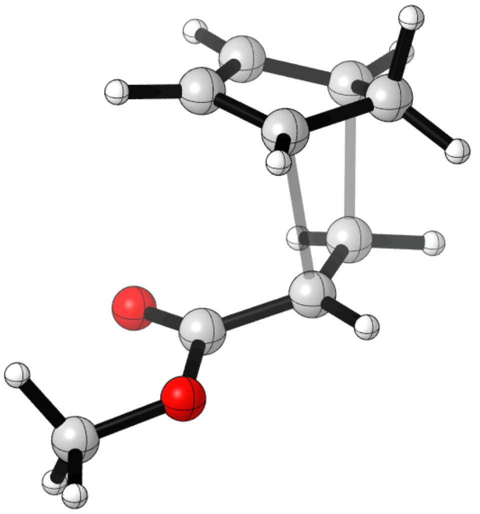

endo s-cis methyl acrylate cyclopentadiene transition structure
CBS-QB3 PCM Benzene
Energy (0K) = -499.691021
Enthalpy (298K) = -499.679531
Gibbs free Energy (298K) = -499.727331

6	2.390204	0.408857	0.257842
6	1.890100	-0.473896	1.235914
6	1.064564	-1.423416	0.618148
6	1.077493	-1.210749	-0.753154
6	-0.165148	0.944465	-0.684584
6	0.840810	1.673844	-0.041709
6	-1.307104	0.457228	0.077760
8	-1.414613	0.476640	1.290771
8	-2.280192	-0.044780	-0.733437
6	-3.448640	-0.543748	-0.066934
1	3.215736	1.083902	0.452609
1	2.003356	-0.348311	2.304585
1	0.434334	-2.131375	1.138940
1	0.550293	-1.804337	-1.487641
1	-0.253046	0.925856	-1.761887
1	1.417300	2.377382	-0.631852
1	0.652354	1.985432	0.977756
1	-3.908292	0.233496	0.546182
1	-3.199156	-1.394037	0.571250
1	-4.128158	-0.853705	-0.858505
6	2.242052	-0.315803	-1.068161
1	3.133852	-0.944965	-1.211915
1	2.133887	0.315093	-1.948939

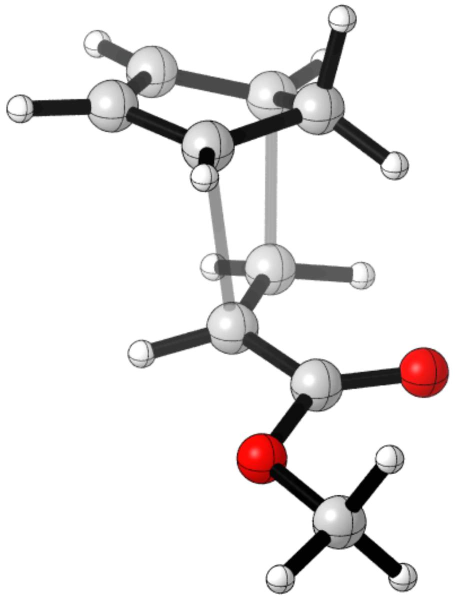

exo s-cis methyl acrylate cyclopentadiene transition structure
CBS-QB3 PCM Benzene
Energy (0K) = -499.689883
Enthalpy (298K) = -499.678350
Gibbs free Energy (298K) = -499.726321

6	2.231285	-0.737552	0.373644
6	2.835833	0.260572	-0.420332
6	2.064875	1.427407	-0.349906
6	1.017029	1.224423	0.541910
6	-0.190451	-0.363551	-0.949739
6	0.747522	-1.398121	-0.840604
1	2.741915	-1.653393	0.648212
1	3.669519	0.101024	-1.092404
1	2.204207	2.308772	-0.962551
1	0.279323	1.964422	0.821766
1	0.469012	-2.248398	-0.227256
1	1.345495	-1.646600	-1.707430
6	1.319051	-0.008596	1.338780
1	1.920233	0.283756	2.213847
1	0.453534	-0.565142	1.692314
1	-0.177241	0.318389	-1.787433
6	-1.414145	-0.381143	-0.155522
8	-1.655934	-1.118561	0.785626
8	-2.304465	0.552661	-0.589772
6	-3.549813	0.605828	0.122270
1	-3.387128	0.863146	1.170730
1	-4.135564	1.379331	-0.370356
1	-4.067230	-0.353712	0.070721

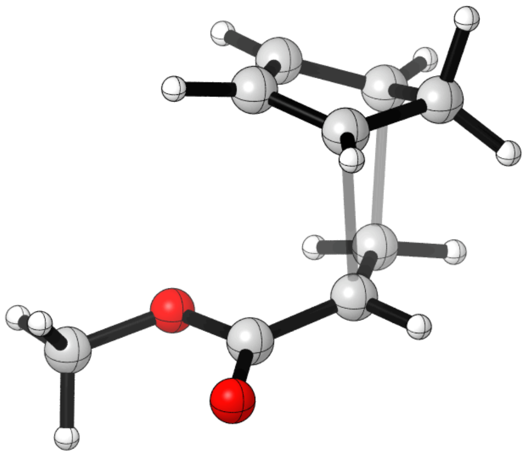

endo s-trans methyl acrylate cyclopentadiene transition structure
CBS-QB3 PCM Benzene
Energy (0K) = -499.689384
Enthalpy (298K) = -499.677896
Gibbs free Energy (298K) = -499.725705

6	-2.175134	0.388795	0.722774
6	-1.534919	1.491993	0.125383
6	-0.970697	1.089085	-1.092309
6	-1.295530	-0.242002	-1.323548
6	0.095512	-1.197092	0.453734
6	-0.616563	-0.649520	1.527686
6	1.384470	-0.694270	-0.015945
8	2.115349	-1.266429	-0.802343
8	1.709428	0.511317	0.530150
6	2.960087	1.069229	0.101649
1	-2.856727	0.492995	1.559137
1	-1.375570	2.451260	0.600279
1	-0.301556	1.684878	-1.698552
1	-1.019102	-0.813096	-2.199452
1	-0.127974	-2.192261	0.093749
1	-1.255221	-1.313549	2.099938
1	-0.146202	0.130362	2.110717
1	2.966237	1.224725	-0.978773
1	3.788432	0.410116	0.367169
1	3.050996	2.020561	0.622293
6	-2.424148	-0.602359	-0.399539
1	-3.371605	-0.328505	-0.888593
1	-2.488387	-1.649742	-0.109682

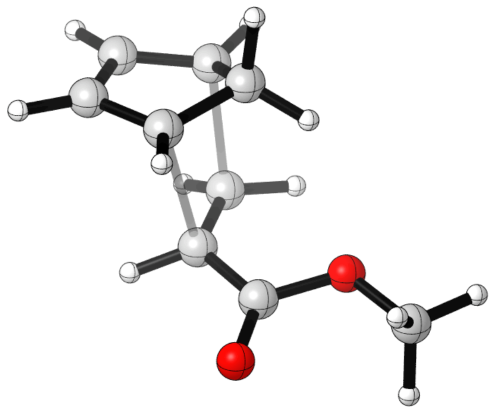

exo s-trans methyl acrylate cyclopentadiene transition structure
CBS-QB3 PCM Benzene
Energy (0K) = -499.687544
Enthalpy (298K) = -499.676040
Gibbs free Energy (298K) = -499.723907

6	-1.942206	-1.119984	0.062674
6	-2.849992	-0.052470	0.223499
6	-2.344662	1.078207	-0.429602
6	-1.158379	0.736967	-1.072579
6	0.128022	0.498875	1.003283
6	-0.507110	-0.667414	1.450159
1	-2.197565	-2.146180	0.300573
1	-3.716775	-0.065142	0.872082
1	-2.752611	2.078181	-0.358516
1	-0.561633	1.401715	-1.682514
1	0.036680	-1.601051	1.369468
1	-1.152146	-0.600431	2.316522
6	-1.087421	-0.760698	-1.135468
1	-1.622227	-1.091987	-2.039296
1	-0.088298	-1.188392	-1.155037
1	-0.183750	1.466462	1.369872
6	1.450347	0.536840	0.379467
8	2.100654	1.546281	0.187483
8	1.917921	-0.698132	0.028792
6	3.221236	-0.720244	-0.573762
1	3.965764	-0.302032	0.105327
1	3.434784	-1.768835	-0.771523
1	3.230164	-0.147978	-1.503184

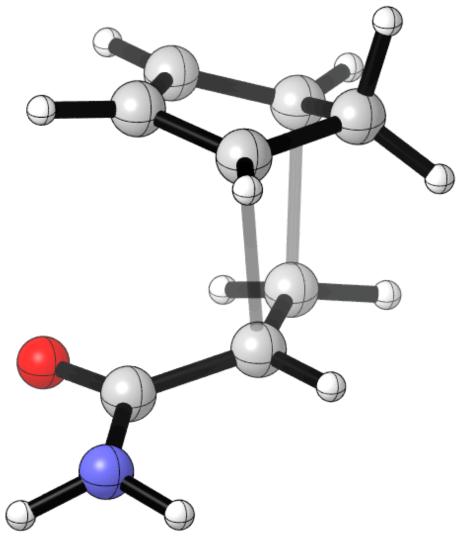

endo s-cis acrylamide cyclopentadiene transition structure
CBS-QB3 PCM Benzene
Energy (0K) = -440.603994
Enthalpy (298K) = -440.593598
Gibbs free Energy (298K) = -440.638465

6	1.955296	0.595442	0.268314
6	1.563623	-0.359542	1.226090
6	0.861320	-1.388987	0.586469
6	0.842242	-1.142052	-0.780147
6	-0.656764	0.788382	-0.673642
6	0.234904	1.651973	-0.030745
6	-1.736273	0.153675	0.101031
8	-1.773142	0.166895	1.329979
1	2.682423	1.371310	0.478921
1	1.653826	-0.240521	2.297618
1	0.316671	-2.175604	1.090239
1	0.401819	-1.789104	-1.527056
1	-0.734389	0.786764	-1.754647
1	0.713108	2.429396	-0.615953
1	0.006810	1.921708	0.992274
6	1.898045	-0.112742	-1.072723
1	2.857119	-0.630756	-1.226240
1	1.718722	0.521764	-1.939613
7	-2.698692	-0.501687	-0.632538
1	-3.522069	-0.802142	-0.134684
1	-2.792416	-0.333060	-1.620813

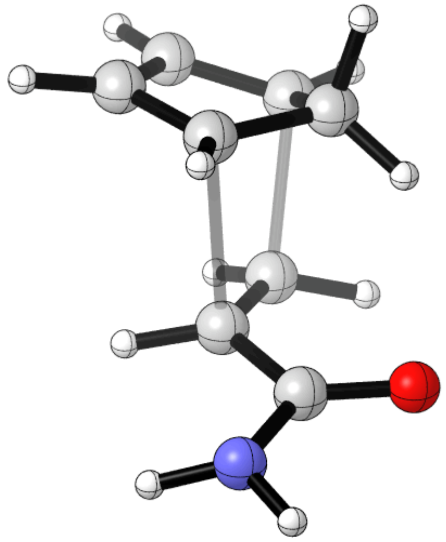

exo s-cis acrylamide cyclopentadiene transition structure
CBS-QB3 PCM Benzene
Energy (0K) = -440.603251
Enthalpy (298K) = -440.592977
Gibbs free Energy (298K) = -440.637401

6	1.776759	-0.922547	0.175505
6	2.455394	0.134748	-0.465485
6	1.812060	1.339469	-0.158630
6	0.767959	1.082084	0.726639
6	-0.642302	-0.050959	-0.913149
6	0.161289	-1.187549	-1.055726
1	2.188815	-1.921886	0.255087
1	3.246128	0.015714	-1.195552
1	2.024988	2.297508	-0.615810
1	0.122616	1.828351	1.170598
1	-0.192914	-2.096341	-0.581677
1	0.695786	-1.336901	-1.984410
6	0.974451	-0.291343	1.293643
1	1.630255	-0.206307	2.174280
1	0.068518	-0.817453	1.588348
1	-0.566742	0.755465	-1.631993
6	-1.851567	-0.090150	-0.069440
8	-2.086029	-0.982190	0.746308
7	-2.713822	0.969517	-0.221889
1	-2.641981	1.579548	-1.019676
1	-3.614746	0.890692	0.223416

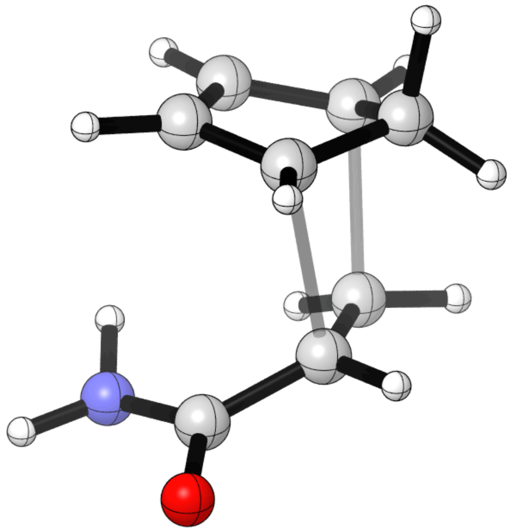

endo s-trans acrylamide cyclopentadiene transition structure

CBS-QB3 PCM Benzene

Energy (0K) = -440.601516

Enthalpy (298K) = -440.591322

Gibbs free Energy (298K) = -440.635657

6	-1.966607	-0.061361	0.686412
6	-1.602528	1.246779	0.315068
6	-0.903684	1.197388	-0.899343
6	-0.854084	-0.123153	-1.341039
6	0.655841	-0.975441	0.283200
6	-0.168735	-0.844341	1.405387
6	1.839706	-0.135935	-0.005260
8	2.712142	-0.498155	-0.788346
1	-2.668495	-0.274918	1.483822
1	-1.728345	2.127288	0.932931
1	-0.390632	2.030101	-1.361785
1	-0.423743	-0.450787	-2.277643
1	0.692741	-1.918545	-0.246181
1	-0.641959	-1.736766	1.798684
1	0.071850	-0.114854	2.168788
6	-1.906369	-0.885632	-0.583318
1	-2.865261	-0.762148	-1.110308
1	-1.728386	-1.951935	-0.456662
7	1.946875	1.052765	0.680702
1	1.129488	1.477868	1.088217
1	2.666243	1.680761	0.355352

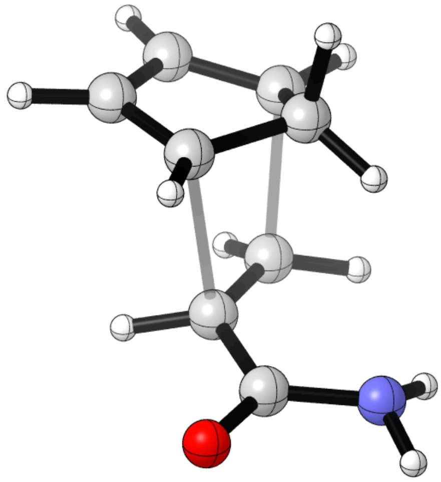

exo s-trans acrylamide cyclopentadiene transition structure
CBS-QB3 PCM Benzene
Energy (0K) = -440.598696
Enthalpy (298K) = -440.588539
Gibbs free Energy (298K) = -440.632847

6	1.745013	-0.970535	0.274023
6	2.475648	0.022849	-0.408849
6	1.857932	1.261362	-0.205157
6	0.769516	1.090170	0.649648
6	-0.612296	-0.038651	-0.928092
6	0.119658	-1.231047	-0.999714
1	2.117471	-1.977419	0.423777
1	3.287441	-0.166246	-1.099755
1	2.110381	2.180326	-0.718272
1	0.124731	1.879194	1.011200
1	-0.246151	-2.125014	-0.505371
1	0.672318	-1.444520	-1.905024
6	0.933684	-0.241422	1.324640
1	1.575965	-0.102541	2.208004
1	0.013645	-0.722303	1.650099
1	-0.455361	0.724284	-1.677613
6	-1.878808	0.174902	-0.193558
8	-2.486775	1.239535	-0.245483
7	-2.342976	-0.861319	0.594852
1	-3.272735	-0.727017	0.964198
1	-2.094753	-1.811551	0.371019

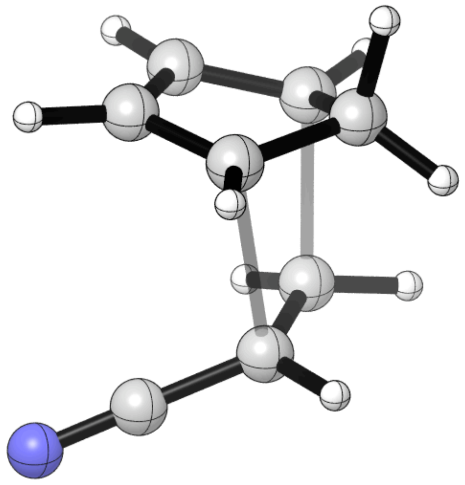

endo acrylonitrile cyclopentadiene transition structure
CBS-QB3 PCM Benzene
Energy (0K) = -364.235889
Enthalpy (298K) = -364.227075
Gibbs free Energy (298K) = -364.268345

6	-1.556252	0.549956	-0.567586
6	-1.185239	-0.666188	-1.175069
6	-0.605236	-1.501627	-0.210904
6	-0.666497	-0.874095	1.027091
6	1.001503	0.875293	0.491138
6	0.211125	1.547424	-0.455512
1	-2.206755	1.269237	-1.051562
1	-1.209695	-0.863057	-2.239035
1	-0.096906	-2.434020	-0.415453
1	-0.322958	-1.295317	1.962154
1	1.008273	1.192379	1.525895
1	-0.241214	2.484006	-0.150825
1	0.513933	1.521349	-1.494168
6	-1.641655	0.261547	0.919305
1	-2.649727	-0.127904	1.128699
1	-1.468409	1.106263	1.584368
6	2.084696	0.051457	0.098052
7	2.974113	-0.616504	-0.229880

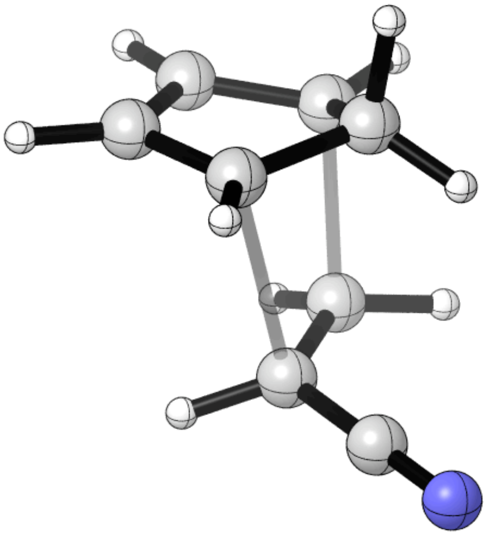

exo acrylonitrile cyclopentadiene transition structure CBS-QB3 PCM Benzene
Energy (0K) = -364.235581
Enthalpy (298K) = -364.226799
Gibbs free Energy (298K) = -364.267955

6	-1.307154	0.678279	0.796558
6	-2.111947	0.296780	-0.297181
6	-1.598848	-0.882713	-0.852973
6	-0.520798	-1.311667	-0.087655
6	0.988921	0.526104	-0.781715
6	0.277305	1.552380	-0.137403
1	-1.613402	1.433325	1.511237
1	-2.904510	0.897149	-0.725251
1	-1.926851	-1.332762	-1.780979
1	0.058966	-2.208722	-0.256848
1	0.709498	1.986885	0.756994
1	-0.260042	2.259373	-0.754789
6	-0.559986	-0.571046	1.216255
1	-1.208202	-1.130704	1.907920
1	0.399272	-0.423055	1.708745
1	0.840992	0.334333	-1.835674
6	2.165927	-0.027987	-0.222178
7	3.129108	-0.482372	0.238056

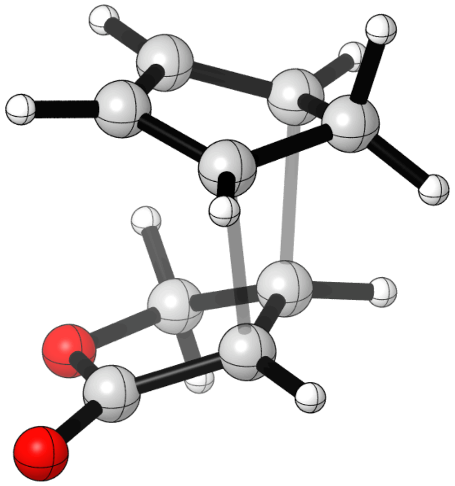

endo furan-2(5H)-one cyclopentadiene transition structure
CBS-QB3 PCM Benzene
Energy (0K) = -498.510353
Enthalpy (298K) = -498.500785
Gibbs free Energy (298K) = -498.544107

1	-2.188053	1.860390	-0.153453
6	-1.725916	0.891469	-0.001788
6	-1.414055	0.390267	1.278882
6	0.127963	1.090933	-0.885728
6	-1.100640	-0.968893	1.173748
1	-1.303960	0.986270	2.175235
6	0.565766	-0.224399	-1.093963
1	-0.238351	1.713744	-1.692151
6	-1.266206	-1.368875	-0.151109
1	-0.684352	-1.579019	1.963923
6	1.638258	-0.527890	-0.139751
1	0.453445	-0.812214	-1.990686
1	-1.137857	-2.378803	-0.516226
8	2.297658	-1.525297	0.012425
8	1.811999	0.578110	0.667571
6	1.087861	1.695697	0.120672
1	0.618496	2.230358	0.946133
1	1.801530	2.363739	-0.373892
6	-2.095058	-0.318361	-0.838457
1	-1.949197	-0.221548	-1.913354
1	-3.156793	-0.545118	-0.660532

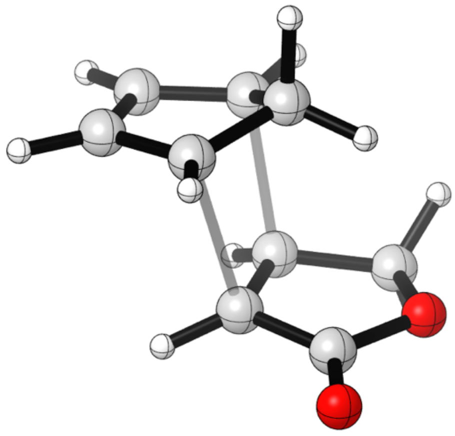

exo furan-2(5H)-one cyclopentadiene transition structure
CBS-QB3 PCM Benzene
Energy (0K) = -498.508449
Enthalpy (298K) = -498.498905
Gibbs free Energy (298K) = -498.542078

1	-0.756104	-2.245922	0.734682
6	-1.151917	-1.272886	0.477459
6	-2.205093	-1.029920	-0.405252
6	0.151557	1.008346	-0.831807
6	-2.460180	0.344421	-0.439332
1	-2.668239	-1.770107	-1.044744
6	0.507825	-0.343158	-0.957599
1	-0.276710	1.578045	-1.644219
6	-1.521587	1.005970	0.380216
1	-3.163314	0.833824	-1.101178
6	1.713482	-0.600054	-0.164625
1	0.237535	-1.011727	-1.756775
1	-1.624265	2.046017	0.669151
8	2.362794	-1.605434	-0.021657
8	2.052882	0.569989	0.493514
6	1.255822	1.655829	-0.011272
1	0.913507	2.247038	0.840058
1	1.888111	2.288163	-0.642793
6	-0.988065	-0.048398	1.328138
1	0.003338	0.119874	1.743211
1	-1.690338	-0.122550	2.172201

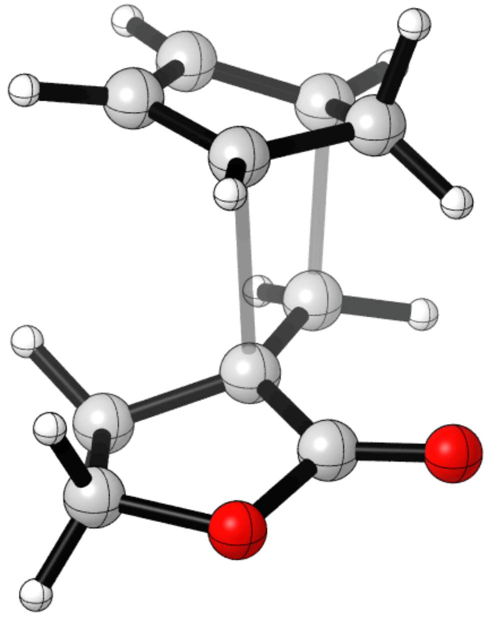

exo 3-methylenedihydrofuran-2(3H)-one cyclopentadiene transition structure
CBS-QB3 PCM Benzene
Energy (0K) = -537.737722
Enthalpy (298K) = -537.726725
Gibbs free Energy (298K) = -537.773214

6	2.199695	0.545216	0.490601
6	2.461299	-0.826075	0.267356
6	1.693645	-1.263822	-0.822029
6	1.006114	-0.184078	-1.357403
6	-0.512777	0.018602	0.744961
6	0.507178	0.551217	1.539357
6	-1.451714	0.849763	-0.010195
8	-1.449817	2.047017	-0.198227
8	-2.450303	0.054351	-0.516918
6	-2.155874	-1.339896	-0.268501
1	2.842363	1.159571	1.111070
1	3.049305	-1.462200	0.916452
1	1.596087	-2.292973	-1.143348
1	0.361883	-0.212502	-2.225988
1	0.489095	1.625413	1.691455
1	0.826817	-0.013564	2.407438
6	-1.098181	-1.362052	0.845388
1	-1.566298	-1.510811	1.826598
1	-0.363944	-2.160828	0.712375
1	-3.090805	-1.831264	-0.001615
1	-1.774737	-1.774393	-1.197077
6	1.599380	1.072280	-0.799297
1	2.427591	1.385809	-1.453911
1	0.911012	1.909869	-0.703717

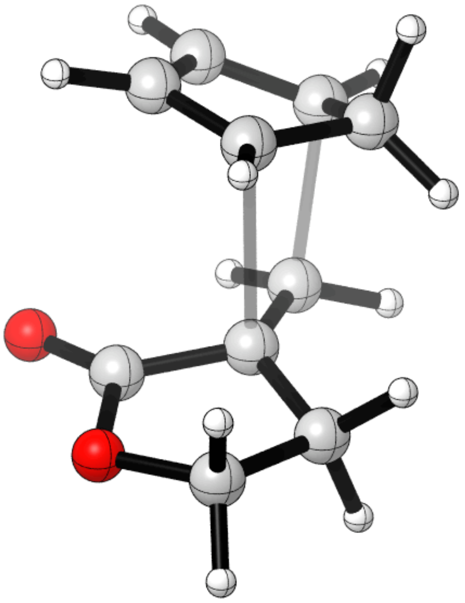

endo 3-methylenedihydrofuran-2(3H)-one cyclopentadiene transition structure
CBS-QB3 PCM Benzene
Energy (0K) = -537.735531
Enthalpy (298K) = -537.724430
Gibbs free Energy (298K) = -537.771192

6	-2.243461	-0.314688	0.618761
6	-2.301954	0.906597	-0.086264
6	-1.506901	0.810165	-1.235828
6	-0.995742	-0.475789	-1.327606
6	0.542630	-0.262353	0.713817
6	-0.510646	-0.170887	1.629784
6	1.215535	0.944197	0.226172
8	0.908494	2.104832	0.374470
8	2.349860	0.589140	-0.463234
6	2.432142	-0.847329	-0.587777
1	-2.945847	-0.563299	1.406151
1	-2.755222	1.817115	0.282396
1	-1.250724	1.635853	-1.885256
1	-0.379490	-0.849303	-2.134468
1	-0.689654	-1.016470	2.286278
1	-0.674530	0.794869	2.090839
6	1.461555	-1.431005	0.454428
1	3.470933	-1.134329	-0.427010
1	2.143384	-1.115906	-1.607729
1	1.998955	-1.706165	1.370266
1	0.968544	-2.334243	0.088242
6	-1.770182	-1.348796	-0.384003
1	-1.237647	-2.205025	0.026871
1	-2.653391	-1.735546	-0.915370

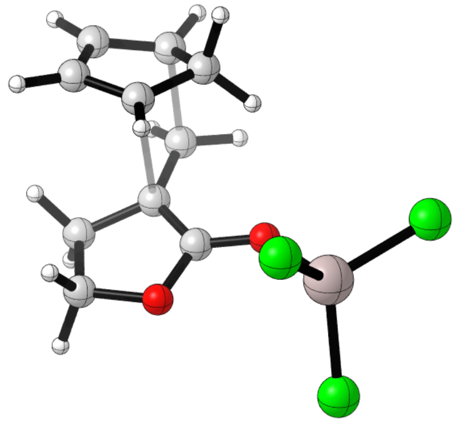

exo anti 3-methylenedihydrofuran-2(3H)-one $AlCl_3$ complex cyclopentadiene transition structure
CBS-QB3 PCM Benzene
Energy (0K) = -2159.287308
Enthalpy (298K) = -2159.269108
Gibbs free Energy (298K) = -2159.336343

6	3.089045	-1.614446	-0.726942
6	4.099760	-0.976718	0.021475
6	3.560919	-0.508235	1.238490
6	2.251627	-0.924952	1.340083
6	1.743366	0.826834	-0.934592
6	2.263666	-0.162964	-1.779792
6	0.415408	0.875448	-0.454824
8	0.165189	1.955443	0.278028
6	1.411497	2.693816	0.490228
1	3.326632	-2.299555	-1.532345
1	5.109363	-0.796734	-0.325200
1	4.084562	0.108681	1.956502
1	1.572017	-0.709539	2.153845
1	1.545862	-0.805800	-2.277487
1	3.112661	0.106091	-2.397354
6	2.423270	2.101237	-0.505579
1	2.583731	2.767292	-1.359963
1	3.399199	1.924560	-0.043467
1	1.183652	3.744970	0.331671
1	1.694744	2.532075	1.531155
8	-0.518616	0.046747	-0.680755
13	-2.191949	-0.216357	-0.001541
17	-1.928724	-0.426950	2.123211
17	-2.810938	-2.050742	-0.918755
17	-3.416479	1.448838	-0.551837
6	1.959571	-1.895797	0.247336
1	0.948026	-1.860506	-0.151946
1	2.107933	-2.911210	0.646612

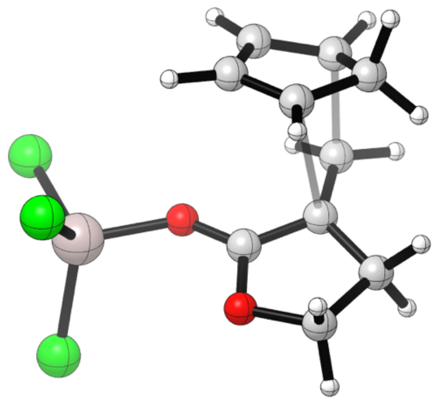

endo anti 3-methylenedihydrofuran-2(3H)-one $AlCl_3$ complex cyclopentadiene transition structure
CBS-QB3 PCM Benzene
Energy (0K) = -2159.285645
Enthalpy (298K) = -2159.267431
Gibbs free Energy (298K) = -2159.334368

6	-3.111543	-1.595732	-0.711953
6	-1.981188	-2.234901	-0.167194
6	-1.666742	-1.661716	1.083121
6	-2.635779	-0.741390	1.407201
6	-1.861191	0.916539	-0.837489
6	-2.315911	-0.077974	-1.717280
6	-0.532225	0.958703	-0.359660
8	-0.264219	2.035328	0.370114
6	-1.498967	2.796127	0.568090
1	-3.696721	-2.052427	-1.501404
1	-1.378631	-2.974557	-0.677670
1	-0.775870	-1.869987	1.658793
1	-2.653948	-0.131149	2.300156
1	-3.206481	0.143729	-2.295954
1	-1.564231	-0.628883	-2.267446
6	-2.516673	2.220269	-0.434714
1	-1.251955	3.842636	0.407070
1	-1.794401	2.645339	1.607189
1	-2.631788	2.878850	-1.302108
1	-3.502925	2.109818	0.020089
8	0.378663	0.108709	-0.588393
13	2.089455	-0.176504	-0.029479
17	3.275604	1.496773	-0.639893
17	2.634482	-1.995232	-1.024335
17	2.002760	-0.425511	2.104624
6	-3.768805	-0.856712	0.440448
1	-4.279677	0.072700	0.193548
1	-4.526062	-1.525625	0.877051

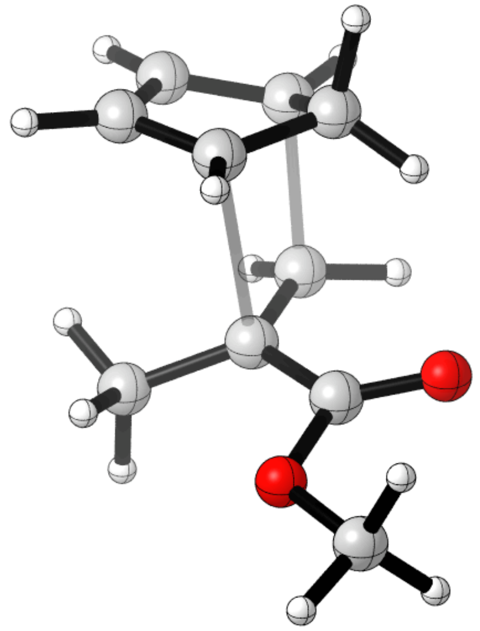

exo s-cis methyl methacrylate cyclopentadiene transition structure
CBS-QB3 PCM Benzene
Energy (0K) = -538.919839
Enthalpy (298K) = -538.906831
Gibbs free Energy (298K) = -538.957647

6	2.232218	-0.731370	-0.580996
6	2.812570	0.325367	0.159954
6	2.048900	0.540352	1.316145
6	1.041273	-0.411248	1.378699
6	-0.193223	0.631773	-0.657201
6	0.744615	0.127639	-1.577585
6	-1.381128	-0.180276	-0.378915
8	-1.570300	-1.328817	-0.750121
8	-2.313223	0.493548	0.350861
6	-3.515961	-0.230986	0.646194
1	2.769159	-1.236720	-1.376241
1	3.622372	0.956379	-0.183472
1	2.172421	1.369637	2.000552
1	0.310603	-0.502315	2.171291
1	0.456536	-0.756332	-2.134698
1	1.318560	0.847027	-2.150503
1	-3.299859	-1.110204	1.256256
1	-4.010232	-0.552432	-0.272059
1	-4.148479	0.464047	1.194986
6	-0.172057	2.094858	-0.294403
1	0.859041	2.456232	-0.248640
1	-0.688481	2.693005	-1.056291
1	-0.656138	2.293200	0.660944
6	1.361008	-1.496758	0.398294
1	1.992926	-2.247070	0.898827
1	0.500462	-2.008401	-0.027990

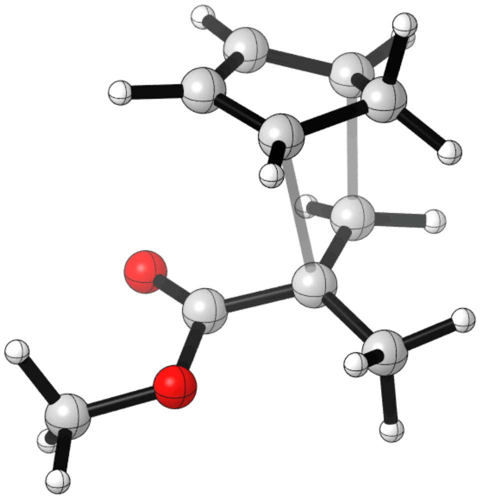

endo s-cis methyl methacrylate cyclopentadiene transition structure
CBS-QB3 PCM Benzene
Energy (0K) = -538.919262
Enthalpy (298K) = -538.906190
Gibbs free Energy (298K) = -538.957246

6	2.369278	-0.175706	0.536516
6	1.874414	-1.484841	0.335991
6	1.097953	-1.505740	-0.830161
6	1.140081	-0.254809	-1.425273
6	-0.171694	0.951379	0.433910
6	0.853040	0.780993	1.385622
6	-1.238537	-0.051295	0.448426
8	-1.243659	-1.069566	1.120033
8	-2.268955	0.266891	-0.385825
6	-3.353308	-0.671193	-0.419833
1	3.184164	0.029153	1.222272
1	1.948109	-2.294540	1.049476
1	0.477519	-2.332988	-1.147279
1	0.639993	0.025175	-2.342704
1	1.412282	1.667392	1.669709
1	0.661982	0.095511	2.200283
1	-3.767569	-0.823001	0.578305
1	-3.021373	-1.634352	-0.813140
1	-4.100160	-0.232639	-1.078956
6	-0.403847	2.271312	-0.265947
1	-0.744470	2.147761	-1.294861
1	-1.175730	2.851653	0.254605
1	0.503621	2.878920	-0.269039
6	2.282636	0.505119	-0.818820
1	2.183405	1.588208	-0.805506
1	3.199043	0.263831	-1.379421

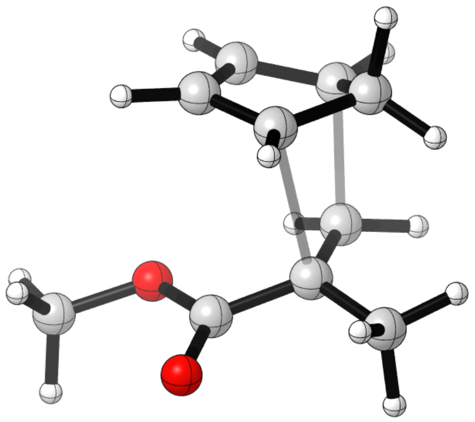

endo s-trans methyl methacrylate cyclopentadiene transition structure
CBS-QB3 PCM Benzene
Energy (0K) = -538.918780
Enthalpy (298K) = -538.905684
Gibbs free Energy (298K) = -538.956793

6	2.086830	-0.672911	0.741147
6	1.389821	-1.778538	0.202658
6	0.859855	-1.418253	-1.041965
6	1.264368	-0.125961	-1.350491
6	-0.083337	1.052465	0.422524
6	0.626772	0.471455	1.492590
6	-1.364978	0.490838	-0.023038
8	-2.126303	1.024310	-0.809780
8	-1.653158	-0.710737	0.553177
6	-2.882324	-1.318759	0.132303
1	2.767476	-0.774654	1.579229
1	1.162997	-2.691308	0.737725
1	0.160347	-2.009798	-1.617525
1	1.020334	0.409841	-2.257914
1	1.279722	1.130395	2.057606
1	0.124036	-0.270247	2.096453
1	-2.872995	-1.510386	-0.942519
1	-3.732471	-0.676037	0.366926
1	-2.950905	-2.254723	0.683446
6	0.155382	2.484041	-0.001588
1	0.021480	2.624469	-1.075157
1	-0.561936	3.150097	0.493204
1	1.154657	2.821289	0.279708
6	2.409727	0.219012	-0.443913
1	2.551255	1.274892	-0.226988
1	3.338999	-0.152755	-0.902728

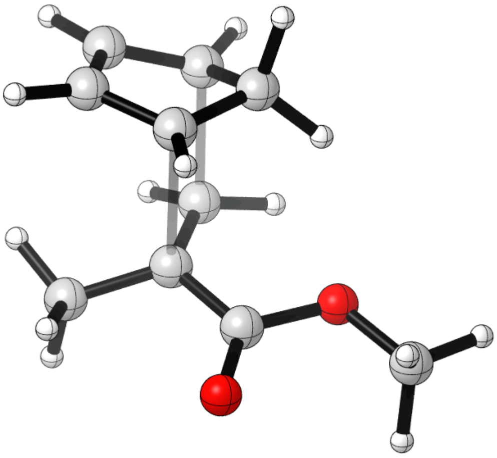

exo s-trans methyl methacrylate cyclopentadiene transition structure
CBS-QB3 PCM Benzene
Energy (0K) = -538.918663
Enthalpy (298K) = -538.905601
Gibbs free Energy (298K) = -538.956642

6	-1.869019	-1.074444	0.729792
6	-2.788027	-0.175947	0.137931
6	-2.278636	0.243099	-1.099440
6	-1.091196	-0.428660	-1.353352
6	0.140717	0.843411	0.522123
6	-0.477598	0.095436	1.542286
6	1.455618	0.485329	-0.023042
8	2.118025	1.186334	-0.766537
8	1.917174	-0.727512	0.409901
6	3.210297	-1.109599	-0.082154
1	-2.144746	-1.709982	1.564263
1	-3.665240	0.229881	0.625409
1	-2.695570	1.032369	-1.711289
1	-0.483987	-0.318984	-2.241841
1	0.095138	-0.701769	1.999416
1	-1.108293	0.637329	2.238368
1	3.964673	-0.376273	0.206825
1	3.203082	-1.197899	-1.170192
1	3.424088	-2.073980	0.374826
6	-0.307863	2.257191	0.259658
1	-1.394937	2.336729	0.344379
1	0.125075	2.943555	0.998847
1	0.002324	2.600376	-0.726784
6	-1.006636	-1.589158	-0.408440
1	-1.526555	-2.447082	-0.863006
1	-0.002597	-1.904800	-0.138300

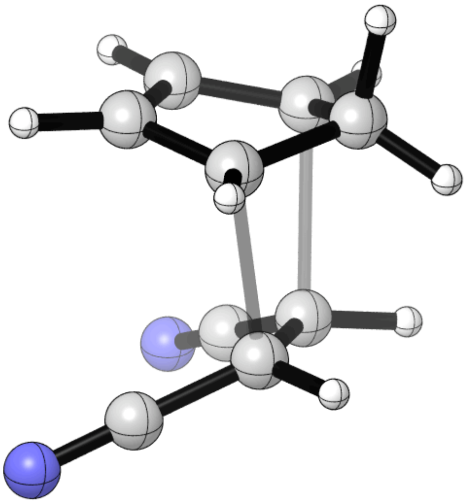

endo maleonitrile cyclopentadiene transition structure
CBS-QB3 PCM Benzene
Energy (0K) = -456.361993
Enthalpy (298K) = -456.351486
Gibbs free Energy (298K) = -456.397016

6	1.435652	1.153710	-0.046415
6	1.183862	0.697781	1.257123
6	1.183861	-0.697781	1.257123
6	1.435651	-1.153711	-0.046415
6	-0.519330	-0.708104	-0.976791
6	-0.519330	0.708104	-0.976792
1	1.596851	2.191571	-0.307689
1	0.906216	1.324313	2.093567
1	0.906215	-1.324312	2.093568
1	1.596849	-2.191572	-0.307689
1	-0.242411	-1.203202	-1.899181
1	-0.242411	1.203202	-1.899181
6	2.044617	-0.000001	-0.802164
1	3.127264	-0.000001	-0.608519
1	1.903399	0.000000	-1.881944
6	-1.420850	-1.460004	-0.166936
7	-2.140834	-2.091972	0.480449
6	-1.420849	1.460005	-0.166937
7	-2.140833	2.091973	0.480449

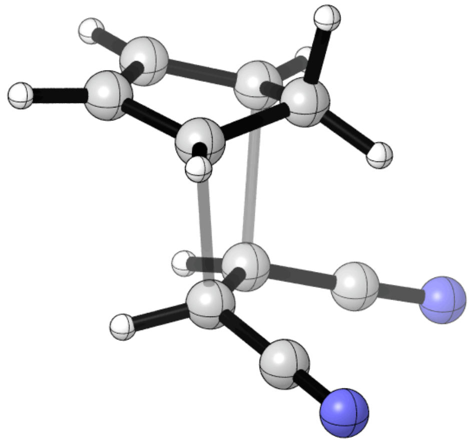

exo maleonitrile cyclopentadiene transition structure
CBS-QB3 PCM Benzene
Energy (0K) = -456.361589
Enthalpy (298K) = -456.351074
Gibbs free Energy (298K) = -456.396658

6	1.216944	-1.152940	0.445292
6	2.259931	-0.697189	-0.380032
6	2.259975	0.697056	-0.380084
6	1.217021	1.152933	0.445209
6	-0.484111	0.709624	-0.894601
6	-0.484138	-0.709638	-0.894578
1	1.056439	-2.189380	0.711695
1	2.880854	-1.326168	-1.004404
1	2.880938	1.325950	-1.004503
1	1.056580	2.189402	0.711537
1	-0.083757	-1.207654	-1.766831
6	0.828261	0.000044	1.332567
1	1.503509	0.000052	2.200796
1	-0.192472	0.000104	1.710291
1	-0.083727	1.207600	-1.766876
6	-1.490622	1.456500	-0.215075
7	-2.286638	2.086737	0.339037
6	-1.490698	-1.456463	-0.215064
7	-2.286755	-2.086661	0.339033

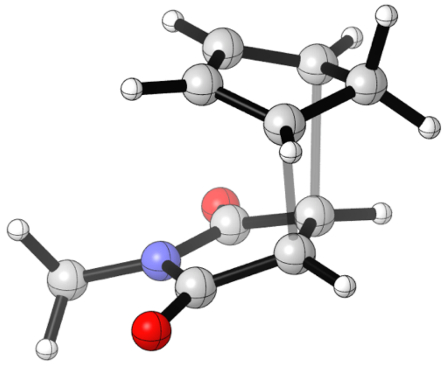

endo N-methylmaleimide cyclopentadiene transition structure
CBS-QB3 gas phase

Energy (0K) = -591.851931
Enthalpy (298K) = -591.839703
Gibbs free energy (298K) = -591.889599

6	1.883885	1.154046	0.183366
6	1.335097	0.696103	1.387574
6	1.334795	-0.703166	1.384814
6	1.883223	-1.156751	0.178747
6	0.213560	-0.697770	-1.215968
6	0.214225	0.699265	-1.214866
6	-0.975972	-1.158498	-0.443693
6	-0.973960	1.161377	-0.442108
8	-1.381109	2.287546	-0.266488
8	-1.388261	-2.282949	-0.268785
1	2.096037	2.193129	-0.031301
1	0.872582	1.323292	2.137770
1	0.872092	-1.333182	2.132537
1	2.095410	-2.195074	-0.039565
1	0.623731	-1.335911	-1.984277
1	0.626006	1.338734	-1.981190
7	-1.573543	0.000833	0.069242
6	-2.773401	-0.000862	0.885869
1	-3.245728	0.976159	0.790383
1	-3.444797	-0.786109	0.537472
1	-2.536998	-0.184155	1.937387
6	2.655733	-0.000367	-0.409979
1	2.763611	0.001782	-1.493773
1	3.664705	-0.001531	0.029507

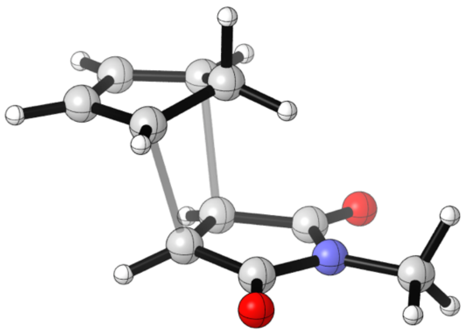

exo N-methylmaleimde cyclopentadiene transition structure
CBS-QB3 gas phase
Energy (0K) = -591.847036
Enthalpy (298K) = -591.834815
Gibbs free energy (298K) = -591.884553

6	1.699711	-1.158017	0.533466
6	2.824949	-0.701920	-0.167530
6	2.827596	0.696822	-0.164951
6	1.704752	1.154349	0.538387
6	0.178031	0.699252	-1.046338
6	0.177836	-0.698485	-1.046436
6	-1.079325	1.160504	-0.385141
6	-1.081791	-1.157023	-0.385898

8	-1.491034	-2.284315	-0.222043
8	-1.482276	2.290059	-0.221717
1	1.494349	-2.193715	0.770121
1	3.507593	-1.328903	-0.726357
1	3.512492	1.323294	-0.721575
1	1.501574	2.189916	0.777338
1	0.637919	1.338639	-1.782369
1	0.635205	-1.336896	-1.784927
7	-1.749690	0.001026	0.035653
6	-3.009561	-0.001286	0.756030
1	-3.700074	-0.702763	0.286328
1	-2.870647	-0.296531	1.799753
1	-3.414025	1.009334	0.717243
6	1.219012	-0.002433	1.371017
1	0.165856	-0.000287	1.641596
1	1.796800	-0.005797	2.307729

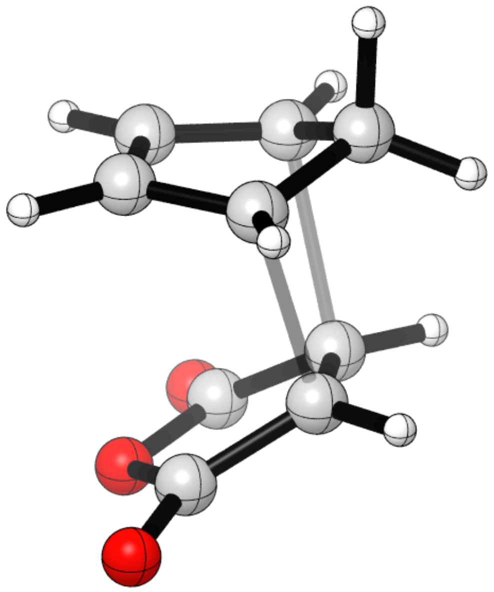

endo maleic anhydride cyclopentadiene transition structure
CBS-QB3 PCM Benzene
Energy (0K) = -572.499184
Enthalpy (298K) = -572.488979
Gibbs free Energy (298K) = -572.533983

6	-0.030849	-1.613023	-1.154147
6	1.262907	-1.309765	-0.697784
6	1.262907	-1.309765	0.697784
6	-0.030849	-1.613023	1.154147
6	-1.085820	0.259897	0.700792
6	-1.085820	0.259897	-0.700792
6	-0.116052	1.280857	1.141597
6	-0.116052	1.280857	-1.141597
8	0.154291	1.692644	-2.231655
8	0.154291	1.692644	2.231655
1	-0.282290	-1.782924	-2.192850
1	2.089302	-1.003693	-1.325023
1	2.089302	-1.003693	1.325023
1	-0.282290	-1.782924	2.192850

1	-1.918866	0.010657	1.340212
1	-1.918866	0.010657	-1.340212
6	-0.754977	-2.263330	0.000000
1	-1.840088	-2.175026	0.000000
1	-0.502823	-3.333911	0.000000
8	0.533198	1.767868	0.000000

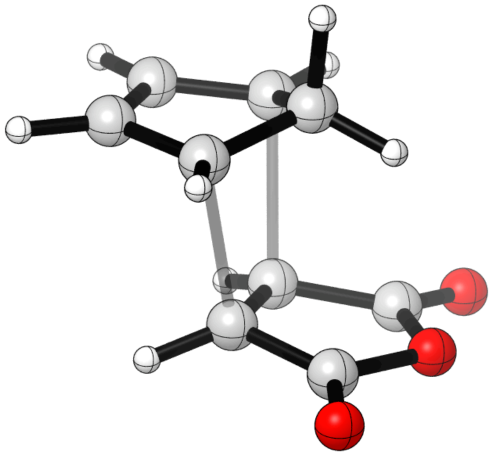

exo maleic anhydride cyclopentadiene transition structure
CBS-QB3 PCM Benzene
Energy (0K) = -572.495570
Enthalpy (298K) = -572.485294
Gibbs free Energy (298K) = -572.530713

6	-0.445094	1.440672	1.154557
6	0.385088	2.478468	0.697654
6	0.385088	2.478468	-0.697654
6	-0.445094	1.440672	-1.154557
6	0.947824	-0.225107	-0.701299
6	0.947824	-0.225107	0.701299
6	0.142193	-1.382689	-1.140684
6	0.142193	-1.382689	1.140684
8	-0.076713	-1.816851	2.233675
8	-0.076713	-1.816851	-2.233675
1	-0.706427	1.272635	2.191147
1	1.016466	3.093115	1.325804
1	1.016466	3.093115	-1.325804
1	-0.706427	1.272635	-2.191147
1	1.738609	0.129400	-1.342080
1	1.738609	0.129400	1.342080
6	-1.336648	1.065482	0.000000
1	-1.747706	0.058925	0.000000
1	-2.188817	1.761026	0.000000
8	-0.409203	-1.983706	0.000000

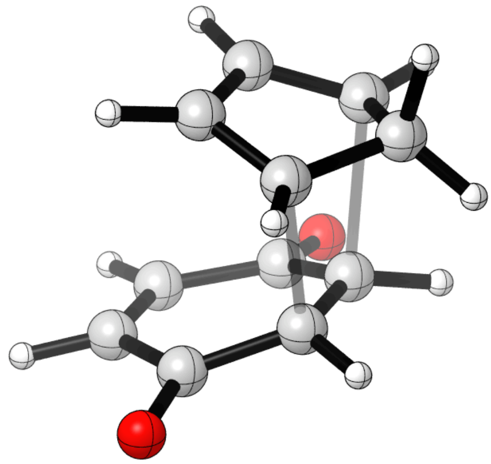

endo benzoquinone cyclopentadiene transition structure
CBS-QB3 PCM Benzene
Energy (0K) = -574.577891
Enthalpy (298K) = -574.566619
Gibbs free Energy (298K) = -574.613840

6	-1.703316	-1.152211	0.037804
6	-1.378463	-0.696802	1.329245
6	-1.378465	0.696801	1.329247
6	-1.703320	1.152212	0.037808
6	0.126373	0.706278	-1.059923
6	0.126371	-0.706277	-1.059922
6	1.110999	1.457283	-0.255289
8	1.294752	2.660376	-0.382443
1	-1.873794	-2.191201	-0.212584
1	-1.059621	-1.325782	2.149617
1	-1.059623	1.325780	2.149619
1	-1.873798	2.191202	-0.212580
1	-0.224138	1.240678	-1.935044
1	-0.224138	-1.240677	-1.935044
6	1.898524	0.670438	0.730666
6	1.898524	-0.670438	0.730666
1	2.515396	1.248138	1.411218
1	2.515396	-1.248138	1.411219
6	1.110998	-1.457282	-0.255288
8	1.294751	-2.660376	-0.382443
6	-2.384031	0.000000	-0.662666
1	-2.332167	0.000002	-1.749866
1	-3.444706	-0.000002	-0.371549

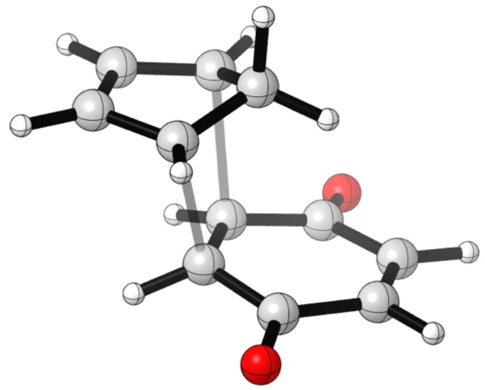

exo benzoquinone cyclopentadiene transition structure
CBS-QB3 PCM Benzene
Energy (0K) = -574.572741
Enthalpy (298K) = -574.561386
Gibbs free Energy (298K) = -574.609013

6	1.540457	-1.152813	0.496308
6	2.603287	-0.696643	-0.306354
6	2.603300	0.696645	-0.306337
6	1.540489	1.152814	0.496345
6	-0.099726	-0.706663	-0.906001
6	-0.099736	0.706666	-0.906008
6	-1.192767	-1.456597	-0.249446
8	-1.320637	-2.668881	-0.358512
1	1.368827	-2.190681	0.749959
1	3.230329	-1.326250	-0.923930
1	3.230354	1.326255	-0.923898
1	1.368860	2.190680	0.750006
1	0.354013	-1.244713	-1.727189
1	0.354021	1.244718	-1.727186
6	-2.173941	-0.669784	0.548665
6	-2.173942	0.669783	0.548663
1	-2.907125	-1.251063	1.097811
1	-2.907127	1.251063	1.097807
6	-1.192770	1.456598	-0.249453
8	-1.320649	2.668882	-0.358522
6	1.155790	-0.000006	1.387074
1	0.139254	0.000003	1.770697
1	1.836237	-0.000030	2.251452

Cartesian Coordinates of B3LYP-D3/6-311++G(d.p) Optimised Structures and CCSD(T) Energies
Endo s-cis-acrolein butadiene transition structure
CCSD(t)/6-311++G(d.p) CPCM Benzene
Energy (0K) = -346.9323229
Optimisation and Frequency calculation at B3LYP-D3/6-311++G(d.p) CPCM Benzene
Free energy correction: (418K) = 0.099443
Gibbs free energy (418K) = -346.8328799

C	-1.57961	1.10659	-0.13506
H	-1.91556	1.99286	-0.66726
H	-1.88059	1.08316	0.90838
C	-1.51177	-0.10024	-0.84127
H	-1.58927	-0.07644	-1.92494

C	-1.08983	-1.30486	-0.24163
H	-0.88623	-2.14758	-0.89680
C	-0.70648	-1.38850	1.07500
H	-0.24413	-2.29099	1.46213
H	-1.00766	-0.65787	1.81462
C	0.32702	1.62770	0.29649
H	0.06163	2.47134	0.92840
H	0.59539	1.87618	-0.72569
C	1.04480	0.57001	0.87559
H	1.16957	0.48699	1.95093
C	1.77381	-0.34956	0.02055
H	2.39188	-1.11129	0.53886
O	1.71967	-0.32416	-1.21082

Exo s-cis-acrolein butadiene transition structure
CCSD(t)/6-311++G(d.p) CPCM Benzene
Energy (0K) = -346.9300378
Optimisation and Frequency calculation at B3LYP-D3/6-311++G(d.p) CPCM Benzene
Free energy correction: (418K) = 0.098792
Gibbs free energy (418K) = -346.8312458

C	-1.05131	-1.21574	-0.70568
H	-1.27894	-2.26582	-0.87254
H	-0.33419	-0.80289	-1.41111
C	-2.05958	-0.40301	-0.17788
H	-2.95080	-0.87807	0.22695
C	-1.85725	0.96526	0.10161
H	-2.58789	1.46764	0.73281
C	-0.70348	1.63345	-0.23455
H	-0.52512	2.64368	0.12080
H	-0.05182	1.28707	-1.02607
C	0.35266	-1.31247	0.74567
H	0.95831	-1.98537	0.14331
H	-0.22300	-1.78901	1.53254
C	0.90337	-0.04710	1.00096
C	1.97090	0.47589	0.16608
H	2.46602	1.39741	0.53806
O	2.31871	-0.01973	-0.90749
H	0.64584	0.50552	1.89786

Endo s-trans-acrolein butadiene transition structure
CCSD(t)/6-311++G(d.p) CPCM Benzene
Energy (0K) = -346.9295644
Optimisation and Frequency calculation at B3LYP-D3/6-311++G(d.p) CPCM Benzene
Free energy correction: (418K) = 0.099159
Gibbs free energy (418K) = -346.8304054

C	-1.04260	-1.45027	-0.25733
H	-0.87034	-2.20927	-1.01735
C	-0.17864	-1.38421	0.81630
H	0.65112	-2.07683	0.90809
H	-0.42729	-0.83134	1.71333
C	-0.19668	1.63206	-0.07845
H	-0.48842	2.50702	0.49553

H	-0.27458	1.75813	-1.15508
C	0.84391	0.83526	0.41811
H	1.16488	0.93494	1.45190
C	1.77243	0.14379	-0.47416
H	1.41237	0.04129	-1.52304
O	2.87273	-0.28730	-0.15157
C	-1.98021	-0.43822	-0.53629
H	-2.48817	-0.46785	-1.49795
C	-2.05495	0.73435	0.21843
H	-2.75004	1.50989	-0.09338
H	-1.89100	0.69585	1.29088

Exo s-trans-acrolein butadiene transition structure
CCSD(t)/6-311++G(d.p) CPCM Benzene
Energy (0K) = -346.9280605
Optimisation and Frequency calculation at B3LYP-D3/6-311++G(d.p) CPCM Benzene
Free energy correction: (418K) = 0.0988
Gibbs free energy (418K) = -346.8292305

C	1.77213	-0.93053	0.63709
H	2.36807	-1.83911	0.66834
H	1.18013	-0.76003	1.53044
C	2.24623	0.15997	-0.09392
H	3.10670	0.01502	-0.74381
C	1.48716	1.33451	-0.25917
H	1.78946	2.03066	-1.03887
C	0.25964	1.51084	0.34429
H	-0.39717	2.32614	0.06001
H	0.01758	1.02714	1.28285
C	0.05534	-1.53232	-0.41071
H	-0.20818	-2.27963	0.33580
H	0.59039	-1.92635	-1.26732
C	-0.83485	-0.46577	-0.61010
C	-1.99249	-0.29159	0.26285
H	-1.96086	-0.89389	1.20173
O	-2.94755	0.44065	0.03806
H	-0.86468	0.06413	-1.55562

Endo 3-methylenedihydrofuran-2(3H)-one butadiene transition structure
CCSD(t)/6-311++G(d.p) CPCM Benzene
Energy (0K) = -499.0744232
Optimisation and Frequency calculation at B3LYP-D3/6-311++G(d.p) CPCM Benzene
Free energy correction: (418K) = 0.135438
Gibbs free energy (418K) = -498.9389852

C	-2.47071	0.97321	-0.14013
H	-3.16691	1.44710	-0.82757
H	-2.11037	1.63402	0.64342
C	-2.62051	-0.39103	0.12355
H	-3.29677	-0.96943	-0.50130
C	-1.72127	-1.09591	0.94617
H	-1.75001	-2.18189	0.91612
C	-0.67421	-0.48151	1.59462
H	0.08228	-1.07566	2.09655

H	-0.69476	0.57151	1.84439
C	-0.70316	1.09450	-1.18351
C	0.38557	0.63656	-0.43908
H	-1.00825	0.47524	-2.02078
H	-0.79751	2.16669	-1.33760
C	0.99687	-0.67478	-0.71705
O	0.58761	-1.57243	-1.42306
O	2.20648	-0.75489	-0.06334
C	2.39918	0.39564	0.78813
H	3.44154	0.70621	0.69229
H	2.21370	0.08833	1.82326
C	1.39027	1.46576	0.31934
H	1.87155	2.19168	-0.34965
H	0.97067	2.03008	1.15978

Exo 3-methylenedihydrofuran-2(3H)-one butadiene transition structure
CCSD(t)/6-311++G(d.p) CPCM Benzene
Energy (0K) = -499.0749321
Optimisation and Frequency calculation at B3LYP-D3/6-311++G(d.p) CPCM Benzene
Free energy correction: (418K) = 0.135209
Gibbs free energy (418K) = -498.9397231

C	2.49243	0.88582	0.18604
H	3.16837	1.42119	0.84783
H	2.14157	1.47600	-0.65497
C	2.67095	-0.49069	0.01724
H	3.36801	-1.01616	0.66644
C	1.79483	-1.25424	-0.77885
H	1.87500	-2.33857	-0.73352
C	0.72302	-0.69331	-1.44029
H	-0.00600	-1.32135	-1.94253
H	0.69306	0.35385	-1.71242
C	0.68683	1.12733	1.14818
C	-0.32372	0.31155	0.63492
H	1.06605	0.89254	2.13783
H	0.61697	2.18647	0.91396
C	-1.36080	0.81872	-0.27904
O	-1.39362	1.86803	-0.88912
O	-2.37301	-0.10659	-0.36265
C	-0.82732	-0.96290	1.25392
H	-0.06871	-1.75205	1.29243
H	-1.16654	-0.78087	2.28288
C	-2.01089	-1.32142	0.33482
H	-2.89634	-1.66908	0.87045
H	-1.73034	-2.06868	-0.41589

Endo N-methylmaleimide butadiene transition structure
CCSD(t)/6-311++G(d.p) CPCM Benzene
Energy (0K) = -553.1234238
Optimisation and Frequency calculation at B3LYP-D3/6-311++G(d.p) CPCM Benzene
Free energy correction: (418K) = 0.147685
Gibbs free energy (418K) = -552.9757388

C	1.97925	1.43135	0.03426

H	1.81821	2.50454	0.03603
H	2.64174	1.05839	-0.73859
C	1.73473	0.69101	1.18077
H	1.32213	1.20029	2.04823
C	1.72700	-0.71740	1.17637
H	1.30873	-1.22753	2.04067
C	1.96344	-1.45362	0.02555
H	1.79061	-2.52498	0.02114
H	2.63004	-1.08349	-0.74514
C	0.30717	0.69946	-1.32256
C	0.29895	-0.69638	-1.32574
H	0.66370	1.33771	-2.11970
H	0.64778	-1.33473	-2.12619
C	-0.79471	1.16424	-0.42976
C	-0.80918	-1.14924	-0.43448
N	-1.32967	0.00809	0.15538
C	-2.43472	0.00207	1.09777
H	-2.60580	1.03112	1.41618
H	-2.18631	-0.62541	1.95691
H	-3.33910	-0.39315	0.62584
O	-1.21834	-2.27846	-0.22305
O	-1.18362	2.29993	-0.21647

Exo N-methylmaleimide butadiene transition structure
CCSD(t)/6-311++G(d.p) CPCM Benzene
Energy (0K) = -553.1169201
Optimisation and Frequency calculation at B3LYP-D3/6-311++G(d.p) CPCM Benzene
Free energy correction: (418K) = 0.147023
Gibbs free energy (418K) = -552.9698971

C	-1.69866	-1.44768	-0.58140
H	-1.67625	-2.52252	-0.43421
H	-0.97228	-1.07270	-1.29386
C	-2.83228	-0.71277	-0.27441
H	-3.67806	-1.22419	0.18043
C	-2.83726	0.69637	-0.27673
H	-3.68673	1.20329	0.17626
C	-1.70899	1.43822	-0.58606
H	-1.69359	2.51347	-0.44109
H	-0.97888	1.06617	-1.29626
C	-0.26758	-0.69753	1.02994
C	-0.27108	0.69812	1.02913
C	0.97862	-1.15012	0.33886
C	0.97069	1.15961	0.33694
N	1.61198	0.00477	-0.13564
O	1.37613	2.29801	0.17894
O	1.39837	-2.28376	0.18159
C	2.86134	-0.00278	-0.87716
H	3.13911	1.03472	-1.06775
H	2.73880	-0.53814	-1.82230
H	3.64445	-0.49607	-0.29473
H	-0.73869	-1.33498	1.76362
H	-0.74659	1.33494	1.76047
